# Safety and immunogenicity of multivalent SARS-CoV-2 protein vaccines: a randomized phase 3 trial

**DOI:** 10.1016/j.eclinm.2023.102195

**Published:** 2023-09-08

**Authors:** Suad Hannawi, Lixin Yan, Linda Saifeldin, Alaa Abuquta, Ahmad Alamadi, Sally A. Mahmoud, Aala Hassan, Miaomiao Zhang, Cuige Gao, Yuanxin Chen, Wenlin Gai, Liangzhi Xie

**Affiliations:** a Internal Medicine Department, Al Kuwait-Dubai (ALBaraha) Hospital, Dubai, United Arab Emirates; b Beijing Engineering Research Center of Protein and Antibody, Sinocelltech Ltd., Beijing, China; c General Surgery Department, Al Kuwait-Dubai (ALBaraha) Hospital, Dubai, United Arab Emirates; d Ear, Nose and Throat Department (ENT), Al Kuwait-Dubai (ALBaraha) Hospital, Dubai, United Arab Emirates; e Biogenix Labs, G42 Healthcare, Dubai, United Arab Emirates

**Keywords:** Safety, Immunogenicity, SARS-CoV-2, Tetravalent vaccine, Booster

## Abstract

**Background:**

COVID-19 vaccines that offer broad-spectrum protection are needed. We aimed to evaluate the safety and immunogenicity of multivalent vaccines, SCTV01E and SCTV01C, and compare them with an inactivated vaccine.

**Methods:**

In the phase 3 trial (ClinicalTrials.gov: NCT05323461), adult participants previously vaccinated with Sinopharm's inactivated SARS-CoV-2 vaccine (BBBIP-CorV) were assigned to receive one booster dose of BBBIP-CorV, 20 μg SCTV01C, or 30 μg SCTV01E. The primary endpoint was to evaluate the geometric mean titers (GMT) of neutralizing antibody (nAb) against the Delta and Omicron BA.1 variants on day 28 after injection. Additional endpoints included GMTs of nAb against Delta (B.1.617.2) and Omicron BA.1 variants on day 180, GMTs against BA.5 on day 28, as well as solicited adverse events (AEs) within seven days, unsolicited AEs within 28 days, and serious AEs, AEs of special interest within 180 days after vaccination.

**Findings:**

Between May 30, 2022 and October 28, 2022, a total of 1351 participants were randomized to BBBIP-CorV, SCTV01C, or SCTV01E in a 1:1:1 ratio, with immunogenicity assessments performed on the first 300 participants. For BBBIP-CorV, SCTV01C, and SCTV01E groups, the day 28 GMTs of neutralizing antibody against Omicron BA.1 were a 2.38-, 19.37-, and 28.06-fold increase from baseline; the GMTs against Omicron BA.5 were 2.07-, 15.89- and 21.11-fold increases; the GMTs against Delta variants were 1.97-, 12.76-, and 15.88-fold increases, respectively. The day 28 geometric mean ratio (GMR) of SCTV01C/BBIBP-CorV for Omicron BA.1 was 6.49 (95% CI: 4.75, 8.88), while the GMR of SCTV01E/BBIBP-CorV was 9.56 (95% CI: 6.85, 13.33). For the Delta variant, the day 28 GMR of SCTV01C/BBIBP-CorV was 6.26 (95% CI: 4.78, 8.19), and the day 28 GMR of SCTV01E/BBIBP-CorV was 7.26 (95% CI: 5.51, 9.56). On Day 180, the GMTs against Omicron BA.1 were 2.80-, 9.51-, and 15.56-fold increase from baseline, while those against Delta were 1.58-, 5.49-, and 6.63-fold for BBBIP-CorV, SCTV01C, and SCTV01E groups, respectively. Subgroup analyses showed that SCTV01C and SCTV01E induced uniformly high GMTs against both BA.1 and BA.5, demonstrating its superiority over BBIBP-CorV, regardless of baseline GMT levels. Safety and reactogenicity were similar among the three vaccines. Most AEs were Grade 1 or 2. There were 15 ≥Grade 3 AEs: 6 in the BBIBP-CorV group, 4 in the SCTV01C group and 5 in the SCTV01E group. No SAE was reported and one grade 1 AESI (Bell's palsy) was observed in SCTV01C group.

**Interpretation:**

A booster dose of the tetravalent vaccine SCTV01E consistently induced high neutralizing antibody responses against Omicron BA.1, BA.5, and Delta variants, demonstrating superiority over inactivated vaccine. There is evidence to suggest that SCTV01E may have GMT superiority over bivalent vaccine SCTV01C against Delta, BA.1 and BA.5 variants.

**Funding:**

This study was sponsored by Sinocelltech Ltd., and funded by the 10.13039/501100012401Beijing Science and Technology Planning Project [Z221100007922012] and the 10.13039/501100012166National Key Research and Development Program of China [2022YFC0870600].

**Research in context**

**Evidence before this study**

We searched PubMed until April 10th, 2023, using terms "COVID-19," "SARS-CoV-2," "vaccine," "multivalent," and "clinical trial." Multivalent anti-coronavirus vaccines are being explored or studied, including bivalent recombinant protein vaccines for original and Beta variants (Sanofi, Medigen), bivalent protein vaccine for Beta and Delta variants (Livzon), protein vaccine for original, Beta, and Kappa variants (Sinopharm), bivalent mRNA vaccine encoding original and Beta variants (Moderna), bivalent mRNA vaccine for original and Omicron B.1.1.529 (Moderna, Pfizer) and bivalent mRNA vaccine encoding original and Omicron BA.4/5 (Moderna, Pfizer).

**Added value of this study**

This study demonstrated that administering a booster dose of SCTV01E or SCTV01C is safe and generates significantly higher levels of neutralizing antibody against the Delta and Omicron BA.1 and BA.5 variants compared to inactivated viral vaccine. The findings of this investigation suggest that utilizing a tetravalent recombinant protein could be an effective strategy in addressing both current and potential future epidemiological challenges of COVID-19, particularly in situations where multiple major variants are prevalent simultaneously.

**Implications of all the available evidence**

SCTV01E booster has demonstrated significant potential as a vaccine tool in combating the current COVID-19 pandemic. The current investigation suggests the multivalent vaccine design is a promising strategy for developing broad-spectrum anti-coronavirus vaccine.

## Introduction

As of April 2023, the SARS-CoV-2 infections remain a persistent global health threat. The World Health Organization reports over 760 million confirmed cases of COVID-19 worldwide, with nearly 6.9 million deaths occurring over the past three years.^1^ SARS-CoV-2 has the propensity to mutate and the new variants are associated with higher transmissibility, and immune escape to vaccines as well as to treatment options such as monoclonal antibodies and antivirals. The recent surges globally have been associated with the spread of highly transmissible SARS-CoV-2 Omicron sublineages BA.1, BA.4/5, XBB.1 and BQ.1 that have been associated with increases in COVID-19 case rates and increased reinfection risk.^2^, ^3^, ^4^, ^5^ In addition, existing evidence shows the waning protection of COVID-19 primary and booster vaccination and the reduced effectiveness of the monovalent vaccines developed based on the original SARS-CoV-2 strain against COVID-19.^6^, ^7^, ^8^, ^9^, ^10^ Consequently, the development of an Omicron-effective vaccine and broad-spectrum anti-coronavirus vaccine is crucial to combat the SARS-CoV-2 infections.

The multivalent vaccine may provide broad-spectrum protection, as each variant could contribute with unique neutralizing epitope(s) that expand the repertoire of neutralizing antibody, and highly frequent mutations present in current circulating variants are likely to re-appear in future variants. For example, the Alpha variant had the greatest identity rate with the Omicron variant (99.63%).^11^ Mutations such as T95I, G142D, K417N, T478K, N501Y, P681H, delta69/70 and delta145 are shared by Alpha, Beta Delta, Gamma and Omicron variants, and associated with increased transmissibility.^12^ Moderna and Pfizer have released promising immunogenicity data on their bivalent booster candidates. These include mRNA-1273.211 (original and Beta variant),^13^ mRNA-1273.214 (original and Omicron B.1.1.529),^14^ mRNA-1237.222 (original and Omicron BA.4/5),^15^ and Pfizer bivalent mRNA vaccines (original and Omicron BA.1 or BA.4/5).^16^ These vaccine candidates have demonstrated immunogenicity superiority against Omicron and maintained non-inferior status against ancestral strains compared to their monovalent progenitor vaccines.^13^, ^14^, ^15^, ^16^

SCTV01E is a recombinant protein SARS-CoV-2 vaccine with a tetravalent formulation containing spike protein ectodomain (S-ECD) of Alpha, Beta, Delta and Omicron BA.1 variants. It is manufactured by the same process as the progenitor vaccine SCTV01C which is a bivalent design comprising equal amounts of S-ECD of Alpha and Beta variants. Both vaccine candidates are adjuvanted with a squalene-based oil-in-water emulsion SCT-VA02B and possess a trimerization auxiliary domain (T4-Foldon) to stabilize the trimeric protein conformation and boost the immune responses. The thermostability test showed that both SCTV01C and SCTV01E remained stable at 25 °C for over six months, making them suitable for remote and resource-poor settings.^17^^,^^18^

Three safety and immunogenicity phase 1/2 trials had assessed SCTV01C given as two-dose primary series in vaccine naïve people (NCT05148091) and a booster dose in people previously vaccinated with mRNA vaccine (NCT05043311) and inactivated vaccine (NCT05043285). SCTV01C demonstrated favorable safety profiles in a total of 922 participants, and both primary series and one booster dose of SCTV01C induced significant neutralizing antibody responses against antigen-matched variants Alpha and Beta and cross-strain protection against Delta and Omicron variants in these studies.^19^, ^20^, ^21^ Both SCTV01C (granted Emergency Use Authorization on December 2, 2022) and SCTV01E (granted Emergency Use Authorization on March 22, 2023) have been recommended by the National Health Commission of the People's Republic of China as a booster dose for individuals who received an inactivated vaccine as their primary series, as well as a primary dose for individuals who have already been infected.

Herein, we present the results of a phase III trial that evaluated the safety and immunogenicity of SCTV01C and SCTV01E in people that had previously received two or three doses of inactivated vaccines of COVID-19 and compared with inactivated vaccine.

## Methods

### Study design and participants

The study conducted at Al Kuwait Hospital and Emirates Health Services in Dubai, United Arab Emirates (UAE) was a randomized, double-blind, positive-controlled phase 3 booster study. Eligible participants were ≥18 years old and had previously received 2 or 3 doses of BBIBP-CorV (Sinopharm inactivated COVID-19 vaccine) with or without COVID-19 history, or previously vaccinated with 1 dose of BBIBP-CorV and previously diagnosed with COVID-19, 3–24 months earlier. Health status and history of infections with SAR-CoV-2 and/or other coronaviruses were assessed based on the medical history, clinical laboratory findings, vital signs and physical examination during the screening visits. Participants who tested positive for SARS-Cov-2 nucleic acid detection during the screening period, individuals with fever within 3 days, and those with a history of severe acute respiratory syndrome (SARS), Middle East Respiratory Syndrome (MERS) were excluded (Supplementary Materials 1).

### Ethics

This clinical trial adhered to the ethical requirements of Good Clinical Practice and the Declaration of Helsinki. The Ministry of Health and Prevention reviewed and approved the protocol and its amendments (reference number: RCMOHP/CT1/0123/2021). All trial participants voluntarily enrolled and provided informed consent before undergoing any study procedures.

### Randomization and masking

The Interactive Network Response System (IWRS) was used to randomize eligible participants prior to study vaccination. The randomization codes were generated using block randomization with SAS software (version 9.4). The participants were stratified by age (18–54 years and ≥55 years), the number of previous BBIBP-CorV injections, and the interval between previous vaccination and study vaccination (3–5 months, 6–8 months, 9–12 months, 13–24 months). The syringes utilized for injections were indistinguishable in appearance and had stickers affixed to conceal the contents of the solution. To ensure blinding, all individuals involved, including participants, investigators, clinical research associates, data analysts, and laboratory staff, were unaware of the group allocation.

### Procedures

SCTV01C and SCTV01E are recombinant protein vaccines developed and manufactured by Sinocelltech Ltd. These vaccines are produced using Chinese hamster ovary (CHO) cells in accordance with good manufacturing practice guidelines. The primary components of SCTV01C consist of trimeric S-ECD proteins derived from the Alpha (B.1.1.7) and Beta (B.1.351) variants of the SARS-CoV-2 virus. SCTV01E is a tetravalent vaccine that includes S-ECD proteins from the Alpha, Beta, Delta, andicron BA.1 variants. Both vaccine candidates arejuvanted with SCT-VA02B, which is an oil-in-water emulsion containing squalene. The composition, formula, process and main quality control (particle size) of SCT-VA02B are consistent with the commercial marketable MF59 adjuvant. SCTV01C and SCTV01E were provided in single-use vials as sterile, emulsified, white solutions, with a volume of 0.5 mL per vial. These vaccines were stored and transported at temperatures between 2 and 8 °C, while being protected from light. The validity period for these vaccines was 24 months.^19^^,^^22^ BBIBP-CorV served as the control vaccine, and its dosage form, packaging, and route of administration were consistent with those of the study vaccines.

A total of 1351 participants who had previously received BBIBP-CorV 3–24 months earlier were enrolled to receive one dose of BBIBP-CorV, 20 μg SCTV01C or 30 μg SCTV01E in a ratio of 1:1:1. The doses of 20 μg SCTV01C and 30 μg SCTV01E were chosen based on preclinical studies in animals and subsequent clinical trials.^17^, ^18^, ^19^, ^20^, ^21^, ^22^ The antigens for the vaccines, including Alpha (B.1.1.7), Beta (B.1.351), Delta (B.1.617.2), and Omicron BA.1 variants have a high degree of similarity in their amino acid sequences (homology >96%) and secondary protein structures, leading to similar immunogenicity and reactogenicity responses. During non-clinical dose evaluation, in rats, 20 μg–40 μg of SCTV01C and 30 μg of SCTV01E induced comparable humoral immune responses. Correspondingly, in mice, 1.5 μg of SCTV01E generated a higher total S-specific IgG titer, T cell immune responses, and broadened neutralizing antibody against variants compared to 1.0 μg of SCTV01C.^22^ The results of three clinical trials investigating SCTV01C as a two-dose primary series or one booster vaccination indicated that increasing the dose of the SCTV01C vaccine from 20 μg to 40 μg induced a similar immunogenic response and did not raise any safety concerns.^19^, ^20^, ^21^ Based on these findings and taking into account the inclusion of two extra variant antigens in SCTV01E, a 30 μg spike protein dosage was chosen for SCTV01E.

Among them, the first 300 participants who had no history of SARS-CoV-2 infection were used for immunogenicity assessment, and stratified by age (18–54 years, ≥55 years), number of previous BBIBP-CorV injection, and the interval between previous vaccination and study vaccination (3–5 months, 6–8 month, 9–12 months, 13–24 months). The selected stratification factors were informed by our prior trials with protein vaccines and other clinical studies exploring COVID-19 booster vaccinations.^23^, ^24^, ^25^, ^26^ The first 150 participants of the immunogenicity subgroup were also tested for T-helper-1 (Th1) and T-helper-2 (Th2) responses. Post injection, solicited adverse event (AE) within 7 days; unsolicited AE within 28 days; serious AE (SAE) and AE of special interest (AESI) within 180 days were monitored and recorded. AEs and abnormal changes in laboratory tests were graded according to the Toxicity Grading Scale for Healthy Adult and Adolescent Volunteers in Preventive Vaccine Clinical Trial–FDA Standard.^27^ Serum samples were obtained on days 0, 28, and 180 to assess the geometric mean titer (GMT) of neutralizing antibody activity against live SARS-CoV-2 variants, including Delta, Omicron BA.1, and BA.5 with plaque reduction neutralization test (PRNT).^20^^,^^28^^,^^29^ The peripheral blood mononuclear cells were collected to assess specific Th1 (interferon-gamma (IFN-γ) release) and Th2 (interleukin-4 (IL-4) release) responses before and at day 28 after vaccination using T-SPOT^Ⓡ^.COVID test and enzyme-linked immunospot (ELISpot) IL-4 COVID TEST assay.^30^ The ELISpot assay and live virus neutralization assay were performed according to the manufacturer’s guidelines (Biogenix, Abu Dhabi, United Arab Emirates) at G42 LABORATORY LLC in Abu Dhabi, United Arab Emirates.

The Plaque Reduction Neutralization Assay (PRNT) was utilized to measure vaccine-induced neutralizing activity, which was validated by Biogenix Labs and G42 Healthcare and met predefined acceptance criteria. The validation parameters followed the EMA/FDA guidance on biomarker assays, including criteria such as low/maximum limit of detection, precision and accuracy, limits of quantification, dilution linearity, stability, and interference. Optimizations were undertaken for key experimental parameters, such as cell seeding duration, working viral dilution, and infection time. Intra- and inter-assay precision were evaluated, and 90% of the observed results within a 2-fold difference of the tested samples were considered acceptable. Positive controls were incorporated in each run. Serum samples were subjected to a 30-min incubation at 56 °C in a water bath to inactivate complement and other nonspecific inhibiting antibodies. The sera were diluted five times initially and then serially diluted from 1:10 to 1:640. These dilutions were mixed with SARS-CoV-2 variants (Delta, Omicron BA.1, and BA.5); and transferred in duplicate to sub-confluent Vero E6 cell monolayer plates. Following incubation for 3–5 days at 37 °C and 5% CO_2_ in 6-well plates, antibody titers were defined as the highest serum dilution resulting in >50% (PRNT50) reduction in the number of plaques compared to negative control. The negative control was defined as plaque count ≥50, while the positive control was designated as plaque count ≤50% of the negative control. A cut-off for positivity was set at 1:20.

ELISpot assays (Biogenix Labs, G42 Healthcare) were used to quantify Th1 and Th2 responses by counting the number of Spot Forming Units (SFU) that secrete antigen-specific IFN-γ or IL-4. Cryopreserved peripheral blood mononuclear cells (PBMC) were rapidly thawed and allowed to rest overnight. For PBMC stimulation, peptide pools derived from the Spike protein of the ancestral strain of SARS-CoV-2. These peptide pools were provided as two lyophilized mixtures (subpools) and each subpool contains 158 peptides, for a total of 316 peptides. The subpools consist of 15-mer peptides with 11-amino-acid overlaps that cover amino acids 1–1273 on the spike protein (Catalog 100-0676, STEMCELL Technologies). Cells were dispensed and stimulated with a pool of peptides containing Spike antigens, bovine serum albumin and antimicrobial agents, and incubated at 37 °C for 24–48 h. Cells stimulated with phytohemagglutinin (PHA) were used as the positive control. Following the manuals, IFN-γ or IL-4 release was detected, and the spots were counted directly from the well using a magnifying glass or stereomicroscope, or from a digital image captured from a microscope or plate imager. The number of specific T cells secreting IFN-γ and IL-4 was quantified as spot per million PBMC. Only subjects with available baseline and post-baseline data were included in the analysis.

### Outcomes

The primary objective of this trial was to evaluate the immunogenicity of two protein-based vaccines as compared to inactivated vaccine. Additionally, the secondary objectives included assessing the levels of neutralizing antibody and T-cell responses between SCTV01C and SCTV01E. The primary endpoints of the trial were the GMTs of neutralizing antibody against Delta (B.1.617.2) and Omicron BA.1 at day 28 post-booster injection. Safety endpoints included evaluating the occurrence and severity of adverse reactions (ARs) within 7 days, solicited AEs within 7 days, unsolicited AEs within 28 days, as well as SAEs and AESIs within 180 days following vaccination. Other endpoints for immunogenicity included GMTs of neutralizing antibody to Delta and Omicron BA.1 on day 180, the seroresponse rate at day 28, and day 28 GMTs to newly identified Variants of Concerns (VOCs) that may have emerged during the study. Since Omicron BA.5 was classified as a VOC during the trial, the Day 28 neutralizing antibody response against it was evaluated as a secondary endpoint. IFN-γ positive (characterizing Th1) and IL-4 positive (characterizing Th2) T cell responses were explored at day 28 post-injection. An independent data and safety monitoring board (DSMB) reviewed the data.

### Statistics

The statistical analyses were done with SAS software (version 9.4). The statistical analysis was carried out with descriptive and pre-specified statistical test methods. For the safety analysis, the proportions of participants with at least one solicited AEs within 7 days and unsolicited AEs within 28 days were reported for each group. In the immunogenicity analysis, data that fell below the lower limit of detection were imputed as half of the threshold value. GMT and geometric mean fold increase over baseline with corresponding 95% CI were provided at each time point. The 95% CIs were computed based on the t-distribution of the log-transformed values then converted back to the original scale for presentation. To analyze the comparisons of the GMTs and geometric mean ratios (GMR) across groups, analysis of Covariance (ANCOVA) was performed on the log-transformed data. The ANCOVA included covariates such as the intervention group, age group, interval from last COVID-19 vaccination, and baseline values (in log-transformed scale). To comply with regulatory requirements for Emergency Use Authorization (EUA), a total of 1351 participants were enrolled in the trial, as recommended by the local regulatory authorities to ensure safe evaluation of the vaccine. The sample size for the immunogenicity assessment was determined based on a superiority design intended to demonstrate that SCTV01C and SCTV01E were superior to BBIBP-CorV in terms of the GMTs of neutralizing antibody against Omicron BA.1 and Delta variants. The statistical assumptions included the following: standard deviation of GMTs under log10 transformation was 0.4, GMR between SCTV01C, SCTV01E and BBBIP-CorV was 1.6 and the dropout rate during the study was about 10% with the 1-sided type I error of 0.025 and a power of 80%. To control the type I error at a one-sided significance level of 0.025, a fixed sequential hierarchical approach was used as follows, where the GMTE1_**variant**_ GMTC1_**variant**_, and GMTS1_**variant**_ are the geometric mean of SCTV01E, SCTV01C and BBIBP-CorV for a specific variant (Omicron BA.1, Delta, and Omicron BA.5) respectively. (H11: GMR13 = GMTE1_Omicron1_/GMTS1_Omicron1_ ≤1; H12: GMR14 = GMTC1_Omicron1_/GMTS1_Omicron1_ ≤1; H13: GMR12 = GMTE1_Delta_/GMTS1_Delta_ ≤1; H14: GMR12 = GMTE1_Delta_/GMTS1_Delta_ ≤1; H15: GMR15 = GMTE1_Omicron5_/GMTS1_Omicron5_ ≤1; H16: GMR16 = GMTC1_Omicron5_/GMTS1_Omicron5_ ≤1). The hypotheses were tested in the following order: H11, H12, H13, H14, H15, and H16. Each subsequent test was conducted only after the previous one had achieved statistical significance at the one-sided significance level of 0.025. Seroresponse for participants with pre-dose GMTs lower than the low limit of quantitation (LLOQ) is defined as equal to or above, and seroresponse for participants with pre-dose ≥ LLOQ is defined as ≥4-fold over pre-dose titer (Supplementary Materials 1).

### Role of the funding source

The funders of the study were not involved in protocol design, data collection, statistical analysis, data interpretation, or writing of the report. All the authors had full access to all the data in the study and had final responsibility for the decision to submit for publication.

## Results

### Study participants

**Fig. 1:**
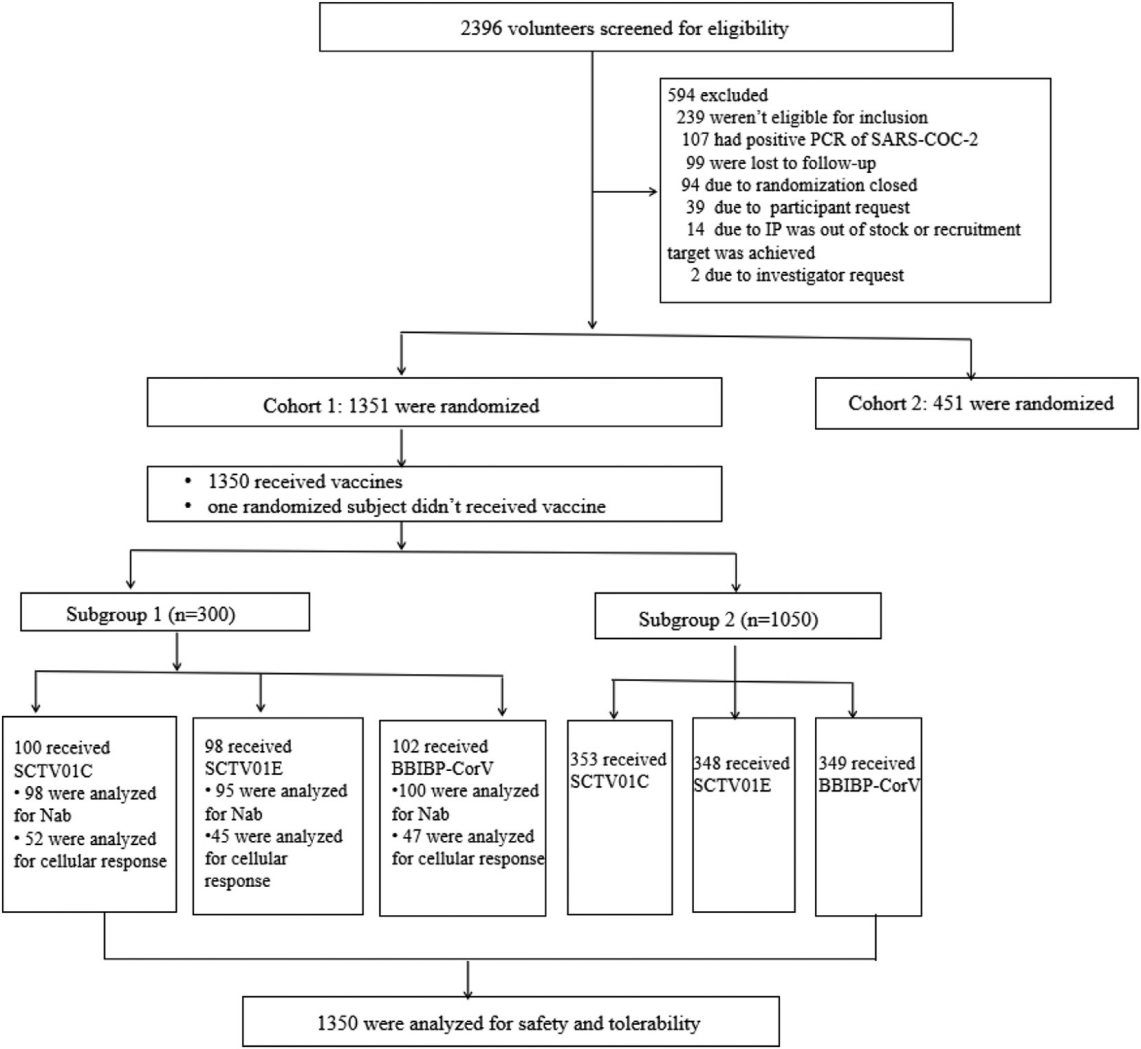
**Flow diagram of the participants.** Nab, neutralizing antibody.

Between May 30, 2022 and October 28, 2022, 1351 participants were enrolled, with 452, 453 and 446 participants in BBIBP-CorV, SCTV01C and SCTV01E booster groups, respectively (Fig. 1 and Table 1). The demographic and other baseline characteristics were generally comparable for participants in each group. Participants in each group had similar time intervals between investigational vaccination and prior vaccination. Of all participants, 99.5% were male and the median age was 29 years old (range from 18 to 58). All 1350 participants had received 2 doses (80.7%) or 3 doses (19.3%) of inactivated vaccine before entering the study. Among them, 79 (5.8%) of them were previously diagnosed with COVID-19. The interval between investigational vaccination and prior COVID-19 vaccination were 3–5 months (9.4%), 6–8 months (16.6%), 9–12 months (32.7%) and 13–24 months (41.3%), respectively. The immunogenicity subgroup included 300 participants, with 102, 100 and 98 injected with BBIBP-CorV, SCTV01C and SCTV01E, respectively (Supplementary Table S1 in Supplementary Materials 2). The median age for the immunogenicity subgroup was 29.0 years old (range from 20 to 58). Among them, 236 (78.7%) had previously received 2 doses of inactivated vaccine and 64 (21.3%) had received 3 doses previously, and none of them were previously diagnosed with COVID-19. None of the three groups reported any cases of symptomatic COVID-19 infection during the follow-up period when the data was locked for analysis.

**Table 1:** Baseline demographics of participants.

	SCTV01C (N = 453)	SCTV01E (N = 446)	Overall (N = 1351)
Age (years)			
n	453	446	1351
Mean (SD)	30.3 (7.31)	30.1 (7.39)	30.2 (7.52)
Median	29	29	29
Min, max	19, 58	18, 57	18, 58
18–54	451 (99.6)	445 (99.8)	1347 (99.7)
≥55	2 (0.4)	1 (0.2)	4 (0.3)
Gender, n (%)			
Female	2 (0.4)	4 (0.9)	7 (0.5)
Male	451 (99.6)	442 (99.1)	1344 (99.5)
Race, n (%)			
Asian	450 (99.3)	438 (98.2)	1336 (98.9)
Black or African American	1 (0.2)	6 (1.3)	10 (0.7)
Other	2 (0.4)	2 (0.4)	5 (0.4)
BMI (kg/m^2^)			
n	453	446	1351
Mean (SD)	24.80 (4.209)	24.77 (4.103)	24.69 (4.111)
Median	24.8	24.4	24.4
Min, max	16.5, 41.7	16.9, 40.9	15.5, 41.7
Number of prior COVID-19 vaccine doses			
1	0	0	0
2	364 (80.4)	360 (80.7)	1090 (80.7)
3	89 (19.6)	86 (19.3)	261 (19.3)
Infection history of COVID-19			
Yes	27 (6.0)	25 (5.6)	79 (5.8)
No	426 (94.0)	421 (94.4)	1272 (94.2)
Interval from last COVID-19 vaccination (month)			
3–5	45 (9.9)	39 (8.7)	127 (9.4)
6–8	77 (17.0)	75 (16.8)	224 (16.6)
9–12	143 (31.6)	140 (31.4)	442 (32.7)
13–24	188 (41.5)	192 (43.0)	558 (41.3)

Abbreviations: SD, standard deviation; BMI, body mass index.

### Safety outcomes

All participants completed the day 28 visit. The occurrences and severities of adverse reactions were similar among BBIBP-CorV, SCTV01C and SCTV01E booster groups. Most AEs were Grade 1 or 2. There were 15 ≥Grade 3 AEs: 6 (1.3%) in BBIBP-CorV group; 4 (0.9%) in SCTV01C group and 5 (1.1%) in SCTV01E group. No SAE and one grade 1 AESI (Bell's palsy) were observed (SCTV01C group). Overall, 62 (13.7%), 67 (14.8%) and 70 (15.7%) participants experienced at least one treatment related AE (TRAE) in BBIBP-CorV, SCTV01C and SCTV01E groups, respectively. The frequency of solicited AEs was 38 (8.4%) in BBIBP-CorV group, 50 (11%) in SCTV01C group and 56 (12.6%) in SCTV01E group. The most frequent (≥3%) solicited AEs were injection pain and pyrexia. The occurrences of unsolicited AEs within 28 days after the injection were also similar for BBIBP-CorV group (8.0%), SCTV01C group (6.4%) and SCTV01E group (7.6%) (Table 2 and Supplementary Figure S1 in Supplementary Materials 2).

**Table 2:** Summary of AEs.

	BBIBP-CorV	SCTV01C	SCTV01E
	n = 451^a^	n = 453	n = 446
	n (%)	n (%)	n (%)
TEAE	67 (14.9)	72 (15.9)	77 (17.3)
TRAEs	62 (13.7)	67 (14.8)	70 (15.7)
≥Grade 3 AE	6 (1.3)	4 (0.9)	5 (1.1)
≥Grade 3 TRAEs	6 (1.3)	4 (0.9)	5 (1.1)
Solicited AEs	38 (8.4)	50 (11.0)	56 (12.6)
IP related solicited AEs	38 (8.4)	50 (11.0)	55 (12.3)
Solicited systemic AEs	26 (5.8)	25 (5.5)	27 (6.1)
Grade 1	17 (3.8)	19 (4.2)	19 (4.3)
Grade 2	3 (0.7)	3 (0.7)	3 (0.7)
≥Grade 3	6 (1.3)	3 (0.7)	5 (1.1)
Pyrexia	16 (3.5)	14 (3.1)	11 (2.5)
Grade 1	8 (1.8)	9 (2.0)	4 (0.9)
Grade 2	2 (0.4)	2 (0.4)	2 (0.4)
≥Grade 3	6 (1.3)	3 (0.7)	5 (1.1)
Headache	9 (2.0)	8 (1.8)	13 (2.9)
Grade 1	7 (1.6)	7 (1.5)	12 (2.7)
Grade 2	2 (0.4)	1 (0.2)	1 (0.2)
≥Grade 3	0	0	0
Myalgia	2 (0.4)	8 (1.8)	6 (1.3)
Grade 1	1 (0.2)	8 (1.8)	5 (1.1)
Grade 2	1 (0.2)	0	1 (0.2)
≥Grade 3	0	0	0
Fatigue	2 (0.4)	1 (0.2)	3 (0.7)
Grade 1	2 (0.4)	1 (0.2)	3 (0.7)
Grade 2	0	0	0
≥Grade 3	0	0	0
Arthralgia	1 (0.2)	1 (0.2)	0
Grade 1	1 (0.2)	1 (0.2)	0
Grade 2	0	0	0
≥Grade 3	0	0	0
Solicited local AEs	15 (3.3)	27 (6.0)	31 (7.0)
Injection site pain	12 (2.7)	25 (5.5)	30 (6.7)
Injection site erythema	2 (0.4)	2 (0.4)	2 (0.4)
Injection site swelling	1 (0.2)	2 (0.4)	2 (0.4)
Unsolicited AEs	36 (8.0)	29 (6.4)	34 (7.6)
IP related unsolicited AEs	31 (6.9)	22 (4.9)	27 (6.1)
AESI	0	1 (0.2)	0

Abbreviations: AE, adverse event; TEAE, treatment emerged adverse event; TRAE, treatment related adverse event; IP, investigational product; AESI, adverse event of special interest.

a The group size for BBIBP-CorV is n = 452, but one participant in BBIBP-CorV did not receive the vaccine.

### GMT of live virus neutralizing antibody

**Fig. 2:**
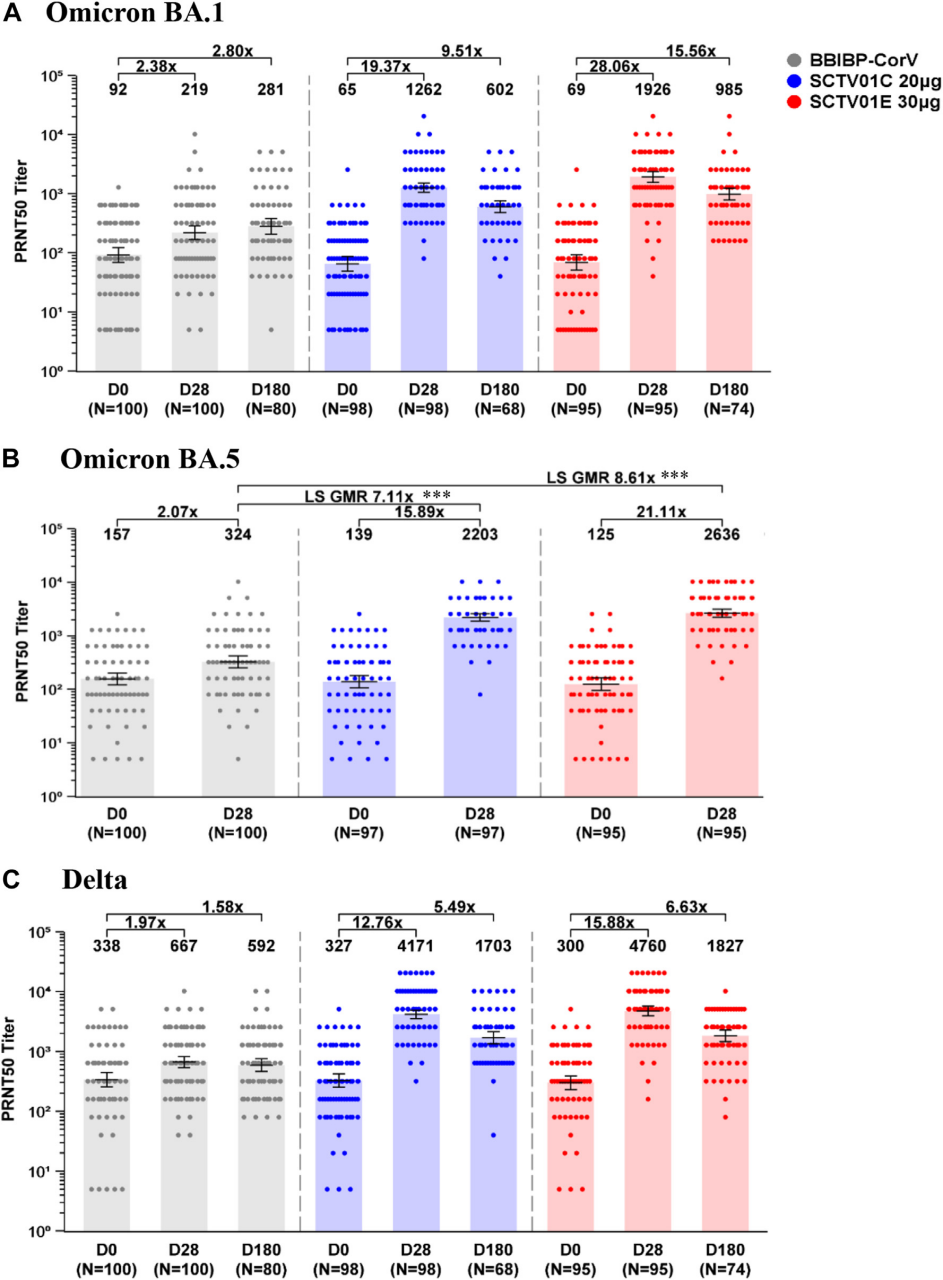
**GMTs of live virus neutralizing antibody against Omicron BA.1 (A), BA.5 (B) and Delta (C).** GMTs of neutralizing antibody were measured using 50% plaque reduction neutralization test (PRNT50). Bars show the GMTs with 95% CIs at day 0, day 28 and day 180. Centre of the error bars represents the GMT. Dots represent the values for individual participants. Note: BNT162B2 group (grey), SCTV01C group (blue) and SCTV01E group (red). Abbreviations: GMT, geometric mean titer; GMR, geometric mean ratio; PRNT50, 50% plaque reduction neutralization test. ∗∗∗p < 0.0001.

Immunogenicity assessment data were acquired from 100 participants in BBIBP-CorV group, 98 in SCTV01C group and 95 participants in SCTV01E group. At day 28 after vaccination, the GMT (95% CI) of live virus neutralizing antibody against Omicron BA.1 were 219 (167, 286) with 2.38-fold over baseline, 1262 (1056, 1509) with 19.37-fold and 1926 (1557, 2382) with 28.06-fold over baseline in BBIBP-CorV, SCTV01C and SCTV01E groups, respectively. Likewise, the GMR of SCTV01C/BBIBP-CorV, SCTV01E/BBIBP-CorV and SCTV01E/SCTV01C were 6.49 (p < 0.0001), 9.56 (p < 0.0001) and 1.50 (p < 0.01), which met the pre-specified criterion for superiority. The GMTs (95% CI) against Omicron BA.5 were 324 (251, 419) with 2.07-fold over baseline, 2203 (1872, 2593) with 15.89-fold and 2636 (2227, 3120) with 21.11-fold increase from baseline in BBIBP-CorV, SCTV01C and SCTV01E booster groups, respectively. The GMR of SCTV01C/BBIBP-CorV, SCTV01E/BBIBP-CorV and SCTV01E/SCTV01C were 7.11 (p < 0.0001), 8.61 (p < 0.0001) and 1.20 (p = 0.12). The GMTs (95% CI) against Delta variant were 667 (541, 823) with 1.97-fold over baseline, 4171 (3545, 4906) with 12.76-fold over baseline, and 4760 (3939, 5752) with 15.88-fold over baseline in BBIBP-CorV, SCTV01C and SCTV01E booster groups, respectively. The GMR of SCTV01C/BBIBP-CorV, SCTV01E/BBIBP-CorV and SCTV01E/SCTV01C were 6.26 (p < 0.0001), 7.26 (p < 0.0001) and 1.15 (p = 0.28) (Fig. 2).

The live virus neutralizing antibody GMT (95% CI) against Omicron BA.1 at day 180 post-vaccination was 281 (207, 381), representing 2.80-fold over baseline in the BBIBP-CorV group, 602 (479, 757) with 9.51-fold over baseline in the SCTV01C group, and 985 (781, 1241) with 15.56-fold over baseline in the SCTV01E group. The GMRs of SCTV01C/BBIBP-CorV, SCTV01E/BBIBP-CorV, and SCTV01E/SCTV01C were 2.42 (p < 0.0001), 3.78 (p < 0.0001), and 1.64 (p = 0.0032), respectively, meeting the pre-specified criterion for superiority. For the Delta variant, the GMTs (95% CI) were 592 (463, 758) with 1.58-fold over baseline, 1703 (1347, 2152) with 5.49-fold over baseline, and 1827 (1469, 2272) with 6.63-fold over baseline in the BBIBP-CorV, SCTV01C, and SCTV01E booster groups, respectively. The GMRs of SCTV01C/BBIBP-CorV, SCTV01E/BBIBP-CorV, and SCTV01E/SCTV01C were 2.95 (p < 0.0001), 3.24 (p < 0.0001), and 1.10 (p = 0.559) (Fig. 2).

From day 28 to day 180 post-vaccination, some participants exhibited an increase neutralizing in antibody titers against Omicron BA.1. Specifically, in the BBBIP-CorV group, 36.3% of participants showed an increase, while in the SCTV01C and SCTV01E groups, 11.8% and 13.5%, respectively, exhibited such an increase (Supplementary Figure S2). These findings suggest a possibility of asymptomatic breakthrough infections, particularly in the BBBIP-CorV group.

The participants were stratified based on the time intervals between the previous and study vaccination, and the number of prior doses of COVID-19 vaccine. For all time interval subgroups (3–5 months, 6–8 months, 9–12 months and 13–24 months), 2 prior doses subgroup and 3 prior doses subgroup, SCTV01E and SCTV01C induced significantly higher GMTs of neutralizing antibody against Omicron BA.1, BA.5 and Delta than that of BBIBP-CorV on day 28 after vaccination (Supplementary Figures S3–S5 and Supplementary Tables S2–S4 in Supplementary Materials 2).

At day 28 after vaccination, the seroresponse rates of live virus neutralizing antibody against variants: Omicron BA.1 were 28.0%, 87.8% and 92.6%; Omicron BA.5 were 23.0%, 88.7% and 92.6%; Delta were 18.0%, 86.7% and 92.6% in BBIBP-CorV, SCTV01C and SCTV01E groups, respectively (Supplementary Table S5 in Supplementary Materials 2). Both SCTV01E and SCTV01C elicited significantly higher seroresponse rates than those with BBIBP-CorV group for Omicron BA.1, BA.5, and Delta (p < 0.0001).

### Post hoc analysis of neutralizing antibody

**Fig. 3:**
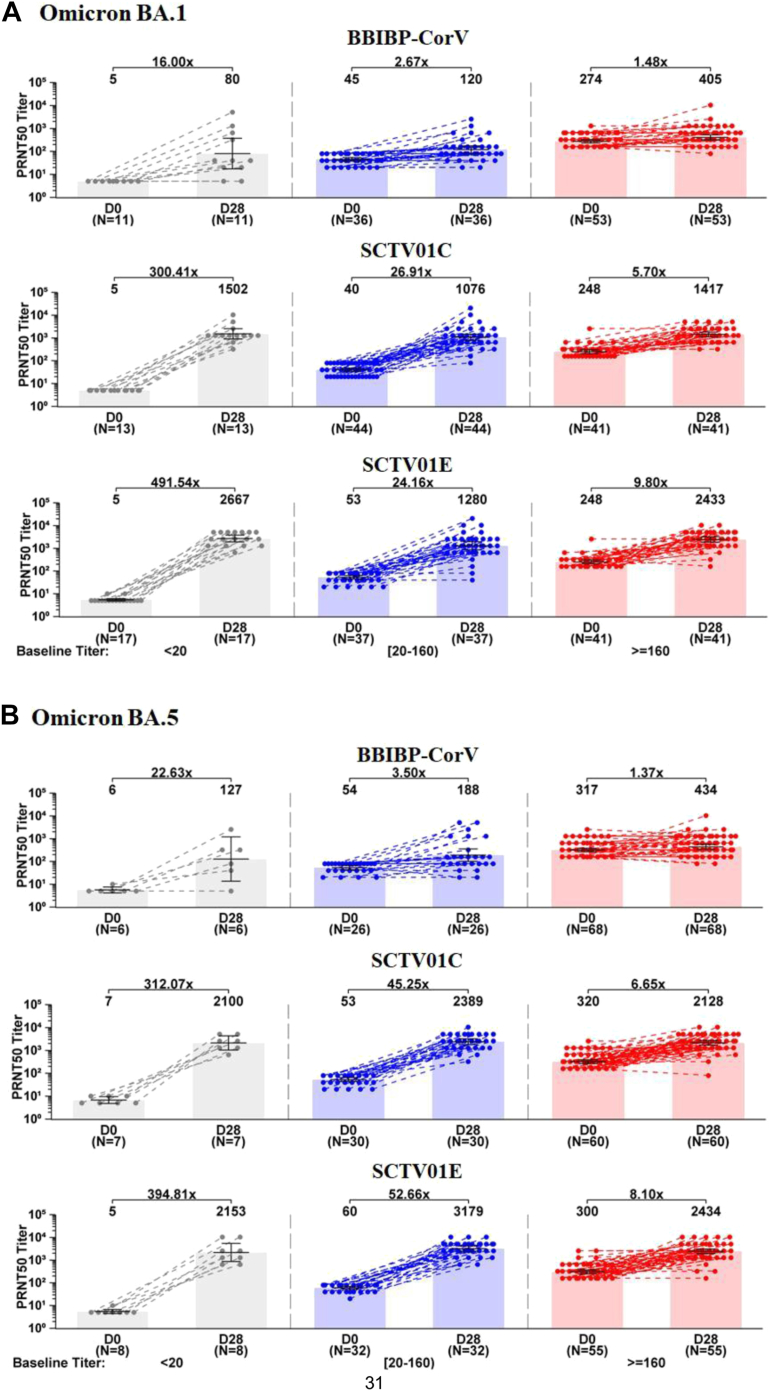
**GMTs of neutralizing antibody against live Omicron BA.1 (A) and BA.5 (B) in groups with low, medium and high baseline titers.** Participants were assigned to three groups based on the GMT levels at baseline. Pre-dose GMTs lower than the lower limit of quantitation (<LLOQ: 20), in the range of 20–160 and equal to or over 160 were considered as low (grey), medium (blue) and high (red) baseline titers, respectively. Bars show the GMTs with 95% CIs at day 0 and day 28. Centre of the error bars represents the GMT. Dots represent the values for individual participants. Note: Only those with available baseline and post-baseline data were included. Abbreviations: GMT, geometric mean titer; PRNT50, 50% plaque reduction neutralization test.

The participants were assigned to three groups based on the pre-dose GMT levels: low baseline titer group (<LLOQ: 20), medium baseline titer group (20–160), and high baseline titer group (≥160) (Fig. 3). Day 28 GMTs of the neutralizing antibody against BA.1 and BA.5 with SCTV01E were 2667 and 2153, 1280 and 3179, 2433 and 2434 with the corresponding fold of increase of 491.54 and 394.81, 24.16 and 52.66, 9.80 and 8.10, for the low, medium and high baseline titer groups, respectively. The neutralizing antibody responses with SCTV01E and SCTV01C were consistently superior to those with BBIBP-CorV groups, irrespective of baseline GMTs levels of the participants. Both SCTV01E and SCTV01C groups induced similar high GMTs against Omicron BA.1 and BA.5 in low baseline GMT compared with those with high baseline titer. However, Day 28 GMTs to Omicron BA.1 and BA.5 with BBIBP-CorV were 5.06 and 3.42-fold lower in the low baseline groups than those in high baseline groups.

### T-cell responses

**Fig. 4:**
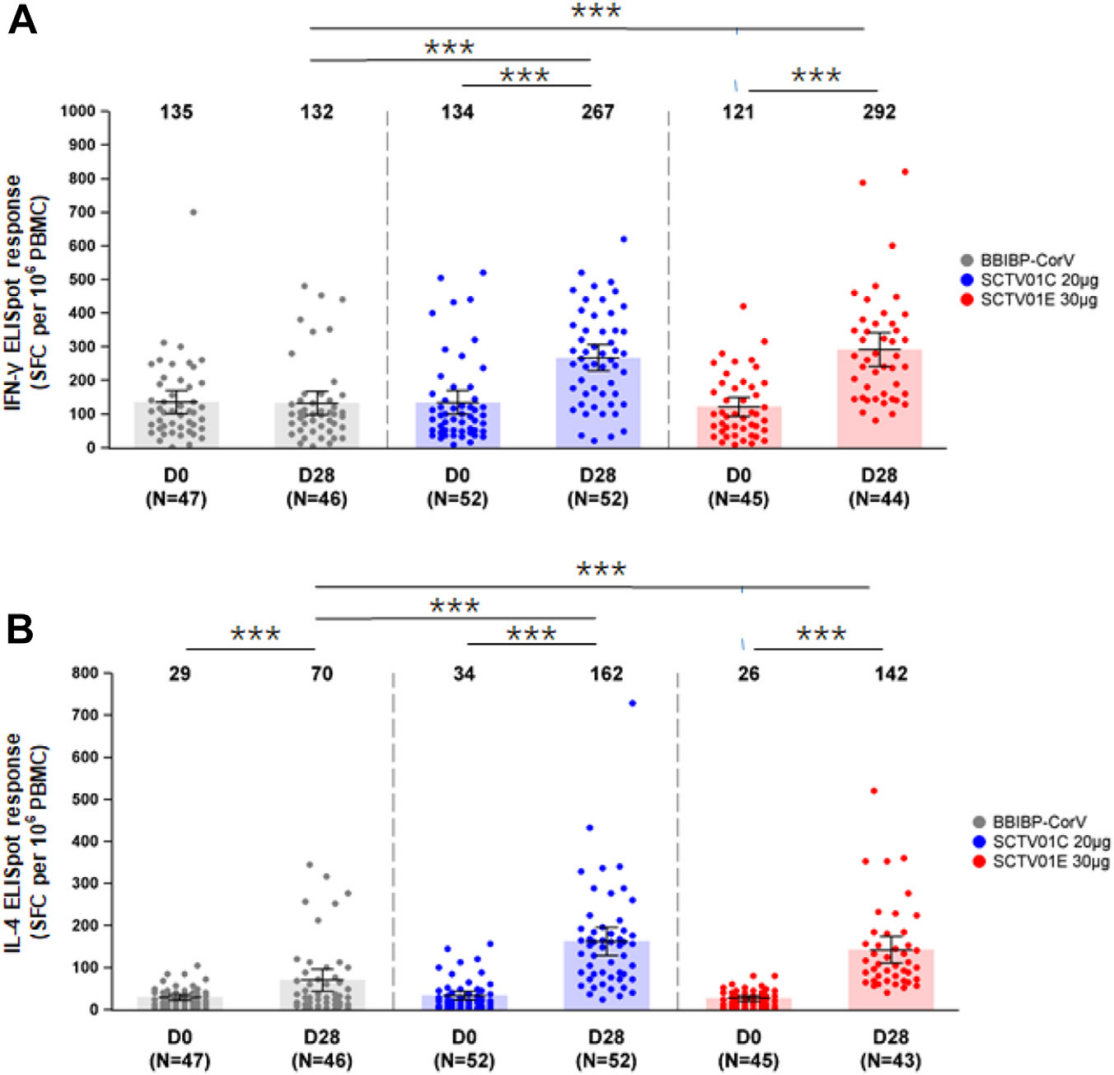
**Th1 (A. IFN-γ release) and Th2 (B. IL-4 release) responses.** The peripheral blood mononuclear cells (PBMC) were collected from the participants before, and at day 28 after booster vaccination. The number of specific T cells with secretion of IFN-γ (Th1) and IL-4 (Th2) were measured with spot per 10⁶ PBMC using enzyme-linked immunospot (ELISpot) assay. Error bars show the number of SFC per 10^6^ PBMC with 95% CIs at day 0 and day 28. Centre of the error bars represents the mean number of SFC per 10^6^ PBMC. Dots represent the values for individual participants. Abbreviations: ELISpot, enzyme-linked immunospot; SFC, spots forming cells; PBMC, peripheral blood mononuclear cells; ∗∗∗p < 0.0001.

The peripheral blood mononuclear cells were collected to assess specific Th1 (IFN-γ release) and Th2 (IL-4 release) responses at day 28 after booster vaccination, For SCTV01C and SCTV01E groups, the means (SD) of IFN-γ expressing Th1 cell were 267.1/10^6^ peripheral blood mononuclear cell (PBMC) (140.9) with a 2-fold increase (p < 0.0001) and 291.5/10^6^ PBMC (164.92) with a 2.4-fold increase (p < 0.0001) from baseline, respectively. IFN-γ expressing Th1 cells in BBIBP-CorV group did not increase as compared with the baseline level. The means (SD) of IL-4 expressing T cells were 70.0/10^6^ PBMC (88.49) with 2.4-fold increase (p < 0.0001), 162.0/10^6^ PBMC (120.94) with a 4.8-fold increase (p < 0.0001) and 142/10^6^ PBMC (101.36) with a 5.5-fold increase (p < 0.0001) from baseline in BBIBP-CorV, SCTV01C and SCTV01E booster groups respectively. Both SCTV01C and SCTV01E vaccination elicited significantly higher number of IFN-γ expressing Th1 cells and IL-4 expressing Th2 cells than those with BBIBP-CorV (p < 0.0001) (Fig. 4).

## Discussion

This phase 3 trial evaluated the safety, reactogenicity, and immunogenicity of a booster dose of SCTV01E and SCTV01C, in comparison to the inactivated vaccine. The target population was adults who had previously received 1, 2 or 3 doses of inactivated COVID-19 vaccine. Inactivated vaccines such as CoronaVac (Sinovac) and BBIBP-CorV (Sinopharm) had been widely used as COVID-19 primary series of vaccination in most countries due to their less stringent transport and storage conditions, as well as relatively lower AE rates, compared to adenoviral vector vaccines like ChAdOx1-S or mRNA-based vaccines like Pfizer BNT162b2.^31^^,^^32^ SCTV01E, a variant-adapted vaccine, designed to provide broad-spectrum protection and developed as a thermostable and easy-to-administer vaccine, could be a promising booster candidate for people with primary series of inactivated vaccine.

SCTV01E was developed as an enhanced version of the bivalent (Alpha + Beta) vaccine SCTV01C. It incorporates two additional components, specifically targeting the Delta and Omicron BA.1 variants. Previously, SCTV01C was assessed as primacy series and boosting vaccination in three clinical trials.^19^, ^20^, ^21^ In these studies, SCTV01C demonstrated a low rate of AE that was comparable to those of inactivated vaccine (BBBIP-CorV).^28^^,^^33^ Consistent with the findings of these studies, participants with SCTV01C and SCTV01E booster reported a similar occurrences and severities of adverse reactions (ARs) as participants who received BBBIP-CorV. The frequencies of solicited ARs were 38 (8.4%) in BBIBP-CorV recipients, 50 (11%) in SCTV01C recipients and 56 (12.6%) in SCTV01E recipients. Most adverse reactions were Grade 1 or 2, and the most frequent (≥3%) solicited AEs were injection pain and pyrexia. There were no reported cases of serious adverse events that were assessed as related to the study vaccine and no safety concerns were identified. Overall, the safety and reactogenicity profiles of SCTV01E and SCTV01C booster were similar to the reported AEs of primary^33^^,^^34^ and/or homologous booster vaccination with inactivated vaccines^25^^,^^35^ (CoronaVac showed 6–18% solicited ARs and 1–16% of injection-site pain; BBIBP-CorV showed 12.72% solicited ARs, 3.98% of injection-site pain and 4.2% of headaches.

Viral neutralizing antibody levels are highly predictive of immune protection from symptomatic SARS-CoV-2 and have been used to infer COVID-19 vaccine effectiveness.^36^, ^37^, ^38^, ^39^ In this study, the primary analyses evaluated the live virus GMTs and seroresponse rates (SRRs) against Omicron BA.1, BA.5 and Delta variant. The day 28 GMTs of neutralizing antibody against Omicron BA.1 BA.5 and Delta variants with SCTV01E booster were 28.06-, 21.11- and 15.88-fold over baseline, respectively. The pre-specified statistical success criteria were met for superiority of GMT ratios of SCTV01E/BBIBP-CorV and SCTV01C/BBIBP-CorV against Omicron BA.1, BA.5 and Delta variants. Additionally, SCTV01E booster showed GMT superiority to SCTV01C against Omicron BA.1. The statistical success criteria were also met for superiority of difference in SRRs (SCTV01E minus BBIBP-CorV and SCTV01C minus BBIBP-CorV) against Omicron BA.1, BA.5 and Delta, based on protocol definition for seroresponse. These results, together with previous findings with SCTV01C^19^, ^20^, ^21^ and recent publications on Moderna^14^^,^^15^ and Pfizer^16^ Omicron-containing bivalent vaccines, suggest increased immunogenicity responses and cross-reactivity with multivalent vaccines.

Our study, mainly conducted between May 2022 and October 2022 in the UAE, found a high neutralizing antibody (nAb) response against Omicron BA.5 on day 28 after vaccination. During this period, Omicron BA.5 was the dominant strain with peak circulation.^40^ Our findings showed that individuals had higher baseline GMTs of nAb against BA.5 compared to Omicron BA.1 (125 vs. 69). It is possible that some asymptomatically infected individuals with Omicron BA.5 participated in the study, leading to a robust vaccine-induced immune response against this variant. Similar observations have been made in other investigations of COVID-19 vaccines; indicating that individuals who receive the vaccine shortly after contracting the Omicron variant may experience an increased immune response to the vaccine.^14^ Even though the GMTs of neutralizing antibody against Omicron BA.5 were high on day 28 post-vaccination, their fold-increase over baseline was significantly lower than that for Omicron BA.1 (21.11-fold vs. 28.09-fold).

Post hoc analyses evaluated the impact of the pre-existing SARS-COV-2 immunity on the neutralizing antibody responses to Omicron BA.1 and BA.5. The participants were assigned to three groups based on the pre-dose GMTs levels. The neutralizing antibody responses with SCTV01E and SCTV01C were consistently superior to those with BBIBP-CorV, irrespective of baseline GMTs levels of the participants. Notably, both SCTV01E and SCTV01C induced high GMTs in the participants with low baseline that were comparable to those with high baseline titers. Compared to BBIBP-CorV, both SCTV01E and SCTV01C boosters elicited higher GMTs of nAb in participants with low baseline titers. This is consistent with previous research on the immunogenicity of SCTV01C^20^^,^^21^ and the study of SCTV01E in individuals who previously received mRNA vaccines (not yet published). These trials consistently demonstrate that both SCTV01C and SCTV01E triggered robust immune responses in participants with low initial levels of nAb. One possible explanation for this observation is that individuals with low baseline titers of nAb may have had weaker or less effective natural immune responses to the virus before vaccination. As a result, the vaccine may stimulate a stronger and more effective immune response in these individuals, leading to higher GMTs of nAb. The exact mechanisms underlying this observation are still being studied and more research is needed to fully understand the relationship between baseline nAb titers and vaccine-induced antibody responses.

To date, SCTV01C and/or SCTV01E have been evaluated in seven clinical trials, collectively demonstrating their potential as important platforms amid the challenging epidemiological situation where multiple major variants are prevalent. The platform's flexibility allows for rapid replacement of new variant antigens to adapt to immune-evading strains. While this study showed statistically significant differences in post-booster antibody titers between study groups, further clinical evidence is needed to determine whether the higher antibody titers translate into superior clinical efficacy or longer-lasting protection. This safety and immunogenicity trial was conducted in two cohorts, with the current manuscript presenting data from cohort 1. For cohort 2, Pfizer mRNA BNT16262 was used as a comparator. A total of 450 participants who had already received 2 or 3 doses of an mRNA COVID-19 vaccine were enrolled. The results showed that SCTV01E demonstrated both clinically acceptable safety profiles, while also exhibiting superior immunogenicity compared to both the bivalent vaccine SCTV01C and BNT16262). The data for this cohort have been submitted to another medical journal and are currently under review. A phase 3 efficacy study with SCTV01E is currently ongoing in China (NCT05308576).

The study had several limitations. Firstly, this study was conducted in an environment with a high prevalence of the Omicron variant, which may have led to asymptomatic breakthrough infections during the trial, however, there was no standard way to differentiate asymptomatic infection and previous vaccination with an inactivated vaccine within the population. Secondly, the immunogenicity assessments were focused on the Delta variant (B.1.617.2) and Omicron variants, which were the most prominent circulating variants at the time of the study. However, the neutralizing antibody responses to antigen-matched variants such as Alpha (B.1.1.7) and Beta (B.1.351) were not evaluated. Additionally, the study's sample population was mostly composed of young male adults. This lack of diversity may affect the generalizability and applicability of the study results. Although previous clinical studies involving SCTV01C did not reveal any significant differences in AEs or immunogenicity between male and female participants, further investigations on SCTV01E with a more balanced demographic representation are necessary. Currently, a large-scale efficacy phase 3 trial on SCTV01E, involving 5274 male and 3949 female participants is ongoing.

In summary, 30-μg tetravalent protein vaccine SCTV01E, when administered as a heterologous booster dose, had a safety and reactogenicity profile that was similar to that of bivalent vaccine SCTV01C and inactivated vaccine BBBIP-CorV, and elicited consistently high neutralizing antibody responses against Omicron BA.1, BA.5 and Delta variant, showing superior immunogenicity compared to those with BBIBP-CorV booster. SCTV01E may also have GMT superiority over bivalent vaccine SCTV01C against Delta, BA.1 and BA.5 variants. These findings indicate that tetravalent vaccine could help maintain optimal broad protection against emerging variants of SARS-CoV-2.

## Contributors

Dr. Suad Hannawi was the study site principal investigators, responsible for the supervision of the study, the coordination of resources, data analysis, data verification and interpretation. Dr. Linda Safeldin, Dr. Alaa Abuquta and Dr. Ahmad Alamadi contributed to participant management and implementation of the study (vaccine management, vaccination, participant screening management, communication and coordination with the sponsor, CRO, and ethics). Dr. Sally A Mahmoud was responsible for laboratory testing and assay development. Dr. Miaomiao Zhang and Dr. Yuanxin Chen contributed to the medical management, data processing and manuscript drafting. Dr. Cuige Gao and Dr. Lixin Yan contributed to the protocol drafting and medical monitoring. Dr. Wenlin Gai contributed to study conception and project management. Dr. Liangzhi Xie contributed to the study conception, study design, project management, data interpretation and manuscript writing. Dr. Suad Hannawi and Dr. Liangzhi Xie have verified the data, and were responsible for submitting the manuscript. All authors critically reviewed and approved the final version of the manuscript.

## Data sharing statement

Anonymized participant data will be made available when the trials are complete, upon requests directed to the corresponding author. Proposals will be reviewed and approved by the sponsor, investigator, and collaborators on the basis of scientific merit. After approval of a proposal, data can be shared through a secure online platform after signing a data access agreement. All data will be made available for a minimum of 5 years from the end of the trial.

## Declaration of interests

Lixin Yan, Cuige Gao, Miaomiao Zhang, Yuanxin Chen and Wenlin Gai are employees of Sinocelltech Ltd., and Liangzhi Xie has potential stock option interests in the company. All authors declare no other conflicts of interest.
