# Supplementary material for: Safety and immunogenicity of multivalent SARS-CoV-2 protein vaccines: a randomized phase 3 trial
Source: eClinicalMedicine. 2023 Sep 8;64:102195. doi: 10.1016/j.eclinm.2023.102195 (PMC10507195; doi:10.1016/j.eclinm.2023.102195)
Supplement: Supplementary Materials 2 [file mmc1.docx]

**Supplementary Online Content**

[Table S1. Baseline Demographics of participants in Immunogenicity Group 2](#_Toc7376)

[Table S2. Subgroup Analysis of Live virus nAb against Omicron BA.1 by Baseline Characteristic 4](#_Toc20326)

[Table S3. Subgroup Analysis of Live virus nAb against Omicron BA.5 by Baseline Characteristic 7](#_Toc16564)

[Table S4. Subgroup Analysis of Live virus nAb against Delta by Baseline Characteristic 10](#_Toc13893)

[Table S5. Seroresponse to Omicron BA.1, BA.5 and Delta 13](#_Toc2832)

[Figure S1. Incidence of solicited local (A) and systemic (B) AEs. 14](#_Toc30314)

[Figure S2: GMTs of nAb against Omicron BA.1 among individuals with increase (A) and decrease (B) in GMT on day 180 compared to day 28. 15](#_Toc6303)

[Figure S3. Subgroup Analysis of Live Virus nAb Against Omicron BA.1 by Baseline Characteristics. 16](#_Toc24806)

[Figure S4. Subgroup Analysis of Live Virus nAb Against Omicron BA.5 by Baseline Characteristics. 17](#_Toc10318)

[Figure S5. Subgroup Analysis of Live Virus nAb Against Delta by Baseline Characteristics. 18](#_Toc10897)

**Table S1. Baseline Demographics of participants in Immunogenicity Group**

|  | BBIBP-CorV  (N=102) | SCTV01C  (N=100) | SCTV01E  (N=98) | Overall  (N=300) |
| --- | --- | --- | --- | --- |
| Age (years) |  |  |  |  |
| n | 102 | 100 | 98 | 300 |
| Mean (SD) | 30.4 (7.57) | 30.8 (7.72) | 30.4 (7.88) | 30.5 (7.70) |
| Median | 29.5 | 29.5 | 29.0 | 29.0 |
| Min, Max | 20, 50 | 20, 58 | 20, 53 | 20, 58 |
| 18-54 | 102 (100.0) | 99 (99.0) | 98 (100.0) | 299 (99.7) |
| >=55 | 0 | 1 (1.0) | 0 | 1 (0.3) |
| Gender, n (%) |  |  |  |  |
| Female | 0 | 1 (1.0) | 1 (1.0) | 2 (0.7) |
| Male | 102 (100.0) | 99 (99.0) | 97 (99.0) | 298 (99.3) |
| Race, n (%) |  |  |  |  |
| Asian | 98 (96.1) | 99 (99.0) | 97 (99.0) | 294 (98.0) |
| Black or African American | 3 (2.9) | 1 (1.0) | 1 (1.0) | 5 (1.7) |
| Other | 1 (1.0) | 0 | 0 | 1 (0.3) |
| BMI (kg/m^2) [2] |  |  |  |  |
| n | 102 | 100 | 98 | 300 |
| Mean (SD) | 25.34 (4.153) | 25.03 (4.328) | 25.56 (3.858) | 25.31 (4.111) |
| Median | 24.90 | 25.00 | 25.50 | 25.20 |
| Min, Max | 18.7, 41.7 | 17.0, 39.0 | 16.9, 37.2 | 16.9, 41.7 |
| Number of Prior COVID-19 Vaccine Doses |  |  |  |  |
| 1 | 0 | 0 | 0 | 0 |
| 2 | 80 (78.4) | 79 (79.0) | 77 (78.6) | 236 (78.7) |
| 3 | 22 (21.6) | 21 (21.0) | 21 (21.4) | 64 (21.3) |
| Infection History of COVID-19 |  |  |  |  |
| Yes | 0 | 0 | 0 | 0 |
| No | 102 (100.0) | 100 (100.0) | 98 (100.0) | 300 (100.0) |
| Interval from last COVID-19 vaccination (month)-Calculated ^[1]^ |  |  |  |  |
| 3-5 | 13 (12.7) | 9 (9.0) | 14 (14.3) | 36 (12.0) |
| 6-8 | 11 (10.8) | 16 (16.0) | 6 (6.1) | 33 (11.0) |
| 9-12 | 30 (29.4) | 20 (20.0) | 20 (20.4) | 70 (23.3) |
| 13-24 | 48 (47.1) | 55 (55.0) | 58 (59.2) | 161 (53.7) |

**Note:**[1] Interval is recalculated as (date of randomization – date of last vaccination) / 30

**Abbreviation:**BMI, Body Mass Index.

**Table S2. Subgroup Analysis of Live virus nAb against Omicron BA.1 by Baseline Characteristic**

|  | | | |
| --- | --- | --- | --- |
| **Subgroup** | **BBIBP-CorV**  **(N=100)** | **SCTV01C**  **(N=98)** | **SCTV01E**  **(N=95)** |
|  | | | |
|  | | | |
| **Number of Prior COVID-19 Vaccine Doses: 2** |  |  |  |
| Baseline (Day 0) |  |  |  |
| n | 78 | 78 | 74 |
| GMT (95% CI) | 76 (54,106) | 58 (42,80) | 71 (50,100) |
| Day 28 |  |  |  |
| n | 78 | 78 | 74 |
| GMT (95% CI) | 191 (141,260) | 1362 (1136,1634) | 2143 (1741,2637) |
| Fold Increase Over Baseline (95% CI) | 2.52 (1.84,3.44) | 23.66 (16.33,34.27) | 30.25 (20.33,45.02) |
| LS GMR vs. BBIBP-CorV (95% CI) |  | 7.71 (5.47,10.87) | 11.60 (8.11,16.59) |
| P value [1] |  | <.0001 | <.0001 |
|  | | | |
| **Number of Prior COVID-19 Vaccine Doses: 3** |  |  |  |
| Baseline (Day 0) |  |  |  |
| n | 22 | 20 | 21 |
| GMT (95% CI) | 181 (119,276) | 106 (57,196) | 61 (33,116) |
| Day 28 |  |  |  |
| n | 22 | 20 | 21 |
| GMT (95% CI) | 352 (205,603) | 937 (551,1594) | 1323 (696,2515) |
| Fold Increase Over Baseline (95% CI) | 1.94 (1.22,3.07) | 8.88 (4.09,19.27) | 21.53 (8.33,55.66) |
| LS GMR vs. BBIBP-CorV (95% CI) |  | 3.62 (1.70,7.68) | 4.38 (1.73,11.08) |
| P value [1] |  | 0.0014 | 0.0027 |
|  | | | |
| **Interval from last COVID-19 vaccination (month): 3-5** |  |  |  |
| Baseline (Day 0) |  |  |  |
| n | 13 | 8 | 14 |
| GMT (95% CI) | 169 (95,301) | 87 (37,203) | 57 (28,116) |
| Day 28 |  |  |  |
| n | 13 | 8 | 14 |
| GMT (95% CI) | 376 (181,778) | 905 (357,2292) | 1640 (804,3343) |
| Fold Increase Over Baseline (95% CI) | 2.23 (1.04,4.77) | 10.37 (3.26,32.98) | 28.98 (10.89,77.11) |
| LS GMR vs. BBIBP-CorV (95% CI) |  | 2.96 (0.89,9.81) | 5.42 (1.80,16.34) |
| P value [1] |  | 0.0732 | 0.0043 |
|  | | | |
| **Interval from last COVID-19 vaccination (month): 6-8** |  |  |  |
| Baseline (Day 0) |  |  |  |
| n | 11 | 16 | 6 |
| GMT (95% CI) | 85 (33,219) | 70 (24,209) | 40 (10,159) |
| Day 28 |  |  |  |
| n | 11 | 16 | 6 |
| GMT (95% CI) | 219 (105,456) | 987 (541,1801) | 1437 (211,9800) |
| Fold Increase Over Baseline (95% CI) | 2.57 (1.01,6.57) | 14.05 (3.96,49.87) | 35.92 (2.96,435.49) |
| LS GMR vs. BBIBP-CorV (95% CI) |  | 4.85 (1.97,11.91) | 8.24 (1.51,44.86) |
| P value [1] |  | 0.0014 | 0.0186 |
|  | | | |
| **Interval from last COVID-19 vaccination (month): 9-12** |  |  |  |
| Baseline (Day 0) |  |  |  |
| n | 29 | 20 | 20 |
| GMT (95% CI) | 84 (51,139) | 83 (55,124) | 86 (37,196) |
| Day 28 |  |  |  |
| n | 29 | 20 | 20 |
| GMT (95% CI) | 198 (119,332) | 1522 (1112,2083) | 2307 (1529,3481) |
| Fold Increase Over Baseline (95% CI) | 2.36 (1.57,3.56) | 18.38 (12.13,27.85) | 26.91 (10.38,69.74) |
| LS GMR vs. BBIBP-CorV (95% CI) |  | 7.77 (4.55,13.26) | 11.50 (5.95,22.23) |
| P value [1] |  | <.0001 | <.0001 |
|  | | | |
| **Interval from last COVID-19 vaccination (month): 13-24** |  |  |  |
| Baseline (Day 0) |  |  |  |
| n | 47 | 54 | 55 |
| GMT (95% CI) | 84 (52,134) | 56 (38,83) | 71 (48,104) |
| Day 28 |  |  |  |
| n | 47 | 54 | 55 |
| GMT (95% CI) | 200 (131,305) | 1330 (1056,1675) | 1940 (1497,2514) |
| Fold Increase Over Baseline (95% CI) | 2.39 (1.55,3.68) | 23.82 (14.93,38.00) | 27.51 (17.29,43.76) |
| LS GMR vs. BBIBP-CorV (95% CI) |  | 7.59 (4.85,11.88) | 10.15 (6.43,16.00) |
| P value [1] |  | <.0001 | <.0001 |

**Note:** [1] The comparison is based on ANCOVA model with intervention group, age group, number of prior COVID-19 vaccine doses, recalculated interval from last COVID-19 vaccination, and log-transformed pre-baseline value as covariates. Abbreviations: GMT,Geometric Mean titer; LS GMR, Least Square Geometric Mean Ratio.

**Table S3. Subgroup Analysis of Live virus nAb against Omicron BA.5 by Baseline Characteristic**

|  | | | |
| --- | --- | --- | --- |
| **Subgroup** | **BBIBP-CorV**  **(N=100)** | **SCTV01C**  **(N=98)** | **SCTV01E**  **(N=95)** |
|  | | | |
|  | | | |
| **Number of Prior COVID-19 Vaccine Doses: 2** |  |  |  |
| Baseline (Day 0) |  |  |  |
| N | 78 | 77 | 74 |
| GMT (95% CI) | 140 (103,190) | 136 (101,184) | 123 (90,169) |
| Day 28 |  |  |  |
| N | 78 | 77 | 74 |
| GMT (95% CI) | 303 (228,404) | 2340 (1986,2757) | 2785 (2350,3302) |
| Fold Increase Over Baseline (95% CI) | 2.17 (1.56,3.01) | 17.19 (12.28,24.07) | 22.63 (16.20,31.61) |
| LS GMR vs. BBIBP-CorV (95% CI) |  | 7.85 (5.66,10.87) | 9.70 (6.99,13.46) |
| P value [1] |  | <.0001 | <.0001 |
|  | | | |
| **Number of Prior COVID-19 Vaccine Doses: 3** |  |  |  |
| Baseline (Day 0) |  |  |  |
| N | 22 | 20 | 21 |
| GMT (95% CI) | 234 (143,381) | 149 (74,300) | 131 (72,239) |
| Day 28 |  |  |  |
| N | 22 | 20 | 21 |
| GMT (95% CI) | 412 (223,759) | 1749 (1062,2880) | 2171 (1319,3571) |
| Fold Increase Over Baseline (95% CI) | 1.76 (1.04,2.98) | 11.71 (4.50,30.46) | 16.54 (6.68,40.92) |
| LS GMR vs. BBIBP-CorV (95% CI) |  | 5.00 (2.18,11.47) | 5.58 (2.46,12.66) |
| P value [1] |  | 0.0004 | 0.0001 |
|  | | | |
| **Interval from last COVID-19 vaccination (month) : 3-5** |  |  |  |
| Baseline (Day 0) |  |  |  |
| N | 13 | 8 | 14 |
| GMT (95% CI) | 198 (124,315) | 147 (60,361) | 108 (47,246) |
| Day 28 |  |  |  |
| N | 13 | 8 | 14 |
| GMT (95% CI) | 490 (203,1183) | 1660 (522,5276) | 2319 (1321,4071) |
| Fold Increase Over Baseline (95% CI) | 2.48 (1.08,5.66) | 11.31 (2.40,53.24) | 21.53 (6.96,66.59) |
| LS GMR vs. BBIBP-CorV (95% CI) |  | 3.69 (0.88,15.54) | 4.69 (1.63,13.51) |
| P value [1] |  | 0.0725 | 0.0061 |
|  | | | |
| **Interval from last COVID-19 vaccination (month) : 6-8** |  |  |  |
| Baseline (Day 0) |  |  |  |
| N | 11 | 16 | 6 |
| GMT (95% CI) | 150 (55,412) | 174 (64,474) | 127 (53,306) |
| Day 28 |  |  |  |
| N | 11 | 16 | 6 |
| GMT (95% CI) | 282 (138,577) | 2153 (1224,3785) | 2560 (830,7900) |
| Fold Increase Over Baseline (95% CI) | 1.88 (0.81,4.37) | 12.34 (3.67,41.49) | 20.16 (3.08,131.87) |
| LS GMR vs. BBIBP-CorV (95% CI) |  | 7.83 (3.30,18.57) | 10.17 (3.06,33.75) |
| P value [1] |  | <.0001 | 0.0011 |
|  | | | |
| **Interval from last COVID-19 vaccination (month) : 9-12** |  |  |  |
| Baseline (Day 0) |  |  |  |
| N | 29 | 20 | 20 |
| GMT (95% CI) | 135 (85,216) | 135 (87,207) | 171 (87,336) |
| Day 28 |  |  |  |
| N | 29 | 20 | 20 |
| GMT (95% CI) | 328 (201,535) | 2307 (1674,3179) | 3152 (2123,4679) |
| Fold Increase Over Baseline (95% CI) | 2.42 (1.51,3.88) | 17.15 (9.93,29.60) | 18.38 (8.63,39.14) |
| LS GMR vs. BBIBP-CorV (95% CI) |  | 7.01 (3.85,12.77) | 9.13 (4.83,17.26) |
| P value [1] |  | <.0001 | <.0001 |
|  | | | |
| **Interval from last COVID-19 vaccination (month): 13-24** |  |  |  |
| Baseline (Day 0) |  |  |  |
| N | 47 | 53 | 55 |
| GMT (95% CI) | 162 (105,250) | 130 (89,189) | 115 (80,167) |
| Day 28 |  |  |  |
| N | 47 | 53 | 55 |
| GMT (95% CI) | 297 (202,437) | 2276 (1896,2731) | 2560 (2081,3149) |
| Fold Increase Over Baseline (95% CI) | 1.83 (1.16,2.88) | 17.53 (11.57,26.57) | 22.20 (14.91,33.06) |
| LS GMR vs. BBIBP-CorV (95% CI) |  | 8.08 (5.40,12.09) | 9.21 (6.14,13.82) |
| P value [1] |  | <.0001 | <.0001 |

**Note:** [1] The comparison is based on ANCOVA model with intervention group, age group, number of prior COVID-19 vaccine doses, recalculated interval from last COVID-19 vaccination, and log-transformed pre-baseline value as covariates.

Abbreviations: GMT,Geometric Mean titer; LS GMR, Least Square Geometric Mean Ratio.

**Table S4. Subgroup Analysis of Live virus nAb against Delta by Baseline Characteristic**

|  | | | |
| --- | --- | --- | --- |
| **Subgroup** | **BBIBP-CorV**  **(N=100)** | **SCTV01C**  **(N=98)** | **SCTV01E**  **(N=95)** |
|  | | | |
|  | | | |
| **Number of Prior COVID-19 Vaccine Doses: 2** |  |  |  |
| Baseline (Day 0) |  |  |  |
| n | 78 | 78 | 74 |
| GMT (95% CI) | 288 (209,397) | 288 (213,388) | 308 (229,414) |
| Day 28 |  |  |  |
| n | 78 | 78 | 74 |
| GMT (95% CI) | 618 (481,792) | 4363 (3679,5175) | 5025 (4156,6075) |
| Fold Increase Over Baseline (95% CI) | 2.15 (1.52,3.03) | 15.17 (10.73,21.44) | 16.30 (12.26,21.68) |
| LS GMR vs. BBIBP-CorV (95% CI) |  | 6.98 (5.17,9.43) | 8.07 (5.95,10.94) |
| P value [1] |  | <.0001 | <.0001 |
|  | | | |
| **Number of Prior COVID-19 Vaccine Doses: 3** |  |  |  |
| Baseline (Day 0) |  |  |  |
| n | 22 | 20 | 21 |
| GMT (95% CI) | 601 (385,937) | 538 (315,919) | 271 (150,489) |
| Day 28 |  |  |  |
| n | 22 | 20 | 21 |
| GMT (95% CI) | 877 (602,1277) | 3497 (2198,5564) | 3932 (2227,6943) |
| Fold Increase Over Baseline (95% CI) | 1.46 (0.94,2.27) | 6.50 (2.76,15.29) | 14.49 (6.28,33.45) |
| LS GMR vs. BBIBP-CorV (95% CI) |  | 4.29 (2.34,7.88) | 4.92 (2.42,10.01) |
| P value [1] |  | <.0001 | <.0001 |
|  | | | |
| **Interval from last COVID-19 vaccination (month): 3-5** |  |  |  |
| Baseline (Day 0) |  |  |  |
| n | 13 | 8 | 14 |
| GMT (95% CI) | 490 (245,983) | 381 (107,1352) | 276 (110,691) |
| Day 28 |  |  |  |
| n | 13 | 8 | 14 |
| GMT (95% CI) | 930 (532,1623) | 2792 (1135,6865) | 4873 (2739,8668) |
| Fold Increase Over Baseline (95% CI) | 1.90 (0.97,3.71) | 7.34 (1.69,31.82) | 17.67 (6.10,51.14) |
| LS GMR vs. BBIBP-CorV (95% CI) |  | 3.11 (1.22,7.94) | 5.66 (2.54,12.62) |
| P value [1] |  | 0.0206 | 0.0002 |
|  | | | |
| **Interval from last COVID-19 vaccination (month) : 6-8** |  |  |  |
| Baseline (Day 0) |  |  |  |
| n | 11 | 16 | 6 |
| GMT (95% CI) | 341 (110,1055) | 415 (173,993) | 254 (140,460) |
| Day 28 |  |  |  |
| n | 11 | 16 | 6 |
| GMT (95% CI) | 682 (337,1379) | 4695 (2655,8304) | 3225 (627,16584) |
| Fold Increase Over Baseline (95% CI) | 2.00 (0.53,7.59) | 11.31 (3.15,40.67) | 12.70 (1.84,87.82) |
| LS GMR vs. BBIBP-CorV (95% CI) |  | 7.33 (3.18,16.88) | 4.84 (1.08,21.64) |
| P value [1] |  | <.0001 | 0.0403 |
|  | | | |
| **Interval from last COVID-19 vaccination (month) : 9-12** |  |  |  |
| Baseline (Day 0) |  |  |  |
| n | 29 | 20 | 20 |
| GMT (95% CI) | 336 (236,477) | 355 (229,550) | 381 (228,636) |
| Day 28 |  |  |  |
| n | 29 | 20 | 20 |
| GMT (95% CI) | 596 (385,922) | 4159 (2976,5811) | 5301 (3372,8333) |
| Fold Increase Over Baseline (95% CI) | 1.77 (1.16,2.73) | 11.71 (7.45,18.41) | 13.93 (7.57,25.64) |
| LS GMR vs. BBIBP-CorV (95% CI) |  | 6.88 (3.97,11.93) | 8.62 (4.68,15.87) |
| P value [1] |  | <.0001 | <.0001 |
|  | | | |
| **Interval from last COVID-19 vaccination (month) : 13-24** |  |  |  |
| Baseline (Day 0) |  |  |  |
| n | 47 | 54 | 55 |
| GMT (95% CI) | 306 (192,489) | 289 (202,413) | 286 (200,408) |
| Day 28 |  |  |  |
| n | 47 | 54 | 55 |
| GMT (95% CI) | 650 (476,886) | 4278 (3520,5200) | 4747 (3793,5941) |
| Fold Increase Over Baseline (95% CI) | 2.12 (1.34,3.36) | 14.81 (9.72,22.59) | 16.62 (11.86,23.28) |
| LS GMR vs. BBIBP-CorV (95% CI) |  | 6.85 (4.83,9.72) | 7.48 (5.28,10.59) |
| P value [1] |  | <.0001 | <.0001 |

**Note:** [1] The comparison is based on ANCOVA model with intervention group, age group, number of prior COVID-19 vaccine doses, recalculated interval from last COVID-19 vaccination, and log-transformed pre-baseline value as covariates. Abbreviations: GMT,Geometric Mean titer; LS GMR, Least Square Geometric Mean Ratio.

**Table S5. Seroresponse to Omicron BA.1, BA.5 and Delta**

|  | **SCTV01E** | **SCTV01C** | **BBIBP-CorV** |
| --- | --- | --- | --- |
| **Omicron BA.1** |  |  |  |
| Seroresponse rate, n (%) | 88 (92.6) | 86 (87.8) | 28 (28.0) |
| 95% CI ^[1]^ | 85.4, 97.0 | 79.6, 93.5 | 19.5, 37.9 |
| Increase over BBIBP-CorV (95% CI) | 65.0 (54.8, 75.3) | 62.2 (51.8, 72.6) |  |
| P value ^[2]^ | <.0001 | <.0001 |  |
| **Omicron BA.5** |  |  |  |
| Seroresponse rate, n (%) | 88 (92.6) | 86 (88.7) | 23 (23.0) |
| 95% CI ^[1]^ | 85.4, 97.0 | 80.6, 94.2 | 15.2, 32.5 |
| Increase over BBIBP-CorV (95% CI) | 69.7 (59.7, 79.6) | 67.9 (57.9, 77.8) |  |
| P value ^[2]^ | <.0001 | <.0001 |  |
| **Delta** |  |  |  |
| Seroresponse rate, n (%) | 88 (92.6) | 85 (86.7) | 18 (18.0) |
| 95% CI ^[1]^ | 85.4, 97.0 | 78.4, 92.7 | 11.0, 26.9 |
| Increase over BBIBP-CorV (95% CI) | 74.4 (65.0, 83.7) | 70.7 (60.9, 80.5) |  |
| P value ^[2]^ | <.0001 | <.0001 |  |

**Note:** Seroresponse for participants with pre-dose < LLOQ is defined as equal to or above LLOQ; seroresponse for participants with pre-dose ≥ LLOQ is defined as ≥4-fold increase in titers compared to pre-dose titer.

1. : 95% CI of seroresponse rate is based on Clopper-Pearson exact method. [2]: the comparisons between SCTV01C or SCTV01E and BBBIBP-CoV are based on Cochran-Mantel-Haenszel test (CMH) stratified by randomization stratification factors.


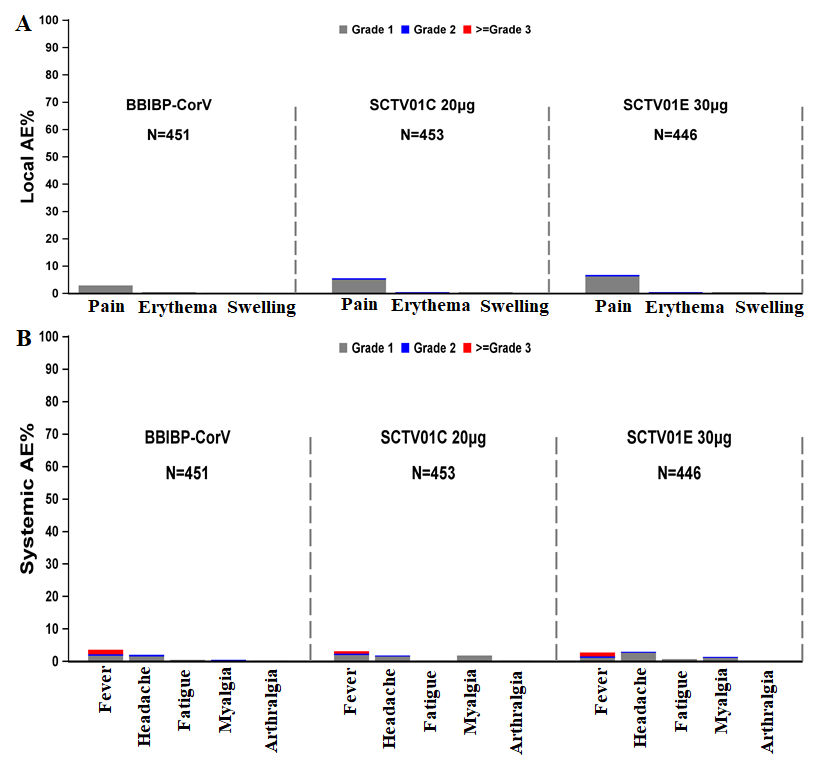


**Figure S1. Incidence of solicited local (A) and systemic (B) AEs**.

The grading scales are derived from the Toxicity Rating Scale for Healthy Adult and Adolescent Volunteers in Preventive Vaccine Clinical Trial-FDA Standard (Grade 1: mild, Grade 2: moderate or Grade 3: severe). The percentages of participants in each group with adverse events during the 7 days after vaccination are plotted for solicited local (Panel A) and systemic (Panel B) adverse events. The most frequent solicited AEs after vaccination were Grade 1 injection-site pain and fever. The overall frequencies and severities of solicited AEs were similar across three groups.


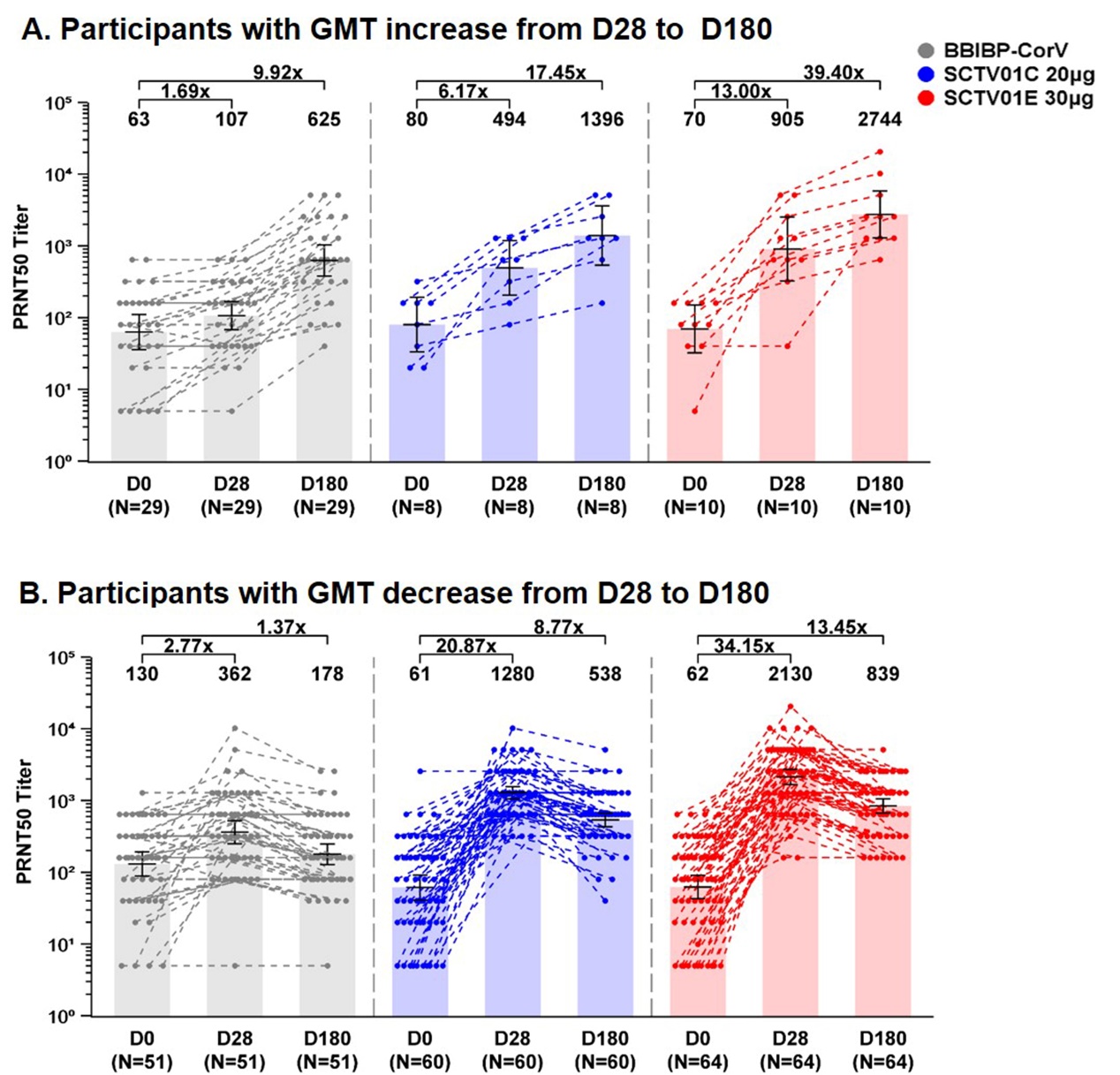


**Figure S2**: GMTs of nAb against Omicron BA.1 among individuals with increase (A) and decrease (B) in GMT on day 180 compared to day 28.

Note: Grey, BBIBP-CorV; green, SCTV01C; red, SCTV01E.

Abbreviations: GMT, Geometric Mean titer.


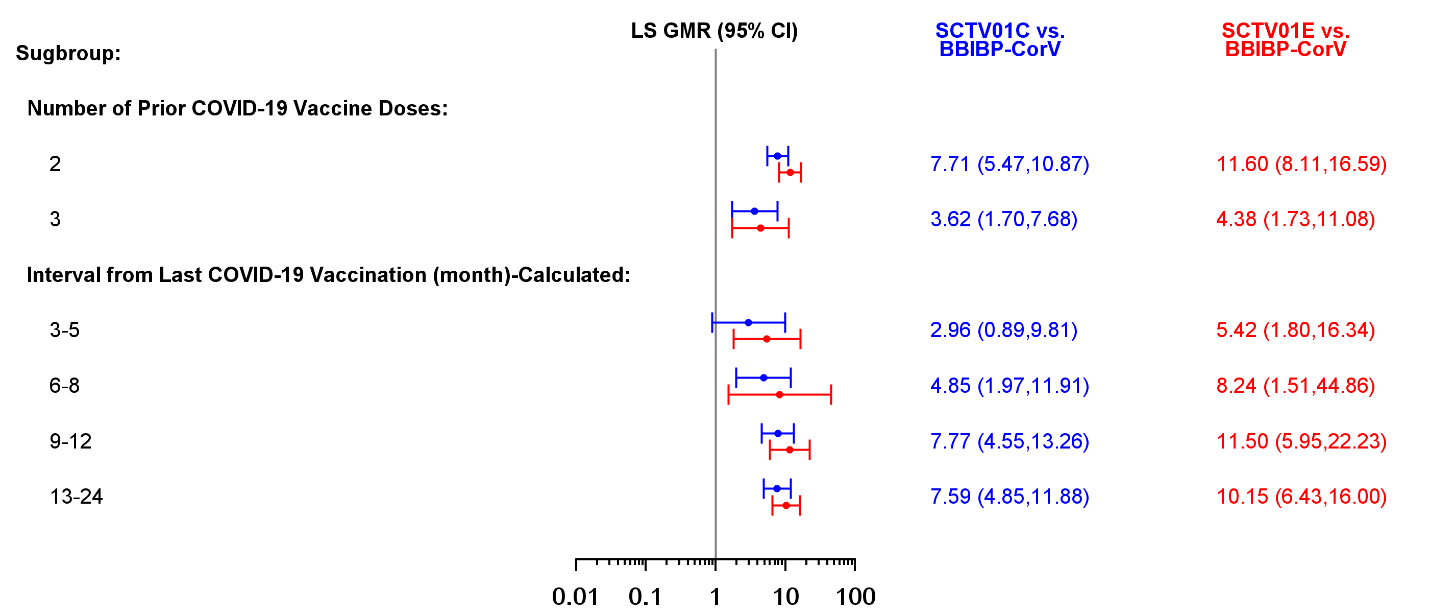


**Figure S3. Subgroup Analysis of Live Virus nAb Against Omicron BA.1 by Baseline Characteristics.**

Note: Subjects who were COVID-19 infected between Day 0 and Day 28 were excluded from analysis. Blue, SCTV01C vs. BBIBP-CorV; red, SCTV01E vs. BBIBP-CorV. Abbreviations:  LS GMR, Least Square Geometric Mean Ratio.


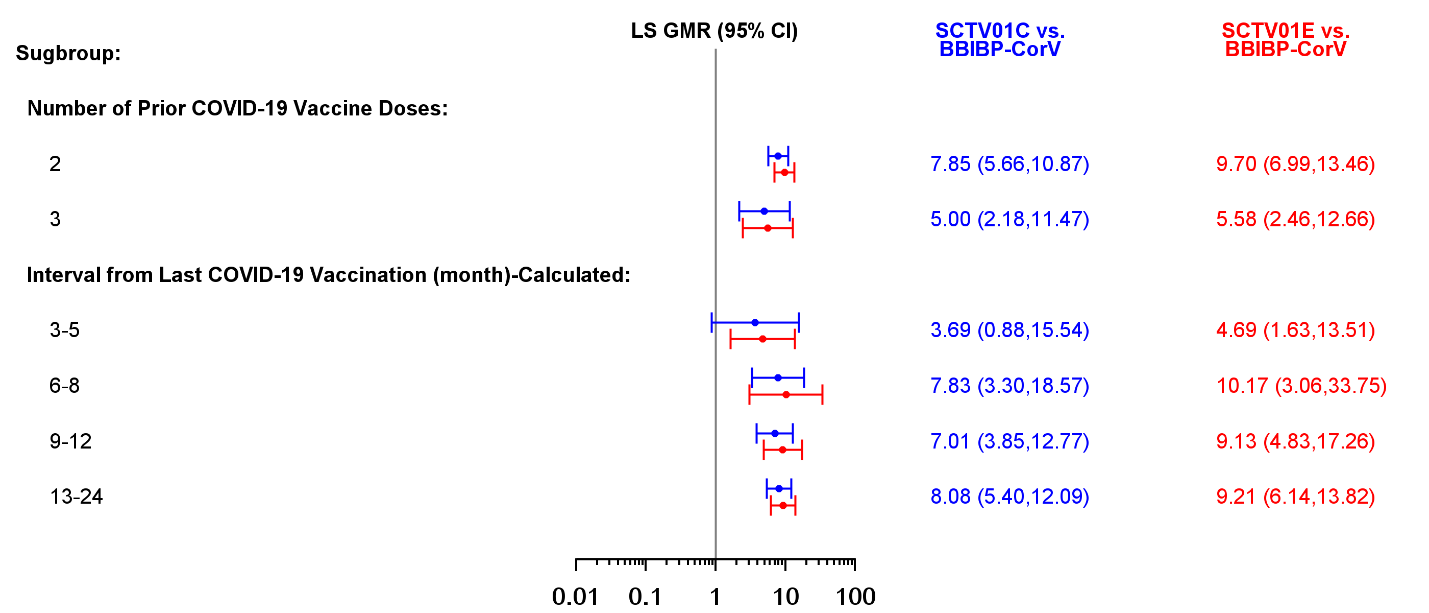


**Figure S4. Subgroup Analysis of Live Virus nAb Against Omicron BA.5 by Baseline Characteristics.**

Note: Subjects who were COVID-19 infected between Day 0 and Day 28 were excluded from analysis. Blue, SCTV01C vs. BBIBP-CorV; red, SCTV01E vs. BBIBP-CorV.

Abbreviations: LS GMR, Least Square Geometric Mean Ratio.


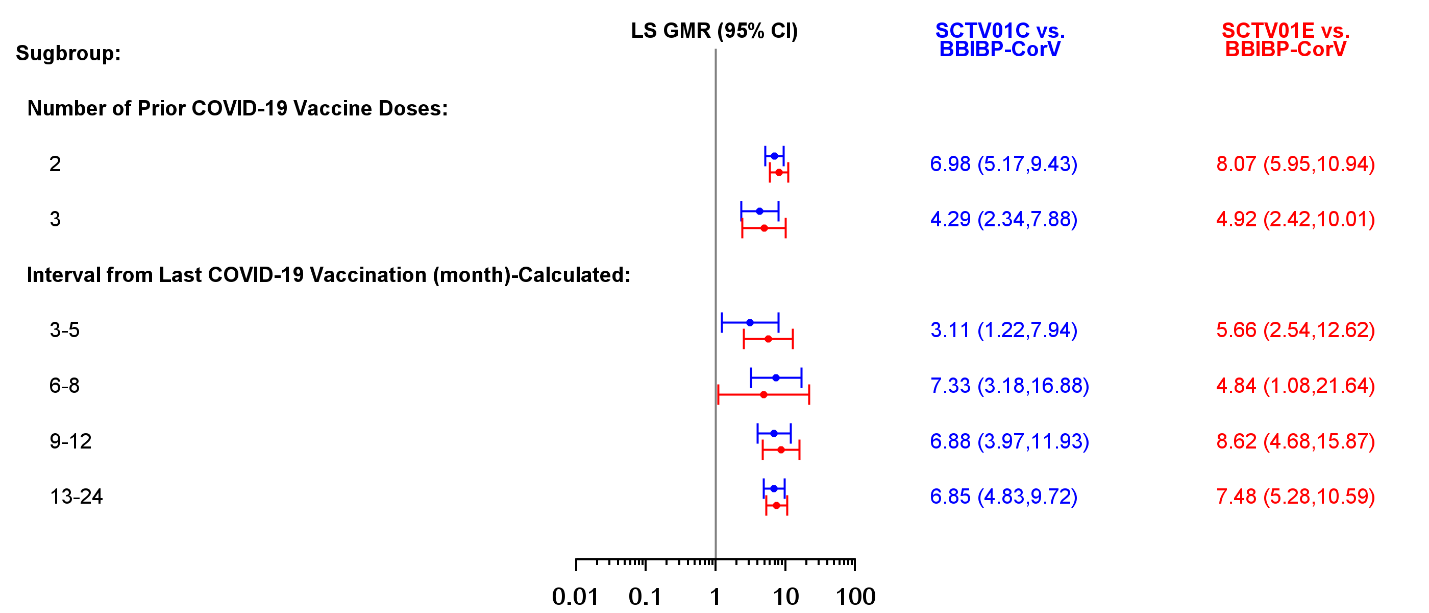


**Figure S5. Subgroup Analysis of Live Virus nAb Against Delta by Baseline Characteristics.**

Note: Subjects who were COVID-19 infected between Day 0 and Day 28 were excluded from analysis. Blue, SCTV01C vs. BBIBP-CorV; red, SCTV01E vs. BBIBP-CorV.

Abbreviations:  LS GMR, Least Square Geometric Mean Ratio.
