# Supplementary material for: Safety and immunogenicity of multivalent SARS-CoV-2 protein vaccines: a randomized phase 3 trial
Source: eClinicalMedicine. 2023 Sep 8;64:102195. doi: 10.1016/j.eclinm.2023.102195 (PMC10507195; doi:10.1016/j.eclinm.2023.102195)
Supplement: Supplementary Materials 1 [file mmc2.pdf]

This supplement contains the following items:

1) Original protocol, final protocol, and summary of changes.
2) Final statistical analysis plan, first and only version.

Clinical Trial Protocol

A randomized, double-blind, and positive-controlled Phase III clinical trial to evaluate the immunogenicity and safety of SCTV01C (A Bivalent SARS-CoV-2 Trimeric Spike Protein Vaccine) and SCTV01E (A COVID-19 Alpha/Beta/Delta/Omicron Variants S-Trimer Vaccine) in population aged ≥18 years previously vaccinated with either inactivated or mRNA COVID-19 vaccine or previously diagnosed with COVID-19

**Protocol No.:**	SCTV01C/E-01-UAE-1
**Protocol Version No.:**	Version 1.0
**Version date:**	March 9, 2022
**Sponsor:**	Sinocelltech Ltd.

Confidentiality Statement

All information in this protocol constitutes proprietary property of Sinocelltech Ltd. Therefore, it is only provided to investigators, co-investigators, ethics committees, regulatory authorities, and other relevant medical institutions for review. Without the written approval of Sinocelltech Ltd., it is strictly forbidden to inform any third party unrelated to this study of any information herein, except where required by applicable law. In the event of any actual or suspected breach of this obligation, Sinocelltech Ltd. must be promptly notified.

Protocol Signature Page

I agree to:

- Conduct this study in strict accordance with the protocol, quality management practices of clinical drug trials and relevant laws and regulations.
- Keep all materials and information provided by Sinocelltech Ltd. in accordance with confidentiality requirements and indicate that they are confidential when submitted to Institution Review Committee or Independent Ethics Committee.

I have read the protocol in full and agree with all requirements.

________________________	________________________	________________________
Director of Sponsor	Signature	Date

Protocol Signature Page

I agree to:

- Follow this study in strict accordance with the protocol, quality management practices of clinical drug trials and relevant laws and regulations.
- Keep all materials and information provided by Sinocelltech Ltd. in accordance with confidentiality requirements and indicate that they are confidential when submitted to Institution Review Committee or Independent Ethics Committee.

I have read the protocol in full and agree with all requirements.

______________________	______________________	______________________
Statistician	Signature	Date

Protocol Signature Page

I agree to:

- Conduct this study in strict accordance with the protocol, quality management practices of clinical drug trials and relevant laws and regulations.
- Keep all materials and information provided by Sinocelltech Ltd. in accordance with confidentiality requirements and indicate that they are confidential when submitted to Institution Review Committee or Independent Ethics Committee.

I have read the protocol in full and agree with all requirements.

______________________	______________________	______________________
Principal Investigator	Signature	Date

PROTOCOL SYNOPSIS

**Protocol No.**	SCTV01C/E-01-UAE-1
**Protocol Title**	A randomized, double-blind, and positive-controlled Phase III clinical trial to evaluate the immunogenicity and safety of SCTV01C (A Bivalent SARS-CoV-2 Trimeric Spike Protein Vaccine) and SCTV01E (A COVID-19 Alpha/Beta/Delta/Omicron Variants S-Trimer Vaccine) in population aged ≥18 years previously vaccinated with either inactivated or mRNA COVID-19 vaccine or previously diagnosed with COVID-19
**Version No.**	Version 1.0
**Version Date**	March 9, 2022
**Sponsor**	Sinocelltech Ltd.
**Study Phase**	Phase III
**Indication**	Prevention of COVID-19 (COVID-19 in this protocol refers to COVID-19 patients diagnosed according to the US FDA standards)
**Target Population**	Individuals aged ≥18 years who were previously vaccinated with either inactivated or mRNA COVID-19 vaccine or previously diagnosed with COVID-19
**Study Objectives**	**Primary Objective:**<br>• To evaluate the immunogenicity of SCTV01C;<br>• To evaluate the immunogenicity of SCTV01E.<br>**Secondary Objective:**<br>• To evaluate the cellular immune response of SCTV01C;<br>• To evaluate the cellular immune response of SCTV01E;<br>• To evaluate the safety of SCTV01C within 180 days after the vaccination;

	• To evaluate the safety of SCTV01E within 180 days after the vaccination.
**Study endpoint**	**Primary endpoints**<br>**Cohort 1**<br>***Immunogenicity***<br>• Geometric mean titer (GMT)of neutralizing antibodies (nAb) against Delta variant on D28;<br>• GMT of nAb against Omicron variant on D28.<br>**Cohort 2**<br>***Immunogenicity***<br>• GMT of nAb against Delta variant on D28.<br>**Secondary endpoints:**<br>**Cohort 1**<br>***Immunogenicity***<br>• GMT of nAb against Delta variant on D180;<br>• GMT of nAb against Omicron variant on D180.<br>• Number of IFN-γ positive (characterizing Th1) and IL-4 positive (characterizing Th2) T cell subsets on D28;<br>• Seroresponse of nAb (defined as a change from below the low limit of quantitation [LLOQ] to equal to or above LLOQ, or a ≥4-fold rise if baseline is equal to or above LLOQ in nAb to Delta variant from D0) rates on D28;<br>• Seroresponse of nAb (defined as a change from below LLOQ to equal to or above LLOQ, or a ≥4-fold rise if baseline is equal to or above LLOQ in nAb to Omicron variant from D0) rates on D28;<br>***Safety***<br>• Incidence and severity of solicited AEs of SCTV01C from D0 to D7.<br>• Incidence and severity of all unsolicited AEs of SCTV01C from D0 to D28;<br>• Incidence and severity of SAEs and AESIs of SCTV01C within 180 days;

	• Incidence and severity of solicited AEs of SCTV01E from D0 to D7;<br>• Incidence and severity of all unsolicited AEs of SCTV01E from D0 to D28;<br>• Incidence and severity of SAEs and AESIs of SCTV01E within 180 days.<br>**Cohort 2**<br>***Immunogenicity***<br>• GMT of nAb against Omicron variant on D28;<br>• GMT of nAb against Delta variant on D180;<br>• GMT of nAb against Omicron variant on D180;<br>• Number of IFN-γ positive (characterizing Th1) and IL-4 positive (characterizing Th2) T cell subsets on D28;<br>• Seroresponse of nAb (defined as a change from below the low limit of quantitation [LLOQ] to equal to or above LLOQ, or a ≥4-fold rise if baseline is equal to or above LLOQ in nAb to Delta variant from D0) rates on D28;<br>• Seroresponse of nAb (defined as a change from below LLOQ to equal to or above LLOQ, or a ≥4-fold rise if baseline is equal to or above LLOQ in nAb to Omicron variant from D0) rates on D28.<br>***Safety***<br>• Incidence and severity of solicited AEs of SCTV01C from D0 to D7;<br>• Incidence and severity of all unsolicited AEs of SCTV01C from D0 to D28;<br>• Incidence and severity of SAEs and AESIs of SCTV01C within 180 days;<br>• Incidence and severity of solicited AEs of SCTV01E from D0 to D7;<br>• Incidence and severity of all unsolicited AEs of SCTV01E from D0 to D28;<br>• Incidence and severity of SAEs and AESIs of SCTV01E within 180 days.
**Study Design**	Although there is no clinical data for SCTV01E so far, SCT had initiated three clinical Phase I/II trials for SCTV01C to evaluate the safety and

	immunogenicity, which can be instructive and meaningful for SCTV01E clinical study consideration because of the same manufacturing processes, extremely similar molecular characteristics and clinical dosing between SCTV01E and SCTV01C. The SCTV01C trials will provide supportive safety and immunogenicity clinical data prior to the start of SCTV01E trials. The details of these trials are summarized in investigator's brochure.<br>SCTV01E, the quadrivalent vaccine, will be tested in this Phase III immunogenicity study based on the clinical data on safety, reactogenicity, and immunogenicity generated with the bivalent vaccine (SCTV01C) similarity in manufacturing process for four TM (trimeric drug substance) components of the quadrivalent product compared to the bivalent product; similarity in construct design supporting a similar safety profile of the quadrivalent product to that of the bivalent vaccine. The dose strength of SCTV01E is 30μg (5/5/5/15μg for TM22/TM23/TM28/TM41)/dose based on the nonclinical study of SCTV01E and SCTV01C in combination with the clinical studies of SCTV01C.<br>The study is a randomized, double-blind, and positive-controlled Phase III booster study. It will evaluate the immunogenicity and safety of one dose of SCTV01C or SCTV01E as booster compared with either one dose of Sinopharm inactivated COVID-19 vaccine (Cohort 1) or one dose of mRNA-1273 (Cohort 2).<br>Approximately 1,800 participants aged 18 years old and above will be enrolled in this study. 1,350 participants who previously received Sinopharm inactivated COVID-19 vaccine will be enrolled to Cohort 1. 450 participants who previously received mRNA COVID-19 vaccine (Comirnaty from Pfizer or mRNA-1273 from Moderna) or previously diagnosed with COVID-19 will be enrolled to Cohort 2.<br>In Cohort 1, 300 participants who were previously fully vaccinated with 2 or 3 doses of Sinopharm inactivated COVID-19 vaccine and with no previous COVID-19 history will form an immunogenicity subgroup (Subgroup 1) for nAb tests, and will be randomly assigned to SCTV01C Group, SCTV01E Group and the Sinopharm inactivated COVID-19 vaccine Group in a ratio of 1:1:1. The 300 participants for nAb tests will

	be stratified by age (18-54 years, ≥55 years), number of doses of previously received COVID-19 vaccines (2, 3), and interval between previous vaccination and the study vaccination (3-5 months, 6-8 months, 9-12 months, 13-24 months). The first 150 participants will form a cellular immune response subgroup for cellular immune response tests.<br>In Cohort 1, in addition to the 300 participants for immunogenicity tests, there are 1050 other participants who previously received at least one shot of Sinopharm COVID-19 inactivated vaccine, will form a subgroup (Subgroup 2) mainly for safety observation, and will be randomly assigned to SCTV01C Group, SCTV01E Group and Sinopharm inactivated COVID-19 vaccine Group in a ratio of 1:1:1. The 1050 participants mainly for safety observation will be stratified by age (18-54 years, ≥55 years), previous COVID-19 infection history (yes or no), number of doses of previously received COVID-19 vaccines (1, 2, 3) and interval between previous vaccination and the study vaccination (3-5 months, 6-8 months, 9-12 months, 13-24 months).<br>In Cohort 2, 450 participants who previously received 2 or 3 doses of mRNA COVID-19 vaccine (Comirnaty or mRNA-1273) or previously diagnosed with COVID-19 will be randomly assigned to SCTV01C Group, SCTV01E Group and the mRNA-1273 Group in a ratio of 1:1:1. Participants will be stratified by age (18-54 years, ≥55 years), number of doses of previously received COVID-19 vaccines (0, 1, 2, 3), previous COVID-19 infection history (yes or no), and interval between previous vaccination and the study vaccination (3-5 months, 6-8 months, 9-12 months, 13-24 months). In Cohort 2, the number of participants previously diagnosed with COVID-19 and previously not received any mRNA COVID-19 vaccine, should not be more than 50. All participants will have nAb tests. The first 150 participants will form a cellular immune subgroup for cellular immune response tests.<br>In Cohort 1, each participant in SCTV01C Group will receive one dose of SCTV01C on D0; each participant in SCTV01E Group will receive one dose of SCTV01E on D0; each participant in Sinopharm inactivated

COVID-19 vaccine Group will receive one dose of Sinopharm inactivated COVID-19 vaccine on D0.

In Cohort 2, each participant in SCTV01C Group will receive one dose of SCTV01C on D0; each participant in SCTV01E Group will receive one dose of SCTV01E on D0; each participant in mRNA-1273 Group will receive one dose of mRNA-1273 on D0.

Trial procedures:

The study procedure is described as Figure A and Figure B. An independent data and safety monitoring board (DSMB) will review the data of the study.

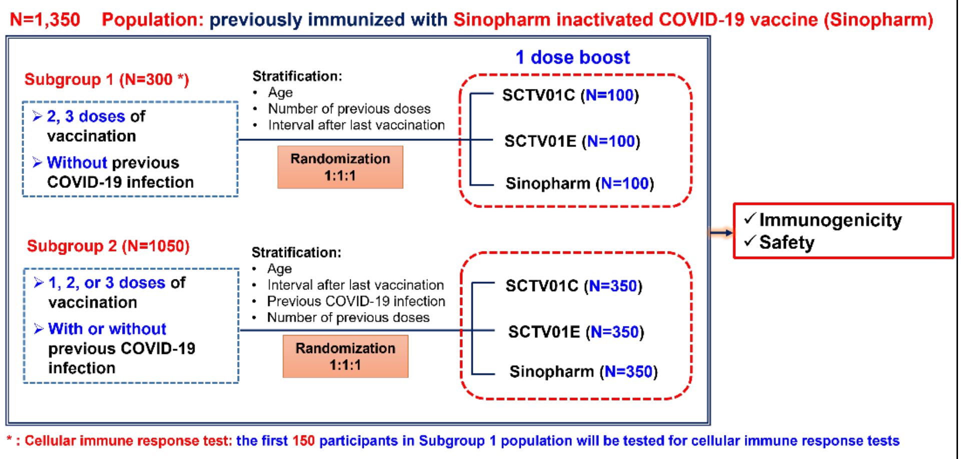

Figure A Study design for Cohort 1

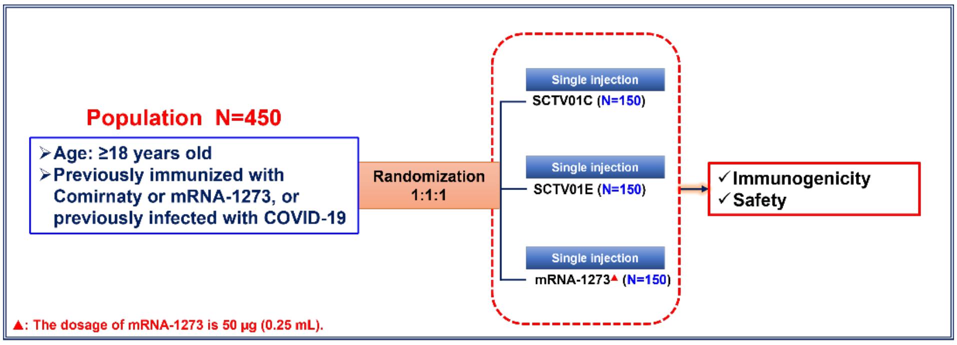

Figure B Study design for Cohort 2

The study consists of a screening period, a randomization and vaccination period, and a follow-up period.

Screening period: After participants sign the ICF, the screening phase visit will be conducted within 14 days.

Randomization: The qualified participants will be randomized before the study vaccination.

Vaccination: Randomized participants will be vaccinated on D0.

	**Follow-up period:**<br>**Safety follow-up:** All participants will be observed at site for at least 30 minutes after the study vaccination. Both the active monitoring and the spontaneous reporting will be used to collect the solicited and the unsolicited AEs. Solicited AEs within 7 days after study vaccination and unsolicited AEs within 28 days after study vaccination will be collected through vaccination record cards. AEs, SAEs and AESIs will be followed for 180±7 days after the study vaccination.<br>**Immunogenicity follow-up:** The participants in Subgroup 1 in Cohort 1 and all participants in Cohort 2 will be sampled for immunogenicity on D0 (before vaccination), D28 and D180. The nAb against Delta and Omicron variants will be tested.<br>The participants in the cellular immune response subgroup will be sampled for cellular immune response test on D0 (before vaccination) and D28.<br>After administration of the study vaccination, the participants will be continuously and systematically monitored for 180±7 days to ensure a prompt diagnosis and treatment according to FDA diagnosis and treatment practice when a participant experiences the suspicious symptoms of COVID-19. If a SARS-CoV-2 infection is confirmed 14 days after the study vaccination, sample will be collected from the nasopharyngeal/throat swab and viral sequencing will be used to identify the major SARS-CoV-2 variants.<br>The DSMB will review the safety data within D0-D28 of all the participants to assess the safety of SCTV01C and SCTV01E.<br>**Note:**<br>Participants aged between 54 years to less than 55 years old will be taken as 54 years old.
**Total Number of Participants**	1.800 participants are planned to be enrolled
**Study Site**	United Arab Emirates.

**Study Duration**	Each participant will be followed up for about 180 days after the vaccination on D0.
**Inclusion Criteria**	Participants are eligible to be included in the study only if the following conditions are met:<br>1. Male or female aged ≥18 years old when signing ICF;<br>2. **For Subgroup 1 in Cohort 1:** Participants who were previously vaccinated with 2 or 3 doses of Sinopharm inactivated COVID-19 vaccine. The interval between the date of last dose and the date of this study vaccination should be 3 to 24 months.<br>**For Subgroup 2 in Cohort 1:** 1) Participants who were previously vaccinated with 2 or 3 doses of Sinopharm inactivated COVID-19 vaccine, with or without COVID-19 history; or 2) Participants who were previously vaccinated with 1 dose of Sinopharm inactivated COVID-19 vaccine and previously diagnosed with COVID-19. The interval between the date of last dose/COVID-19 diagnosis and the date of this study vaccination should be 3 to 24 months.<br>**For Cohort 2:** 1) Participants who were previously vaccinated with 2 or 3 doses of mRNA COVID-19 vaccine (Comirnaty or mRNA-1273) , with or without COVID-19 history; or 2) Participants who were previously vaccinated with 1 doses of mRNA COVID-19 vaccine (Comirnaty or mRNA-1273) and previously diagnosed with COVID-19; 3) Participants who were previously not vaccinated with any COVID-19 vaccine and previously diagnosed with COVID-19. The interval between the date of last dose/COVID-19 diagnosis and the date of this study vaccination should be 3 to 24 months.<br>3. The participant and/or his legally acceptable representative can sign written ICF, and can fully understand the trial procedure, the risk of participating in the trial, and other interventions that can be selected if

	they do not participate in the trial;<br>4. The participant and/or his legally acceptable representative have the ability to read, understand, and fill in record cards;<br>5. Healthy participants or participants with pre-existing medical conditions who are in stable condition. The “pre-existing medical conditions” include but not limited to hypertension, diabetes, Chronic cholecystitis and cholelithiasis, chronic gastritis that meet the described criteria. A stable medical condition is defined as disease not requiring significant change in therapy or no need for hospitalization as a consequence of worsening disease state for at least 3 months prior to enrollment;<br>6. Fertile men and women of childbearing potential voluntarily agree to take effective contraceptive measures from signing ICF to 6 months after the last dose of study vaccination; the pregnancy test results of women of childbearing potential are negative on screening.
**Exclusion Criteria**	A participant who conforms to any of the following criteria should be excluded from the study:<br>1. **For Subgroup 1 in Cohort 1 only**: Previously diagnosed with COVID-19.<br>2. Presence of fever within 3 days before the study vaccination;<br>3. A history of infection or disease related to severe acute respiratory syndrome (SARS), Middle East respiratory syndrome (MERS), or other disease corresponding use of immunosuppressants;<br>4. A history of allergic reactions to any vaccine or drug, such as allergy, urticaria, severe skin eczema, dyspnea, laryngeal edema, and angioneurotic edema;<br>5. A medical or family history of seizure, epilepsy, encephalopathy and psychosis;

	6. Immunocompromised patients suffering from immunodeficiency diseases, important organ diseases, immune diseases (including Guillain-Barre Syndrome [GBS], systemic lupus erythematosus, rheumatoid arthritis, asplenia or splenectomy caused by any circumstances, and other immune diseases that may have an impact on immune response in the investigator's opinion), etc.;<br>7. Long-term use of immunosuppressant therapy or immunomodulatory drugs for ≥14 days within the six months prior to enrollment. Whereas short-term (≤14 days) use of oral, inhaled and topical steroids are allowed;<br>8. Patients on antituberculosis therapy;<br>9. Presence of severe or uncontrollable cardiovascular diseases, or severe or uncontrollable disorders related to endocrine system, blood and lymphatic system, liver and kidney, respiratory system, metabolic and skeletal systems, or malignancies (skin basal cell carcinoma and carcinoma in-situ of cervix are exceptions and will not be excluded), such as severe heart failure, severe pulmonary heart disease, unstable angina, liver failure, or uremia;<br>10. Contraindications for intramuscular injection or intravenous blood sampling, including thrombocytopenia and other blood coagulation disorders;<br>11. Participants who received any immunoglobulin or blood products in the previous 3 months before enrollment, or plan to receive similar products during the study;<br>12. Participants who received other investigational drugs within 1 month before the study vaccination;<br>13. Participants who is at the acute state of disease, such as acute onset of chronic heart failure, acute sore throat, hypertensive encephalopathy,

	acute pneumonia, acute renal insufficiency, acute cholecystitis;<br>14. Participants received other drugs or vaccines used to prevent COVID-19, but participants previously received Sinopharm inactivated COVID-19 vaccine, Comirnaty or mRNA-1273 will not be excluded ;<br>15. Participants vaccinated with influenza vaccine within 14 days or with other vaccines within 28 days before the study vaccination;<br>16. Those who donated blood or had blood loss (≥450 mL) within 3 months before the vaccination or plan to donate blood during the study period;<br>17. Those who are pregnant or breast-feeding or plan to be pregnant during the study period;<br>18. Those who plan to donate ovum or sperms during the study period;<br>19. Those who cannot follow the trial procedures, or cannot cooperate to complete the study due to planned relocation or long-term outing;<br>20. Those unsuitable for participating in the clinical trial as determined by the investigator because of other abnormalities that are likely to confuse the study results, or non-conformance with the maximal benefits of the participants;<br>21. Those who are tested positive for HIV in terms of serology.
**Withdrawal Criteria**	**A participant may withdraw from the study at any time at his/her own request.**<br>**Reasons for discontinuation from the study may include the following:**<br>• Refused further follow-up;<br>• Lost to follow-up;<br>• Death;<br>• Study terminated by sponsor;<br>• AEs;<br>• Participant request;

	• Investigator request;<br>• Protocol deviation.
**Study Suspension/Termination Criteria**	**In one of the following situations, the trial should be suspended or terminated:**<br>• When the DSMB requires a suspension/complete termination of the trial and the sponsor agrees;<br>• When the sponsor requires a suspension/complete termination of the trial and gives reasons for it;<br>• When the Ethics Committee requires a suspension/complete termination of the trial and gives reasons for it;<br>• When the regulatory agency requires a suspension/complete termination of the trial and gives reasons for it.
**Study Vaccine**	**Study Vaccine 1:** a bivalent SARS-CoV-2 trimeric spike protein vaccine (SCTV01C)<br>Appearance: emulsified, white suspension (due to the presence of adjuvant);<br>Components:<br>• Main active ingredients: SCTV01C-TM22 protein, SCTV01C-TM23 protein;<br>• SCT-VA02B adjuvant: the adjuvant 1X is comprised of 0.09 mg of citric acid, 0.59 mg of sodium citrate, 1.25 mg of polysorbate 80, 1.25 mg of span 85 and 10.75 mg of squalene;;<br>• Excipients: citrate, sodium citrate, sodium chloride, polysorbate 80, sodium hydroxide, WFI;<br>Dosage form: solution for injection;<br>Strength: 20μg(10/10μg for TM22/TM23) /0.5mL/vial;<br>Route of vaccination: intramuscular injection into the lateral deltoid of the upper arm;

	Dosage of vaccination: 20µg;<br>Immunization procedure: 1 dose, inoculated with 1 dose on D0;<br>Storage conditions: stored and transported at 2 ~ 8°C away from light;<br>Validity period: tentatively 24 months;<br>Manufacturer: Sinocelltech Ltd.<br>**Study Vaccine 2:** a COVID-19 Alpha/Beta/Delta/Omicron Variants S-Trimer Vaccine (SCTV01E);<br>Appearance: emulsified, white suspension(due to the presence of adjuvant);<br>Components:<br>• Main active ingredients: SCTV01E-TM22 protein, SCTV01E-TM23 protein, SCTV01E-TM28 protein, and SCTV01E-TM41 protein;<br>• SCT-VA02B (1×) adjuvant: the adjuvant 1X is comprised of 0.09 mg of citric acid, 0.59 mg of sodium citrate, 1.25 mg of polysorbate 80, 1.25 mg of span 85 and 10.75 mg of squalene.<br>• Excipients: citrate, sodium citrate, sodium chloride, polysorbate 80, sodium hydroxide, WFI;<br>Dosage form: solution for injection;<br>Strength: 30µg (5/5/5/15µg for TM22/TM23/TM28/TM41)/0.5mL/vial;<br>Route of vaccination: intramuscular injection into the lateral deltoid of the upper arm;<br>Dosage of vaccination: 30µg;<br>Immunization procedure: different procedures according to the study design<br>Storage conditions: stored and transported at 2 ~ 8℃ away from light;<br>Validity period: tentatively 24 months;<br>Manufacturer: Sinocelltech Ltd.

	**Sinopharm inactivated COVID-19 vaccine**: The dosage is 0.5 mL. The strength of Sinopharm inactivated COVID-19 vaccine is 6.5U/0.5mL/vial. The vaccine will be stored and transported at 2~8℃ away from light.<br>**mRNA-1273:** The dosage of mRNA-1273 is 50 µg (0.25 mL). The vaccine will be stored and transported at -50~-15°C away from light.
**Statistical Analysis**	Detailed methodology for summary and statistical analyses of the data collected in this study is outlined here and further detailed in a statistical analysis plan (SAP). The SAP may modify what is outlined in the protocol where appropriate; however, any major modifications of the primary endpoint definitions or their analyses will also be reflected in a protocol amendment.<br>**Hypothesis:**<br>$GMTC1_{Delta}$= GMT of nAb against Delta variant on D28 of SCTV01C in Cohort 1;<br>$GMTE1_{Delta}$= GMT of nAb against Delta variant on D28 of SCTV01E in Cohort 1;<br>$GMTS1_{Delta}$= GMT of nAb against Delta variant on D28 of Sinopharm inactivated vaccine in Cohort 1;<br>$GMTC1_{Omicron}$= GMT of nAb against Omicron variant on D28 of SCTV01C in Cohort 1;<br>$GMTE1_{Omicron}$ = GMT of nAb against Omicron variant on D28 of SCTV01E in Cohort 1;<br>$GMTS1_{Omicron}$ = GMT of nAb against Omicron variant on D28 of Sinopharm inactivated vaccine in Cohort 1;<br>$GMTC2_{Delta}$= GMT of nAb against Delta variant on D28 of SCTV01C in Cohort 2;

$GMTE2_{Delta}$= GMT of nAb against Delta variant on D28 of SCTV01E in Cohort 2;

$GMTM2_{Delta}$= GMT of nAb against Delta variant on D28 of mRNA-1273 in Cohort 2.

$GMTC2_{Omicron}$= GMT of nAb against Omicron variant on D28 of SCTV01C in Cohort 2;

$GMTE2_{Omicron}$= GMT of nAb against Omicron variant on D28 of SCTV01E in Cohort 2;

$GMTm2_{Omicron}$= GMT of nAb against Omicron variant on D28 of mRNA-1273 in Cohort 2.

For the primary efficacy objectives, the null hypotheses are:

- **H11**: GMR11=$GMTC1_{Delta}/GMTS1_{Delta} \leq 1$;
- **H12**: GMR12= $GMTE1_{Delta}/GMTS1_{Delta} \leq 1$;
- **H13**: GMR13=$GMTC1_{Omicron}/GMTS1_{Omicron} \leq 1$;
- **H14**: GMR14=$GMTE1_{Omicron}/GMTS1_{Omicron} \leq 1$;
- **H21**: GMR21= $GMTE2_{Delta}/GMTM2_{Delta} \leq 0.67$;
- **H22**: GMR22=$GMTE2_{Omicron}/GMTM2_{Omicron} \leq 0.67$;
- **H23**: GMR23= $GMTC2_{Delta}/GMTM2_{Delta} \leq 0.67$;
- **H24**: GMR24=$GMTC2_{Omicron}/GMTM2_{Omicron} \leq 0.67$;

The estimand framework of primary efficacy objectives is listed in Table A.

Table A Estimand framework of primary objectives

Population	Population aged ≥18 years previously vaccinated with either inactivated or mRNA COVID-19 vaccine or previously diagnosed with COVID-19
Treatment conditions	Test: SCTV01C, SCTVO1E<br>Control: Sinopharm inactivated COVID-19 vaccine, mRNA-1273
Variables	Neutralizing antibody titers against Delta or Omicron variant on D28 after first vaccination
Intercurrent event 1	COVID-19 infection up to D28 after first vaccination. A principal stratum strategy will be used, the

		participants who is infection of COVID-19 up to D28 after first vaccination are excluded from this estimand.
	Intercurrent event 2	Receiving of other drugs or vaccines which will modify the immunity against Delta or Omicron variant without COVID-19 infection up to D28 after first vaccination. A principal stratum strategy will be used, the participants who receive other drugs or vaccines which will modify the immunity against Delta or Omicron variant without COVID-19 infection up to D28 after first vaccination are excluded from this estimand.
	Population-level summary	Ratio of geometric means of the neutralizing antibody titers

Multiplicity:

The sequential approach will be applied for the multiple testing, as shown in Figure B, H11 will firstly be tested with a one-sided type I error of 0.025, H1i, i=2,…,4, H2i,i=1,…,4 will then be sequentially tested with a one-sided type I error of 0.025, i.e., H1i, i=2,…,4, H2i, i=1,…,4 will not be tested unless the previous one has been rejected. The sequential testing method is a closed testing procedure and the family-wise error rate can be controlled.

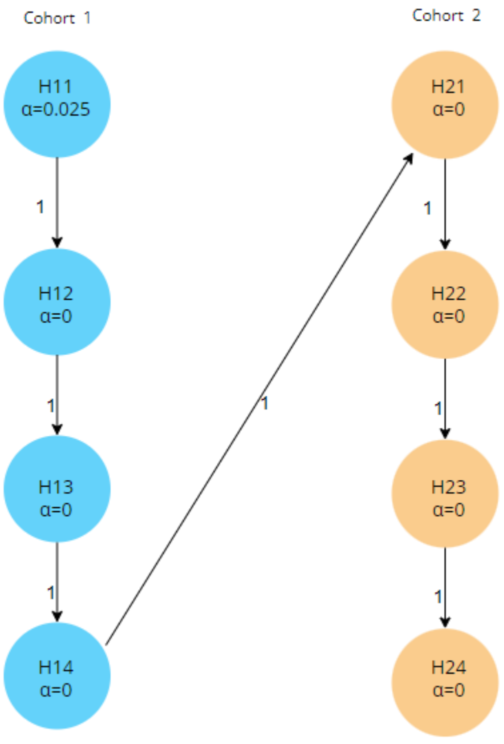

Figure C Sequential testing

Sample size calculation:

Totally 1800 participants aged ≥18 years who were previously vaccinated with either inactivated or mRNA COVID-19 vaccine or previously diagnosed with COVID-19 will be enrolled. 300 participants (100 in SCTV01C Group, 100 in SCTV01E Group, 100 in Sinopharm COVID-19 vaccine Group) in subgroup 1 in Cohort 1 will have nAb tests. 450 participants (150 in SCTV01C Group, 150 in SCTV01E Group, 150 in mRNA-1273 Group) in Cohort 2 will have nAb tests.

Assumptions for the sample size calculation of **H1i**, i=1,…,4, **H2i**,i=1,…,4 are listed as follows:

- The standard deviation of neutralizing antibody titers under log10 transformation is 0.4;
- The 1-sided type I error is 0.025
- Power is about 90%;
- GMR1i≥1.6, i=1,…,4; GMR2i≥1, i=1,…,4;
- The dropout rate of Cohort 1 during study is about 10%;

	• The rate of participants with SARS-CoV-2 infection history and rate of dropout in Cohort 2 is about 20%.<br><br>**Statistical populations:**<br>Full Analysis Set (FAS): All randomized participants who received one dose of investigational product (IP).<br>Per-Protocol Set (PPS): All participants in the FAS set who received planned doses of IP per schedule and have no major protocol deviations, as determined and documented by Sponsor prior to DBL and unblinding, that impact critical or key study data.<br>Safety Set (SS): All randomized participants who received one dose of IP.<br>Immunogenicity full analysis set (I-FAS): All participants in the FAS who had a valid immunogenicity test result prior to receiving the IP and at least 1 valid result after receiving the IP.<br>Immunogenicity per-protocol set (I-PPS): All participants in the PPS who had a valid immunogenicity test result prior to receiving the IP and at least 1 valid result after receiving the IP.<br>Immunogenicity full analysis set (I-FAS1): All participants in the I-FAS who were previously fully vaccinated with 2 doses of Sinopharm inactivated COVID-19 vaccine and with no previous COVID-19 infection history.<br>Immunogenicity per-protocol set (I-PPS1): All participants in the I-PPS who were previously fully vaccinated with 2 doses of Sinopharm inactivated COVID-19 vaccine and with no previous COVID-19 infection history.<br>Immunogenicity full analysis set (I-FAS2): All participants in the I-FAS who previously received mRNA-1273 or Comirnaty and with no previous COVID-19 infection history.<br>Immunogenicity per-protocol set (I-PPS2): All participants in the I-PPS

	who previously received mRNA-1273 or Comirnaty and with no previous COVID-19 infection history.<br>**Statistical analysis methods:**<br>Once the safety data within 28 days and immunogenicity data on D28+3 for each cohort were acquired, it will be analyzed by unblinded team who are independent to the study operation team and are not directly involved in the study activities. The result will be further used for submission to regulatory authority. The specific analysis time point may be adjusted according to the progress of the trial.<br>**General principles**<br>The statistical analysis is carried out with the descriptive and pre-specified statistical test method. The analytical procedures will be detailed in the statistical analysis plan (SAP).<br>American SAS 9.4 or above will be used for statistical analysis.<br>Descriptive statistics of continuous variables will include mean, standard deviation, median, minimum, and maximum values. The classification variable will be described by number and percentage. The calculation method of percentage will be defined in the SAP.<br>The expected values, standard errors, and 95% confidence interval (Cl) will be calculated based on the assumed distribution and pre-specified models, as defined in the SAP.<br>**The Demographic and Baseline Characteristics**<br>The Demographic and Baseline Characteristics, including protocol deviations will be listed.<br>Demographic data and baseline indicators will be analyzed among the FAS. All demographic data (age, sex, race, ethnicity, et al) and baseline variables (physical examination, pregnancy test, history of diseases, history of COVID-19, medication history, interval between time of administration of

	IP and the last time of COVID-19 vaccine administration/diagnosed with COVID-19, the type of previous COVID-19 vaccinations and serum antibody titer before the administration of IP) are summarized.<br>For continuous variables, descriptive statistics (the number of participants, mean, standard deviation, minimum, median and maximum values) are used; and for classified variables, the number and percentage are calculated.<br>**Study treatment exposure and compliance**<br>The exposure dose and trial compliance are descriptively summarized, including safety evaluation and immunogenicity-testing compliance.<br>**Immunogenicity and exploratory analysis**<br>The Immunogenicity analysis will be based on I-FAS, I-PPS, I-FAS1, I-PPS1, I-FAS2, I-PPS2. The main analysis will be based on I-FAS1 and I-FAS2.<br>The GMT of neutralizing antibody for each group with corresponding 2-sided 95% CI will be estimated at each post-baseline time point using an analysis of covariance. The comparison of GMT of neutralizing antibody between the treatment groups at each post-baseline time point will also be provided using an analysis of covariance.<br>The 95% CI of seroresponse using the Clopper-Pearson method will be provided. Cochran-Mantel-Haenszel method will be used for comparison of the seroresponse between the treatment groups.<br>The change in the number of IFN-γ positive (characterizing Th1) and IL-4 positive (characterizing Th2) T cell subsets at each post-baseline time point will be statistically described, and the nonparametric test will be applied for the statistical comparison between groups. Detailed statistical analysis methods are described in the SAP for further reference.<br>**Safety Analysis**

	Safety analysis will be based on SS.<br>AEs and SAEs are encoded based on the *Medical Dictionary for Regulatory Activities* (MedDRA) and also based on the document the classified statistics was made according to the system organ class (SOC) and preferred term (PT). In this trial, the treatment emergent adverse events (TEAEs) are summarized, and the adverse medical conditions occurring before the study vaccination are listed. Unless otherwise specified, the adverse events as described below are TEAEs.<br>The incidence of AEs, SAEs and AESIs, the number and percentage of participants with AEs, SAEs and AESIs in each group will be summarized respectively. The 2-sided 95% CI will be also provided for the percentage of participants with any solicited AE for each treatment group using the Clopper-Pearson method. The adverse events related to the study vaccine, SAEs and AESIs will be listed.
**Independent Data and Safety Monitoring Board**	The sponsor will establish the DSMB to review the safety data of the clinical trials. The DSMB members include experts in the field of vaccine clinical trials, biostatisticians and epidemiologists. See the "DSMB charter" for details of its working documents.

Table 1 Schedule of Activities

	Screening period	Vaccination	Follow-up period		
**Visit**	**V1**	**V2**	**V3**	**V4**	**V5**
**Planned visit date**	**D-14～D0**	**D0**	**D7**	**D28**	**D180 (EOS)**
**Visit window period**	/	/	**+2 days**	**+3 day**	**±7 days**
**Management and general procedures**					
Signing the informed consent form	●				
Confirm participant meets inclusion and exclusion criteria[1]	●	●★			
Demographic data[2]	●				
Recording the medical history[3]	●				
Assigning the screening number	●				
Physical examination[4]	●				
Vital signs[5]	●	●★			
Nasal/pharyngeal/throat swab nucleic acid test[6]	●				
HIV testing	●				
Urine pregnancy test (for women of childbearing potential only)[7]	●	●★			
Randomization		●*			
Vaccination		●			
Viral sequencing			●**		
**Immunogenicity follow-up visit**					
Neutralizing antibodies test for Delta and Omicron variants[8]		●		●	●
Cellular immune response[9]		●		●	
**Safety follow-up visit**					
Solicited AEs[10]		Record solicited AE			
Unsolicited AEs[10]		Record unsolicited AE			
AEs, SAEs and AESIs[11]		●	●	●	●
Observing for at least 30 minutes after the vaccination		●			
Distributing the vaccination record cards (VRCs)		●			
Reviewing and recovering the VRCs			●	●	
Distributing the thermometer		●			
Recording the concomitant medication		●	●	●	●#

Comments:

★: If screening and vaccination are on the same day (D0), there is no need to repeat the items corresponding to '●★' before vaccination.

#: 28 days after the study vaccination, only the concomitant medication used to treat AEs, SAEs and AESIs should be recorded.

*: Participants are randomized after all the tests have been done and before the vaccination on the vaccination day.

**: If the SARS-CoV-2 infection is confirmed after 14 days of the study vaccination, virus will be isolated

from the nasal/nasopharyngeal/throat swab and viral sequencing will be used to identify the major SARS-CoV-2 variants.

1. Inclusion/exclusion should be reviewed during the screening period and on the day of vaccination.
2. Demographic data: including age, sex, race, ethnicity, occupation (working and living circumstances), height, body weight, BMI (derived from height and body weight). The participants should also provide contact information like current phone number and/or E-mail. In subsequent follow-up visits, if the contact information is changed, it should be updated accordingly (if applicable).
3. Records of medical history: including the history of SARS-COV-2 vaccination, history of COVID-19, other vaccinations within 90 days, medication use within 28 days, major surgery, allergic history, and other known significant diseases.
4. Physical examinations: general conditions, head & neck, lymph node, skin, chest, abdomen, musculoskeletal system and other examinations necessary for the study.
5. Vital signs: blood pressure, respiration rate, pulse rate, and body temperature.
6. Nasal/pharyngeal/throat swab nucleic acid test: The result of nucleic acid test for SARS-CoV-2 within 7 days prior to vaccination (including on D0) shall be obtained. If there is no available nucleic acid test report, the patient shall be tested and negative result of nucleic acid test for SARS-CoV-2 should be obtained before vaccination.
7. Urine pregnancy test (for women of childbearing potential only): the urine pregnancy test may be performed routinely, while the blood pregnancy test may be performed if the investigator deems it necessary. A woman is considered of childbearing potential (WOCBP), i.e. fertile, following menarche and until becoming post-menopausal unless permanently sterile. Permanent sterilization methods include hysterectomy, bilateral salpingectomy and bilateral oophorectomy. A postmenopausal state is defined as no menses for 12 months without an alternative medical cause.
8. Neutralizing antibodies test for Delta and Omicron variants: only applied to participants of Subgroup 1 in Cohort 1 and all participants of Cohort 2.
9. Cellular immune response (only applied to the cellular immune response subgroup): the number of IFN-γ positive (characterizing Th1) and IL-4 positive (characterizing Th2) T cell subsets before and 28 days after each study vaccination.
10. Solicited and unsolicited AEs: collect solicited AEs from D0 to D7; and unsolicited AEs from D0 to D28.
11. AEs, SAEs, and AESIs: AEs, SAEs, and AESIs will be collected on visit day or reported by participants actively at any time. If participants cannot come to site on visit day, phone call, short message, email or other contacting method will be used for safety follow-up.

Table of Contents

PROTOCOL SYNOPSIS 1

TABLE OF CONTENTS 24

LIST OF TABLES 26

LIST OF FIGURES 27

ABBREVIATIONS 28

1 STUDY BACKGROUND 31

1.1 BACKGROUND 31
1.2 ETIOLOGY FEATURES 32
1.3 INTRODUCTION OF STUDY VACCINE 33
1.4 SUMMARY OF PRECLINICAL STUDY 35
1.5 CLINICAL SAFETY OF SCTV01C 39
1.6 STUDY SIGNIFICANCE 39
1.7 ASSESSMENT OF BENEFIT/RISK 40

2 STUDY OBJECTIVES AND ENDPOINT 42

2.1 STUDY OBJECTIVES 42
2.2 STUDY ENDPOINT 42

3 STUDY DESIGN 43

3.1 STUDY DESIGN 44
3.2 BLINDING AND UNBLINDING METHODS 47
3.3 DEFINITION OF END OF STUDY 48

4 STUDY POPULATION 48

4.1 INCLUSION CRITERIA 48
4.2 EXCLUSION CRITERIA 49

5 DISCONTINUATION OF STUDY INTERVENTION 51

5.1 WITHDRAWAL CRITERIA 51
5.2 STUDY SUSPENSION/TERMINATION CRITERIA 52

6 DESCRIPTION OF STUDY PROCEDURES AND VISITS 52

6.1 V1 (SCREENING, D-14~D0) 52
6.2 V2 (D0, VACCINATION) 53
6.3 V3 (D7+2D) 54
6.4 V4 (D28+3D) 54
6.5 V5/EOS (D180±7D) 55
6.6 UNPLANNED CONTACT AND FOLLOW-UP 55

7 STUDY VACCINES 55

7.1 BASIC INFORMATION OF VACCINES 55
7.2 STUDY VACCINE MANAGEMENT 56
7.3 VACCINATION ROUTE AND DOSE 57
7.4 VACCINATION PROCEDURE 57
7.5 CONCOMITANT MEDICATION 57
7.6 PARTICIPANT COMPLIANCE 58

8 COLLECTION, PROCESSING AND TESTING OF BIOLOGICAL SAMPLES 58

8.1 COLLECTION, PROCESSING AND TESTING OF IMMUNOGENICITY SAMPLES 58
8.2 COLLECTION, PROCESSING AND TESTING OF VIRUS TEST SAMPLES 59

9 STUDY EVALUATIONS AND REPORTS 60

9.1 SAFETY ASSESSMENT 60
9.2 IMMUNOGENICITY ASSESSMENT 69

9.3 DIAGNOSIS AND TREATMENT OF COVID-19 INFECTION 70
10 DATA MANAGEMENT 73
10.1 SOURCE DATA AND THE FILLING AND TRANSFER OF ECRF 73
10.2 DATABASE PROPOSAL AND DESIGN 73
10.3 ENTRY OF DATA 74
10.4 MEDICAL CODING 74
10.5 DATABASE LOCKING AND EXPORTING 74
10.6 ARCHIVE OF STUDY RECORDS 74
11 STATISTICAL ANALYSIS 75
11.1 HYPOTHESIS 75
11.2 MULTIPLICITY 76
11.3 SAMPLE SIZE ESTIMATION 77
11.4 STATISTICAL POPULATIONS 77
11.5 STATISTICAL ANALYSIS METHOD 78
12 MANAGEMENT OF CLINICAL TRIAL 80
12.1 DECLARATION 80
12.2 ETHICS 80
12.3 INFORMED CONSENT 80
12.4 REVISION OF CLINICAL TRIAL PROTOCOL 81
12.5 PROTOCOL DEVIATION 82
12.6 MONITORING 82
12.7 QUALITY ASSURANCE AND AUDIT 83
12.8 INTELLECTUAL PROPERTY 83
12.9 PARTICIPANTS' PRIVACY 83
12.10 MONITORING BOARD 83
13 FINANCE AND INSURANCE 84
14 PUBLISHING AND DATA SHARING POLICIES 85
15 APPENDICES 86
15.1 APPENDIX I: TOXICITY RATING SCALE FOR HEALTHY ADULT AND ADOLESCENT VOLUNTEERS IN PREVENTIVE VACCINE CLINICAL TRIAL - FDA STANDARD 86
16 REFERENCES 90

List of Tables

Table 1 Schedule of Activities 22
Table 2 List of solicited AE 61
Table 3 List of Potential Immune-mediated Diseases to be Collected in the Context of Vaccines 62
Table 4 List of Adverse Events of Special Interest Applicable to COVID-19 vaccines (Guidance Document from SPEAC) 63
Table 5 List of Adverse Events of Special Interest Relevant to COVID-19 (Guidance Document from SPEAC) 63
Table 6 General principles for the grading of adverse events 64
Table 7 COVID-19 disease severity 71
Table 8 Estimand framework of primary objectives 76
Table 9 Scale of clinical abnormalities 86
Table 10 Scale of vital signs 86
Table 11 Scale of adverse events 87
Table 12 Abnormal Results of Laboratory Examinations 87
Table 13 Hematological Abnormality 88
Table 14 Abnormalities of Urine Routine Examination 89

List of Figures

Figure 1 Study design for Cohort 1 46
Figure 2 Study design for Cohort 2 46
Figure 3 Diagnosis process of COVID-19 cases 72
Figure 4 Sequential testing 77

ABBREVIATIONS

Acronym or terminology	Explanation
ACE-2	Angiotensin converting enzyme 2
ADE	Antibody-mediated infection enhancement
AE	Adverse event
AESI	Adverse event of special interest
ALT	Alanine aminotransferase
AST	Aspartate aminotransferase
AR	Adverse reaction
BUN	Blood Urea Nitrogen
CDISC	Clinical Data Interchange Standards Consortium
CI	Confidence interval
COVID-19	Novel coronavirus pneumonia
CRO	Contract Research Organization
DBL	Database Lock
DM	Data manager
DMP	Data management plan
DSMB	Data and Safety Monitoring Board
eCRF	Electronic case report form
EDC	Electronic data capture
ERC	Ethics Committee
FAS	Full analysis set
GBS	Guillain-Barre Syndrome
GCP	Good Clinical Practice
GMT	Geometric mean titers
H_0	Null hypothesis
HIV	Human immunodeficiency virus
HR	Hazard ratio
IB	Investigator's brochure
ICF	Informed consent form
ICH	International Conference on Harmonization of Technical Requirements
I-FAS	Immunogenicity of full analysis set
IFN-γ	Interferon-γ
IL	Interleukin

Acronym or terminology	Explanation
I-PPS	Immunogenicity Per-Protocol Set
IRB	Institutional Review Board
IP	Investigational product
ITT	Intent-to-treat
IWRS	Interactive web response system
MedDRA	Medical Dictionary for Regulatory Activities
MERS	Middle East respiratory syndrome
mITT	Modified intent-to-treat set
MTD	Maximum tolerated dose
mRNA	Messenger ribonucleic acid
NMPA	National Medical Products Administration
nAb	Neutralizing antibody
PCR	Polymerase chain reaction
PPS	Per-Protocol Set
PT	Preferred term
QC	Quality control
RBD	Receptor-binding domain
SAE	Severe adverse event
SAP	Statistical analysis plan
SARS	Severe acute respiratory syndrome
SAS	Statistical analysis system
SDV	Source data verification
SARS-CoV-2	Novel coronavirus
S-ECD	Extracellular region of recombinant spike protein of novel coronavirus mutant strain
SOC	Systematic organ classification
SOP	Standard operating procedure
SpO_2	Oxygen saturation
SS	Safety analysis set
SUSAR	Suspected and unexpected severe adverse reaction
TEAE	Treatment emergent adverse events
Th1	Helper T cell 1
Th2	Helper T cell 2
TNF-α	Tumor necrosis factor-α

**Acronym or terminology**	**Explanation**
RBC	Red blood cell count
RdRp	Ribonucleic acid polymerase
RNA	Ribonucleic acid
RR	Respiratory rate
RT-PCR	Reverse polymerase chain reaction
URL	Uniform resource locater
US FDA	United States Food and Drug Administration
VE	Vaccine efficacy
VED	Vaccine-enhanced disease
VRC	Vaccination record card
VOC	Variants of Concern
WHO	World Health Organization
WHO DD	Dictionary of Drugs of World Health Organization
2019-nCoV	Novel coronavirus

1 STUDY BACKGROUND

1.1 BACKGROUND

Novel coronavirus pneumonia (COVID-19) is a newly emerging acute respiratory infection caused by the novel coronavirus (2019-nCOV or SARS-CoV-2) infection. COVID-19, first detected in humans in December 2019, has quickly spread to more than 210 countries and regions worldwide. World Health Organization (WHO) declared the COVID-19 outbreak a public health emergency of international concern on January 30, 2020, followed by the declaration that the disease has pandemic characteristics on March 11, 2020. As of December 24, 2021, WHO reported a total of 278,551,962 confirmed cases, including 5,401,376 deaths. The outbreak and epidemic of COVID-19 pose a serious threat to human health and survival. At present, vaccine becomes the most effective means to prevent virus infection. Several COVID-19 vaccines have been approved for conditional marketing or emergency use, and COVID-19 vaccines have been inoculated in large scale worldwide[1].

SARS-CoV-2 is an RNA single-stranded virus that is prone to deletion mutation, which occurs mostly in recurrent deletion regions (RDRs) of the S protein. Deletion or mutation may change the conformation of S protein, resulting in the decrease of vaccine immune effect and virus immune escape. Although the early D614G mutation (B.1) that enhances the binding of the S protein to the ACE2 receptor does not reduce sensitivity to neutralizing antibodies, with the pandemic of SARS-CoV-2, several high-risk mutant strains have emerged worldwide: Alpha (B.1.1.7), Beta (B.1.351), Gamma (P.1), Delta (B.1.617.2) and Omicron (B.1.1.529). Studies have shown that these high-risk strains increase the transmission of the virus, aggravate disease development (increase hospitalization or mortality), reduce the COVID-19 immunity induced by previous infections or immunizations, reduce the efficacy of treatments or vaccines, and invalidate diagnostic tests. The Delta variant, first detected in India in December 2020, now accounts for most of the coronavirus infections in many regions. However, the newly emerging Omicron (B.1.1.529) variant, which was first reported to WHO from South Africa on November 24 2021, and was designated as a VOC on November 26 2021, spreads much faster than Delta variant. The Omicron variant has turn to be the dominant variant in many provinces across the world. The Omicron variant has at least 50 mutations, 30 of which are on the S protein, most of which are located in the domains that interacts with hACE2. The Omicron variant comprises of mutations discovered in the Delta variant that are considered to increase transmissibility and mutations discovered in the Beta and Delta variants that are believed to promote immune escape. Preliminary evidence suggests an increased risk of reinfection with

this variant, as compared to other VOCs. The number of cases of this variant appears to be increasing in almost all provinces in South Africa. Omicron has spread to various countries in Europe, including Belgium, the Netherlands, France, the UK, Australia and Canada within a short time. Given the adverse effects and high transmissibility of Omicron and Delta, there is an urgent need for new generation of vaccines with high protective efficacy against high-risk variants.

Original-strain based 1st generation vaccine provides insufficient protection to variants with decreased vaccine-efficacy. Omicron can escape first generation vaccine, and a boost dose only provides likely less than 3-6 month protection resulting vaccinated population essentially naïve to Omicron. 3 doses of Omicron vaccine are needed to induce long-lasting immunity.

SCTV01C is the first generation vaccine containing two antigens TM22 and TM23 while SCTV01E containing four antigens TM22, TM23, TM28 and TM41. Compared with single antigen with sequence from one variant, multiple antigens with sequences from different variants can induce board neutralizing antibodies spectrum against multiple variants.

SCTV01E is specifically designed for the prevention of infection with SARS-CoV-2 and its variants. An affordable and thermal stable 2nd generation COVID-19 vaccine providing broad immunity to existing and emerging variants.

1.2 ETIOLOGY FEATURES

SARS-CoV-2 belongs to β genus coronavirus and has an envelope with round or elliptic particles in diameter of 60~140 nm. It has five essential genes, which target four structural proteins, i.e., nucleoprotein (N), virus envelope (E), matrix protein (M) and spike protein (S), and ribonucleic acid (RNA) dependent RNA polymerase (RdRp). The nucleoprotein (N) wraps the RNA genome to form the nucleocapsid, which is surrounded by a viral envelope (E), in which the matrix protein (M) and spike protein (S) are embedded. The spike protein enters the cell by binding with angiotensin converting enzyme 2 (ACE-2). With isolation and co-culture test *in vitro*, novel coronavirus can be detected in human respiratory tract epithelial cells in about 96 hours and in Vero E6 and Huh-7 cell lines in about 4 to 6 days[2].

Coronaviruses are sensitive to ultraviolet, heat, and can be effectively inactivated by ethyl ether, 75% ethanol, chlorine-containing disinfectant, peracetic acid and chloroform at 56°C for 30 minutes, except for chlorhexidine.

1.2.1 CLINICAL MANIFESTATION

The most common symptom of COVID-19 is fever, dry cough and fatigue, while some patients

experience smell and taste disorder as the initial symptom. A small portion of patients also have nasal congestion, runny nose, throat pain, conjunctivitis, myalgia and diarrhea and other symptoms. Severe patients usually develop dyspnea and/or hypoxemia one week after the onset, and more severe patients may rapidly progress to acute respiratory distress syndrome, septic shock, refractory metabolic acidosis, coagulation dysfunction and failure of multiple organs. Very few patients may also have central nervous system involvement and avascular necrosis of the extremum. Severe and critical patients may have moderate to low fever or even no fever during the COVID-19 infection. Mild patients may show low fever, mild weakness, smell, and taste disorders, but no pneumonia symptom, and a small number of patients with the COVID-19 infection can have no obvious clinical symptoms. While COVID-19 infection generally have a mild course in most patients, a number of pre-existing co-morbidities determine the severity of infection and the outcome in the patients, and the critical conditions are mostly seen in elderly with chronic underlying diseases, women in the third trimester and perinatal period and the obese population[2].

1.2.2 INFECTION SOURCE AND ROUTE OF TRANSMISSION

Patients infected with novel coronavirus and asymptomatic infected persons are the main source of spreading. They are contagious during the incubation period and are highly contagious within 5 days after the onset of the disease. Transmission is mainly through respiratory droplets and close contact. Infection can also be caused by contact with objects contaminated with the virus. As there is a possibility of aerosol transmission in a relatively closed environment when exposed to high concentration of aerosols for a long time and novel coronavirus can be isolated from feces and urine, attention should be paid to its exposure to environmental pollution or aerosol transmission.

1.3 INTRODUCTION OF STUDY VACCINE

1.3.1 INTRODUCTION OF SCTV01C

The bivalent SARS-CoV-2 trimeric spike protein vaccine (code name SCTV01C) is a recombinant protein vaccine developed by Sinocelltech Ltd., with genetic engineering technology adopted to express in CHO cells. SCTV01C is SARS-CoV-2 bivalent recombinant trimeric subunit protein with oil-in-water adjuvant suspension, containing TM22 and TM23 in three dose levels of 10/20/30μg with 0.5 mL/vial[3].

The S protein of COVID 19 is a key antigen in the design of COVID-19 vaccine. The S protein binds to the host cell's ACE-2 receptor via the receptor-binding domain and is cleaved by the host protease into S1 polypeptide containing the receptor-binding domain (RBD) and S2

polypeptide mediating the virus's fusion with the cell membrane, stimulating B cells to produce high titer neutralizing antibodies against RBD and abundant T cell epitopes that can induce specific CTL reaction in T cells. SCTV01C trimeric subunit protein consists of the ECD region of S protein and T4-foldon. SCTV01C, simulating S protein, has complete biological function and natural structural properties, which ensures the high proportion of neutralizing epitope of correct structure and the induction of neutralizing antibodies with high titers. At the same time, SCTV01C has more T cell epitopes and can induce stronger resistance to variants infection than the RBD protein vaccines due to 5 time larger in its molecular weight.

The adjuvant (SCT-VA02B) is an oil-in-water adjuvant, which its composition, the formula, process and main quality control (particle size) are consistent with the commercial marketable MF59 adjuvant. MF59 adjuvant can induce helper T cell 1 (Th1) response in human body. Its immune response effect is better than that of aluminum adjuvant, which mainly induces helper T cell 2(Th2) response.

1.3.2 INTRODUCTION OF SCTV01E

The quadrivalent SARS-CoV-2 trimeric spike protein vaccine (code name SCTV01E) is a recombinant protein vaccine developed by Sinocelltech Ltd., with genetic engineering technology adopted to express in CHO cells.

SCTV01E contains the mixture of TM22, TM23, TM28 and TM41 recombinant proteins combined with SCT-VA02B adjuvant, formulated with sodium citrate, sodium chloride and other excipients, without preservatives and antibiotics. The adjuvant (SCT-VA02B (1×)) is an oil-in-water adjuvant, whose composition, formula, process and main quality control (such as particle size) are consistent with those of the commercial marked MF59 adjuvant which was introduced in vaccine since 1997. Adjuvant like MF59® can induce helper T cell 1 (Th1) response in human body. Its immune response effect is better than that of aluminum-based adjuvant, which mainly induces helper T cell 2(Th2) response. The TM22, TM23, TM28 and TM41 antigens used in SCTV01E are the recombinant Spike-ECD trimeric proteins produced in classic CHO platform, which amino acid sequences are from Alpha, Beta, Delta and Omicron variants.

SCTV01E has identical trimeric protein molecular design with SCTV01C. Using the same CHO cell production platform technology, SCTV01E has highly similar quality (high purity, low impurity), and all four variants share high sequence homology. SCTV01E's immunogenicity is similar to SCTV01C in terms of total IgG titer so is not expected to have different safety profile. Based on the excellent nonclinical safety of SCTV01C, the similarity

among the four antigens and the lower clinical dose, SCTV01E is equivalent to SCTV01C in terms of vaccine safety and no safety risk is expected for SCTV01E. SCTV01E dose will not exceed the high dose of SCTV01C tested in Phase I.

SCTV01E is specifically designed for the prevention of infection with SARS-CoV-2 and its variants. SCTV01E make a difference to the Delta and Omicron variants due to higher NAT50 titer: SCTV01E trimeric subunit protein consists of the ECD region of S protein and T4-foldon. SCTV01E, simulating S protein, has complete biological function and natural structural properties, which ensures the high proportion of neutralizing epitope of correct structure and the induction of neutralizing antibodies with high titers. At the same time, SCTV01E has more T cell epitopes and can induce stronger resistance to variants infection than the RBD protein vaccines due to 5 time larger in its molecular weight.

1.4 SUMMARY OF PRECLINICAL STUDY

1.4.1 NON-CLINICAL PHARMACOLOGY

1.4.1.1 In Vitro Efficacy Study

The structural frame of all SCTV01C and SCTV01E trimeric protein consists of the extracellular domain region of S protein (S-ECD, including S1 and S2 part) and T4-foldon.

S-ECD trimer protein removes the Furin cleavage site between S1 and S2 to solve the stability problem caused by the cleavage. The S-ECD is made with a fusion with T4-foldon (C-terminal of phage fibrin) to promote the formation and stability of trimer. SCTV01C and SCTV01E trimer proteins, retaining of their natural structural properties, having complete biological functions and correct conformational characteristics, can produce effective neutralizing antibodies with multiple epitopes, which are featured with the protein structural basis of protection against virus infection[3].

1.4.1.2 In Vivo Efficacy Study

Immunogenicity Study

Immunogenicity study shows that SCTV01C and SCTV01E can induce a high level of humoral immune response in female C57BL/6J mice, and the neutralization activity against antigen related/unrelated strains of COVID19 and Th1/Th2 immune response induced are better than monovalent vaccines. The humoral immune response and Th1/Th2 immune response induced by the combination of SCTV01C or SCTV01E with adjuvant SCT-VA02B are significantly better than the control adjuvant.

SCTV01C and SCTV01E shows high level of the humoral immune response in female SD rats (200-220g) in the dose range of 20μg ~40μg. The neutralizing antibodies titers are increase

following with the increase of the level of adjuvant. When testing at 40μg dose level, the highest immunogenicity is detected in the high-dose adjuvant group (10 mg/rat), and the non-adjuvant group shows the lowest immunogenicity. The specific total IgG titers of SCTV01C and SCTV01E showed the same level in rats 1 week after second administration of equal total antigen dose in the dose range of 20μg ~ 40μg.

In addition, SCTV01C-TM23 and SCTV01E-TM23 shows strong humoral immune response and T cell response in cynomolgus monkeys.

Virus-Challenge and Attacking Protection Efficacy in Mice

As a demonstration of feasibility and working mechanism, a virus-challenge study was conducted with the bivalent vaccine (covering Alpha and Beta variants). The load of hACE2-KI/NIFDC murine pneumonia virus was reduced by $10^{5.32}$ times in the bivalent vaccine group (SCTV01C, the first generation of vaccine), no viral RNA was observed in the lung tissue, and no antibody mediated infection enhancement (ADE) and vaccine-enhanced disease (VED) were observed. The results showed that the bivalent vaccine had a clear protective effect against South African variant of novel coronavirus. The results also showed that SCTV01C-TM23 monovalent vaccine had a clear protective effect against South African (Beta) variant, and had a cross-protection effect on Beijing prime strain infection.

1.4.2 TOXICOLOGY

The active components of SCTV01E contain four recombinant proteins, named TM22 (Alpha), TM23 (Beta), TM28 (Delta) and TM41 (Omicron) proteins, which are homotrimeric proteins with a few mutated residues difference on RBD region for TM22, TM23 and TM28, and some mutant sites difference on the spike protein between TM41 and other antigens. To accelerate the speed of development, by referencing the concept in EMA guidelines "Reflection paper on the regulatory requirements for vaccines intended to provide protection against variant strains of SARS-CoV-2" and "Guideline on Influenza Vaccines Non-clinical and Clinical Module", nonclinical testing to support the development of variant vaccines are not required if comprehensive studies have been done and accepted for the parent vaccine. Therefore, SCTV01C had been developed in advance to estimate the safety of SCTV01E. Based on systematic safety evaluation results of SCTV01C, decreased target dose of SCTV01E in human, and sufficient immunogenicity study data in animals, SCTV01E had be estimated on the safety risk which is expected to be low. The toxicology evaluation of SCTV01E will be conducted in January 2022 and completed in April 2022.

1.4.2.1 Single Dose Toxicity Test of SCTV01C

To demonstrate the acute toxicity, the single dose toxicity study was done with the bivalent

vaccine at high dose as representative model (the worst case scenario) in two studies. In the first single dose toxicity test, SD rats were observed for 14 days after single intramuscular injection of SCTV01C with TM23 produced from the stable pool-cell which was available earlier, and in the second single dose toxicity test, the study was repeated with SCTV01C with TM23 produced from the stable clonal-cell. There were no mortality or morbidity observed, no abnormal reaction observed in clinical observation and necropsy in both studies. The study results show no acute toxicity, and the maximum tolerated dose (MTD) in rats is ≥4 doses/rat (240 μg/rat).

1.4.2.2 Repeat Dose Toxicity Test of SCTV01C

To evaluate the long-term toxicity and to accelerate the project development based on the high similarity of the antigens, the repeat dose toxicity study was completed with the bivalent vaccine at high dose as representative model of SCTV01E (the worst case scenario). SCTV01C was intramuscularly administrated to SD rats in 1 and 3 doses/rat once every 2 weeks for consecutive 6 weeks (4 times in total, much larger safety margin supporting 2 human doses), accompanied by a 2-week recovery period. Increase of Neut, Eos, FIB, Glb and Ca, decrease of Retic, CHO, Alb and A/G and increase of spleen weight coefficient could be seen in male and/or female animals in both placebo control group (adjuvant) and low/high-dose groups. Where, the decrease of Retic and CHO was thought to relate to the adjuvant, the increase of Ca was thought to be associated with the test sample, while other indicators were thought to be related to the acute phase reaction and/or immune response caused by administration. The changes, except for Alb, Glb and A/G, have fully disappeared at the end of the recovery period. There was no obvious systemic toxicity associated with administration.

1.4.2.3 Immunogenicity Test of SCTV01C

A robust immune response can be induced in SD rats by repeated intramuscular injection of SCTV01C used as a model for SCTV01E at 1 dose/rat and 3 doses/rat for 6 weeks. The titers of anti-TM22 and anti-TM23 antibodies increased following with the increase of doses, showing a dose correlation, and the geometric mean titers (GMT) of the low and high dose groups reached the peak at Week 2 and Week 1 in recovery period, respectively. The similar trend was observed in neutralizing test in which the titers of neutralizing antibodies against the pseudoviruses of SARS-CoV-2 variants increased as the administration doses went up. GMT of both low and high dose groups reached the peak at Week 1 in recovery period.

1.4.2.4 Immunotoxicity Test of SCTV01C

No immunotoxicity was observed after repeated intramuscular injection with SCTV01C used as the model for SCTV01E at 1 dose/rat and 3 doses/rat in SD rats for 6 weeks (4 times in

total). 3 days after last administration (D46), the decrease of Alb and A/G, the increase of Glb, Neut and Eos, and the increase of spleen weight/organ-to-body weight ratio/organ-to-brain weight ratio were observed, which were thought to be related to the immune response. All of these indicators returned to normal at the end of the 2-week recovery period. There was no abnormality related to drug administration in macroscopic observation of the lymphatic organs/tissues of the animals microscopic observation of thymus, bone marrow and so on. No notable change of peripheral T lymphocyte subsets distribution and the weight of thymus were observed in each group.

1.4.2.5 Local Tolerance and Other Toxicity Studies of SCTV01C

The study in animals, again used SCTV01C as the representative case supporting SCTV01E, shows no notable local reaction associated with the drug administration in clinical observation and necropsy, and the local irritation noted in microscopic examination were related to the adjuvant. The local irritation study of SCTV01C on SD rats showed that no abnormal reactions such as erythema, hyperemia, swelling, ulcer and induration were observed locally in each group and no abnormalities were observed in necropsy during the experiment. The local irritation study in New Zealand rabbits showed that there was no abnormal reaction such as swelling, fever, edema, erythema, hyperemia and ulcer in the local site of the drug administration in each group. The local tolerance was evaluated first with SCTV01C with the TM23 produced in stable pool-cell process combined with repeat-dose toxicity study in rats, and then two batches of SCTV01C with the TM23 produced in stable pool-cell process or stable clonal-cell process were evaluated and compared in a single local tolerance test in rabbits, with consistent results between two batches. The microscopic examination results showed that minimal/slight to moderate mixed inflammation were observed in all animals on the placebo control side, CHO-pool product side and stable CHO clone product side, which were considered to be related to the local irritation induced by the adjuvant placebo. All related abnormal changes were partially disappeared after two weeks recovery.

The sensitization was induced by intramuscular injection of SCTV01C at 0.1 dose/animal and 1 dose/animal and simulated by intravenous injection at 0.2 dose/animal and 2 doses/animal, respectively. No active systemic anaphylaxis was observed in guinea pigs.

Reproductive toxicity test, which have not been conducted yet, will be carried out according to the subsequent clinical trial plan.

According to both the ICH guideline and the *General Principles for Technical Review of Preclinical Safety Evaluation of Biological Products for Prophylaxis* issued by National Medical Products Administration (NMPA) in 2008[4], vaccines generally do not require

genotoxicity tests, carcinogenicity tests, dependence tests and routine pharmacokinetic studies. And hemolysis study is also not applicable for a recombinant protein vaccine which is administrated by intramuscular injection. For SCTV01C or SCTV01E, as the novel coronavirus recombinant protein vaccine, relevant studies have not been done in accordance with the regulatory requirements.

1.5 CLINICAL SAFETY OF SCTV01C

A Phase I/II clinical trial to evaluate immunogenicity and safety of SCTV01C in population unvaccinated with COVID-19 vaccine and aged ≥ 18 years old, has been initiated in China. The first participant was enrolled on December 01, 2021. As of December 23, 2021, 65 participants were enrolled into Phase I (planned 112): 12 (18.4%) of them reported AEs, including solicited local and systemic AEs and unsolicited AEs. The blinded data shows that all of these AEs were Grade 1 or 2. There was no ≥ Grade 3 AE observed, and no SAE or AESI either. The most common adverse reactions were injection site pain (8 participants, 12.3%). Other solicited local AEs reported by participants were induration (1, 1.5%), redness (1, 1.5%) and swelling (1, 1.5%). The solicited systemic AEs reported by participants were fever (1, 1.5%), anxiety (1, 1.5%), fatigue (1, 1.5%), and diarrhea (1, 1.5%).

1.6 STUDY SIGNIFICANCE

The purpose of this study is to evaluate the safety and immunogenicity of SCTV01C and SCTV01E in preventing COVID-19 caused by SARS-COV-2 and its concerning variants (VOC) infection, particularly Delta and Omicron, in populations aged 18 years and older. The outbreak and pandemic of COVID-19 have caused serious impact and challenge to the global health care system and posed a serious threat to human survival and health. The nearly all currently vaccine marketed or EUA approved design is basically based on the sequence of the early epidemic strain (Wuhan strain), while SARS-COV-2 is an RNA single-stranded virus that is prone to deletion mutation. Given with fast spread of high-risk mutant strains such as Delta and Omicron, which had the higher transmissibility of the epidemic, aggravate the development of the disease and severely reduce the protective effect of neutralization of antibodies generated by prior infection or immunization, the rapid development of the second-generation vaccines with high protection against those high-risk variants is of utmost importance.

1.7 ASSESSMENT OF BENEFIT/RISK

1.7.1 RISK ASSESSMENT

1.7.1.1 The Risk of Study Vaccine Administration

While the results of preclinical study of SCTV01C have shown the favorable safety profile and immunogenicity, and from mechanistic perspective, supporting the direct use of SCTV01E, and safety risk of SCTV01E has been estimated to be low, there is no long-term study showing persistence of neutralization antibodies, against any individual variants. Though there are some safety data from the on-going clinical trials with SCTV01C, there are no data available from clinical trials on the use of SCTV01E vaccines in humans at the outset of this study. Meanwhile, as with other vaccines, there is a potential risk of allergic reaction, and this vaccine may not be effective for all vaccinated people.

1.7.1.2 Vaccine-enhanced disease

The mechanism of ADE/VED is unclear, and there are no specific clinical indications or laboratory indicators for clinical diagnosis, but ADE/VED is somewhat associated with non-neutralizing antibodies. ADE effects during Fc receptors cell recognition and internalization of non-neutralizing virus-antibody complex will lead to increased viral ingestion and aggravation of viral infection. VED may occur via ADE, activation of leukocyte differentiation antigen 4 positive (CD4+) memory T cells, Th2 deviation or abnormal T cell, which will enhance the viral transfection and replication. While no ADE/VED due to SCTV01C intervention was shown in the nonclinical findings, risk control measures should be developed in clinic and all participants should be closely monitored for VED and followed up throughout the study.

1.7.1.3 Risk of Biological Sample Collection

Venipuncture is a routine clinical procedure adopted by the medical community to collect blood samples. Immediate complications may include mild pain during skin piercing and uncommon dizziness and syncope. In addition, venipuncture may cause hematoma with low risk. Skin/soft tissue infection may occur in the puncture point, vein or the blood flow but in a low change. The risk associated with the nasal/pharyngeal/throat swab collection process is low. Some people may cough and sneeze briefly during and after the swab, and a few may experience irritation in the nasal passages or may bleed slightly.

Researchers will strictly supervise qualified and experienced medical personnel or health workers trained to collect venous blood samples and nasal/pharyngeal/throat swab in accordance with prescribed procedures to minimize the pain and risk to the participants (including local pain and the low probability of venipuncture site infection and nasal mucosal damage).

1.7.1.4 Exposure during Pregnancy

At present, no reproductive toxicity test has been done. In this study, a contraceptive period is set according to the experience of the marketed recombinant protein vaccine, that is, effective contraceptive measures shall be taken by the participants entering the clinical trial from the signing of informed consent to 6 months after vaccination.

Examples of acceptable forms of highly effective contraception include:

- Combined (estrogen and progestogen containing) hormonal birth control that prevents ovulation (oral, intravaginal, transdermal)
- Progestogen-only hormonal birth control that prevents ovulation (oral, injectable, implantable)
- Intrauterine device or intrauterine hormone-releasing system
- Bilateral tubal occlusion and ligation
- Sterilized sexual partner with documented absence of sperm Abstinence (Avoid sexual intercourse)

During this period, the investigator will keep contact with the participants to determine whether pregnancy and related complications occur.

1.7.2 BENEFIT ASSESSMENT

All participants will undergo a physical examination (including but not limited to routine physical examination, vital signs and SARS-COV-2, etc.) and receive the examination results free of charge.

Participation in this study will result in a better understanding of COVID-19, leading to better prevention measures. If SCTV01C and SCTV01E is successful in preventing COVID-19, it will provide an effective way against the virus and make a significant contribution to global public health progress.

1.7.3 ASSESSMENT OF OVERALL BENEFIT-RISK

Control measures against potential risks such as allergic reaction and ADE/VDE will be developed prior to the start of the clinical trial, and all participants will be closely monitored and followed in strict compliance with inclusion and exclusion criteria to ensure timely management and maximum benefit for participants at risk. The investigators will be trained for participant follow-up and safety data collection to ensure the timely updating of adverse events. The independent Data and Safety Monitoring Board (DSMB) will review the safety data collected throughout the study, conduct periodic or temporary meetings to assess risk and benefit, and make recommendations to sponsors for the safety concerns. In summary, the

anticipated risks associated with SCTV01E are expected to be manageable. Given the great potential of COVID-19 prevention, the profile of this vaccine candidate supports initiation of this Phase III clinical trial.

2 STUDY OBJECTIVES AND ENDPOINT

2.1 STUDY OBJECTIVES

Primary Objective:

- To evaluate the immunogenicity of SCTV01C;
- To evaluate the immunogenicity of SCTV01E;

Secondary Objective:

- To evaluate the cellular immune response of SCTV01C;
- To evaluate the cellular immune response of SCTV01E;
- To evaluate the safety of SCTV01C within 180 days after the vaccination.
- To evaluate the safety of SCTV01E within 180 days after the vaccination.

2.2 STUDY ENDPOINT

Primary endpoints

Cohort 1

Immunogenicity

- GMT of nAb against Delta variant on D28.
- GMT of nAb against Omicron variant on D28.

Cohort 2

Immunogenicity

- GMT of nAb against Delta variant on D28.

Secondary endpoints:

Cohort 1

Immunogenicity

- GMT of nAb against Delta variant on D180.
- GMT of nAb against Omicron variant on D180.
- Number of IFN-γ positive (characterizing Th1) and IL-4 positive (characterizing Th2) T cell subsets on D28.
- Seroresponse of nAb (defined as a change from below the low limit of quantitation [LLOQ] to equal to or above LLOQ, or a ≥4-fold rise if baseline is equal to or above LLOQ in nAb to Delta variant from D0) rates on D28.
- Seroresponse of nAb (defined as a change from below LLOQ to equal to or above

LLOQ, or a ≥4-fold rise if baseline is equal to or above LLOQ in nAb to Omicron variant from D0) rates on D28.

Safety

- Incidence and severity of solicited AEs of SCTV01C from D0 to D7.
- Incidence and severity of all unsolicited AEs of SCTV01C from D0 to D28.
- Incidence and severity of SAEs and AESIs of SCTV01C within 180 days.
- Incidence and severity of solicited AEs of SCTV01E from D0 to D7.
- Incidence and severity of all unsolicited AEs of SCTV01E from D0 to D28.
- Incidence and severity of SAEs and AESIs of SCTV01E within 180 days.

Cohort 2

Immunogenicity

- GMT of nAb against Omicron variant on D28.
- GMT of nAb against Delta variant on D180.
- GMT of nAb against Omicron variant on D180.
- Number of IFN-γ positive (characterizing Th1) and IL-4 positive (characterizing Th2) T cell subsets on D28.
- Seroresponse of nAb (defined as a change from below the low limit of quantitation [LLOQ] to equal to or above LLOQ, or a ≥4-fold rise if baseline is equal to or above LLOQ in nAb to Delta variant from D0) rates on D28.
- Seroresponse of nAb (defined as a change from below LLOQ to equal to or above LLOQ, or a ≥4-fold rise if baseline is equal to or above LLOQ in nAb to Omicron variant from D0) rates on D28.

Safety

- Incidence and severity of solicited AEs of SCTV01C from D0 to D7.
- Incidence and severity of all unsolicited AEs of SCTV01C from D0 to D28.
- Incidence and severity of SAEs and AESIs of SCTV01C within 180 days.
- Incidence and severity of solicited AEs of SCTV01E from D0 to D7.
- Incidence and severity of all unsolicited AEs of SCTV01E from D0 to D28.
- Incidence and severity of SAEs and AESIs of SCTV01E within 180 days.

3 STUDY DESIGN

Many studies have shown that the neutralizing abilities against SARS-CoV-2 variants decreased substantially after a period time from the initial fully vaccination mainly because of decreased antibody titers, which justify the needs of booster dose[5-9]. In terms of the time

interval between the booster dose and the last dose of the initial vaccination, most of the studies chose more than 3 to 6 months as the interval time[10-13]. Considering participants safety and the ongoing SARS-CoV-2 vaccine booster studies, we choose ≥3 months and ≤12 months post the last dose administration of the initial vaccination as one of the inclusion criteria.

3.1 STUDY DESIGN

Although there is no clinical data for SCTV01E so far, SCT had initiated three clinical Phase I/II trials for SCTV01C to evaluate the safety and immunogenicity, which can be instructive and meaningful for SCTV01E clinical study consideration because of the same manufacturing processes, extremely similar molecular characteristics and clinical dosing between SCTV01E and SCTV01C. The SCTV01C trials will provide sufficient supportive safety and immunogenicity clinical data prior to the start of SCTV01E trials. The details of these trials are summarized in investigator's brochure.

SCTV01E, the quadrivalent vaccine, will be tested in this Phase III immunogenicity study based on the clinical data on safety, reactogenicity, and immunogenicity generated with the bivalent vaccine (SCTV01C) similarity in manufacturing process for four TM (trimeric drug substance) components of the quadrivalent product compared to the bivalent product; similarity in construct design supporting a similar safety profile of the quadrivalent product to that of the bivalent vaccine. The dose strength of SCTV01E is 30μg (5/5/5/15μg for TM22/TM23/TM28/TM41)/dose based on the nonclinical study of SCTV01E and SCTV01C in combination with the clinical studies of SCTV01C.

The study is a randomized, double-blind, and positive-controlled Phase III booster study. It will evaluate the immunogenicity and safety of one dose of SCTV01C or SCTV01E as booster compared with either one dose of Sinopharm inactivated COVID-19 vaccine (Cohort 1) or one dose of mRNA-1273 (Cohort 2).

Approximately 1,800 participants aged 18 years old and above will be enrolled in this study. 1,350 participants who previously received Sinopharm inactivated COVID-19 vaccine will be enrolled to Cohort 1. 450 participants who previously received mRNA COVID-19 vaccine (Comirnaty from Pfizer or mRNA-1273 from Moderna) or previously diagnosed with COVID-19 will be enrolled to Cohort 2.

In Cohort 1, 300 participants who were previously fully vaccinated with 2 or 3 doses of Sinopharm inactivated COVID-19 vaccine and with no previous COVID-19 history will form an immunogenicity subgroup (Subgroup 1) for nAb tests, and will be randomly assigned to SCTV01C Group, SCTV01E Group and the Sinopharm inactivated COVID-19 vaccine Group in a ratio of 1:1:1. The 300 participants for nAb tests will be stratified by age (18-54 years, ≥

55 years) , number of doses of previously received COVID-19 vaccines (2, 3), and interval between previous vaccination and the study vaccination (3-5 months, 6-8 months, 9-12 months, 13-24 months).The first 150 participants will form a cellular immune response subgroup for cellular immune response tests.

In Cohort 1, in addition to the 300 participants for immunogenicity tests, there are 1050 other participants who previously received at least one shot of Sinopharm COVID-19 inactivated vaccine, will form a subgroup (Subgroup 2) mainly for safety observation, and will be randomly assigned to SCTV01C Group, SCTV01E Group and Sinopharm inactivated COVID-19 vaccine Group in a ratio of 1:1:1. The 1050 participants mainly for safety observation will be stratified by age (18-54 years, ≥55 years), previous COVID-19 infection history (yes or no), number of doses of previously received COVID-19 vaccines (1, 2, 3) and interval between previous vaccination and the study vaccination (3-5 months, 6-8 months, 9-12 months, 13-24 months).

In Cohort 2, 450 participants who previously received 2 or 3 doses of mRNA COVID-19 vaccine (Comirnaty or mRNA-1273) or previously diagnosed with COVID-19 will be randomly assigned to SCTV01C Group, SCTV01E Group and the mRNA-1273 Group in a ratio of 1:1:1. Participants will be stratified by age (18-54 years, ≥55 years), number of doses of previously received COVID-19 vaccines (0, 1, 2, 3), previous COVID-19 infection history (yes or no), and interval between previous vaccination and the study vaccination (3-5 months, 6-8 months, 9-12 months, 13-24 months). In Cohort 2, the number of participants previously diagnosed with COVID-19 and previously not received any mRNA COVID-19 vaccine, should not be more than 50. All participants will have nAb tests. The first 150 participants will form a cellular immune subgroup for cellular immune response tests.

In Cohort 1, each participant in SCTV01C Group will receive one dose of SCTV01C on D0; each participant in SCTV01E Group will receive one dose of SCTV01E on D0; each participant in Sinopharm inactivated COVID-19 vaccine Group will receive one dose of Sinopharm inactivated COVID-19 vaccine on D0.

In Cohort 2, each participant in SCTV01C Group will receive one dose of SCTV01C on D0; each participant in SCTV01E Group will receive one dose of SCTV01E on D0; each participant in mRNA-1273 Group will receive one dose of mRNA-1273 on D0.

Trial procedures:

The study procedure is described as Figure 1 and Figure 2. An independent data and safety monitoring board (DSMB) will review the data of the study.

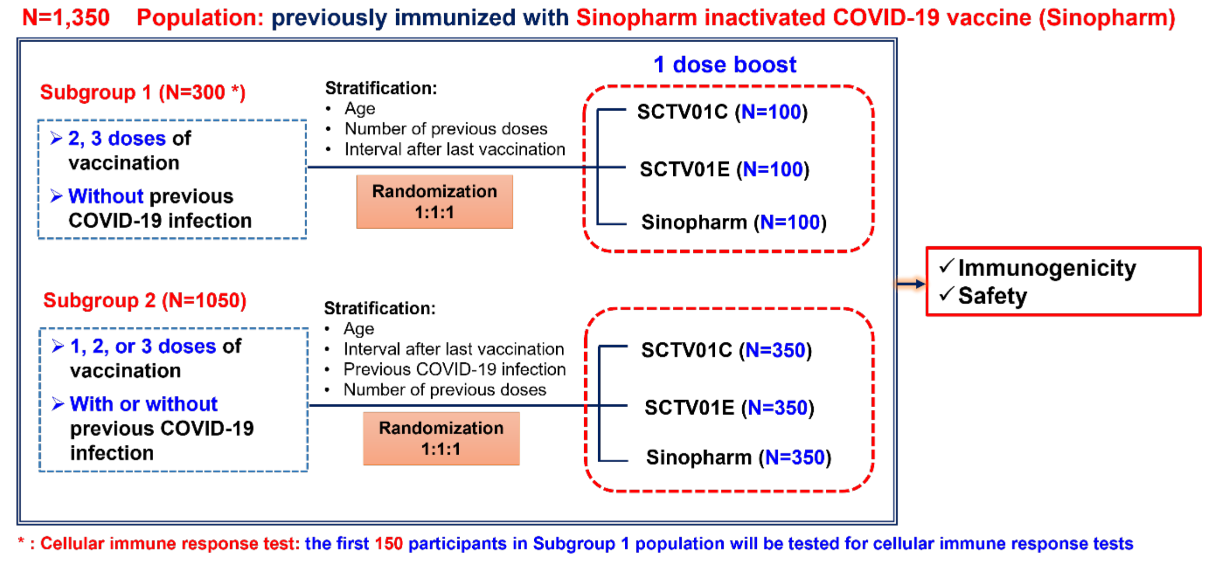

Figure 1 Study design for Cohort 1

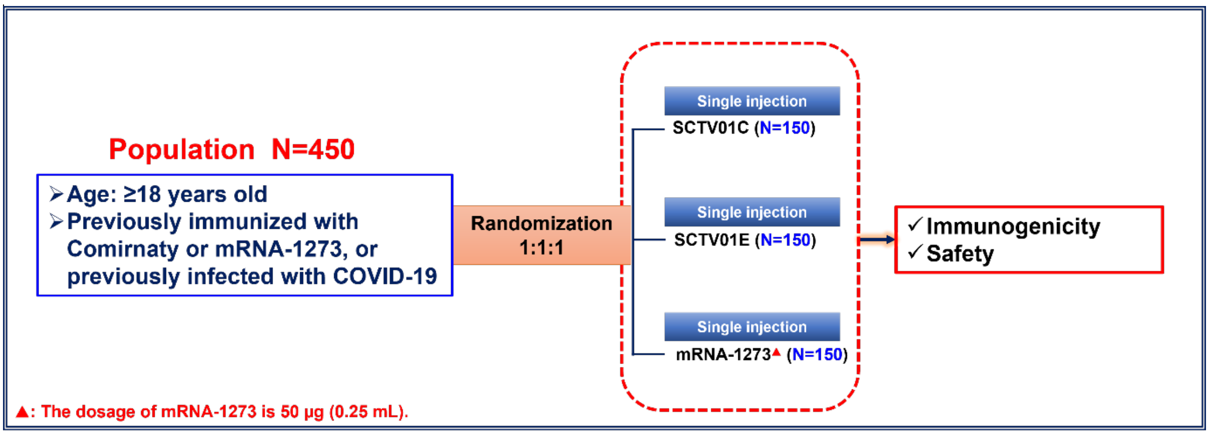

Figure 2 Study design for Cohort 2

The study consists of a screening period, a randomization and vaccination period, and a follow-up period.

Screening period: After participants sign the ICF, the screening phase visit will be conducted within 14 days.

Randomization: The qualified participants will be randomized before the study vaccination.

Vaccination: Randomized participants will be vaccinated on D0.

Follow-up:

Safety follow-up: The participants will be observed at site for at least 30 minutes after the study vaccination. Both the active monitoring and the spontaneous reporting will be used to collect the solicited and the unsolicited AEs. Solicited AEs within 7 days after study vaccination and unsolicited AEs within 28 days after study vaccination will be collected through vaccination record cards. AEs, SAEs and AESIs will be followed for 180±7 days after the study vaccination.

Immunogenicity follow-up: The participants in Subgroup 1 in Cohort 1 and all participants in Cohort 2 will be sampled for immunogenicity on D0 (before vaccination), D28 and D180. The nAb against Delta and Omicron variants will be tested.

The participants in the cellular immune response subgroup will be sampled for cellular immune response test on D0 (before vaccination) and D28.

After administration of the study vaccination, the participants will be continuously and systematically monitored for 180±7 days to ensure a prompt diagnosis and treatment according to FDA diagnosis and treatment practice when a participant experiences the suspicious symptoms of COVID-19. If a SARS-CoV-2 infection is confirmed 14 days after the study vaccination, sample will be collected from the nasopharyngeal/throat swab and viral sequencing will be used to identify the major SARS-CoV-2 variants.

The DSMB will review the safety data within D0-D28 of all the participants to assess the safety SCTV01C and SCTV01E.

Note:

Participants aged between 54 years to less than 55 years old will be taken as 54 years old.

3.2 BLINDING AND UNBLINDING METHODS

3.2.1 RANDOMIZATION

This clinical trial is a randomized, double-blind Phase III clinical trial. Participants are randomly assigned into the different groups according to the ratio specified in the protocol. In order to achieve the relative balance among each group, the participants will be stratified by specified stratification factors. The detail will be described in the randomization plan.

The eligible participants will be randomized by the Interactive Network Response System (IWRS) and vaccinated according to the random number. For randomized participants who withdraw from clinical trials for any reason, regardless of whether they have received the study vaccine, their random numbers will be retained, and participants who withdrew can no longer participate in this trial.

3.2.2 BLINDING AND UNBLINDING METHODS

3.2.2.1 Blinding

This study is randomized and double-blind. An unblinded team at the study site will be set. The unblinded study site personnel will manage vaccine logistics, preparation, and administration so as to maintain the blind from the remainder of the study site personnel and participants. The unblinded study site personnel administer study vaccine, but will not be involved in study-related assessments or have participant contact for data collection after administration of trial

vaccine.

Unblinded analysis by independent unblinded statistician can be performed after all participants have completed the safety assessment 28 days after the study vaccination.

When the experiment is in progress, the unblinded statistician shall ensure that the random blind codes are properly kept and handed over to the blind statistician to save with other project-related materials when the study is completed.

3.2.2.2 Unblinding

The study will be in an EDC/IWRS database. The sponsor and investigator will determine the time for unblinding in each phase of the clinical trial based on the progress of the study. Before unblinding, the principal investigator, sponsor and statistician must jointly sign relevant documents.

3.2.2.3 Unblinding under emergency

If there are serious adverse events or emergencies during the trial, when the investigator believes that it is essential to know the participant's group for his clinical treatment or health, it can be unblinded in emergency. The principal investigator or his designated person in charge should contact the sponsor directly to discuss the necessity of unblinding under emergency. After the sponsor confirms, unblinding under emergency of individual participants can be carried out. The investigator or his designated person in charge can apply for unblinding under emergency at IWRS and record the reason for unblinding. The participant will withdraw from the trial, and the reason for withdrawal must be recorded in the original data.

3.3 DEFINITION OF END OF STUDY

Definition of end of the study for individual participant: the participant completes all the visits specified in the protocol or terminates the study early due to various reasons.

Definition of end of study: the last participant planned to be enrolled completes the last visit specified in the protocol or terminates the study early due to various reasons.

4 STUDY POPULATION

4.1 INCLUSION CRITERIA

Participants are eligible to be included in the study only if the following conditions are met:

1. Male or female aged ≥18 years old when signing ICF;
2. **For Subgroup 1 in Cohort 1:** Participants who were previously vaccinated with 2 or 3 doses of Sinopharm inactivated COVID-19 vaccine. The interval between the date of last dose and the date of this study vaccination should be 3 to 24 months.

   For Subgroup 2 in Cohort 1: 1) Participants who were previously vaccinated with 2 or 3

doses of Sinopharm inactivated COVID-19 vaccine, with or without COVID-19 history; or 2) Participants who were previously vaccinated with 1 dose of Sinopharm inactivated COVID-19 vaccine and previously diagnosed with COVID-19. The interval between the date of last dose/COVID-19 diagnosis and the date of this study vaccination should be 3 to 24 months.

For Cohort 2: 1) Participants who were previously vaccinated with 2 or 3 doses of mRNA COVID-19 vaccine (Comirnaty or mRNA-1273), with or without COVID-19 history; or 2) Participants who were previously vaccinated with 1 doses of mRNA COVID-19 vaccine (Comirnaty or mRNA-1273) and previously diagnosed with COVID-19; or 3) Participants who were previously not vaccinated with any COVID-19 vaccine and previously diagnosed with COVID-19. The interval between the date of last dose/COVID-19 diagnosis and the date of this study vaccination should be 3 to 24 months.

3. The participant and/or his legally acceptable representative can sign written ICF, and can fully understand the trial procedure, the risk of participating in the trial, and other interventions that can be selected if they do not participate in the trial;
4. The participant and/or his legally acceptable representative have the ability to read, understand, and fill in record cards;
5. Healthy participants or participants with pre-existing medical conditions who are in stable condition. The “pre-existing medical conditions” include but not limited to hypertension, diabetes, chronic cholecystitis and cholelithiasis, chronic gastritis that meet the described criteria. A stable medical condition is defined as disease not requiring significant change in therapy or no need for hospitalization as a consequence of worsening disease state for at least 3 months prior to enrollment;
6. Fertile men and women of childbearing potential voluntarily agree to take effective contraceptive measures from signing ICF to 6 months after the study vaccination; the pregnancy test results of women of childbearing potential are negative on screening.

4.2 EXCLUSION CRITERIA

A participant who conforms to any of the following criteria should not be enrolled in the study:

1. **For Subgroup 1 in Cohort 1 only**: Previously diagnosed with COVID-19.
2. Presence of fever within 3 days before the study vaccination;
3. A history of infection or disease related to severe acute respiratory syndrome (SARS), Middle East respiratory syndrome (MERS), or other disease corresponding use of immunosuppressants;
4. A history of allergic reactions to any vaccine or drug, such as allergy, urticaria, severe

skin eczema, dyspnea, laryngeal edema, and angioneurotic edema;

5. A medical or family history of seizure, epilepsy, encephalopathy and psychosis;
6. Immunocompromised patients suffering from immunodeficiency diseases, important organ diseases, immune diseases (including Guillain-Barre Syndrome [GBS], systemic lupus erythematosus, rheumatoid arthritis, asplenia or splenectomy caused by any circumstances, and other immune diseases that may have an impact on immune response in the investigator's opinion), etc.;
7. Long-term use of immunosuppressant therapy or immunomodulatory drugs for ≥14 days within the first six months prior to enrollment. Whereas short-term (≤14 days) use of oral, inhaled and topical steroids are allowed;
8. Patients on antituberculosis therapy;
9. Presence of severe or uncontrollable cardiovascular diseases, or severe or uncontrollable disorders related to endocrine system, blood and lymphatic system, liver and kidney, respiratory system, metabolic and skeletal systems, or malignancies (skin basal cell carcinoma and carcinoma in-situ of cervix are exceptions and will not be excluded), such as severe heart failure, severe pulmonary heart disease, unstable angina, liver failure, or uremia;
10. Contraindications for intramuscular injection or intravenous blood sampling, including thrombocytopenia and other blood coagulation disorders;
11. Participants who received any immunoglobulin or blood products in the previous 3 months before enrollment, or plan to receive similar products during the study;
12. Participants who received other investigational drugs within 1 month before the study vaccination;
13. Participants who is at the acute state of disease, such as acute onset of chronic heart failure, acute sore throat, hypertensive encephalopathy, acute pneumonia, acute renal insufficiency, acute cholecystitis;
14. Participants received other drugs or vaccines used to prevent COVID-19, but participants previously received Sinopharm inactivated COVID-19 vaccine, Comirnaty or mRNA-1273 will not be excluded;
15. Participants vaccinated with influenza vaccine within 14 days or with other vaccines within 28 days before the study vaccination;
16. Those who donated blood or had blood loss (≥450 mL) within 3 months before the vaccination or plan to donate blood during the study period;
17. Those who are pregnant or breast-feeding or plan to be pregnant during the study period;

18. Those who plan to donate ovum or sperms during the study period;
19. Those who cannot follow the trial procedures, or cannot cooperate to complete the study due to planned relocation or long-term outing;
20. Those unsuitable for participating in the clinical trial as determined by the investigator because of other abnormalities that are likely to confuse the study results, or non-conformance with the maximal benefits of the participants;
21. Those who are tested positive for HIV in terms of serology.

5 DISCONTINUATION OF STUDY INTERVENTION

5.1 WITHDRAWAL CRITERIA

A participant may withdraw from the study at any time at his/her own request.

Reasons for discontinuation from the study may include the following:

- Refused further follow-up;
- Lost to follow-up;
- Death;
- Study terminated by sponsor;
- AEs;
- Participant request;
- Investigator request;
- Protocol deviation.

Handling participants withdrawing:

If a participant withdraws consent, the investigator must make every effort to determine the primary reason for this decision and record this information on the treatment disposition eCRF page. If the participant decides to completely withdraw from the study (refuses any further study participation or contact), all study participation for that participant will cease and data to be collected at subsequent visits will be considered missing. Further attempts to contact the participant are not allowed unless safety findings require communication or follow-up.

Participants may refuse further procedures (including vaccination) but are encouraged to remain in the study for safety follow-up. In such cases where only safety is being conducted, participant contact could be managed via phone call, short message, email or other contacting method.

If a participant withdraws from the study or terminates the study (including loss to follow-up) after enrollment, no participant replacement is allowed.

Participants will be informed of the importance of continuing to take other public health

measures, such as social distancing, wearing masks and washing hands, to control the spread of the disease.

5.2 STUDY SUSPENSION/TERMINATION CRITERIA

In one of the following situations, the trial should be suspended or terminated:

- When the DSMB requires a suspension/complete termination of the trial and the sponsor agrees;
- When the sponsor requires a suspension/complete termination of the trial and gives reasons for it;
- When the Ethics Committee requires a suspension/complete termination of the trial and gives reasons for it;
- When the regulatory agency requires a suspension/complete termination of the trial and gives reasons for it.

6 DESCRIPTION OF STUDY PROCEDURES AND VISITS

All required study procedures and evaluations are to be conducted as outlined in this protocol. In the event of a deviation from the protocol due to an emergency, accident, or mistake, the investigator or designee must notify the sponsor as soon as possible.

Additional assessments (vital signs, ECG, laboratory test, etc.) can be done at the discretion of the investigators as clinically indicated.

This study includes a screening period, a randomization and vaccination period, and follow-up period (the follow-up period includes safety follow-up and immunogenicity follow-up), using a combination of on-site visits and electronic forms such as phone call visits.

6.1 V1 (SCREENING, D-14~D0)

After the participants sign the informed consent form, they will be screened during the screening period (14 days before vaccination to the day of vaccination), and their baseline data will be collected. Investigators will determine whether the participants can be included in the clinical trial according to the "inclusion/exclusion criteria". Investigators fill the participant information collected during the screening period (including screening number, age, disease history or vaccination history, test results in screening period, screening date, enrollment status and location [if applicable], and reasons for non-compliance with study enrollment [if applicable], etc.) into the original file and the corresponding part of the eCRF.

The following check must be completed before enrollment:

- Confirming and collecting the ICF signed by the participant;
- Reviewing the inclusion/exclusion criteria;

- Demographic data: including age, sex, race, ethnicity, occupation (work, living environment), height, weight, BMI (calculated by height and weight). The participants should also provide contact information like current phone number and/or E-mail. In subsequent follow-up visits, if the contact information is changed, it should be updated accordingly (if applicable).
- Records of medical history: including the history of SARS-COV-2 vaccination, history of COVID-19, other vaccinations within 90 days, medication use within 28 days, major surgery, allergic history, and other known significant diseases.
- Assigning screening number;
- Physical examination: including general conditions, head and neck, lymph nodes, skin, chest, abdomen, musculoskeletal system and other examinations necessary for the study;
- Vital signs: including blood pressure, respiration rate, pulse rate, and body temperature;
- HIV test.
- Nasal/pharyngeal/throat swab nucleic acid test: The result of nucleic acid test for SARS-CoV-2 within 7 days prior to vaccination shall be obtained. If there is no available nucleic acid test report, the patient shall be tested at the study site, with negative result in nucleic acid test for SARS-CoV-2 to be obtained before vaccination.
- Urine pregnancy test (for women of childbearing potential only).

6.2 V2 (D0, VACCINATION)

The specific visit content is as follows (if the screening and the vaccination are on the same day, there is no need to repeat the vital signs and urine pregnancy test before vaccination):

- Vital signs: including blood pressure, respiration rate, pulse rate, and body temperature;
- Urine pregnancy test (for women of childbearing potential only);
- Randomization: participants are randomized after all the tests have been done and eligibility has been confirmed;
- Vaccination;

Immunogenicity follow-up visit

- Cellular immune response test (before vaccination and only applied to the cellular immune response subgroup): IL-4 and IFN-γ;
- Neutralizing antibodies test against Delta and Omicron variants (before vaccination and only applied to participants of Subgroup 1 in Cohort 1 and all participants in

Cohort 2);

Safety follow-up visit

- Observing for at least 30 minutes after the vaccination;
- Distribution of vaccination record cards (VRCs): distribute the VRCs after the study vaccination;
- Distributing clinical thermometers and instructing participants to measure and record body temperature;
- Solicited and unsolicited AEs: collect solicited AEs and unsolicited AEs after the study vaccination;
- AEs, SAEs and AESIs;
- Recording the concomitant medication.

Providing the participants with an emergency contact number and instructing them to contact the designated medical center immediately when an event that requires emergency medical treatment occurs after the study vaccination. Providing the participants with thermometers and tape measures, and instructing them to record symptoms, signs, and the severity of adverse events within 28 days after the study vaccination. Participants should record solicited and unsolicited AEs within 7 days after the study vaccination, unsolicited AEs within 28 days and concomitant medications after the study vaccination, and other information on the VRC.

6.3 V3 (D7+2D)

Safety follow-up visit

- Solicited and unsolicited AEs: collect solicited AEs and unsolicited AEs after the study vaccination;
- AEs, SAEs and AESIs;
- Reviewing and recovering the VRCs;
- Recording the concomitant medication.

6.4 V4 (D28+3D)

Immunogenicity follow-up visit

- Cellular immune response test (only applied to the cellular immune response subgroup): IL-4 and IFN-γ;
- Neutralizing antibodies test against Delta and Omicron variants (only applied to participants of Subgroup 1 in Cohort 1 and all participants in Cohort 2);

Safety follow-up visit

- Reviewing and recovering the VRCs;

- Unsolicited AEs: collect unsolicited AEs after the study vaccination;
- AEs, SAEs and AESIs;
- Recording the concomitant medication.

6.5 V5/EOS (D180±7D)

Immunogenicity follow-up visit

- Neutralizing antibodies test against Delta and Omicron variants (only applied to participants of Subgroup 1 in Cohort 1 and all participants in Cohort 2);

Safety follow-up visit

- AEs, SAEs and AESIs;
- Recording the concomitant medication: After 28 days after the study vaccination, only the concomitant medication used to treat AEs, SAEs and AESIs should be recorded.

6.6 UNPLANNED CONTACT AND FOLLOW-UP

At the request of the participant, or during the study period, the investigator may conduct unplanned contact and follow-up with the participant (a visit other than the follow-up specified in the regular schedule) according to the situation. All unplanned contacts and follow-ups will be recorded in the participant's original file and eCRF.

7 STUDY VACCINES

7.1 BASIC INFORMATION OF VACCINES

Study vaccine 1

Name:	Bivalent SARS-CoV-2 trimeric spike protein vaccine (SCTV01C)
Components:	Main active ingredients: SCTV01C-TM22 protein, SCTV01C-TM23 protein;<br>SCT-VA02B adjuvant: The adjuvant 1X is comprised of 0.09 mg of citric acid, 0.59 mg of sodium citrate, 1.25 mg of polysorbate 80, 1.25 mg of span 85 and 10.75 mg of squalene;<br>Excipients: citrate, sodium citrate, sodium chloride, polysorbate 80, sodium hydroxide, WFI;
Dosage form:	Solution for injection
Appearance:	Emulsified, white suspension (due to the presence of adjuvant)
Strength:	20μg (10/10μg for TM22/TM23) /0.5mL/vial;
Storage conditions:	Stored and transported at 2~8°C away from light;
Validity period:	24 months
Manufacturer:	Sinocelltech Ltd.

Study vaccine 2

Name:	COVID-19 Alpha/Beta/Delta/Omicron Variants S-Trimer Vaccine (SCTV01E)
Components:	Main active ingredients: SCTV01E-TM22 protein, SCTV01E-TM23 protein, SCTV01E-TM28 protein, SCTV01E-TM41 protein;<br>SCT-VA02B adjuvant: The adjuvant 1X is comprised of 0.09 mg of citric acid, 0.59 mg of sodium citrate, 1.25 mg of polysorbate 80, 1.25 mg of span 85 and 10.75 mg of squalene;<br>Excipients: citrate, sodium citrate, sodium chloride, polysorbate 80, sodium hydroxide, WFI;
Dosage form:	Solution for injection
Appearance:	Emulsified, white suspension (due to the presence of adjuvant)
Strength:	30μg (5/5/5/15μg for TM22/TM23/TM28/TM41)/0.5mL/vial;
Storage conditions:	Stored and transported at 2~8°C away from light;
Validity period:	24 months
Manufacturer:	Sinocelltech Ltd.

Sinopharm inactivated COVID-19 vaccine： The dosage is 0.5 mL. The strength of Sinopharm inactivated COVID-19 vaccine is 6.5U/0.5mL/vial. The vaccine will be stored and transported at 2~8℃ away from light.

mRNA-1273: The dosage of mRNA-1273 is 50 μg (0.25 mL). The vaccine will be stored and transported at -50~-15℃ away from light.

7.2 STUDY VACCINE MANAGEMENT

SCTV01C, SCTV01E and Sinopharm inactivated COVID-19 vaccine will be stored and transported at 2~8°C away from light. mRNA-1273 will be stored and transported at -50~-15°C away from light.

Study vaccines are provided by the sponsor free of charge and distributed to the study sites as planned. Trained personnel at the study site are responsible for recording the receipt and preservation of vaccines, medication for each participant, recovery and maintenance. Only eligible participants will receive the study vaccine. All study vaccines must be stored in a safe, environmentally controlled and (manual or automatic) monitoring area according to the prescribed storage conditions, and only investigators and authorized site staff can obtain them. The disposed, expired, and remaining study vaccines should be destroyed in accordance with the requirements of the sponsor, the vaccine management guidelines or equivalent documents.

7.3 VACCINATION ROUTE AND DOSE

Vaccination site: the deltoid muscle on the outer side of the upper arm;

Vaccination route: intramuscular injection;

Vaccination dose: SCTV01C 20μg; SCTV01E 30μg; Sinopharm inactivated COVID-19 vaccine: 0.5mL; mRNA-1273: 50 μg (0.25 mL).

Before injection, the injection site should be disinfected with 75% alcohol, and the study vaccine is injected intramuscularly after the skin is slightly dry. Before each vaccination, the study vaccine should be shaken slightly before extraction. If the vaccine is found to be abnormal, such as abnormal color, broken vial, insufficient medicine, unclear label, etc., it must not be used, and a spare vaccine can be used for vaccination. The participants will be observed at site for at least 30 minutes after each study vaccination. The site should be equipped with appropriate emergency medical treatment measures to treat possible allergic reactions after the study vaccination.

Note: The deltoid muscle of the non-dominant hand is preferred for injection; it is forbidden to inoculate in the buttocks and other parts. Do not vaccinate within 2 cm of tattoos, scars or skin defects. Strictly follow the standard vaccination method and do not inject the vaccine into the blood vessel. For other precautions for vaccination, please refer to the SCTV01C Investigator's Brochure and SCTV01E Investigator's Brochure.

7.4 VACCINATION PROCEDURE

Before each vaccination, the information of the participant and the study vaccine must be checked. All participants receive 1 doses of study vaccine (SCTV01C or SCTV01E), active comparator (Sinopharm inactivated COVID-19 vaccine or mRNA-1273) on D0.

7.5 CONCOMITANT MEDICATION

Concomitant medication refers to all drugs used by participants during the study period except the study vaccine, including treatments related to AEs, SAEs and AESIs that occurred during the study period.

The information of the concomitant medication, including the name of the drug, the purpose of administration, the usage and dosage, and the time of use, must be recorded in the eCRF in detail.

7.5.1 ALLOWED CONCOMITANT MEDICATION

During the study period, the following drugs are allowed:

- Drugs used to control concomitant diseases are allowed to be used continuously during the study period if the investigator judges that they are not expected to interfere with the test results;

- Necessary drug treatment for the participant's AEs is allowed;
- Participants diagnosed with COVID-19 after the study vaccination are allowed to be treated according to local standards;
- During the study period, if participants are participant to routine immunization with vaccines other than COVID-19 vaccine, they can be vaccinated according to the product manual, but there must be an interval of 14 days between the routine immunization and the test vaccination. Vaccinations for medical emergencies, such as rabies or tetanus, can be vaccinated in time according to the product instructions.

7.5.2 PROHIBITED CONCOMITANT MEDICATION

During the study period, the following drugs are prohibited:

- Prohibiting any other COVID-19 preventive medication;
- Prohibiting unapproved drugs/vaccines other than the study vaccine;
- Prohibiting long-term use of (continuous use> 14 days) glucocorticoids (dose ≥0.5 mg/kg/d prednisone or equivalent) or other immunosuppressive agents (except for inhaled and topical corticosteroid, or short-term ≤14 days oral steroids);
- Prohibiting immunoglobulin or other blood products;
- The participants should avoid taking over-the-counter drugs, such as antipyretics (such as acetaminophen) and anti-inflammatory drugs (such as ibuprofen, naproxen, etc.) within 12 hours before each vaccination.

7.6 PARTICIPANT COMPLIANCE

Participants will receive the study vaccine directly from the staff of the study site. The staff of the study site record the detail of the date and specific time of the participant's vaccination in the original documents and eCRF.

8 COLLECTION, PROCESSING AND TESTING OF BIOLOGICAL SAMPLES

8.1 COLLECTION, PROCESSING AND TESTING OF IMMUNOGENICITY SAMPLES

8.1.1 COLLECTION OF BLOOD SAMPLES

The participants in Subgroup 1 in Cohort 1 and all participants in Cohort 2 will be sampled for neutralizing antibody test against Delta and Omicron variants on D0 (before vaccination), D28 and D180. The amount of collected blood for nAb testing each time is not more than 10mL, which will depend on actual laboratory requirement.

The participants in the cellular immune response subgroup will be sampled for cellular immune response test on D0 (before vaccination) and D28. The amount of collected blood for cellular

immune response testing each time is not more than 10mL, which will depend on actual laboratory requirement.

8.1.2 PROCESSING AND STORAGE OF BLOOD SAMPLES

The biological samples collected in this study will be properly stored as required and will only be used for the study and tests specified in the protocol. The participant can revoke the permission for other ways of using the sample in the future at any time. In this case, the sample will be destroyed after the end of the study. The investigator will be responsible for destroying all remaining samples and reporting to the institutional review board (IRB).

For processing, storage and transportation of blood samples, please refer to the relevant documents of the laboratory management manual.

8.2 COLLECTION, PROCESSING AND TESTING OF VIRUS TEST SAMPLES

8.2.1 COLLECTION OF VIRUS TEST SAMPLES

It is necessary to obtain a SARS-CoV-2 nucleic acid test negative report within 7 days before vaccination. If the patient has no nucleic acid test report, he will be tested at the study site and obtain SARS-CoV-2 nucleic acid test negative report before vaccination.

For all participants who have first suspicious symptoms, two samples of nasal/nasopharyngeal/throat swab should be collected within 72 hours, one of which is for the RT-PCR test, the other one is for sequencing in case that the diagnosis of COVID-19 is confirmed. If the onset is more than 72 hours, it should be collected as soon as possible. The sampling tube should be labeled with the random number. The label should also indicate the initials, gender, type of sample, and date of collection. The specific content of the label can be formulated with reference to the corresponding regulations of the regulatory authority of the country where the trial is located. The collected samples should be sent for tests in time.

8.2.2 STORAGE AND TRANSPORTATION OF VIRUS TEST SAMPLES

For details, please refer to the biological sample management guidelines or equivalent documents.

8.2.3 VIRUS-SPECIFIC NUCLEIC ACID TEST

RT-PCR for SARS-CoV-2 nucleic acid test is adopted, using polymerase chain reaction (PCR) amplifiers and kits approved by the drug regulatory authority approved by the sponsor.

RT-PCR testing of samples from participants is carried out by testing laboratories that meet local regulatory standards. If the test result of a suspicious case is in doubt, the test should be repeated.

9 STUDY EVALUATIONS AND REPORTS

9.1 SAFETY ASSESSMENT

All participants will have safety follow-ups until 180±7 days after the study vaccination.

The participants will be observed at the study site for at least 30 minutes after each study vaccination, and the solicited and unsolicited AEs at the vaccination site (local) and non-vaccination site (systemic) will be reported during this period. Active monitoring and spontaneous reporting will be used to collect the solicited AEs within 7 days and unsolicited AEs within 28 days after the study vaccination and to monitor AEs, SAEs and AESIs within 180±7 days after the study vaccination.

Participants need to record the occurrence of solicited AEs within 7 days and unsolicited AEs within 28 days after the study vaccination on the VRCs and return to the study site with the VRCs on specified time points. And the safety information will be recorded into eCRF.

AEs, SAEs, and AESIs will be collected on visit day or reported by participants actively at any time. If participants cannot come to site on visit day, phone call, short message, email or other contacting method will be used for safety follow-up. The sponsor will provide a telephone number and instruct the patients to call in case of any adverse events to receive medical assistance. Participants can actively report their AEs at any time until 180±7 days after the study vaccination.

Note: The investigator should report all serious adverse events to the sponsor and the contract research organization (CRO) designated by the sponsor within 24 hours after being informed of the SAEs and should also report the SAEs to the ethics committees (ERC/IRB) and local regulatory agencies in accordance with local regulations.

9.1.1 DEFINITION

9.1.1.1 AEs

AE refers to all the adverse medical events that occur after the participant receives the study vaccine. It can be manifested as symptoms and signs, diseases, or abnormal laboratory tests, which does not necessarily have to have a causal relationship with the study vaccine. Previous stable conditions that have been abnormal in the past and whose severity has not changed during the trial period are not regarded as AEs but should be recorded in medical history.

9.1.1.2 Adverse reaction

All noxious and unintended responses to a medicinal product related to any dose should be considered adverse drug reactions. The phrase “responses to a medicinal product” means that a causal relationship between a medicinal product and an adverse event is at least a reasonable possibility, i.e., the relationship cannot be ruled out.

9.1.1.3 Solicited AEs

Solicited AEs are pre-specified and actively monitored during the study, and participants are required to record solicited AEs. Investigators will conduct assessment for solicited AE collected within the first 30 minutes after the study vaccination, and 7 days after the study vaccination (data collected from day 0 to day 7). Participants will be provided with VRCs to record whether solicited AEs occur and record the severity and concomitant medications.

Solicited AEs can be divided into injection-site (local) adverse events and non-injection-site (systemic) adverse events according to the site of occurrence. See Table **2** for detailed information.

Table 2 List of solicited AE

Solicited local AEs	Solicited systemic AEs
• Pain at the injection site<br>• Tenderness at the injection site<br>• Erythema at the injection site<br>• Redness at the injection site<br>• Swelling at the injection site<br>• Induration at the injection site	• Fever<br>• Nausea/Vomiting<br>• Headache<br>• Fatigue<br>• Myalgia<br>• Arthralgia, joint pain<br>• Chill

9.1.1.4 Unsolicited AEs

Unsolicited AEs are not specified for active monitoring. An unsolicited AE is any AE reported by the participant that is not specified as a solicited AE in the protocol; or is specified as a solicited AE in the protocol, but starts outside the protocol-defined period for reporting solicited AEs (ie, for the 7 days after the IP).

The investigators assess the relevance and severity of unsolicited AEs based on the FDA guidelines in the appendix.

9.1.1.5 SAEs

A serious adverse event or reaction is any untoward medical occurrence that at any dose:

* results in death,
* is life-threatening,

NOTE: The term "life-threatening" in the definition of "serious" refers to an event in which the patient was at risk of death at the time of the event; it does not refer to an event which hypothetically might have caused death if it were more severe.

* requires inpatient hospitalization or prolongation of existing hospitalization,
* results in persistent or significant disability/incapacity, or
* is a congenital anomaly/birth defect.

Medical and scientific judgement should be exercised in deciding whether expedited reporting is appropriate in other situations, such as important medical events that may not be immediately life-threatening or result in death or hospitalization but may jeopardize the patient or may require intervention to prevent one of the other outcomes listed in the definition above. These should also usually be considered serious.

Hospitalizations due to elective surgery, routine clinical procedures, annual check-ups, and hospitalization observation or protocol, rather than adverse events are not considered as serious adverse events. If an unexpected event occurs during this process, it should be reported as a "serious" or "non-serious" adverse event according to conventional standards.

Note: Hospitalization or prolongation of the hospitalization period due to non-medical reasons/convenience, etc. or only for clinical trial purposes does not meet the criteria for medical events and therefore cannot be regarded as a SAE.

9.1.1.6 AESIs

AESIs refers to adverse events that are of special concern to study vaccines from a scientific or medical point of view.

Throughout the study, AESIs will be collected according to Safety Platform for Emergency Vaccines (SPEAC) and reported to the sponsor within 24 hours after awareness of investigator.

Table 3 List of Potential Immune-mediated Diseases to be Collected in the Context of Vaccines Containing Adjuvant System

**Neuroinflammatory disorders**	**Musculoskeletal disorders**	**Skin disorders**
● Cranial nerve neuropathy, including paralysis and paresis (eg, Bell's palsy).<br>● Optic neuritis.<br>● Multiple sclerosis.<br>● Transverse myelitis.<br>● Guillain-Barre syndrome, including Miller Fisher syndrome and other variants.<br>● Acute disseminated encephalomyelitis, Including site specific variants, eg, noninfectious encephalitis, encephalomyelitis, myelitis, myeloradiculoneuritis.<br>● Myasthenia gravis, including Lambert-Eaton myasthenic syndrome.<br>● Demyelinating peripheral neuropathies including:<br>- Chronic inflammatory demyelinating polyneuropathy.<br>- Multifocal motor neuropathy.<br>- Polyneuropathies associated with monoclonal gammopathy.<br>● Narcolepsy.	● Systemic lupus erythematosus and associated conditions.<br>● Systemic scleroderma (systemic sclerosis), Including:<br>- Diffuse scleroderma.<br>- CREST Syndrome.<br>● Idiopathic inflammatory myopathies, Including:<br>- Dermatomyositis.<br>- Polymyositis<br>● Antisynthetase syndrome.<br>● Rheumatoid arthritis and associated conditions Including:<br>- Juvenile idiopathic arthritis.<br>- Still' s disease.<br>● Polymyalgia rheumatica.<br>● Spondyloarthropathies, Including:<br>- Ankylosing spondylitis.<br>- Reactive arthritis (Reiter's syndrome).<br>- Undifferentiated Spondyloarthritis.<br>- Psoriatic arthritis.<br>- Enteropathic arthritis.<br>● Relapsing polychondritis.<br>● Mixed connective tissue disorder.<br>● Gout.	● Psoriasis.<br>● Vitiligo.<br>● Erythema nodosum.<br>● Autoimmune bullous skin diseases (including pemphigus, pemphigoid, and dermatitis herpetiformis)<br>● Lichen planus.<br>● Sweet's syndrome.<br>● Localized scleroderma (morphea).
**Vasculitis**	**Blood disorders**	**Others**
● Large vessels vasculitis Including:	● Autoimmune hemolytic anemia.<br>● Autoimmune thrombocytopenia.	● Autoimmune glomerulonephritis Including:

- Giant cell arteritis (temporal arteritis).<br>- Takayasu's arteritis<br>● Medium sized and/or small vessels vasculitis including:<br>- Polyarteritis nodosa.<br>- Kawasaki's disease.<br>- Microscopic polyangiitis.<br>- Wegener's granulomatosis (granulomatosis with polyangiitis)<br>- Churg-Strauss syndrome (allergic granulomatous angiitis or eosinophilic granulomatosis with polyangiitis)<br>- Buergers disease (thromboangiitis Obliterans).<br>- Necrotizing vasculitis (cutaneous or systemic)<br>- Antineutrophil cytoplasmic antibody positive vasculitis (type unspecified)<br>- Henoch-Schonlein purpura (IgA vasculitis).<br>- Behcet' s syndrome.<br>- Leukocytoclastic vasculitis.	● Antiphospholipid syndrome.<br>● Pernicious anemia.<br>● Autoimmune aplastic anemia<br>● Autoimmune neutropenia.<br>● Autoimmune pancytopenia.	- IgA nephropathy.<br>- Glomerulonephritis rapidly progressive.<br>- Membranous glomerulonephritis.<br>- Membranoproliferative glomerulonephritis.<br>- Mesangioproliferative glomerulonephritis.<br>- Tubulointerstitial-nephritis and uveitis syndrome.<br>● Ocular autoimmune diseases Including:<br>- Autoimmune uveitis.<br>- Autoimmune retinitis.<br>● Autoimmune myocarditis.<br>● Sarcoidosis.<br>● Stevens-Johnson syndrome.<br>● Sjögren's syndrome.<br>● Alopecia areata.<br>● Idiopathic pulmonary fibrosis.<br>● Goodpasture syndrome.<br>● Raynaud's phenomenon.
**Liver disorders**	**Gastrointestinal disorders**	**Endocrine disorders**
● Autoimmune hepatitis.<br>● Primary biliary cirrhosis.<br>● Primary sclerosing cholangitis.<br>● Autoimmune cholangitis.	● Inflammatory bowel disease, including:<br>- Crohn's disease.<br>- Ulcerative colitis.<br>- Microscopic colitis.<br>- Ulcerative proctitis.<br>● Celiac disease.<br>● Autoimmune pancreatitis.	● Autoimmune thyroiditis<br>● (Hashimoto thyroiditis).<br>● Grave' s or Basedow' s disease.<br>● Diabetes mellitus type I.<br>● Addison' s disease.<br>● Polyglandular autoimmune<br>● syndrome.<br>● Autoimmune hypophysitis.

IgA = immunoglobulin A

Table 4 List of Adverse Events of Special Interest Applicable to COVID-19 vaccines (Guidance Document from SPEAC)

**Body System**	**AESI Type**	**Rationale for Inclusion as an AESI (see Footnotes)**
Neurologic	Generalized convulsion	1，2，4
	Guillain-Barré Syndrome	2
	Acute disseminated encephalomyelitis	3
Hematologic	Thrombocytopenia	1，2
Immunologic	Anaphylaxis	1，2
	Vasculitides	3，4
Other	Serious local/systemic AEs following immunization	1，2

AE = adverse event, AESI = adverse events of special interest, COVID-19 = Coronavirus disease-2019.
1.Proven association with immunization encompassing several different vaccines.
2.Proven association with vaccine that could theoretically be true for CEPI vaccines under development.
3.Theoretical concern based on immunopathogenesis.
4.Theoretical concern related to viral replication during wild type disease.
5.Theoretical concern because it has been demonstrated in an animal model with 1 or more candidate vaccine platforms.

Table 5 List of Adverse Events of Special Interest Relevant to COVID-19 (Guidance Document from SPEAC)

**Body System**	**AESI Type**	**Rationale for Inclusion as an AESI (see Footnotes)**
Respiratory	Acute respiratory distress syndrome	3, 4
	Pneumonitis	3, 4
Immunologic	Enhanced disease following immunization	1, 2, 5
Other	Acute cardiac injury	3, 4
	Arrhythmia	3, 4
	Septic shock-like syndrome	3, 4
	Acute kidney injury	3, 4

	Multi-system inflammatory syndrome similar to Kawasaki's disease	
	Angioedema	

AESI = adverse event of special interest, CEPI = Coalition for Epidemic Preparedness Innovations, CoV = Coronavirus, COVID-19 = Coronavirus disease-2019, HIV = human immunodeficiency syndrome, MERS = middle-eastern respiratory syndrome, SARS = severe acute respiratory syndrome.

1.Proven association with immunization encompassing several different vaccines (formalin-inactivated measles/RSV vaccines; HIV vaccine)

2.Proven association with vaccine that could theoretically be true for CEPI vaccines under development (Chimeric Yellow Fever Dengue vaccine)

3.Theoretical concern based on immunopathogenesis.

4.Theoretical concern related to viral replication during wild type disease.

5.Theoretical concern because it has been demonstrated in an animal model with 1 or more candidate vaccine platforms (mouse models SARS/MERS-CoVs).

The AESIs of this study will be updated or revised with the collection of cumulative safety data.

9.1.1.7 Suspected and unexpected severe adverse reaction

Suspected Unexpected Serious Adverse Reaction (SUSAR) means the nature, severity, outcome, or frequency of these adverse reactions are not consistent with the risk information in the current relevant applicable product information (such as the investigator's brochure). The investigator's brochure serves as the main document to provide safety reference information for judging whether an adverse reaction is expected or unexpected.

9.1.1.8 Severity of adverse events

The grading scales used to assess adverse events are derived from the "Toxicity Rating Scale for Healthy Adult and Adolescent Volunteers in Preventive Vaccine Clinical Trial-FDA Standard" (Appendix I);

For adverse events not listed in the grading table, the intensity will be assessed according to the following standards. For details, see Table 6 .

Table 6 General principles for the grading of adverse events

Grade 1	Grade 2	Grade 3	Grade 4	Grade 5
**Mild**: No interference with activity	**Moderate**: Some interference with activity not requiring medical intervention	**Severe**: Prevents daily activity and requires medical intervention	**Potentially life threatening**: ER visit or hospitalization	**Death**

9.1.1.9 Causality between adverse events and vaccines

In this study, solicited local adverse events are considered to be related to vaccination. For solicited systemic adverse events, unsolicited adverse events, serious adverse events and ASEIs, the investigator is obligated to assess the relationship between study vaccine and each occurrence of each AEs/SAEs. Investigators should assess the relationship between adverse events and study vaccine in a timely manner, make clinical judgments based on the information available at the time of the report, and change the opinion of causality in light of the follow-up information.

Investigators are asked to use a simple binary decision for drug causality (related or not related) for adverse events. One possible approach that has been suggested is to ask simply was there a reasonable possibility? Yes or No.

Yes: There is a plausible temporal relationship between adverse events and the study vaccine, and the adverse events cannot be explained by the participant's clinical status, concurrent disease or concomitant treatment; and/or adverse events follow the known response pattern of the study treatment; and/or once the study vaccine is discontinued or the dose is reduced, the adverse event is improved or recovered, and the adverse event occur again after the vaccine is re-administered under appropriate circumstances.

No: Evidence shows that the adverse event has other triggers other than the study vaccine (for example, original medical condition, underlying disease, concurrent disease or concomitant medication); and/or there is no reasonable temporal relationship between the adverse event and the study vaccination.

9.1.2 OUTCOME OF ADVERSE EVENTS

The outcome of AEs can be described as the following:

- Recovered/Resolved: "Termination date of adverse events" should be indicated. Recovered to baseline level is Recovery.
- Recovered/Resolved with sequelae: only if the participant has long-term or lifelong sequelae, such as blindness caused by diabetes and hemiplegia after stroke. "Termination date of (serious) adverse event" should be indicated.
- Recovering/Resolving: The event has not yet been completely resolved, but the participant is already in the recovery phase.
- Not Recovered/Not Resolved: The event is in progress.
- Fatal: Death of participants directly or mainly caused by AEs.
- Unknown: The investigator cannot obtain the information of the AEs, for example, the participant is lost to follow-up.

The end date of the adverse event is the date at which the participant recover or recover with sequelae or the participant died.

If the outcome of the adverse event is assessed as "recovering", or "unrecovered", or "unknown", it is temporarily not necessary to record the end date of the adverse event.

If the outcome of an adverse event is assessed as "recovered" or "recovered with sequelae", the end date of the adverse event must be recorded.

9.1.3 RECORDING OF ADVERSE EVENTS

9.1.3.1 Time Period for Collecting Adverse Events

Solicited AEs are collected within 7 days after each study vaccination; unsolicited AEs are collected within 28 days after each study vaccination; AEs, SAEs and AESIs are collected within 180±7 days after the study vaccination.

The adverse medical occurrences that begin after signing the informed consent and before the study vaccination will be recorded in the "Medical History/Current Medical Condition" section of the CRF instead of the "AE" section.

SAEs (including death) occurring in a participant after withdrawal from the study must be reported to the sponsor or designee if the investigator becomes aware of them and believes have a reasonable possibility of being related to study vaccine.

For the solicited symptoms and unsolicited symptoms, the investigator should confirm with the participant whether he/she received hospitalization, outpatient treatment, or self-administered medication for any reason, and record this information.

The training for participants emphasizes on the timely reporting of AE. Investigators should be highly vigilant about such events, investigate and deal with them in a timely manner.

When a SAE occurs, the investigator is responsible for reviewing all documents related to the event (such as hospital history records, laboratory reports, and diagnostic reports), or in order to clarify the nature and causality of the SAEs. If the participant is confirmed dead during the study period, the hospital's final conclusions about the deceased should be collected. If an autopsy is performed, a copy of the results, including histopathological results, should be obtained.

9.1.3.2 Methods of discovering adverse events

At each visit, AEs can be found by the following methods:

- Information proactively provided by the participant or caregiver; when the participant has an acute or gradually worsening adverse reaction, the investigator or the corresponding contact person should be contacted for further treatment opinions and/or measures.
- At each follow-up, ask the participants open and non-leading questions: such as "How do you feel? Have you had any (other) medical problems since the last follow-up visit? "
- Abnormalities observed by investigators, other medical staff, and family members.

Investigators will also provide participants with VRCs (electronic and/or paper) to record solicited AEs from 0 to 7 days after each study vaccination and unsolicited AEs from 0 to 28 days after each study vaccination.

9.1.3.3 Recording and follow-up of adverse events

The investigator is responsible for recording all AEs and SAEs, and reports to the sponsor and the sponsor designated CRO within 24 hours after learning of the SAEs. It is required to collect AEs from day 0 to day 7 after each study vaccination, and unsolicited AEs from day 0 to day 28 after each study vaccination, SAEs from day 0/vaccination throughout the study period. During each study site visit or remote follow-up, participants will be questioned including COVID-19 symptom monitoring to ensure their safety. At the same time, the participants will be asked whether they have been hospitalized, whether an accident occurred, whether they are using new drugs, whether they have changed the concomitant medication regimen (including prescription drugs and over-the-counter drugs), or whether they are vaccinated with non-experimental vaccines. Physical examination results or other adverse event information related to the safety of the participant should be recorded. After investigators complete the AE and SAEs reports, they should continue to follow up the AE and SAEs during follow-up visits. All AEs and SAEs that occurred during the study should be treated correspondingly, and followed up until recovery, improvement, stability or other outcomes [investigators believe that no further follow-up is necessary for reasonable reasons (such as it cannot be recovered or has improved); when no more information can be obtained (for example, the participant refuses to provide more information, or evidence shows that the participant is still lost to follow-up after best efforts have been made)], or the participant is lost to follow-up.

In order to improve the quality and accuracy of information collection on adverse events, investigators should follow the following guidelines:

- When AE is recorded in eCRF, use recognized medical terms as much as possible;
- Record diagnostic results (i.e., diseases or syndromes), rather than related signs, symptoms, and laboratory test results (for example, record congestive heart failure instead of dyspnea, rales and cyanosis);
- Record and report the SAEs that caused the death;
- For patients who are hospitalized due to surgical procedures or diagnostic procedures, the disease that leads to the surgical procedures or diagnostic procedures, not the procedure itself, should be recorded as SAEs. This process should be recorded in the disease treatment measures in the case narrative;
- Pregnancies of participants during the study are not considered as adverse events, but

should be recorded in a separate pregnancy record form, and sent to the sponsor and the sponsor designated CRO. If the pregnancy results meet the SAEs criteria (including spontaneous abortion, stillbirth, or any congenital malformations, etc.), the investigator should report it according to the SAEs reporting process.

9.1.4 SAFETY MONITORING

The investigator and/or designated on-site personnel are responsible for monitoring the safety of all participants and notifying the sponsor when unexpected issues occur. DSMB will conduct independent and continuous monitoring of the safety data of the study vaccine and judge the results.

9.1.5 SAE/SUSAR/PREGNANCY EVENT REPORT

9.1.5.1 Requirement of immediate report by investigators to the sponsor

The following is a list of events that investigators must report to the sponsor within 24 hours of being notified. These events do not necessarily need to be related to the study vaccine:

- SAEs;
- AESI;
- Pregnancy.

For these events, investigators must report new significant follow-up information to the sponsor immediately (that is, within 24 hours after getting the information). New significant information includes the following:

- New signs or symptoms, or changes in diagnosis;
- Important new diagnostic test results;
- New information that may lead to a change in causality assessment;
- Changes in the outcome of the event, including recovered events;
- Other important descriptive information about the clinical course of the event.

All SAEs should also be filled in the eCRF form at the same time, and the information in the SAEs report form must be consistent with the event data recorded in the eCRF.

9.1.5.2 Regulatory Reporting Requirements for SAEs

Prompt notification by the investigator to the sponsor of an SAE is essential so that legal obligations and ethical responsibilities towards the safety of participants and the safety of a study vaccination under clinical investigation are met.

The sponsor has a legal responsibility to notify both the local regulatory authority and other regulatory agencies about the safety of a study vaccination under clinical investigation. The sponsor will comply with country-specific regulatory requirements relating to safety reporting

to the regulatory authority, institutional review boards (IRBs)/ethics committees (ECs), and investigators.

Investigator safety reports must be prepared for suspected unexpected serious adverse reactions (SUSARs) according to local regulatory requirements and sponsor policy and forwarded to investigators as necessary.

An investigator who receives an investigator safety report describing an SAE or other specific safety information (e.g., summary or listing of SAEs) from the sponsor will review and then file it along with the investigator's brochure and will notify the IRB/EC, if appropriate according to local requirements.

9.1.5.3 Pregnancy report

When female participants or female partners of male participants become pregnant during the study period (collection period is the same as SAEs), the "pregnancy report form" should be filled out within the same time limit as SAEs report to the sponsor (or CRO appointed by the sponsor).

Pregnancy itself is not considered as an AE. If spontaneous abortion or artificial abortion, birth defects or congenital abnormalities of newborns, deformities and abnormalities of stillbirths, severe complications of mothers and newborns, and etc. occur during pregnancy, all of them should be recorded and reported as SAEs.

During the study period, female participants of childbearing potential or the female partners of male participants should immediately notify the investigator once they become pregnant. The investigator should make recommendations to the participants, discuss the risks of continuing pregnancy and the possible impact on the fetus. Male participants do not need to withdraw from the study, but their female partners need to be monitored. The follow-up time for pregnancy events lasts at least until the pregnancy outcome or 12 months after the birth of the newborn.

Note: Female participants of childbearing potential or female partners of male participants have the right to know the actual grouping information after unblinding because of pregnancy.

9.2 IMMUNOGENICITY ASSESSMENT

9.2.1 POPULATION AND TIME OF IMMUNOGENICITY ASSESSMENT

For specific sampling points, please refer to the description in section 8.1.1.

9.2.2 ASSESSMENT INDICATORS

Primary assessment indicators:

Cohort 1

- GMT of nAb against Delta variant on D28.
- GMT of nAb against Omicron variant on D28.

Cohort 2

- GMT of nAb against Delta variant on D28.

<u>Secondary assessment indicators:</u>

Cohort 1

- GMT of nAb against Delta variant on D180.
- GMT of nAb against Omicron variant on D180.
- Number of IFN-γ positive (characterizing Th1) and IL-4 positive (characterizing Th2) T cell subsets on D28.
- Seroresponse of nAb (defined as a change from below the low limit of quantitation [LLOQ] to equal to or above LLOQ, or a ≥4-fold rise if baseline is equal to or above LLOQ in nAb to Delta variant from D0) rates on D28.
- Seroresponse of nAb (defined as a change from below LLOQ to equal to or above LLOQ, or a ≥4-fold rise if baseline is equal to or above LLOQ in nAb to Omicron variant from D0) rates on D28.

Cohort 2

- GMT of nAb against Omicron variant on D28.
- GMT of nAb against Delta variant on D180.
- GMT of nAb against Omicron variant on D180.
- Number of IFN-γ positive (characterizing Th1) and IL-4 positive (characterizing Th2) T cell subsets on D28.
- Seroresponse of nAb (defined as a change from below the low limit of quantitation [LLOQ] to equal to or above LLOQ, or a ≥4-fold rise if baseline is equal to or above LLOQ in nAb to Delta variant from D0) rates on D28.
- Seroresponse of nAb (defined as a change from below LLOQ to equal to or above LLOQ, or a ≥4-fold rise if baseline is equal to or above LLOQ in nAb to Omicron variant from D0) rates on D28.

9.3 DIAGNOSIS AND TREATMENT OF COVID-19 INFECTION

9.3.1 DEFINITION OF COVID-19 CONFIRMED CASES

According to FDA's diagnosis and treatment guidelines for COVID-19[14], COVID-19 is defined according to the following criteria:

Participants with positive result of SARS-CoV-2 using a virologic test (i.e., a nucleic acid

amplification test of an antigen test) who have the following clinical symptoms or imaging characteristics of COVID-19:

COVID-19 symptoms consistent with those defined by the US FDA harmonized case definition: fever or chills, cough, shortness of breath or difficulty breathing, fatigue, muscle or body aches, headache, new loss of taste or smell, sore throat, congestion or runny nose, nausea or vomiting, diarrhea.

9.3.2 SEVERITY GRADING CRITERIA OF CONFIRMED COVID-19 CASES

Investigators should closely monitor and treat confirmed patients in accordance with the treatment guidelines formulated by FDA.

Investigators can classify cases according to the FDA COVID-19 disease severity[14]. For details, see Table 7.

Table 7 COVID-19 disease severity

Disease severity	Definition
Mild	Symptoms of mild illness with COVID-19 that could include fever, cough, sore throat, malaise, headache, muscle pain, nausea, vomiting, diarrhea, and loss of taste or smell, without shortness of breath or dyspnea<br>No clinical signs indicative of Moderate, Severe, or Critical Severity
Moderate	Symptoms of moderate illness with COVID-19, which could include any symptom of mild illness or shortness of breath with exertion<br>Clinical signs suggestive of moderate illness with COVID-19, such as respiratory rate ⩾20 breaths per minute, heart rate⩾90 beats per minute; with saturation of oxygen (SpO2) > 93% on room air at sea level<br>No clinical signs indicative of Severe or Critical Illness Severity
Severe	Symptoms suggestive of severe systemic illness with COVID-19, which could include any symptom of moderate illness or shortness of breath at rest, or respiratory distress<br>Clinical signs indicative of severe systemic illness with COVID-19, such as respiratory rate⩾30 per minute, heart rate⩾125 per minute, SpO 2 ⩽ 93% on room air at sea level or PaO 2 /FiO 2 < 300<br>No criteria for Critical Severity
Critical	Evidence of critical illness, defined by at least one of the following:<br>- Respiratory failure defined based on resource utilization requiring at least one of the following:<br>Endotracheal intubation and mechanical ventilation, oxygen delivered by high-flow nasal cannula (heated, humidified, oxygen delivered via reinforced nasal cannula at flow rates > 20 L/min with fraction of delivered oxygen⩾0.5), noninvasive positive pressure ventilation, ECMO, or clinical diagnosis of respiratory failure (i.e., clinical need for one of the preceding therapies, but preceding therapies not able to be administered in setting of resource limitation)<br>- Shock (defined by systolic blood pressure<90 mm Hg, or diastolic blood pressure<60 mm Hg or requiring vasopressors)<br>- Multi-organ dysfunction/failure

9.3.3 DISCOVERY OF SUSPICIOUS CASES OF COVID-19

After the study vaccination, all participants will be monitored for symptoms of COVID-19 at each visit. Participants should actively reported to the investigator once they have any

signs/symptoms related to COVID-19.

9.3.4 CONFIRMATION PROCEDURES FOR COVID-19 CASES

Participants with any suspicious symptoms of COVID-19 (see 9.3.1 chapter Definition of CVOID-19 confirmed cases) should receive nasal/nasopharyngeal/throat swab collection as soon as possible (preferably within 72 hours) for RT-PCR test. Two samples of nasal/throat/pharyngeal swab will be collected at the same time, one of which is for the RT-PCR test, the other one is for sequencing in case that the diagnosis of COVID-19 is confirmed. If the RT-PCR test result is positive, the diagnosis of COVID-19 is confirmed. If the test result is negative and the symptom persists, a second sample will be taken at least 24 hours (but not more than 3 days) apart for RT-PCR test. If the second test result is still negative, the sampling will not be repeated; if the second test result is positive, follow-up will be carried out according to the confirmed case of COVID-19. For specific COVID-19 diagnosis procedures, see Figure 3 .

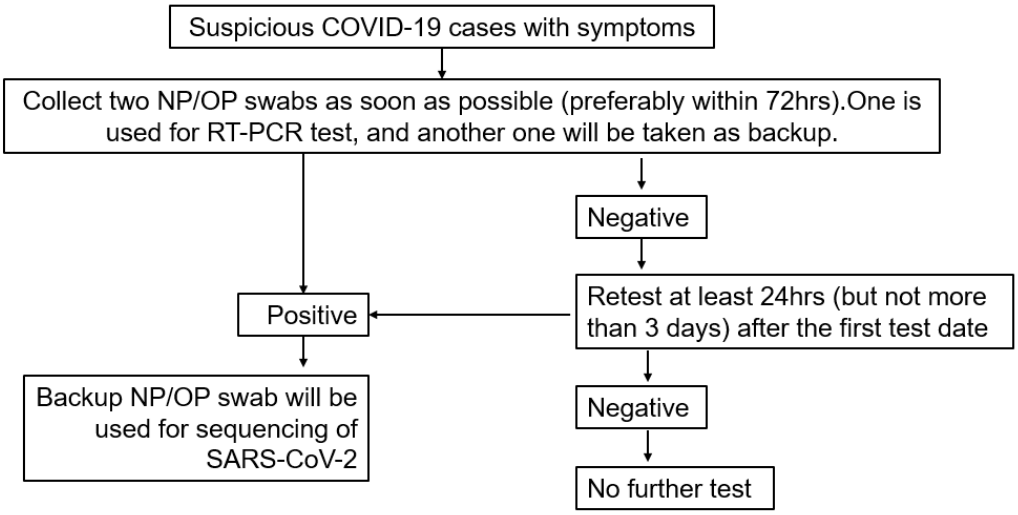

Figure 3 Diagnosis process of COVID-19 cases

9.3.5 FOLLOW-UP OF CONFIRMED COVID-19 CASES

For participants who have been diagnosed with COVID-19, they will be managed and treated in accordance with local policies and regulations. After a participant is diagnosed with COVID-19, the investigator will follow up the participant every 3 to 7 days until at least one negative result of RT-PCR test was received and the symptoms were sustained resolution, which means all COVID-19 related symptoms remain absent or no worse than mild for selected symptoms that may take a longer time to resolve (e.g., cough, fatigue, loss of smell or taste) for a sustained period of 48 hours. Collect treatment status and medical history of the participant to determine whether the participant meet the severe and critical COVID-19 standards (see 9.3.2 Severity grading criteria of confirmed COVID-19 cases).

If a SARS-CoV-2 infection is confirmed, virus will be isolated from the nasal/nasopharyngeal/throat swab and viral sequencing will be used to identify the major SARS-CoV-2 variants.

Record the participant's symptom type, time of occurrence, time of report, time of nucleic acid sample collection, time of test, time of report, time of diagnosis, outcome, etc. to form relevant records.

10 DATA MANAGEMENT

10.1 SOURCE DATA AND THE FILLING AND TRANSFER OF ECRF

The electronic data collection system (EDC) is used for data collection in this study. The data management of this study is the responsibility of the sponsor's data department to ensure the authenticity, integrity, privacy and traceability of clinical trial data.

eCRF: data collection form is designed according to the requirements of protocol, to define the study process, the name of the data form and the data items collected, at the same time, the corresponding eCRF Completion Instructions should be formed, then reviewed by the sponsor to be used by the study Site to fill in the eCRF.

The eCRF data are all derived from the original medical records and filled out by the investigator or investigator designee to ensure the completeness and accuracy of the information. If there are any errors that need to be corrected, the corrections should be made according to the eCRF Completion Instructions, and the EDC system will automatically record the name and date of modification of the data.

After the source data verification (SDV), DM verification, questioning and other processing for the data of the EDC system beyond any doubt, the investigator should conduct electronic signature confirmation before data locking.

10.2 DATABASE PROPOSAL AND DESIGN

The design of eCRF is consistent with the requirements of FDA 21 CFR Part 11 and with the requirements of ICH GCP and GCP (NMPA, 2020) on data collection. The data manager conducts interface tests, including but not limited to: page design, visit period setting, form entry order at visit and order of each data point, etc. As for the new Uniform Resource Locator (URL), the data manager should also test the URL configuration, such as the accuracy of different user browsing permissions, and so on. The database should be established with reference to the Clinical Data Interchange Standards Consortium (CDISC) standards whenever possible.

10.3 ENTRY OF DATA

The investigator should collect participant data in accordance with the requirements of GCP and study protocol, and complete the eCRF accurately, timely, completely, and in accordance with the instructions.

The data is entered into the EDC database by the investigator or a person authorized by the investigator upon completion of the visit. Data entry is carried out in strict accordance with the principle "what you see is what you should record". At the end of data entry, any changes made to the eCRF will be automatically recorded in the system.

10.4 MEDICAL CODING

The coding contents includes, but is not limited to, past medical history, concomitant medications and AEs.

Medical history and AEs will be coded according to the International Medical Dictionary for Regulatory Activities (MedDRA), and concomitant medication will be coded according to the World Health Organization Dictionary of Drugs (WHO DD).

10.5 DATABASE LOCKING AND EXPORTING

The principal investigator, sponsor, statistician, clinical project manager, and DM will decide together to lock the database after all of following items are done: all participants complete the test, the medical records are entered into the system and all data questions are solved, the verifications on the consistency of external data sources show no any errors, the coding report is approved by the sponsor, all problems from database quality control (QC) and data verification meeting (if any) are solved. After all the data is locked, DM will export the data from the system and hand it over to the statistician for statistical analysis. The locked data cannot be edited, and the problems found after the data locking can be corrected in the statistical analysis program after confirmation. If principal investigator, sponsor, statistician, and DM all consider there's solid evidence that it's necessary to unlock after data locking, DM will unlock the data when both the investigator and the sponsor sign the Database Unlock Confirmation Form, then data update is actionable and all updates must be documented. After the update is complete, the locking process should be conducted again.

10.6 ARCHIVE OF STUDY RECORDS

The basic documents for this clinical trial should be maintained for at least 5 years after the approval for marketing of test vaccine by local regulatory authorities. If not used for marketing approval, the documents should be kept for at least 5 years after the end of the clinical trial.

After the period, the study data will be destroyed with the sponsor's written notice.

All documents relating to the test should be stored in strict confidence within the limits of local laws.

11 STATISTICAL ANALYSIS

11.1 HYPOTHESIS

Detailed methodology for summary and statistical analyses of the data collected in this study is outlined here and further detailed in a statistical analysis plan (SAP). The SAP may modify what is outlined in the protocol where appropriate; however, any major modifications of the primary endpoint definitions or their analyses will also be reflected in a protocol amendment.

Let

$GMTC1_{Delta}$= GMT of nAb against Delta variant on D28 of SCTV01C in Cohort 1;

$GMTE1_{Delta}$= GMT of nAb against Delta variant on D28 of SCTV01E in Cohort 1;

$GMTS1_{Delta}$= GMT of nAb against Delta variant on D28 of Sinopharm inactivated vaccine in Cohort 1;

$GMTC1_{Omicron}$= GMT of nAb against Omicron variant on D28 of SCTV01C in Cohort 1;

$GMTE1_{Omicron}$ = GMT of nAb against Omicron variant on D28 of SCTV01E in Cohort 1;

$GMTS1_{Omicron}$ = GMT of nAb against Omicron (B.1.1.529) variant on D28 of Sinopharm inactivated vaccine in Cohort 1;

$GMTC2_{Delta}$= GMT of nAb against Delta variant on D28 of SCTV01C in Cohort 2;

$GMTE2_{Delta}$= GMT of nAb against Delta variant on D28 of SCTV01E in Cohort 2;

$GMTM2_{Delta}$= GMT of nAb against Delta variant on D28 of mRNA-1273 in Cohort 2.

$GMTC2_{Omicron}$= GMT of nAb against Omicron variant on D28 of SCTV01C in Cohort 2;

$GMTE2_{Omicron}$= GMT of nAb against Omicron variant on D28 of SCTV01E in Cohort 2;

$GMTM2_{Omicron}$= GMT of nAb against Omicron variant on D28 of mRNA-1273 in Cohort 2.

For the primary efficacy objectives, the null hypotheses are:

- **H11**: $GMR11=GMTC1_{Delta}/GMTS1_{Delta} \leq 1$;
- **H12**: $GMR12= GMTE1_{Delta}/GMTS1_{Delta} \leq 1$;
- **H13**: $GMR13=GMTC1_{Omicron}/GMTS1_{Omicron} \leq 1$;
- **H14**: $GMR14=GMTE1_{Omicron}/GMTS1_{Omicron} \leq 1$;
- **H21**: $GMR21= GMTE2_{Delta}/GMTM2_{Delta} \leq 0.67$;
- **H22**: $GMR22=GMTE2_{Omicron}/GMTM2_{Omicron} \leq 0.67$;
- **H23**: $GMR23= GMTC2_{Delta}/GMTM2_{Delta} \leq 0.67$;
- **H24**: $GMR24=GMTC2_{Omicron}/GMTM2_{Omicron} \leq 0.67$;

The estimand framework of primary efficacy objectives is listed in Table 8.

Table 8 Estimand framework of primary objectives

Population	Population aged ≥18 years previously vaccinated with either inactivated or mRNA COVID-19 vaccine or previously diagnosed with COVID-19
Treatment conditions	Test: SCTV01C, SCTVO1E<br>Control: Sinopharm inactivated COVID-19 vaccine, mRNA-1273
Variables	Neutralizing antibody titers against Delta or Omicron variant on D28 after first vaccination
Intercurrent event 1	COVID-19 infection up to D28 after first vaccination. A principal stratum strategy will be used, the participants who is infection of COVID-19 up to D28 after first vaccination are excluded from this estimand.
Intercurrent event 2	Receiving of other drugs or vaccines which will modify the immunity against Delta or Omicron variant without COVID-19 infection up to D28 after first vaccination. A principal stratum strategy will be used, the participants who receive other drugs or vaccines which will modify the immunity against Delta or Omicron variant without COVID-19 infection up to D28 after first vaccination are excluded from this estimand.
Population-level summary	Ratio of geometric means of the neutralizing antibody titers

11.2 MULTIPLICITY

The sequential approach will be applied for the multiple testing, as shown in Figure 4, H11 will firstly be tested with a one-sided type I error of 0.025, H1i, i=2,…,4, H2i,i=1,…,4 will then be sequentially tested with a one-sided type I error of 0.025, i.e., H1i, i=2,…,4, H2i, i=1,…,4 will not be tested unless the previous one has been rejected. The sequential testing method is a closed testing procedure and the family-wise error rate can be controlled.

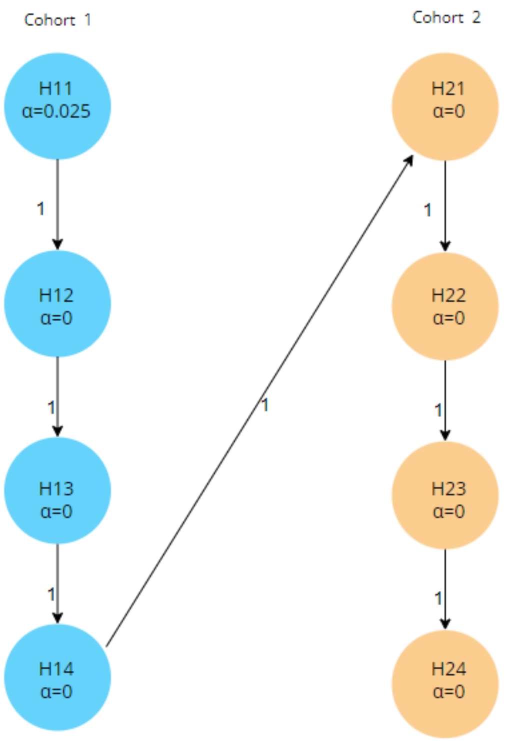

Figure 4 Sequential testing

11.3 SAMPLE SIZE ESTIMATION

Totally 1800 participants aged ≥18 years who were previously vaccinated with either inactivated or mRNA COVID-19 vaccine or previously diagnosed with COVID-19 will be enrolled. 300 participants (100 in SCTV01C Group, 100 in SCTV01E Group, 100 in Sinopharm COVID-19 vaccine Group) in Cohort 1 will have nAb tests. 450 participants (150 in SCTV01C Group, 150 in SCTV01E Group, 150 in mRNA-1273 Group) in Cohort 2 will have nAb tests.

Assumptions for the sample size calculation of **H1i**, i=1,…,4, **H2i**,i=1,…,4 are listed as follows:

- The standard deviation of neutralizing antibody titers under log10 transformation is 0.4;
- The 1-sided type I error is 0.025
- Power is about 90%;
- GMR1i≥1.6, i=1,…,4; GMR2i≥1, i=1,…,4;
- The dropout rate of Cohort 1 during study is about 10%;
- The rate of participants with SARS-CoV-2 infection history and rate of dropout in Cohort 2 is about 20%.

11.4 STATISTICAL POPULATIONS

Full Analysis Set (FAS): All randomized participants who received one dose of investigational

product (IP).

Per-Protocol Set (PPS): All participants in the FAS set who received planned doses of IP per schedule and have no major protocol deviations, as determined and documented by Sponsor prior to DBL and unblinding, that impact critical or key study data.

Safety Set (SS): All randomized participants who received one dose of IP.

Immunogenicity full analysis set (I-FAS): All participants in the FAS who had a valid immunogenicity test result prior to receiving the IP and at least 1 valid result after receiving the IP.

Immunogenicity per-protocol set (I-PPS): All participants in the PPS who had a valid immunogenicity test result prior to receiving the IP and at least 1 valid result after receiving the IP.

Immunogenicity full analysis set (I-FAS1): All participants in the I-FAS who were previously fully vaccinated with 2 doses of Sinopharm inactivated COVID-19 vaccine and with no previous COVID-19 infection history.

Immunogenicity per-protocol set (I-PPS1): All participants in the I-PPS who were previously fully vaccinated with 2 doses of Sinopharm inactivated COVID-19 vaccine and with no previous COVID-19 infection history.

Immunogenicity full analysis set (I-FAS2): All participants in the I-FAS who previously received mRNA-1273 or Comirnaty and with no previous COVID-19 infection history.

Immunogenicity per-protocol set (I-PPS2): All participants in the I-PPS who previously received mRNA-1273 or Comirnaty and with no previous COVID-19 infection history.

11.5 STATISTICAL ANALYSIS METHOD

Once the safety data within 28 days and immunogenicity data on D28+3 for each cohort were acquired, it will be analyzed by unblinded team who are independent to the study operation team and are not directly involved in the study activities. The result will be further used for submission to regulatory authority. The specific analysis time point may be adjusted according to the progress of the trial.

11.5.1 GENERAL PRINCIPLES

The statistical analysis is carried out with the descriptive and pre-specified statistical test method. The analytical procedures will be detailed in the statistical analysis plan (SAP).

American SAS 9.4 or above will be used for statistical analysis.

Descriptive statistics of continuous variables will include mean, standard deviation, median, minimum, and maximum values. The classification variable will be described by number and percentage. The calculation method of percentage will be defined in the SAP.

The expected values, standard errors, and 95% confidence interval (Cl) will be calculated based on the assumed distribution and pre-specified models, as defined in the SAP.

11.5.2 DEMOGRAPHIC AND BASELINE CHARACTERISTICS

The Demographic and Baseline Characteristics, including protocol deviations will be listed. Demographic data and baseline indicators will be analyzed among the FAS. All demographic data (age, sex, race, ethnicity, et al) and baseline variables (physical examination, pregnancy test, history of diseases, history of COVID-19, medication history, interval between time of administration of IP and the last time of COVID-19 vaccine administration/diagnosed with COVID-19, the type of previous COVID-19 vaccinations and serum antibody titer before the administration of IP) are summarized.

For continuous variables, descriptive statistics (the number of participants, mean, standard deviation, minimum, median and maximum values) is used; and for classified variables, the number and percentage are calculated.

11.5.3 EXPOSURE AND COMPLIANCE OF STUDY TREATMENT

The exposure dose and compliance of participants are descriptively summarized, including safety evaluation and immunogenicity-testing compliance.

11.5.4 IMMUNOGENICITY AND EXPLORATORY ANALYSIS

The Immunogenicity analysis will be based on I-FAS, I-PPS, I-FAS1, I-PPS1, I-FAS2, I-PPS2. The main analysis will be based on I-FAS1 and I-FAS2.

The GMT of neutralizing antibody for each group with corresponding 2-sided 95% CI will be estimated at each post-baseline time point using an analysis of covariance. The comparison of GMT of neutralizing antibody between the treatment groups at each post-baseline time point will also be provided using an analysis of covariance.

The 95% CI of seroresponse using the Clopper-Pearson method will be provided. Cochran-Mantel-Haenszel method will be used for comparison of the seroresponse between the treatment groups.

The change in the number of IFN-γ positive (characterizing Th1) and IL-4 positive (characterizing Th2) T cell subsets at each post-baseline time point will be statistically described, and the nonparametric test will be applied for the statistical comparison between groups. Detailed statistical analysis methods are described in the SAP for further reference.

11.5.5 SAFETY ANALYSIS

Safety analysis will be based on SS.

AEs and SAEs are encoded based on the *Medical Dictionary for Regulatory Activities*

(MedDRA), and also based the document the classified statistics was made according to the system organ class (SOC) and preferred term (PT). In this trial, the treatment emergent adverse events (TEAEs) are summarized, and the adverse medical conditions occurring before the study vaccination are listed. Unless otherwise specified, the adverse events as described below are TEAEs.

The incidence of AEs, SAEs and AESIs, the number and percentage of participants with AEs, SAEs and AESIs in each group will be summarized respectively. The 2-sided 95% CI will be also provided for the percentage of participants with any solicited AE for each treatment group using the Clopper-Pearson method. The adverse events related to the study vaccine, SAEs and AESIs will be listed.

12 MANAGEMENT OF CLINICAL TRIAL

12.1 DECLARATION

This study will be conducted in accordance with ICH GCP, the Declaration of Helsinki, the SOPs of the sponsor and its agents (e.g., CRO) and all applicable regulations.

12.2 ETHICS

The clinical trial protocol, ICF, Investigator's Brochure (IB) and other relevant documents should be submitted to the appropriate Ethics Committee (ERC) for approval prior to the start of the test. The test shall not be carried out in any form before the sponsor obtain written consent or approval from the appropriate EC. Any amendments to relevant documents, such as informed consent, to the clinical trial protocol must be implemented with the approval of the ERC.

The investigator and the personnel involved in the study should be familiar with the protocol and be able to prepare measures in advance, such as measures and reports in the event of SUSAR.

In the course of a clinical trial, if any SAEs or SUSAR related to clinical trial safety occurs that may affect the safety of the participant or the conduct of the study, the investigator should report the ERC as required by regulations.

12.3 INFORMED CONSENT

Participants must give informed consent to this study before receiving treatment in order to protect their legal rights and interests. It is the responsibility of the principal investigator or investigator of the clinical trial to fully and comprehensively introduce the purpose, methods, reasonable expected benefits, possible adverse reactions and risks of the study to the participants. At the same time, participants should be informed that the participation to the

clinical trial is voluntary and that they have the right to withdraw from the test at any time without prejudice to their personal interests. ICFs signed by participants own or the legal representative must be obtained before any clinical trial related procedures are performed. The ICF is prepared in two copies, one for the participant and one for the filing.

Prior to obtaining informed consent, the investigator or designee should provide the participant with sufficient time and opportunity to ask about the details of the study and to decide whether to participate in the study. The process of informed consent should be documented in the progress notes on the day of screening visit.

The investigator is responsible for the informed consent process. If any information is obtained during the test relating to the participant's willingness to continue the test, the written informed consent must be updated and given to the participant to confirm the willingness to continue to participate. Ethical approval is required before the revised informed consent is provided to the participant.

By signing the informed consent, participants should also agree to allow the sponsor, the drug approval administration, the auditor and/or the sponsor's authorized clinical trial monitor to review the obtained raw data related to the clinical trial in compliance with the confidentiality statement.

The investigator should use the latest version of ICF and other information provided to participants as agreed by the ERC. If any information is obtained during the test relating to the participant's willingness to continue the test, the written ICF must be updated and given to the participant to confirm the willingness to continue to participate. Ethical approval is required before the revised ICF is provided to the participant. Participants may withdraw unconditionally from the study at any time during the study, and participants will not be penalized for the withdrawal.

12.4 REVISION OF CLINICAL TRIAL PROTOCOL

During the course of the study, the sponsor should communicate with the investigator and make modifications to the protocol, which should be implemented only after the approval of the ERC. Any changes to the protocol, whether material or non-material, are required to be in writing. Approval from the ERCs of all study Sites is required for substantive protocol changes that will clearly affect the safety of participants, the scope of the study, or the scientific quality of the study. For the safety of all participants in the study, the above requirements shall not hinder the investigator or sponsor from taking any urgent actions. If the investigator deems that an immediate change of protocol is necessary for safety reasons, the sponsor's designated institution must be notified in time and the study Site ERC should be notified in accordance

with the policies made by the ERC that approves the study, as well as local regulations and policies. Changes that only affect the management of the study do not require substantial protocol revision or ERC approval, but such changes must be notified to ERC. In these cases, the sponsor will send an official letter to the ERC detailing the changes.

12.5 PROTOCOL DEVIATION

The investigator should conduct the study in accordance with a protocol agreed by the sponsor and the regulatory authority (if necessary) and approved by the ERC.

During the test, the investigator should not deviate from the protocol unless urgent measures are taken to eliminate the immediate risk to participants. In the event of other unexpected circumstances that require deviation from the procedures specified in the protocol, the investigator should consult with the medical monitor (and the ERC, if necessary) to determine appropriate actions.

The study Site should record all protocol deviations in the participant's original data, including but not limited to the time of occurrence of protocol deviations, time of discovery, description of events, and measures taken, etc. In the event of a serious protocol deviation, the center should notify the medical monitor, Clinical Research Associate, or ERC promptly.

12.6 MONITORING

The sponsor and/or its agents (e.g., CRO) conduct Clinical Research Associating of the study. The Clinical Research Associate should follow the appropriate SOPs. The monitor should maintain regular communication with the investigator and sponsor.

Before the clinical trial: the monitor should confirm that the investigator has sufficient qualifications and resources to complete the test, that the clinical trial institution has the appropriate conditions to complete the test, including staffing and training, and that the laboratory is well equipped in good working order and is well-qualified for various tests related to the test. At the same time, the monitor should discuss with the investigator the specific items required as the original data and determine the nature and location of all the original data to ensure that the sponsor or investigator knows the source of the original data used to complete the eCRF.

During the clinical trial: the monitor will regularly visit the clinical site (online visit is allowed) to review the protocol compliance, data integrity, accuracy and consistency, as well as compliance with ICH GCP and relevant regulations. Depending on the risk assessment, remote centralized monitoring may be considered as a replacement or supplement to on-site

monitoring. As necessary, the monitor will also provide clarification and additional training to help resolve on-site issues identified during the monitoring visit.

During the study period, the investigator should agree to direct access to all relevant documents by the monitor and ensure that he/she and relevant study staff meet with the monitor regularly to discuss the findings from the visit and any related issues.

12.7 QUALITY ASSURANCE AND AUDIT

During the study, the sponsor or sponsor's representative will conduct quality assurance audits of the study Site, databases and related documents. At the same time, the relevant regulatory authorities can also inspect the study Site, databases and relevant documents at their own discretion. The purpose is to determine whether the recording, analysis and reporting of these activities and data comply with the study protocol, GCP, ICH guidelines and any relevant regulatory requirements. During the process of audit or inspection, the investigator should support the audit or inspection and allow the auditor or inspector direct access to original data or documents, including all medical records, documents and letters related to the study, and informed consent documents for the clinical trial, etc.

12.8 INTELLECTUAL PROPERTY

All information obtained from the sponsor is the sponsor's intellectual property and thus must be kept strictly confidential by the investigator and all other relevant personnel and shall not be disclosed to third parties without the prior consent of the study sponsor.

12.9 PARTICIPANTS' PRIVACY

Study staff must ensure that the privacy of participants is maintained. For all submissions to the sponsor, participants shall be identified only by the participant code and name abbreviation, but not by the participant name or admission number. The investigator must keep the name, address and other private information of participants in the clinical trial in strict confidence and shall not submit it to the sponsor.

12.10 MONITORING BOARD

12.10.1 DATA AND SAFETY MONITORING BOARD (DSMB)

This study were organized by the sponsor to establish a DSMB to periodically evaluate the progress of clinical trial, and to advise the sponsor about whether to continue, modify or discontinue the ongoing clinical trial based on the data results.

DSMB members include experts in the clinical research field of vaccine, biostatisticians and epidemiologists, etc. DSMB should have prior knowledge of the clinical trial protocol, develop

and sign the DSMB regulations for this study. The primary task of DSMB are to review the safety data of participants reported after the study vaccination for the participants' safety and interests. At the same time, DSMB also monitors the entire process of the clinical trial, including protocol compliance, recruitment status, and drop-out rate of participants, to ensure the validity and credibility of the test.

For more information, see the DSMB charter and carry out the work as required by the DSMB charter.

13 FINANCE AND INSURANCE

The sponsor will provide insurance that meets regulatory and legal requirements. The sponsor has purchased liability insurance for this clinical trial and the liability policy complies with local laws and requirements. The liability insurance policy will be submitted to the ERC, IRB or regulatory authority as required by the corresponding country.

14 PUBLISHING AND DATA SHARING POLICIES

The author should be identified before the writing of the manuscript. Unless the consent of Sinocelltech Ltd. is obtained, no individual writing is allowed to be published before the final report of the study is completed. With respect to the manuscript and publication, the decision of Sinocelltech Ltd. has the right of final decision.

15 APPENDICES

15.1 APPENDIX I: TOXICITY RATING SCALE FOR HEALTHY ADULT AND ADOLESCENT VOLUNTEERS IN PREVENTIVE VACCINE CLINICAL TRIAL - FDA STANDARD

Table 9 Scale of clinical abnormalities

Local reaction of the injected product	Mild (grade 1)	Moderate (grade 2)	severe (grade 3)	Potentially life-threatening (grade 4)
**Pain**	Does not interfere with activity	Repeated use of non-narcotic pain reliever > 24 hours or interferes with activity	Any use of narcotic pain reliever or prevents daily activity	Emergency care or hospitalization required
**Tenderness**	Mild discomfort to touch	Discomfort with movement	Significant discomfort at rest	ER visit or hospitalization
**Erythema/redness***	2.5-5 cm	5.1-10 cm	> 10 cm	Necrotic or exfoliative dermatitis
**Induration/swelling****	2.5 – 5 cm and does not interfere with activity	5.1 – 10 cm or interferes with activity	> 10 cm or prevents daily activity	Necrosis

* In addition to grading of the measured local reactions at the maximum diameter, diameter changes should also be recorded;
** Induration or swelling should be assessed and graded using a functional scale and actual measurements.

Table 10 Scale of vital signs

Vital signs*	Mild (grade 1)	Moderate (grade 2)	Severe (grade 3)	Potentially life-threatening (grade 4)
**Fever °C**<br>**°F****	38.0 – 38.4<br>100.4 – 101.1	38.5 – 38.9<br>101.2 – 102.0	39.0 – 40<br>102.1 – 104	> 40<br>> 104
**Tachycardia (bpm)**	101 – 115	116 – 130	> 130	Emergency care or hospitalization required due to arrhythmias
**Bradycardia (bpm)*****	50 – 54	45 – 49	< 45	Emergency care or hospitalization required due to arrhythmias
**High blood pressure (systolic) mmHg**	141 – 150	151 – 155	> 155	Emergency care or hospitalization resulted from malignant hypertension
**High blood pressure (diastolic) mmHg*****	91 – 95	96 – 100	> 100	Emergency care or hospitalization resulted from malignant hypertension
**Low blood pressure (systolic)**	85 – 89	80 – 84	< 80	Emergency care or hospitalization

**mmHg**				resulted from hypotensive shock
**Respiration rate (times per minute)**	17 – 20	21 – 25	> 25	Endotracheal intubation is required

* All vital signs should be measured for participants after rest;
** Oral temperature, with no hot or cold drinks or smoking before testing;
*** Resting heart rate is between 60 and 100 beats per minute. For some healthy participants, such as certain athletes, the characteristics of bradycardia should be judged clinically.

Table 11 Scale of adverse events

Systemic reaction	Mild (grade 1)	Moderate (grade 2)	Severe (grade 3)	Potentially life-threatening (grade 4)
**Nausea and vomiting**	No interference with activity or 1 – 2 episodes/24 hours	Some interference with activity or > 2 episodes/24 hours	Prevents daily activity, requires outpatient IV hydration	ER visit or hospitalization for hypotensive shock
**Diarrhea**	2 – 3 loose stools or < 400 gms/24 hours	4 – 5 stools or 400 – 800 gms/24 hours	6 or more watery stools or > 800gms/24 hours or requires outpatient IV hydration	ER visit or hospitalization
**Headache**	No interference with activity	Repeated use of non-narcotic pain reliever > 24 hours or some interference with activity	Significant; any use of narcotic pain reliever or prevents daily activity	ER visit or hospitalization
**Fatigue**	No interference with activity	Some interference with activity	Significant; prevents daily activity	ER visit or hospitalization
**Myalgia**	No interference with activity	Some interference with activity	Significant; prevents daily activity	ER visit or hospitalization
**Other diseases or clinical adverse events (as defined in applicable regulations)**	No interference with activity	Some interference with activity not requiring medical intervention	Prevents daily activity and requires medical intervention	ER visit or hospitalization

Table 12 Abnormal Results of Laboratory Examinations

Serum*	Mild (grade 1)	Moderate (grade 2)	Severe (grade 3)	Potentially life-threatening (grade 4)**
**Sodium - hyponatremia mEq/L**	132 – 134	130 – 131	125 – 129	< 125
**Sodium - hypernatremia mEq/L**	144 – 145	146 – 147	148 – 150	> 150
**Potassium - hyperkalemia mEq/L**	5.1 – 5.2	5.3 – 5.4	5.5 – 5.6	> 5.6
**Potassium - hypokalemia mEq/L**	3.5 – 3.6	3.3 – 3.4	3.1 – 3.2	< 3.1
**Glucose - hypoglycemia**	65 – 69	55 – 64	45 – 54	< 45

Serum*	Mild (grade 1)	Moderate (grade 2)	Severe (grade 3)	Potentially life-threatening (grade 4)**
**mg/dL**				
**Glucose - hyperglycemia**<br>**Fasting mg/dL**<br>**Random mg/dL**	100 – 110<br>110 – 125	111 – 125<br>126 – 200	>125<br>>200	Insulin treatment required or hyperosmolar coma
**BUN mg/dL**	23 – 26	27 – 31	> 31	Hemodialysis required
**Serum creatinine mg/dL**	1.5 – 1.7	1.8 – 2.0	2.1 – 2.5	> 2.5 or hemodialysis required
**Calcium - hypocalcemia mg/dL**	8.0 – 8.4	7.5 – 7.9	7.0 – 7.4	< 7.0
**Calcium - hypercalcemia mg/dL**	10.5 – 11.0	11.1 – 11.5	11.6 – 12.0	> 12.0
**Magnesium - hypomagnesemia mg/dL**	1.3 – 1.5	1.1 – 1.2	0.9 – 1.0	< 0.9
**Phosphorus - hypophosphatemia mg/dL**	2.3 – 2.5	2.0 – 2.2	1.6 – 1.9	< 1.6
**CPK mg/dL**	1.25 – 1.5×ULN***	1.6 – 3.0×ULN	3.1 –10×ULN	> 10×ULN
**Albumin – hypoalbuminemia g/dL**	2.8 – 3.1	2.5 – 2.7	< 2.5	--
**Total protein – hypoproteinemia g/dL**	5.5 – 6.0	5.0 – 5.4	< 5.0	--
**Alkaline phosphatase increased**	1.1 – 2.0×ULN	2.1 – 3.0×ULN	3.1 – 10×ULN	> 10×ULN
**Liver function test - ALT and AST increased**	1.1 – 2.5×ULN	2.6–5.0×ULN	5.1 – 10×ULN	> 10×ULN
**Bilirubin increased - with increased liver function indicators**	1.1 – 1.25×ULN	1.26 – 1.5×ULN	1.51 – 1.75×ULN	> 1.75×ULN
**Bilirubin increased - normal liver function**	1.1 – 1.5×ULN	1.6 – 2.0×ULN	2.0 – 3.0×ULN	> 3.0×ULN
**Cholesterol**	201 – 210	211 – 225	> 226	--
**Trypsin, amylase and lipase**	1.1 – 1.5×ULN	1.6 – 2.0×ULN	2.1 – 5.0×ULN	> 5.0×ULN

* The laboratory testing values provided in the table as a guide are determined based on the normal values of the medical facilities. Therefore, a specified range of normal reference values should be provided to prove its applicability.

** Clinical signs and symptoms associated with the abnormal results of laboratory examinations may result in a potentially life-threatening (grade 4) presentation of abnormalities. For example, if the participant had a new seizure attack associated with low sodium level, a sodium level as low as grade 3 (125-129mE/L) will also be recorded as a grade 4 hyponatremia event.

*** "ULN" represents the upper limit of the normal range.

Table 13 Hematological Abnormality

Hematology*	Mild (grade 1)	Moderate (grade 2)	Severe (grade 3)	Potentially life-threatening (grade 4)
**Hemoglobin (female) gm/dL**	11.0 – 12.0	9.5 – 10.9	8.0 – 9.4	< 8.0
**Changes in hemoglobin (female)**	Increase - 1.5	1.6 – 2.0	2.1 – 5.0	> 5.0

**compared to baseline gm/dL**				
**Hemoglobin (male) gm/dL**	12.5 – 13.5	10.5 – 12.4	8.5 – 10.4	< 8.5
**Changes in hemoglobin (male) compared to baseline gm/dL**	Increase - 1.5	1.6 – 2.0	2.1 – 5.0	> 5.0
**Leukocytes increased - cell/mm^3**	10,800 – 15,000	15,001 – 20,000	20,001 – 25, 000	> 25,000
**Leukocytes decreased - cell/mm^3**	2,500 – 3,500	1,500 – 2,499	1,000 – 1,499	< 1,000
**Lymphocyte decreased - cell/mm^3**	750 – 1,000	500 – 749	250 – 499	< 250
**Neutrophil decreased - cell/mm^3**	1,500 – 2,000	1,000 – 1,499	500 – 999	< 500
**Eosnophils decreased - cell/mm^3**	650 – 1500	1501 - 5000	> 5000	Eosinophilia
**Platelet count decreased - cell/mm^3**	125,000 – 140,000	100,000 – 124,000	25,000 – 99,000	< 25,000
**Prolonged coagulation time (PT)**	1.0 – 1.10×ULN**	1.11 – 1.20×ULN	1.21 – 1.25×ULN	> 1.25×ULN
**Prolonged partial thromboplastin time (PTT)**	1.0 – 1.2×ULN	1.21 – 1.4×ULN	1.41 – 1.5×ULN	> 1.5×ULN
**Fibrinogen increased mg/dL**	400 – 500	501 – 600	> 600	--
**Fibrinogen decreased mg/dL**	150 – 200	125 – 149	100 – 124	< 100 or related to the total amount of blooding, or occurance of DIC

* The laboratory testing values provided in the table as a guide are determined based on the normal values of the medical facilities. Therefore, a specified range of normal reference values should be provided to prove its applicability.
** "ULN" represents the upper limit of the normal range.

Table 14 Abnormalities of Urine Routine Examination

**Urine***	**Mild (grade 1)**	**Moderate (grade 2)**	**Severe (grade 3)**	**Potentially life-threatening (grade 4)**
**Protein**	Micro amount	1+	2+	Hospitalization or dialysis treatment
**Glucose**	Micro amount	1+	2+	Hospitalization due to hyperglycemia
**Red blood cells (microscopic examination) Number of red blood cells per high power field (rbc/hpf)**	1-10	11-50	> 50 and/or whole blood	Hospitalization or Packed Red Blood Cells (PRBC) infusion required

* The laboratory testing values provided in the table as a guide are determined based on the normal values of the medical facilities. Therefore, a specified range of normal reference values should be provided to prove its applicability.

16 REFERENCES

1. *World Health Organization*. Available from: https://covid19.who.int/.
2. *National Health Commission of the People's Republic of China. Diagnosis and treatment plan for COVID-19 (trial version 8).* Chin J Clin Infect Dis., 2020. **13**(5).
3. *Sinocelltech Ltd. Bivalent SARS-CoV-2 Trimeric Spike Protein Vaccine (SCTV01C) Investigator's Brochure.*
4. *National Medical Product Administration. General Principles of Technical Review for Preclinical Safety Assessment of Prophylactic Biologics.*
5. Karim, S.S.A., *Vaccines and SARS-CoV-2 variants: the urgent need for a correlate of protection.* Lancet, 2021. **397**(10281): p. 1263-1264.
6. Wang, P., et al., *Antibody resistance of SARS-CoV-2 variants B.1.351 and B.1.1.7.* Nature, 2021. **593**(7857): p. 130-135.
7. Abu-Raddad, L.J., et al., *Effectiveness of the BNT162b2 Covid-19 Vaccine against the B.1.1.7 and B.1.351 Variants.* N Engl J Med, 2021. **385**(2): p. 187-189.
8. Shinde, V., et al., *Efficacy of NVX-CoV2373 Covid-19 Vaccine against the B.1.351 Variant.* N Engl J Med, 2021. **384**(20): p. 1899-1909.
9. Madhi, S.A., et al., *Efficacy of the ChAdOx1 nCoV-19 Covid-19 Vaccine against the B.1.351 Variant.* N Engl J Med, 2021. **384**(20): p. 1885-1898.
10. *A study to evaluate the immunogenicity and safety of mRNA-1273.211 vaccine for COVID-19 variants. ClinicalTrials.gov (NCT04927065).*
11. *The Phase I Clinical trial of booster vaccination of adenovirus type-5 vectored COVID-19 vaccine. ClinicalTrials.gov (NCT04568811).*
12. *Immunogenicity and safety of a third dose, and immune persistence of CoronaVac vaccine in healthy adults aged 18-59 years: interim results from a double-blind, randomized, placebo-controlled phase 2 clinical trial. ClinicalTrials.gov (NCT04979949).*
13. Borobia, A.M., et al., *Immunogenicity and reactogenicity of BNT162b2 booster in ChAdOx1-S-primed participants (CombiVacS): a multicentre, open-label, randomised, controlled, phase 2 trial.* Lancet, 2021. **398**(10295): p. 121-130.
14. *Food and Drug Administration. COVID-19: Developing Drugs and Biological Products for Treatment or Prevention Guidance for Industry, 2021.*

Clinical Trial Protocol

A randomized, double-blind, and positive-controlled Phase III clinical trial to evaluate the immunogenicity and safety of SCTV01C (A Bivalent SARS-CoV-2 Trimeric Spike Protein Vaccine) and SCTV01E (A COVID-19 Alpha/Beta/Delta/Omicron Variants S-Trimer Vaccine) in population aged ≥18 years previously vaccinated with either inactivated or mRNA COVID-19 vaccine or previously diagnosed with COVID-19

**Protocol No.:**	SCTV01C-E-01-UAE-1
**Protocol Version No.:**	Version 4.0
**Version date:**	September 22, 2022
**Sponsor:**	Sinocelltech Ltd.

Confidentiality Statement

All information in this protocol constitutes proprietary property of Sinocelltech Ltd. Therefore, it is only provided to investigators, co-investigators, ethics committees, regulatory authorities, and other relevant medical institutions for review. Without the written approval of Sinocelltech Ltd., it is strictly forbidden to inform any third party unrelated to this study of any information herein, except where required by applicable law. In the event of any actual or suspected breach of this obligation, Sinocelltech Ltd. must be promptly notified.

Protocol Signature Page

I agree to:

- Conduct this study in strict accordance with the protocol, quality management practices of clinical drug trials and relevant laws and regulations.
- Keep all materials and information provided by Sinocelltech Ltd. in accordance with confidentiality requirements and indicate that they are confidential when submitted to Institution Review Committee or Independent Ethics Committee.

I have read the protocol in full and agree with all requirements.

____________________	____________________	____________________
Director of Sponsor	Signature	Date

Protocol Signature Page

I agree to:

- Follow this study in strict accordance with the protocol, quality management practices of clinical drug trials and relevant laws and regulations.
- Keep all materials and information provided by Sinocelltech Ltd. in accordance with confidentiality requirements and indicate that they are confidential when submitted to Institution Review Committee or Independent Ethics Committee.

I have read the protocol in full and agree with all requirements.

______________________	______________________	______________________
Statistician	Signature	Date

Protocol Signature Page

I agree to:

- Conduct this study in strict accordance with the protocol, quality management practices of clinical drug trials and relevant laws and regulations.
- Keep all materials and information provided by Sinocelltech Ltd. in accordance with confidentiality requirements and indicate that they are confidential when submitted to Institution Review Committee or Independent Ethics Committee.

I have read the protocol in full and agree with all requirements.

______________________	______________________	______________________
Principal Investigator	Signature	Date

PROTOCOL SYNOPSIS

**Protocol No.**	SCTV01C-E-01-UAE-1
**Protocol Title**	A randomized, double-blind, and positive-controlled Phase III clinical trial to evaluate the immunogenicity and safety of SCTV01C (A Bivalent SARS-CoV-2 Trimeric Spike Protein Vaccine) and SCTV01E (A COVID-19 Alpha/Beta/Delta/Omicron Variants S-Trimer Vaccine) in population aged ≥18 years previously vaccinated with either inactivated or mRNA COVID-19 vaccine or previously diagnosed with COVID-19
**Version No.**	Version 4.0
**Version Date**	September 22, 2022
**Sponsor**	Sinocelltech Ltd.
**Study Phase**	Phase III
**Indication**	Prevention of COVID-19 (COVID-19 in this protocol refers to COVID-19 patients diagnosed according to the US FDA standards)
**Target Population**	Individuals aged ≥18 years who were previously vaccinated with either inactivated or mRNA COVID-19 vaccine or previously diagnosed with COVID-19
**Study Objectives**	**Primary Objective:**<br>• To evaluate the immunogenicity of SCTV01C;<br>• To evaluate the immunogenicity of SCTV01E.<br>**Secondary Objective:**<br>• To evaluate the cellular immune response of SCTV01C;<br>• To evaluate the cellular immune response of SCTV01E;<br>• To evaluate the safety of SCTV01C within 180 days after the vaccination;

	• To evaluate the safety of SCTV01E within 180 days after the vaccination.
**Study endpoint**	**Primary endpoints**<br>**Cohort 1**<br>***Immunogenicity***<br>• Geometric mean titer (GMT)of neutralizing antibodies (nAb) against Delta variant on D28;<br>• GMT of nAb against Omicron BA.1 (B.1.1.529) variant on D28.<br>**Cohort 2**<br>***Immunogenicity***<br>• GMT of nAb against Omicron BA.1 variant on D28.<br>• GMT of nAb against Delta variant on D28.<br>**Secondary endpoints:**<br>**Cohort 1**<br>***Immunogenicity***<br>• GMT of nAb against Delta variant on D180;<br>• GMT of nAb against Omicron BA.1 variant on D180;<br>• GMT of nAb against Omicron BA.5 variant on D28;<br>• Number of IFN-γ positive (characterizing Th1) and IL-4 positive (characterizing Th2) T cell subsets on D28;<br>• Seroresponse of nAb (defined as a change from below the low limit of quantitation [LLOQ] to equal to or above LLOQ, or a ≥4-fold rise if baseline is equal to or above LLOQ in nAb to Delta variant from D0) rates on D28;<br>• Seroresponse of nAb (defined as a change from below LLOQ to equal to or above LLOQ, or a ≥4-fold rise if baseline is equal to or above LLOQ in nAb to Omicron variant from D0) rates on D28;<br>***Safety***<br>• Incidence and severity of solicited AEs of SCTV01C from D0 to D7.<br>• Incidence and severity of all unsolicited AEs of SCTV01C from D0 to D28;

	• Incidence and severity of SAEs and AESIs of SCTV01C within 180 days;<br>• Incidence and severity of solicited AEs of SCTV01E from D0 to D7;<br>• Incidence and severity of all unsolicited AEs of SCTV01E from D0 to D28;<br>• Incidence and severity of SAEs and AESIs of SCTV01E within 180 days.<br>**Cohort 2**<br>***Immunogenicity***<br>• GMT of nAb against Delta variant on D180;<br>• GMT of nAb against Omicron BA.1 variant on D180;<br>• GMT of nAb against Omicron BA.5 variant on D28;<br>• Number of IFN-γ positive (characterizing Th1) and IL-4 positive (characterizing Th2) T cell subsets on D28;<br>• Seroresponse of nAb (defined as a change from below the low limit of quantitation [LLOQ] to equal to or above LLOQ, or a ≥4-fold rise if baseline is equal to or above LLOQ in nAb to Delta variant from D0) rates on D28;<br>• Seroresponse of nAb (defined as a change from below LLOQ to equal to or above LLOQ, or a ≥4-fold rise if baseline is equal to or above LLOQ in nAb to Omicron variant from D0) rates on D28.<br>***Safety***<br>• Incidence and severity of solicited AEs of SCTV01C from D0 to D7;<br>• Incidence and severity of all unsolicited AEs of SCTV01C from D0 to D28;<br>• Incidence and severity of SAEs and AESIs of SCTV01C within 180 days;<br>• Incidence and severity of solicited AEs of SCTV01E from D0 to D7;<br>• Incidence and severity of all unsolicited AEs of SCTV01E from D0 to D28;<br>• Incidence and severity of SAEs and AESIs of SCTV01E within 180 days.

**Study Design**	Although there is no clinical data for SCTV01E so far, SCT had initiated three clinical Phase I/II trials for SCTV01C to evaluate the safety and immunogenicity, which can be instructive and meaningful for SCTV01E clinical study consideration because of the same manufacturing processes, extremely similar molecular characteristics and clinical dosing between SCTV01E and SCTV01C. The SCTV01C trials will provide supportive safety and immunogenicity clinical data prior to the start of SCTV01E trials. The details of these trials are summarized in investigator's brochure.<br>SCTV01E, the quadrivalent vaccine, will be tested in this Phase III immunogenicity study based on the clinical data on safety, reactogenicity, and immunogenicity generated with the bivalent vaccine (SCTV01C) similarity in manufacturing process for four TM (trimeric drug substance) components of the quadrivalent product compared to the bivalent product; similarity in construct design supporting a similar safety profile of the quadrivalent product to that of the bivalent vaccine. The dose strength of SCTV01E is 30μg (5/5/5/15μg for TM22/TM23/TM28/TM41)/dose based on the nonclinical study of SCTV01E and SCTV01C in combination with the clinical studies of SCTV01C.<br>The study is a randomized, double-blind, and positive-controlled Phase III booster study. It will evaluate the immunogenicity and safety of one dose of SCTV01C or SCTV01E as booster compared with either one dose of Sinopharm inactivated COVID-19 vaccine (Cohort 1) or one dose of mRNA COVID-19 vaccine (Cohort 2).<br>Approximately 1,800 participants aged 18 years old and above will be enrolled in this study. 1,350 participants who previously received Sinopharm inactivated COVID-19 vaccine will be enrolled to Cohort 1. 450 participants who previously received mRNA COVID-19 vaccine (Comirnaty from Pfizer or mRNA-1273 from Moderna) or previously diagnosed with COVID-19 will be enrolled to Cohort 2.<br>In Cohort 1, 300 participants who were previously fully vaccinated with 2 or 3 doses of Sinopharm inactivated COVID-19 vaccine and with no previous COVID-19 history will form an immunogenicity subgroup (Subgroup 1) for nAb tests, and will be randomly assigned to SCTV01C

	Group, SCTV01E Group and the Sinopharm inactivated COVID-19 vaccine Group in a ratio of 1:1:1. The 300 participants for nAb tests will be stratified by age (18-54 years, ≥55 years), number of doses of previously received COVID-19 vaccines (2, 3), and interval between previous vaccination and the study vaccination (3-5 months, 6-8 months, 9-12 months, 13-24 months). The first 150 participants will form a cellular immune response subgroup for cellular immune response tests.<br>In Cohort 1, in addition to the 300 participants for immunogenicity tests, there are 1050 other participants who previously received at least one shot of Sinopharm COVID-19 inactivated vaccine, will form a subgroup (Subgroup 2) mainly for safety observation, and will be randomly assigned to SCTV01C Group, SCTV01E Group and Sinopharm inactivated COVID-19 vaccine Group in a ratio of 1:1:1. The 1050 participants mainly for safety observation will be stratified by age (18-54 years, ≥55 years), previous COVID-19 infection history (yes or no), number of doses of previously received COVID-19 vaccines (1, 2, 3) and interval between previous vaccination and the study vaccination (3-5 months, 6-8 months, 9-12 months, 13-24 months).<br>In Cohort 2, 450 participants who previously received 2 or 3 doses of mRNA COVID-19 vaccine (Comirnaty or mRNA-1273) or previously diagnosed with COVID-19 will be randomly assigned to SCTV01C Group, SCTV01E Group and the mRNA COVID-19 vaccine Group in a ratio of 1:1:1. Participants will be stratified by age (18-54 years, ≥55 years), number of doses of previously received COVID-19 vaccines (0, 1, 2, 3), previous COVID-19 infection history (yes or no), and interval between previous vaccination and the study vaccination (3-5 months, 6-8 months, 9-12 months, 13-24 months). In Cohort 2, the number of participants previously diagnosed with COVID-19 and previously not received any mRNA COVID-19 vaccine, should not be more than 50. All participants will have nAb tests. The first 150 participants will form a cellular immune subgroup for cellular immune response tests.<br>In Cohort 1, each participant in SCTV01C Group will receive one dose of SCTV01C on D0; each participant in SCTV01E Group will receive one

dose of SCTV01E on D0; each participant in Sinopharm inactivated COVID-19 vaccine Group will receive one dose of Sinopharm inactivated COVID-19 vaccine on D0.

In Cohort 2, each participant in SCTV01C Group will receive one dose of SCTV01C on D0; each participant in SCTV01E Group will receive one dose of SCTV01E on D0; each participant in mRNA COVID-19 vaccine Group will receive one dose of mRNA COVID-19 vaccine on D0.

Trial procedures:

The study procedure is described as Figure A and Figure B. An independent data and safety monitoring board (DSMB) will review the data of the study.

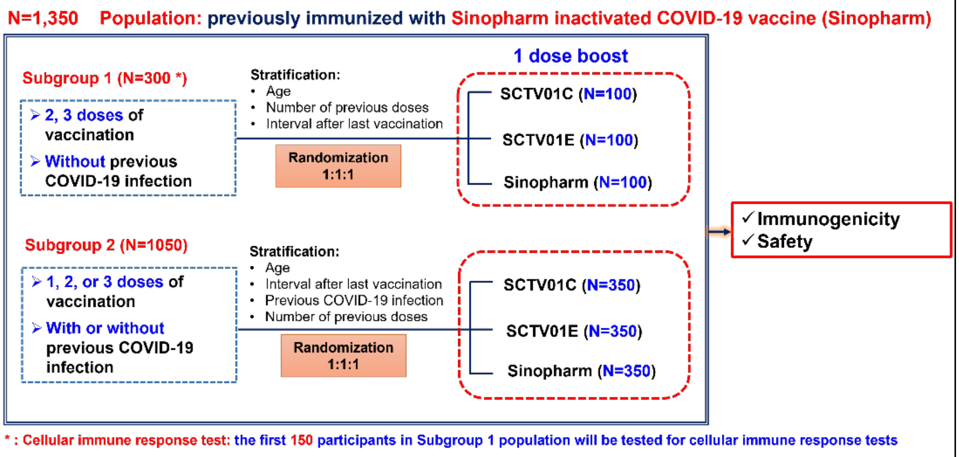

Figure A Study design for Cohort 1

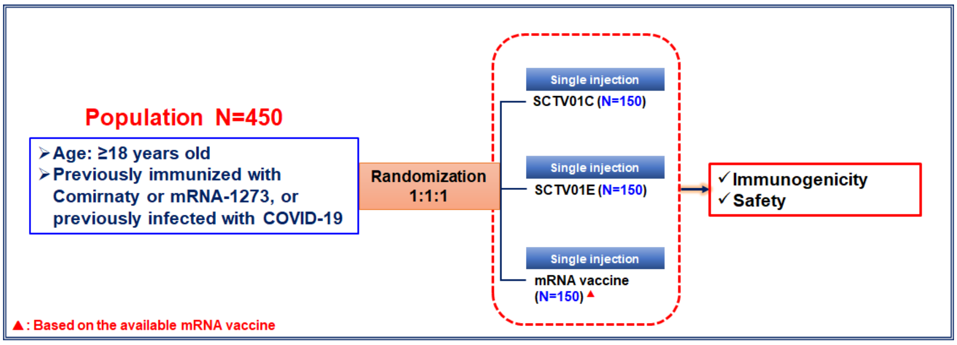

Figure B Study design for Cohort 2

The study consists of a screening period, a randomization and vaccination period, and a follow-up period.

Screening period: After participants sign the ICF, the screening phase visit will be conducted within 14 days.

Randomization: The qualified participants will be randomized before the study vaccination.

	**Vaccination:** Randomized participants will be vaccinated on D0.<br><br>**Follow-up period:**<br>**Safety follow-up:** All participants will be observed at site for at least 30 minutes after the study vaccination. Both the active monitoring and the spontaneous reporting will be used to collect the solicited and the unsolicited AEs. Solicited AEs within 7 days after study vaccination and unsolicited AEs within 28 days after study vaccination will be collected through vaccination record cards. SAEs and AESIs will be followed for 180±7 days after the study vaccination.<br>**Immunogenicity follow-up:** The participants in Subgroup 1 in Cohort 1 and all participants in Cohort 2 will be sampled for immunogenicity on D0 (before vaccination), D28 and D180. The nAb against Delta, Omicron variants and other variants will be tested.<br>The participants in the cellular immune response subgroup will be sampled for cellular immune response test on D0 (before vaccination) and D28.<br>After administration of the study vaccination, the participants will be continuously and systematically monitored for 180±7 days to ensure a prompt diagnosis and treatment according to FDA diagnosis and treatment practice when a participant experiences the suspicious symptoms of COVID-19. If a SARS-CoV-2 infection is confirmed 14 days after the study vaccination, sample will be collected from the nasopharyngeal/throat swab and viral sequencing will be used to identify the major SARS-CoV-2 variants.<br>The DSMB will review the safety data within D0-D28 of all the participants to assess the safety of SCTV01C and SCTV01E.<br><br>**Note:**<br>Participants aged between 54 years to less than 55 years old will be taken as 54 years old.
**Total Number of Participants**	1,800 participants are planned to be enrolled
**Study Site**	United Arab Emirates.

**Study Duration**	Each participant will be followed up for about 180 days after the vaccination on D0.
**Inclusion Criteria**	Participants are eligible to be included in the study only if the following conditions are met:<br>1. Male or female aged ≥18 years old when signing ICF;<br>2. **For Subgroup 1 in Cohort 1:** Participants who were previously vaccinated with 2 or 3 doses of Sinopharm inactivated COVID-19 vaccine. The interval between the date of last dose and the date of this study vaccination should be 3 to 24 months.<br>**For Subgroup 2 in Cohort 1:** 1) Participants who were previously vaccinated with 2 or 3 doses of Sinopharm inactivated COVID-19 vaccine, with or without COVID-19 history; or 2) Participants who were previously vaccinated with 1 dose of Sinopharm inactivated COVID-19 vaccine and previously diagnosed with COVID-19. The interval between the date of last dose/COVID-19 diagnosis and the date of this study vaccination should be 3 to 24 months.<br>**For Cohort 2:** 1) Participants who were previously vaccinated with 2 or 3 doses of mRNA COVID-19 vaccine (Comirnaty or mRNA-1273), with or without COVID-19 history; or 2) Participants who were previously vaccinated with 1 doses of mRNA COVID-19 vaccine (Comirnaty or mRNA-1273) and previously diagnosed with COVID-19; 3) Participants who were previously not vaccinated with any COVID-19 vaccine and previously diagnosed with COVID-19. The interval between the date of last dose/COVID-19 diagnosis and the date of this study vaccination should be 3 to 24 months.<br>3. The participant and/or his legally acceptable representative can sign written ICF, and can fully understand the trial procedure, the risk of participating in the trial, and other interventions that can be selected if

	they do not participate in the trial;<br>4. The participant and/or his legally acceptable representative have the ability to read, understand, and fill in record cards;<br>5. Healthy participants or participants with pre-existing medical conditions who are in stable condition. The "pre-existing medical conditions" include but not limited to hypertension, diabetes, Chronic cholecystitis and cholelithiasis, chronic gastritis that meet the described criteria. A stable medical condition is defined as disease not requiring significant change in therapy or no need for hospitalization as a consequence of worsening disease state for at least 3 months prior to enrollment;<br>6. Fertile men and women of childbearing potential voluntarily agree to take effective contraceptive measures from signing ICF to 6 months after the last dose of study vaccination; the pregnancy test results of women of childbearing potential are negative on screening.
**Exclusion Criteria**	A participant who conforms to any of the following criteria should be excluded from the study:<br>1. **For Subgroup 1 in Cohort 1 only**: Previously diagnosed with COVID-19.<br>2. Presence of fever within 3 days before the study vaccination;<br>3. A history of infection or disease related to severe acute respiratory syndrome (SARS), Middle East respiratory syndrome (MERS), or other disease corresponding use of immunosuppressants;<br>4. A history of allergic reactions to any vaccine or drug, such as allergy, urticaria, severe skin eczema, dyspnea, laryngeal edema, and angioneurotic edema;<br>5. A medical or family history of seizure, epilepsy, encephalopathy and psychosis;

	6. Immunocompromised patients suffering from immunodeficiency diseases, important organ diseases, immune diseases (including Guillain-Barre Syndrome [GBS], systemic lupus erythematosus, rheumatoid arthritis, asplenia or splenectomy caused by any circumstances, and other immune diseases that may have an impact on immune response in the investigator's opinion), etc.;<br>7. Long-term use of immunosuppressant therapy or immunomodulatory drugs for ≥14 days within the six months prior to enrollment. Whereas short-term (≤14 days) use of oral, inhaled and topical steroids are allowed;<br>8. Patients on antituberculosis therapy;<br>9. Presence of severe or uncontrollable cardiovascular diseases, or severe or uncontrollable disorders related to endocrine system, blood and lymphatic system, liver and kidney, respiratory system, metabolic and skeletal systems, or malignancies (skin basal cell carcinoma and carcinoma in-situ of cervix are exceptions and will not be excluded), such as severe heart failure, severe pulmonary heart disease, unstable angina, liver failure, or uremia;<br>10. Contraindications for intramuscular injection or intravenous blood sampling, including thrombocytopenia and other blood coagulation disorders;<br>11. Participants who received any immunoglobulin or blood products in the previous 3 months before enrollment, or plan to receive similar products during the study;<br>12. Participants who received other investigational drugs within 1 month before the study vaccination;<br>13. Participants who is at the acute state of disease, such as acute onset of chronic heart failure, acute sore throat, hypertensive encephalopathy,

	acute pneumonia, acute renal insufficiency, acute cholecystitis;<br>14. Participants received other drugs or vaccines used to prevent COVID-19, but participants previously received Sinopharm inactivated COVID-19 vaccine, Comirnaty or mRNA-1273 will not be excluded;<br>15. Participants vaccinated with influenza vaccine within 14 days or with other vaccines within 28 days before the study vaccination;<br>16. Those who donated blood or had blood loss (≥450 mL) within 3 months before the vaccination or plan to donate blood during the study period;<br>17. Those who are pregnant or breast-feeding or plan to be pregnant during the study period;<br>18. Those who plan to donate ovum or sperms during the study period;<br>19. Those who cannot follow the trial procedures, or cannot cooperate to complete the study due to planned relocation or long-term outing;<br>20. Those unsuitable for participating in the clinical trial as determined by the investigator because of other abnormalities that are likely to confuse the study results, or non-conformance with the maximal benefits of the participants;<br>21. Those who are tested positive for HIV in terms of serology.
**Withdrawal Criteria**	**A participant may withdraw from the study at any time at his/her own request.**<br>**Reasons for discontinuation from the study may include the following:**<br>• Refused further follow-up;<br>• Lost to follow-up;<br>• Death;<br>• Study terminated by sponsor;<br>• AEs;<br>• Participant request;

	• Investigator request;<br>• Protocol deviation.
**Study Suspension/Termination Criteria**	**In one of the following situations, the trial should be suspended or terminated:**<br>• When the DSMB requires a suspension/complete termination of the trial and the sponsor agrees;<br>• When the sponsor requires a suspension/complete termination of the trial and gives reasons for it;<br>• When the Ethics Committee requires a suspension/complete termination of the trial and gives reasons for it;<br>• When the regulatory agency requires a suspension/complete termination of the trial and gives reasons for it.
**Study Vaccine**	**Study Vaccine 1:** a bivalent SARS-CoV-2 trimeric spike protein vaccine (SCTV01C)<br>Appearance: emulsified, white suspension (due to the presence of adjuvant);<br>Components:<br>• Main active ingredients: SCTV01C-TM22 protein, SCTV01C-TM23 protein;<br>• SCT-VA02B adjuvant: the adjuvant 1X is comprised of 0.09 mg of citric acid, 0.59 mg of sodium citrate, 1.25 mg of polysorbate 80, 1.25 mg of span 85 and 10.75 mg of squalene;<br>• Excipients: citrate, sodium citrate, sodium chloride, polysorbate 80, sodium hydroxide, WFI;<br>Dosage form: solution for injection;<br>Strength: 20μg(10/10μg for TM22/TM23) /0.5mL/vial;<br>Route of vaccination: intramuscular injection into the lateral deltoid of the upper arm;

	Dosage of vaccination: 20μg;<br>Immunization procedure: 1 dose, inoculated with 1 dose on D0;<br>Storage conditions: stored and transported at 2 ~ 8°C away from light;<br>Validity period: tentatively 24 months;<br>Manufacturer: Sinocelltech Ltd.<br>**Study Vaccine 2:** a COVID-19 Alpha/Beta/Delta/Omicron Variants S-Trimer Vaccine (SCTV01E);<br>Appearance: emulsified, white suspension (due to the presence of adjuvant);<br>Components:<br>• Main active ingredients: SCTV01E-TM22 protein, SCTV01E-TM23 protein, SCTV01E-TM28 protein, and SCTV01E-TM41 protein;<br>• SCT-VA02B (1×) adjuvant: the adjuvant 1X is comprised of 0.09 mg of citric acid, 0.59 mg of sodium citrate, 1.25 mg of polysorbate 80, 1.25 mg of span 85 and 10.75 mg of squalene.<br>• Excipients: citrate, sodium citrate, sodium chloride, polysorbate 80, sodium hydroxide, WFI;<br>Dosage form: solution for injection;<br>Strength: 30μg (5/5/5/15μg for TM22/TM23/TM28/TM41)/0.5mL/vial;<br>Route of vaccination: intramuscular injection into the lateral deltoid of the upper arm;<br>Dosage of vaccination: 30μg;<br>Immunization procedure: different procedures according to the study design<br>Storage conditions: stored and transported at 2 ~ 8°C away from light;<br>Validity period: tentatively 24 months;<br>Manufacturer: Sinocelltech Ltd.

	**Sinopharm inactivated COVID-19 vaccine**: It will be used according to the medicine specification<br>**mRNA COVID-19 vaccine:** Based on the available mRNA vaccine. Detailed information refers to medicine specification.
**Statistical Analysis**	Detailed methodology for summary and statistical analyses of the data collected in this study is outlined here and will be further detailed in a statistical analysis plan (SAP). The SAP may modify what is outlined in the protocol where appropriate; however, any major modifications of the primary endpoint definitions or their analyses will also be reflected in a protocol amendment.<br>**Hypothesis:**<br>$GMTC1_{Delta}$= GMT of nAb against Delta variant on D28 of SCTV01C in Cohort 1;<br>$GMTE1_{Delta}$= GMT of nAb against Delta variant on D28 of SCTV01E in Cohort 1;<br>$GMTS1_{Delta}$= GMT of nAb against Delta variant on D28 of Sinopharm inactivated vaccine in Cohort 1;<br>$GMTC1_{Omicron1}$= GMT of nAb against Omicron BA.1 variant on D28 of SCTV01C in Cohort 1;<br>$GMTE1_{Omicron1}$ = GMT of nAb against Omicron BA.1 variant on D28 of SCTV01E in Cohort 1;<br>$GMTS1_{Omicron1}$ = GMT of nAb against Omicron BA.1 variant on D28 of Sinopharm inactivated vaccine in Cohort 1;<br>$GMTC1_{Omicron5}$= GMT of nAb against Omicron BA.5 variant on D28 of SCTV01C in Cohort 1;<br>$GMTE1_{Omicron5}$ = GMT of nAb against Omicron BA.5 variant on D28 of SCTV01E in Cohort 1;

	$GMTS1_{Omicron5}$ = GMT of nAb against Omicron BA.5 variant on D28 of Sinopharm inactivated vaccine in Cohort 1;<br>$GMTC2_{Delta}$= GMT of nAb against Delta variant on D28 of SCTV01C in Cohort 2;<br>$GMTE2_{Delta}$= GMT of nAb against Delta variant on D28 of SCTV01E in Cohort 2;<br>$GMTM2_{Delta}$= GMT of nAb against Delta variant on D28 of mRNA COVID-19 vaccine in Cohort 2.<br>$GMTC2_{Omicron1}$= GMT of nAb against Omicron BA.1 variant on D28 of SCTV01C in Cohort 2;<br>$GMTE2_{Omicron1}$= GMT of nAb against Omicron BA.1 variant on D28 of SCTV01E in Cohort 2;<br>$GMTM2_{Omicron1}$= GMT of nAb against Omicron BA.1 variant on D28 of mRNA COVID-19 vaccine in Cohort 2.<br>$GMTC2_{Omicron5}$= GMT of nAb against Omicron BA.5 variant on D28 of SCTV01C in Cohort 2;<br>$GMTE2_{Omicron5}$= GMT of nAb against Omicron BA.5 variant on D28 of SCTV01E in Cohort 2;<br>$GMTM2_{Omicron5}$= GMT of nAb against Omicron BA.5 variant on D28 of mRNA COVID-19 vaccine in Cohort 2.<br>For the primary efficacy objectives, the null hypotheses are:<br>**For Cohort 1:**<br>• **H11**: GMR13=$GMTE1_{Omicron1}$ /$GMTS1_{Omicron1}$$\leq$ 1;<br>• **H12**: GMR14=$GMTC1_{Omicron1}$ /$GMTS1_{Omicron1}$$\leq$ 1;<br>• **H13**: GMR12= $GMTE1_{Delta}$/$GMTS1_{Delta}$$\leq$ 1;<br>• **H14**: GMR12= $GMTE1_{Delta}$/$GMTS1_{Delta}$$\leq$ 1;<br>• **H15**: GMR15=$GMTE1_{Omicron5}$ /$GMTS1_{Omicron5}$$\leq$ 1;<br>• **H16**: GMR16=$GMTC1_{Omicron5}$/$GMTS1_{Omicron5}$$\leq$ 1;<br>**For Cohort 2:**

- **H21**: GMR22=$GMTE2_{Omicron1}$ /$GMTM2_{Omicron1} \leq 0.67$;
- **H22**: GMR24=$GMTC2_{Omicron1}$ /$GMTM2_{Omicron1} \leq 0.67$;
- **H23**: GMR21= $GMTE2_{Delta}$/$GMTM2_{Delta} \leq 0.67$;
- **H24**: GMR23= $GMTC2_{Delta}$/$GMTM2_{Delta} \leq 0.67$;
- **H25**: GMR26= $GMTE2_{Omicron1}$/$GMTM2_{Omicron1} \leq 1$;
- **H26**: GMR28=$GMTC2_{Omicron1}$ /$GMTM2_{Omicron1} \leq 1$;
- **H27**: GMR29=$GMTE2_{Omicron5}$ /$GMTM2_{Omicron5} \leq 0.67$;
- **H28**: GMR210=$GMTC2_{Omicron5}$/$GMTM2_{Omicron5} \leq 0.67$;
- **H29**: GMR211= $GMTE2_{Omicron5}$/$GMTM2_{Omicron5} \leq 1$;
- **H210**: GMR212=$GMTC2_{Omicron5}$ /$GMTM2_{Omicron5} \leq 1$;
- **H211**: GMR25=$GMTE2_{Delta}$ /$GMTM2_{Delta} \leq 1$;
- **H212**: GMR27= $GMTC2_{Delta}$/$GMTM2_{Delta} \leq 1$.

The estimand framework of primary efficacy objectives is listed in Tables below.

For Cohort 1:

Table A Estimand framework of primary objectives

Population	Population aged ≥18 years previously vaccinated with inactivated vaccine
Treatment conditions	Test: SCTV01C, SCTVO1E<br>Control: Sinopharm inactivated COVID-19 vaccine
Variables	Neutralizing antibody titers against Delta or Omicron variant on D28 after first vaccination
Intercurrent event 1	COVID-19 infection up to D28 after first vaccination. A principal stratum strategy will be used, the participants who are diagnosed with COVID-19 up to D28 after first vaccination are excluded from this estimand.
Intercurrent event 2	Receiving of other drugs or vaccines that will modify the immunity against Delta or Omicron variant up to D28 after first vaccination. A principal stratum strategy will be used, the participants who receive other drugs or vaccines that will modify the immunity against Delta or Omicron variant up to D28 after first vaccination are excluded from this estimand.
Population-	Ratio of geometric means of the neutralizing antibody

level summary	titers

For Cohort 2:

Table B Estimand framework of primary objectives

Population	Population aged ≥18 years previously vaccinated with mRNA vaccine
Treatment conditions	Test: SCTV01C, SCTVO1E<br>Control: mRNA COVID-19 vaccine
Variables	Neutralizing antibody titers against Delta or Omicron variant on D28 after first vaccination
Intercurrent event 1	COVID-19 infection up to D28 after first vaccination. A principal stratum strategy will be used, the participants who are diagnosed with COVID-19 up to D28 after first vaccination are excluded from this estimand.
Intercurrent event 2	Receiving of other drugs or vaccines that will modify the immunity against Delta or Omicron up to D28 after first vaccination. A principal stratum strategy will be used, the participants who receive other drugs or vaccines that will modify the immunity against Delta or Omicron variant up to D28 after first vaccination are excluded from this estimand.
Population-level summary	Ratio of geometric means of the neutralizing antibody titers

Multiplicity:

For Cohort 1:

A fixed sequential hierarchical approach will be used to control the type I error at one-sided 0.025. The hypothesis will be tested according to the Estimand frame as defined in Table A in an order of H11, H12, H13, H14, H15 and H16. The following test will be tested only when the previous one reaches the statistical significance at one-sided significance level of 0.025.

For Cohort 2:

A fixed sequential hierarchical approach will be used to control the type I error at one-sided 0.025. The hypothesis will be tested in an order of H21,

	H22, H23, H24, H25, H26, H27, H28, H29, H210, H211, and H212 the participants according to the Estimand frame as defined in **Table B**. The following test will be done only when the previous one reaches the statistical significance at one-sided significance level of 0.025.<br>The multiplicity control procedure may be adjusted according to external information. More details will be defined in the SAP which will be finalized before the study is unblinded.<br>**Sample size calculation:**<br>Totally 1800 participants aged ≥18 years who were previously vaccinated with either inactivated or mRNA COVID-19 vaccine or previously diagnosed with COVID-19 will be enrolled. 300 participants (100 in SCTV01C Group, 100 in SCTV01E Group, 100 in Sinopharm COVID-19 vaccine Group) in subgroup 1 in Cohort 1 will have nAb tests. 450 participants (150 in SCTV01C Group, 150 in SCTV01E Group, 150 in mRNA COVID-19 vaccine Group) in Cohort 2 will have nAb tests.<br>For Cohort 1, the sample size is determined based on below assumptions:<br>• The standard deviation of neutralizing antibody titers under log10 transformation is 0.4;<br>• The 1-sided type I error is 0.025<br>• Power is above 80%;<br>• GMR between SCTV01C/E and Sinopharm vaccine=1.6<br>• The dropout rate during study is about 10%;<br><br>For Cohort 2, the sample size is determined based on below assumptions:<br>• The standard deviation of neutralizing antibody titers under log10 transformation is 0.4;<br>• The 1-sided type I error is 0.025<br>• Power is above 80%;<br>• GMR between SCTV01C/E and mRNA vaccine =1;<br>• non-inferiority margin as 0.67;

	• The dropout rate during study is about 10%;<br><br>**Statistical populations:**<br>Full Analysis Set (FAS): All randomized participants who received one dose of investigational product (IP).<br>Per-Protocol Set (PPS): All participants in the FAS set who received planned doses of IP per schedule and have no major protocol deviations, as determined and documented by Sponsor prior to database lock and unblinding, that impact critical or key study data. Those who are COVID-19 infected or take other vaccine/drug after the vaccination and before D28 that could compromise the immunogenicity evaluation will be excluded from PPS as defined in the Estimand frame.<br>Safety Set (SS): All randomized participants who received one dose of IP.<br>Immunogenicity full analysis set (I-FAS): All participants in the FAS who had a valid immunogenicity test result prior to receiving the IP and at least 1 valid result after receiving the IP.<br>Immunogenicity per-protocol set (I-PPS): All participants in the PPS who had a valid immunogenicity test result prior to receiving the IP and at least 1 valid result after receiving the IP.<br>**Statistical analysis methods:**<br>For each cohort, once the safety data within 28 days and immunogenicity data on D28+3 were acquired, it will be analyzed by unblinded team who are independent to the study operation team and are not directly involved in the study activities. The result will be further used for submission to regulatory authority. The specific analysis time point may be adjusted according to the progress of the trial.<br>**General principles**<br>The statistical analysis is carried out with the descriptive and pre-specified statistical test method. The analytical procedures will be detailed in the

	statistical analysis plan (SAP).<br>American SAS 9.4 or above will be used for statistical analysis.<br>Descriptive statistics of continuous variables will include mean, standard deviation, median, minimum, and maximum values. The classification variable will be described by number and percentage. The calculation method of percentage will be defined in the SAP.<br>The expected values, standard errors, and 95% confidence interval (Cl) will be calculated based on the assumed distribution and pre-specified models, as defined in the SAP.<br>**The Demographic and Baseline Characteristics**<br>The Demographic and Baseline Characteristics, including protocol deviations will be listed.<br>Demographic data and baseline indicators will be analyzed among the FAS. All demographic data (age, sex, race, ethnicity, et al) and baseline variables (physical examination, pregnancy test, history of diseases, history of COVID-19, medication history, interval between time of administration of IP and the last time of COVID-19 vaccine administration/diagnosed with COVID-19, the type of previous COVID-19 vaccinations and serum antibody titer before the administration of IP) are summarized.<br>For continuous variables, descriptive statistics (the number of participants, mean, standard deviation, minimum, median and maximum values) are used; and for classified variables, the number and percentage are calculated.<br>**Study treatment exposure and compliance**<br>The exposure dose and trial compliance are descriptively summarized, including safety evaluation and immunogenicity-testing compliance.<br>**Immunogenicity and exploratory analysis**<br>The GMT of neutralizing antibody for each group with corresponding 2-

	sided 95% CI will be estimated at each post-baseline time point using an analysis of covariance. The comparison of GMT of neutralizing antibody between the treatment groups at each post-baseline time point will also be provided using an analysis of covariance.<br>The 95% CI of seroresponse using the Clopper-Pearson method will be provided. Cochran-Mantel-Haenszel method will be used for comparison of the seroresponse between the treatment groups.<br>The change in the number of IFN-γ positive (characterizing Th1) and IL-4 positive (characterizing Th2) T cell subsets at each post-baseline time point will be statistically described, and the nonparametric test will be applied for the statistical comparison between groups. Detailed statistical analysis methods are described in the SAP for further reference.<br>**Safety Analysis**<br>Safety analysis will be based on SS.<br>AEs and SAEs are encoded based on the *Medical Dictionary for Regulatory Activities* (MedDRA) and also based on the document the classified statistics was made according to the system organ class (SOC) and preferred term (PT). In this trial, the treatment emergent adverse events (TEAEs) are summarized, and the adverse medical conditions occurring before the study vaccination are listed. Unless otherwise specified, the adverse events as described below are TEAEs.<br>The incidence of AEs, SAEs and AESIs, the number and percentage of participants with AEs, SAEs and AESIs in each group will be summarized respectively. The 2-sided 95% CI will be also provided for the percentage of participants with any solicited AE for each treatment group using the Clopper-Pearson method. The adverse events related to the study vaccine, SAEs and AESIs will be listed.
**Independent**	The sponsor will establish the DSMB to review the safety data of the

**Data and Safety Monitoring Board**	clinical trials. The DSMB members include experts in the field of vaccine clinical trials, biostatisticians and epidemiologists. See the "DSMB charter" for details of its working documents.

Table 1 Schedule of Activities

	Screening period	Vaccination	Follow-up period		
**Visit**	**V1**	**V2**	**V3**	**V4**	**V5**
**Planned visit date**	**D-14～D0**	**D0**	**D7**	**D28**	**D180 (EOS)**
**Visit window period**	/	/	**+2 days**	**+3 day**	**±7 days**
**Management and general procedures**					
Signing the informed consent form	●				
Confirm participant meets inclusion and exclusion criteria[1]	●	●★			
Demographic data[2]	●				
Recording the medical history[3]	●				
Assigning the screening number	●				
Physical examination[4]	●				
Vital signs[5]	●	●★			
Nasal/pharyngeal/throat swab nucleic acid test	●				
HIV testing	●				
Urine pregnancy test (for women of childbearing potential only)[6]	●	●★			
Randomization		●*			
Vaccination		●			
Viral sequencing			●**		
**Immunogenicity follow-up visit**					
Neutralizing antibodies test for Delta, Omicron variants and other variants[7]		●		●	●
Cellular immune response[8]		●		●	
**Safety follow-up visit**					
Solicited AEs[9]		Record solicited AE			
Unsolicited AEs[9]		Record unsolicited AE			
SAEs and AESIs[10]		●	●	●	●
Observing for at least 30 minutes after the vaccination		●			
Distributing the vaccination record cards (VRCs)		●			
Reviewing the VRCs			●	●	
Distributing the thermometer		●			
Recording the concomitant medication	●	●	●	●	●#

Comments:

★: If screening and vaccination are on the same day (D0), there is no need to repeat the items corresponding to '•★' before vaccination.

#: 28 days after the study vaccination, only the prohibited concomitant medication and concomitant medication used to treat SAEs, AESIs should be recorded.

*: All the tests should be done and results should be available before the randomization on the vaccination day.

**: If the SARS-CoV-2 infection is confirmed after 14 days of the study vaccination, virus will be isolated

from the nasal/nasopharyngeal/throat swab and viral sequencing will be used to identify the major SARS-CoV-2 variants.

1. Inclusion/exclusion should be reviewed during the screening period and on the day of vaccination.
2. Demographic data: including age, sex, race, ethnicity, occupation (working and living circumstances), height, body weight, BMI (derived from height and body weight). The participants should also provide contact information like current phone number and/or E-mail. In subsequent follow-up visits, if the contact information is changed, it should be updated accordingly (if applicable).
3. Records of medical history: including the history of SARS-COV-2 vaccination, history of COVID-19, other vaccinations within 90 days, medication use within 28 days; major surgery, allergic history, and other known significant diseases.
4. Physical examinations: general conditions, head & neck, lymph node, skin, chest, abdomen, musculoskeletal system and other examinations necessary for the study.
5. Vital signs: blood pressure, respiration rate, pulse rate, and body temperature.
6. Urine pregnancy test (for women of childbearing potential only): the urine pregnancy test may be performed routinely, while the blood pregnancy test may be performed if the investigator deems it necessary. A woman is considered of childbearing potential (WOCBP), i.e. fertile, following menarche and until becoming post-menopausal unless permanently sterile. Permanent sterilization methods include hysterectomy, bilateral salpingectomy and bilateral oophorectomy. A postmenopausal state is defined as no menses for 12 months without an alternative medical cause.
7. Neutralizing antibodies test for Delta, Omicron variants and other variants (the other variants will be tested per pandemic and regulatory requirements): only applied to participants of Subgroup 1 in Cohort 1 and all participants of Cohort 2.
8. Cellular immune response (only applied to the cellular immune response subgroup): the number of IFN-γ positive (characterizing Th1) and IL-4 positive (characterizing Th2) T cell subsets before and 28 days after each study vaccination.
9. Solicited and unsolicited AEs: collect solicited AEs from D0 to D7; and unsolicited AEs from D0 to D28.
10. SAEs, and AESIs: SAEs, and AESIs will be collected on visit day or reported by participants actively at any time. If participants cannot come to site on visit day, phone call, short message, email or other contacting method will be used for safety follow-up.

Table of Contents

PROTOCOL SYNOPSIS 1
TABLE OF CONTENTS 25
LIST OF TABLES 27
LIST OF FIGURES 28
ABBREVIATIONS 29
1 STUDY BACKGROUND 32
1.1 BACKGROUND 32
1.2 ETIOLOGY FEATURES 33
1.3 INTRODUCTION OF STUDY VACCINE 34
1.4 SUMMARY OF PRECLINICAL STUDY 36
1.5 CLINICAL SAFETY OF SCTV01C 41
1.6 STUDY SIGNIFICANCE 41
1.7 ASSESSMENT OF BENEFIT/RISK 42
2 STUDY OBJECTIVES AND ENDPOINT 44
2.1 STUDY OBJECTIVES 44
2.2 STUDY ENDPOINT 44
3 STUDY DESIGN 45
3.1 STUDY DESIGN 46
3.2 BLINDING AND UNBLINDING METHODS 49
3.3 DEFINITION OF END OF STUDY 50
4 STUDY POPULATION 50
4.1 INCLUSION CRITERIA 50
4.2 EXCLUSION CRITERIA 51
5 DISCONTINUATION OF STUDY INTERVENTION 53
5.1 WITHDRAWAL CRITERIA 53
5.2 STUDY SUSPENSION/TERMINATION CRITERIA 54
6 DESCRIPTION OF STUDY PROCEDURES AND VISITS 54
6.1 V1 (SCREENING, D-14~D0) 54
6.2 V2 (D0, VACCINATION) 55
6.3 V3 (D7+2D) 56
6.4 V4 (D28+3D) 56
6.5 V5/EOS (D180±7D) 57
6.6 UNPLANNED CONTACT AND FOLLOW-UP 57
7 STUDY VACCINES 57
7.1 BASIC INFORMATION OF VACCINES 57
7.2 STUDY VACCINE MANAGEMENT 58
7.3 VACCINATION ROUTE AND DOSE 58
7.4 VACCINATION PROCEDURE 59
7.5 CONCOMITANT MEDICATION 59
7.6 PARTICIPANT COMPLIANCE 60
8 COLLECTION, PROCESSING AND TESTING OF BIOLOGICAL SAMPLES 60
8.1 COLLECTION, PROCESSING AND TESTING OF IMMUNOGENICITY SAMPLES 60
8.2 COLLECTION, PROCESSING AND TESTING OF VIRUS TEST SAMPLES 61
9 STUDY EVALUATIONS AND REPORTS 61
9.1 SAFETY ASSESSMENT 61
9.2 IMMUNOGENICITY ASSESSMENT 71

9.3 DIAGNOSIS AND TREATMENT OF COVID-19 INFECTION 72
10 DATA MANAGEMENT 74
10.1 SOURCE DATA AND THE FILLING AND TRANSFER OF ECRF 74
10.2 DATABASE PROPOSAL AND DESIGN 75
10.3 ENTRY OF DATA 75
10.4 MEDICAL CODING 75
10.5 DATABASE LOCKING AND EXPORTING 76
10.6 ARCHIVE OF STUDY RECORDS 76
11 STATISTICAL ANALYSIS 76
11.1 HYPOTHESIS 76
11.2 ESTIMAND 78
11.3 MULTIPLICITY 79
11.4 SAMPLE SIZE CALCULATION 79
11.5 STATISTICAL POPULATIONS 80
11.6 STATISTICAL ANALYSIS METHODS 81
12 MANAGEMENT OF CLINICAL TRIAL 82
12.1 DECLARATION 82
12.2 ETHICS 82
12.3 INFORMED CONSENT 83
12.4 REVISION OF CLINICAL TRIAL PROTOCOL 84
12.5 PROTOCOL DEVIATION 84
12.6 MONITORING 84
12.7 QUALITY ASSURANCE AND AUDIT 85
12.8 INTELLECTUAL PROPERTY 85
12.9 PARTICIPANTS' PRIVACY 85
12.10 MONITORING BOARD 86
13 FINANCE AND INSURANCE 86
14 PUBLISHING AND DATA SHARING POLICIES 87
15 APPENDICES 88
15.1 APPENDIX I: TOXICITY RATING SCALE FOR HEALTHY ADULT AND ADOLESCENT VOLUNTEERS IN PREVENTIVE VACCINE CLINICAL TRIAL - FDA STANDARD 88
16 REFERENCES 92

List of Tables

Table 1 Schedule of Activities 23
Table 2 List of solicited AE 62
Table 3 List of Potential Immune-mediated Diseases to be Collected in the Context of Vaccines 64
Table 4 List of Adverse Events of Special Interest Applicable to COVID-19 vaccines (Guidance Document from SPEAC) 65
Table 5 List of Adverse Events of Special Interest Relevant to COVID-19 (Guidance Document from SPEAC) 65
Table 6 General principles for the grading of adverse events 66
Table 7 COVID-19 disease severity 72
Table 10 Scale of clinical abnormalities 88
Table 11 Scale of vital signs 88
Table 12 Scale of adverse events 89
Table 13 Abnormal Results of Laboratory Examinations 89
Table 14 Hematological Abnormality 90
Table 15 Abnormalities of Urine Routine Examination 91

List of Figures

Figure 1 Study design for Cohort 1......48
Figure 2 Study design for Cohort 2......48
Figure 3 Diagnosis process of COVID-19 cases......74

ABBREVIATIONS

**Acronym or terminology**	**Explanation**
ACE-2	Angiotensin converting enzyme 2
ADE	Antibody-mediated infection enhancement
AE	Adverse event
AESI	Adverse event of special interest
ALT	Alanine aminotransferase
AST	Aspartate aminotransferase
AR	Adverse reaction
BUN	Blood Urea Nitrogen
CDISC	Clinical Data Interchange Standards Consortium
CI	Confidence interval
COVID-19	Novel coronavirus pneumonia
CRO	Contract Research Organization
DBL	Database Lock
DM	Data manager
DMP	Data management plan
DSMB	Data and Safety Monitoring Board
eCRF	Electronic case report form
EDC	Electronic data capture
ERC	Ethics Committee
FAS	Full analysis set
GBS	Guillain-Barre Syndrome
GCP	Good Clinical Practice
GMT	Geometric mean titers
H_0	Null hypothesis
HIV	Human immunodeficiency virus
HR	Hazard ratio
IB	Investigator's brochure
ICF	Informed consent form
ICH	International Conference on Harmonization of Technical Requirements
I-FAS	Immunogenicity of full analysis set
IFN-γ	Interferon-γ
IL	Interleukin

Acronym or terminology	Explanation
I-PPS	Immunogenicity Per-Protocol Set
IRB	Institutional Review Board
IP	Investigational product
ITT	Intent-to-treat
IWRS	Interactive web response system
MedDRA	Medical Dictionary for Regulatory Activities
MERS	Middle East respiratory syndrome
mITT	Modified intent-to-treat set
MTD	Maximum tolerated dose
mRNA	Messenger ribonucleic acid
NMPA	National Medical Products Administration
nAb	Neutralizing antibody
PCR	Polymerase chain reaction
PPS	Per-Protocol Set
PT	Preferred term
QC	Quality control
RBD	Receptor-binding domain
SAE	Serious adverse event
SAP	Statistical analysis plan
SARS	Severe acute respiratory syndrome
SAS	Statistical analysis system
SDV	Source data verification
SARS-CoV-2	Novel coronavirus
S-ECD	Extracellular region of recombinant spike protein of novel coronavirus mutant strain
SOC	Systematic organ classification
SOP	Standard operating procedure
SpO_2	Oxygen saturation
SS	Safety analysis set
SUSAR	Suspected and unexpected serious adverse reaction
TEAE	Treatment emergent adverse events
Th1	Helper T cell 1
Th2	Helper T cell 2
TNF-α	Tumor necrosis factor-α

**Acronym or terminology**	**Explanation**
RBC	Red blood cell count
RdRp	Ribonucleic acid polymerase
RNA	Ribonucleic acid
RR	Respiratory rate
RT-PCR	Reverse polymerase chain reaction
URL	Uniform resource locater
US FDA	United States Food and Drug Administration
VE	Vaccine efficacy
VED	Vaccine-enhanced disease
VRC	Vaccination record card
VOC	Variants of Concern
WHO	World Health Organization
WHO DD	Dictionary of Drugs of World Health Organization
2019-nCoV	Novel coronavirus

1 STUDY BACKGROUND

1.1 BACKGROUND

Novel coronavirus pneumonia (COVID-19) is a newly emerging acute respiratory infection caused by the novel coronavirus (2019-nCOV or SARS-CoV-2) infection. COVID-19, first detected in humans in December 2019, has quickly spread to more than 210 countries and regions worldwide. World Health Organization (WHO) declared the COVID-19 outbreak a public health emergency of international concern on January 30, 2020, followed by the declaration that the disease has pandemic characteristics on March 11, 2020. As of 13 June, 2022, WHO reported a total of 532,887,351 confirmed cases, including 6,307,021 deaths. The outbreak and epidemic of COVID-19 pose a serious threat to human health and survival. At present, vaccine becomes the most effective means to prevent virus infection. Several COVID-19 vaccines have been approved for conditional marketing or emergency use, and COVID-19 vaccines have been inoculated in large scale worldwide[1].

SARS-CoV-2 is an RNA single-stranded virus that is prone to deletion mutation, which occurs mostly in recurrent deletion regions (RDRs) of the S protein. Deletion or mutation may change the conformation of S protein, resulting in the decrease of vaccine immune effect and virus immune escape. Although the early D614G mutation (B.1) that enhances the binding of the S protein to the ACE2 receptor does not reduce sensitivity to neutralizing antibodies, with the pandemic of SARS-CoV-2, several high-risk mutant strains have emerged worldwide: Alpha (B.1.1.7), Beta (B.1.351), Gamma (P.1), Delta (B.1.617.2) and Omicron (B.1.1.529). Studies have shown that these high-risk strains increase the transmission of the virus, aggravate disease development (increase hospitalization or mortality), reduce the COVID-19 immunity induced by previous infections or immunizations, reduce the efficacy of treatments or vaccines, and invalidate diagnostic tests. The Delta variant, first detected in India in December 2020, now accounts for most of the coronavirus infections in many regions. However, the newly emerging Omicron (B.1.1.529) variant, which was first reported to WHO from South Africa on November 24 2021, and was designated as a VOC on November 26 2021, spreads much faster than Delta variant. The Omicron variant has turn to be the dominant variant in many provinces across the world. The Omicron variant has at least 50 mutations, 30 of which are on the S protein, most of which are located in the domains that interacts with hACE2. The Omicron variant comprises of mutations discovered in the Delta variant that are considered to increase transmissibility and mutations discovered in the Beta and Delta variants that are believed to promote immune escape. Preliminary evidence suggests an increased risk of reinfection with

this variant, as compared to other VOCs. The number of cases of this variant appears to be increasing in almost all provinces in South Africa. Omicron has spread to various countries in Europe, including Belgium, the Netherlands, France, the UK, Australia and Canada within a short time. Given the adverse effects and high transmissibility of Omicron and Delta, there is an urgent need for new generation of vaccines with high protective efficacy against high-risk variants.

Original-strain based 1st generation vaccine provides insufficient protection to variants with decreased vaccine-efficacy. Omicron can escape first generation vaccine, and a boost dose only provides likely less than 3-6 month protection resulting vaccinated population essentially naïve to Omicron. 3 doses of Omicron vaccine are needed to induce long-lasting immunity.

SCTV01C is the first generation vaccine containing two antigens TM22 and TM23 while SCTV01E containing four antigens TM22, TM23, TM28 and TM41. Compared with single antigen with sequence from one variant, multiple antigens with sequences from different variants can induce board neutralizing antibodies spectrum against multiple variants.

SCTV01E is specifically designed for the prevention of infection with SARS-CoV-2 and its variants. An affordable and thermal stable 2nd generation COVID-19 vaccine providing broad immunity to existing and emerging variants.

1.2 ETIOLOGY FEATURES

SARS-CoV-2 belongs to β genus coronavirus and has an envelope with round or elliptic particles in diameter of 60~140 nm. It has five essential genes, which target four structural proteins, i.e., nucleoprotein (N), virus envelope (E), matrix protein (M) and spike protein (S), and ribonucleic acid (RNA) dependent RNA polymerase (RdRp). The nucleoprotein (N) wraps the RNA genome to form the nucleocapsid, which is surrounded by a viral envelope (E), in which the matrix protein (M) and spike protein (S) are embedded. The spike protein enters the cell by binding with angiotensin converting enzyme 2 (ACE-2). With isolation and co-culture test *in vitro*, novel coronavirus can be detected in human respiratory tract epithelial cells in about 96 hours and in Vero E6 and Huh-7 cell lines in about 4 to 6 days[2].

Coronaviruses are sensitive to ultraviolet, heat, and can be effectively inactivated by ethyl ether, 75% ethanol, chlorine-containing disinfectant, peracetic acid and chloroform at 56°C for 30 minutes, except for chlorhexidine.

1.2.1 CLINICAL MANIFESTATION

The most common symptom of COVID-19 is fever, dry cough and fatigue, while some patients

experience smell and taste disorder as the initial symptom. A small portion of patients also have nasal congestion, runny nose, throat pain, conjunctivitis, myalgia and diarrhea and other symptoms. Severe patients usually develop dyspnea and/or hypoxemia one week after the onset, and more severe patients may rapidly progress to acute respiratory distress syndrome, septic shock, refractory metabolic acidosis, coagulation dysfunction and failure of multiple organs. Very few patients may also have central nervous system involvement and avascular necrosis of the extremum. Severe and critical patients may have moderate to low fever or even no fever during the COVID-19 infection. Mild patients may show low fever, mild weakness, smell, and taste disorders, but no pneumonia symptom, and a small number of patients with the COVID-19 infection can have no obvious clinical symptoms. While COVID-19 infection generally have a mild course in most patients, a number of pre-existing co-morbidities determine the severity of infection and the outcome in the patients, and the critical conditions are mostly seen in elderly with chronic underlying diseases, women in the third trimester and perinatal period and the obese population[2].

1.2.2 INFECTION SOURCE AND ROUTE OF TRANSMISSION

Patients infected with novel coronavirus and asymptomatic infected persons are the main source of spreading. They are contagious during the incubation period and are highly contagious within 5 days after the onset of the disease. Transmission is mainly through respiratory droplets and close contact. Infection can also be caused by contact with objects contaminated with the virus. As there is a possibility of aerosol transmission in a relatively closed environment when exposed to high concentration of aerosols for a long time and novel coronavirus can be isolated from feces and urine, attention should be paid to its exposure to environmental pollution or aerosol transmission.

1.3 INTRODUCTION OF STUDY VACCINE

1.3.1 INTRODUCTION OF SCTV01C

The bivalent SARS-CoV-2 trimeric spike protein vaccine (code name SCTV01C) is a recombinant protein vaccine developed by Sinocelltech Ltd., with genetic engineering technology adopted to express in CHO cells. SCTV01C is SARS-CoV-2 bivalent recombinant trimeric subunit protein with oil-in-water adjuvant suspension, containing TM22 and TM23 in three dose levels of 10/20/30μg with 0.5 mL/vial[3].

The S protein of COVID-19 is a key antigen in the design of COVID-19 vaccine. The S protein binds to the host cell's ACE-2 receptor via the receptor-binding domain and is cleaved by the host protease into S1 polypeptide containing the receptor-binding domain (RBD) and S2

polypeptide mediating the virus's fusion with the cell membrane, stimulating B cells to produce high titer neutralizing antibodies against RBD and abundant T cell epitopes that can induce specific CTL reaction in T cells. SCTV01C trimeric subunit protein consists of the ECD region of S protein and T4-foldon. SCTV01C, simulating S protein, has complete biological function and natural structural properties, which ensures the high proportion of neutralizing epitope of correct structure and the induction of neutralizing antibodies with high titers. At the same time, SCTV01C has more T cell epitopes and can induce stronger resistance to variants infection than the RBD protein vaccines due to 5 time larger in its molecular weight.

The adjuvant (SCT-VA02B) is an oil-in-water adjuvant, which its composition, the formula, process and main quality control (particle size) are consistent with the commercial marketable MF59 adjuvant. MF59 adjuvant can induce helper T cell 1 (Th1) response in human body. Its immune response effect is better than that of aluminum adjuvant, which mainly induces helper T cell 2(Th2) response.

1.3.2 INTRODUCTION OF SCTV01E

The quadrivalent SARS-CoV-2 trimeric spike protein vaccine (code name SCTV01E) is a recombinant protein vaccine developed by Sinocelltech Ltd., with genetic engineering technology adopted to express in CHO cells.

SCTV01E contains the mixture of TM22, TM23, TM28 and TM41 recombinant proteins combined with SCT-VA02B adjuvant, formulated with sodium citrate, sodium chloride and other excipients, without preservatives and antibiotics. The adjuvant (SCT-VA02B (1×)) is an oil-in-water adjuvant, whose composition, formula, process and main quality control (such as particle size) are consistent with those of the commercial marked MF59 adjuvant which was introduced in vaccine since 1997. Adjuvant like MF59® can induce helper T cell 1 (Th1) response in human body. Its immune response effect is better than that of aluminum-based adjuvant, which mainly induces helper T cell 2(Th2) response. The TM22, TM23, TM28 and TM41 antigens used in SCTV01E are the recombinant Spike-ECD trimeric proteins produced in classic CHO platform, which amino acid sequences are from Alpha, Beta, Delta and Omicron variants.

SCTV01E has identical trimeric protein molecular design with SCTV01C. Using the same CHO cell production platform technology, SCTV01E has highly similar quality (high purity, low impurity), and all four variants share high sequence homology. SCTV01E's immunogenicity is similar to SCTV01C in terms of total IgG titer so is not expected to have different safety profile. Based on the excellent nonclinical safety of SCTV01C, the similarity among the four antigens and the lower clinical dose, SCTV01E is equivalent to SCTV01C in

terms of vaccine safety and no safety risk is expected for SCTV01E. SCTV01E dose will not exceed the high dose of SCTV01C tested in Phase I.

SCTV01E is specifically designed for the prevention of infection with SARS-CoV-2 and its variants. SCTV01E make a difference to the Delta and Omicron variants due to higher NAT50 titer: SCTV01E trimeric subunit protein consists of the ECD region of S protein and T4-foldon. SCTV01E, simulating S protein, has complete biological function and natural structural properties, which ensures the high proportion of neutralizing epitope of correct structure and the induction of neutralizing antibodies with high titers. At the same time, SCTV01E has more T cell epitopes and can induce stronger resistance to variants infection than the RBD protein vaccines due to 5 time larger in its molecular weight.

1.4 SUMMARY OF PRECLINICAL STUDY

1.4.1 NON-CLINICAL PHARMACOLOGY

1.4.1.1 In Vitro Efficacy Study

The structural frame of all SCTV01C and SCTV01E trimeric protein consists of the extracellular domain region of S protein (S-ECD, including S1 and S2 part) and T4-foldon.

S-ECD trimer protein removes the Furin cleavage site between S1 and S2 to solve the stability problem caused by the cleavage. The S-ECD is made with a fusion with T4-foldon (C-terminal of phage fibrin) to promote the formation and stability of trimer. SCTV01C and SCTV01E trimer proteins, retaining of their natural structural properties, having complete biological functions and correct conformational characteristics, can produce effective neutralizing antibodies with multiple epitopes, which are featured with the protein structural basis of protection against virus infection[3].

1.4.1.2 In Vivo Efficacy Study

Immunogenicity Study

Immunogenicity study shows that SCTV01C and SCTV01E can induce a high level of humoral immune response in female C57BL/6J mice, and the neutralization activity against antigen related/unrelated strains of COVID19 and Th1/Th2 immune response induced are better than monovalent vaccines. The humoral immune response and Th1/Th2 immune response induced by the combination of SCTV01C or SCTV01E with adjuvant SCT-VA02B are significantly better than the control adjuvant.

SCTV01C and SCTV01E shows high level of the humoral immune response in female SD rats (200-220g) in the dose range of 20μg ~40μg. The neutralizing antibodies titers are increase following with the increase of the level of adjuvant. When testing at 40μg dose level, the

highest immunogenicity is detected in the high-dose adjuvant group (10 mg/rat), and the non-adjuvant group shows the lowest immunogenicity. The specific total IgG titers of SCTV01C and SCTV01E showed the same level in rats 1 week after second administration of equal total antigen dose in the dose range of 20μg ~ 40μg.

In addition, SCTV01C-TM23 and SCTV01E-TM23 shows strong humoral immune response and T cell response in cynomolgus monkeys.

Virus-Challenge and Attacking Protection Efficacy in Mice

As a demonstration of feasibility and working mechanism, a virus-challenge study was conducted with the bivalent vaccine (covering Alpha and Beta variants). The load of hACE2-KI/NIFDC murine pneumonia virus was reduced by $10^{5.32}$ times in the bivalent vaccine group (SCTV01C, the first generation of vaccine), no viral RNA was observed in the lung tissue, and no antibody mediated infection enhancement (ADE) and vaccine-enhanced disease (VED) were observed. The results showed that the bivalent vaccine had a clear protective effect against South African variant of novel coronavirus. The results also showed that SCTV01C-TM23 monovalent vaccine had a clear protective effect against South African (Beta) variant, and had a cross-protection effect on Beijing prime strain infection.

Virus-Challenge and Attacking Protection Efficacy in Hamster

The load of hamster pneumonia virus was reduced by 103.5 and 103.46 times in the SCTV01C and Alpha+Beta+Delta vaccine respectively, and significantly improving the weight loss caused by challenge (2-13 dpi) without ADE and VED. The results showed that SCTV01C and Alpha+Beta+Delta vaccine had good immunogenicity and clear cross-protection against prototype strain in hamster model.

The clinical dose of SCTV01E is 30 μg in total antigen, which is within the clinical dose range tested for SCTV01C (20-40 μg). The S-trimers of Alpha, Beta, Delta and Omicron have >96% amino acid homology, which means that SCTV01E not only should have the same safety profile, but also could induce similar amount of total S-specific IgG as SCTV01C under the same immunization dose, but with broader neutralizing antibodies against more variants than SCTV01C. This argument was validated in mice immunization study. Thus, SCTV01E is apparently more effective regarding the induction of potent neutralizing activities against Delta and Omicron variants than SCTV01C. According to the consensus mechanism of action of the SARS-CoV-2 vaccine, high neutralization titer can effectively prevent virus infection, whereas high T cell response can prevent serious illness. SCTV01E is expected to have the same safety profile as and higher protective efficacy than SCTV01C, particularly reducing the severe disease induced by Omicron variants. Therefore, with the virus constantly changing currently,

companies are constantly developing new vaccines in order to adapt to the changes in the virus. Speed is the vitality of drug research and development. The SCT evaluated that SCTV01E has no risk of challenge protection and hoped that the company can refer to the development strategy of influenza vaccine and use immunogenicity as the basis of effective protection to reduce the company's research and development time.

1.4.2 TOXICOLOGY

The active components of SCTV01E contain four recombinant proteins, named TM22 (Alpha), TM23 (Beta), TM28 (Delta) and TM41 (Omicron) proteins, which are homotrimeric proteins with a few mutated residues difference on RBD region for TM22, TM23 and TM28, and some mutant sites difference on the spike protein between TM41 and other antigens. The 1st generation bivalent vaccine SCTA01C (TM22 and TM23) has been successfully applied for clinical use in 2021. The toxicology evaluation of SCTV01E has been completed, including single dose toxicity study, repeat dose toxicity study, anaphylaxis study and local tolerance, also showing the good safety profile.

1.4.2.1 Single Dose Toxicity Test of SCTV01C and SCTV01E

SCTV01C

There were no mortality or morbidity observed, no abnormal reaction observed. The study results show no acute toxicity, and the maximum tolerated dose (MTD) in rats is ≥4 doses/rat (240 μg/rat).

SCTV01E

There were no mortality or morbidity observed, no abnormal reaction observed. The study results show no acute toxicity, and the maximum tolerated dose (MTD) in rats is ≥4 doses/rat (120 μg/rat).

1.4.2.2 Repeat Dose Toxicity Test of SCTV01C

SCTV01C

The decrease of Retic and CHO was thought to relate to the adjuvant, the increase of Ca was thought to be associated with the test sample, while other indicators were thought to be related to the acute phase reaction and/or immune response caused by administration. The changes, except for Alb, Glb and A/G, have fully disappeared at the end of the recovery period. There was no obvious systemic toxicity associated with administration.

SCTV01E

Some changes were noted, including increases in Eutrophilic (Neut), Eosinophils (Eos), Fibrinogen (FIB), and Globulin (Glb), decreases in Reticulocyte (Retic), Albumin (Alb), increased cellularity of red pulp in the spleen and plasmacytosis in the inguinal lymph node,

which were considered to be related to the acute phase response or immune response after administration. Glomerulonephritis in the kidney was found in 2/20 animals in the 3 doses/animal group, which may be related to strong antibody titer. The above changes showed a recovery trend 2 weeks after dosing completed and no obvious systemic toxicity was observed. The no observed adverse effect level (NOAEL) is considered to be 1 dose (30 μg)/rat.

1.4.2.3 Immunogenicity Test of SCTV01C

SCTV01C

A robust immune response can be induced in SD rats by repeated intramuscular injection of SCTV01C used as a model for SCTV01E at 1 dose/rat and 3 doses/rat for 6 weeks. The titers of anti-TM22 and anti-TM23 antibodies increased following with the increase of doses, showing a dose correlation, and the geometric mean titers (GMT) of the low and high dose groups reached the peak at Week 2 and Week 1 in recovery period, respectively. The similar trend was observed in neutralizing test in which the titers of neutralizing antibodies against the pseudoviruses of SARS-CoV-2 variants increased as the administration doses went up. GMT of both low and high dose groups reached the peak at Week 1 in recovery period.

SCTV01E

A robust immune response can be induced in SD rats by repeated intramuscular injection of SCTV01E at 1 dose/rat and 3 doses/rat for 6 weeks. The titers of anti-TM22, anti-TM23, anti-TM28 and anti-TM41 antibodies increased with administration times, showing a dose-dependent manner on Day 14 and then almost saturated. The GMTs against proteins of mutated strains in the low and high dose groups reached the peak prior to the third dosing (Day 28) or on the 1st week of the recovery period (Day 50). As for different SARS-CoV-2 variant pseudoviruses, the neutralizing antibodies increased with administration times, showing a consistent trend. The neutralizing antibody titers of the low and high dose groups against the variant strains basically reached peak on the 1st week of recovery period (D50).

1.4.2.4 Immunotoxicity Test

SCTV01C

No immunotoxicity was observed after repeated intramuscular injection with SCTV01C used as the model for SCTV01E at 1 dose/rat and 3 doses/rat in SD rats for 6 weeks (4 times in total). There was no abnormality related to drug administration in macroscopic observation of the lymphatic organs/tissues of the animals microscopic observation of thymus, bone marrow and so on. No notable change of peripheral T lymphocyte subsets distribution and the weight of thymus were observed in each group.

SCTV01E

No immunotoxicity was observed after repeated intramuscular injection with SCTV01E at 1 dose/rat and 3 doses/rat in SD rats for 6 weeks. No abnormalities related to administration were noted in macroscopic examination of lymphoid organs/tissues, thymus weight, and histopathological examination of thymus, bone marrow and so on. The above changes were restored or had a recovery trend at the end of the 2-week recovery period on Day 57.

1.4.2.5 Local Tolerance and Other Toxicity Studies

SCTV01C

The local irritation study of SCTV01C on SD rats showed that no abnormal reactions such as erythema, hyperemia, swelling, ulcer and induration were observed locally in each group and no abnormalities were observed in necropsy during the experiment. The local irritation study in New Zealand rabbits showed that there was no abnormal reaction such as swelling, fever, edema, erythema, hyperemia and ulcer in the local site of the drug administration in each group. The local tolerance was evaluated first with SCTV01C with the TM23 produced in stable pool-cell process combined with repeat-dose toxicity study in rats, and then two batches of SCTV01C with the TM23 produced in stable pool-cell process or stable clonal-cell process were evaluated and compared in a single local tolerance test in rabbits, with consistent results between two batches. The microscopic examination results showed that minimal/slight to moderate mixed inflammation were observed in all animals on the placebo control side, CHO-pool product side and stable CHO clone product side, which were considered to be related to the local irritation induced by the adjuvant placebo. All related abnormal changes were partially disappeared after two weeks recovery.

The sensitization was induced by intramuscular injection of SCTV01C at 0.1 dose/animal and 1 dose/animal and simulated by intravenous injection at 0.2 dose/animal and 2 doses/animal, respectively. No active systemic anaphylaxis was observed in guinea pigs.

Reproductive toxicity test, which have not been conducted yet, will be carried out according to the subsequent clinical trial plan.

According to both the ICH guideline and the *General Principles for Technical Review of Preclinical Safety Evaluation of Biological Products for Prophylaxis* issued by National Medical Products Administration (NMPA) in 2008[4], vaccines generally do not require genotoxicity tests, carcinogenicity tests, dependence tests and routine pharmacokinetic studies. And hemolysis study is also not applicable for a recombinant protein vaccine which is administrated by intramuscular injection.

SCTV01E

The local irritation study was also conducted in New Zealand Rabbits after repeated

intramuscular administration of SCTV01E at 1 dose (30 μg)/animal once weekly for 2 weeks (total of 3 times), followed by a 2-week recovery period. No abnormal findings related to administration in daily clinical observations and injection site observations were noted in any groups during the study. Histopathological examination showed that adjuvant related irritation reactions including interstitial mixed inflammation and/or hemorrhage were observed at the injection sites, and showed a recovery trend after 2 weeks of the last dosing.

1.5 CLINICAL SAFETY OF SCTV01C

A Phase I/II clinical trial to evaluate immunogenicity and safety of SCTV01C in population unvaccinated with COVID-19 vaccine and aged ≥ 18 years old, has been initiated in China. The first participant was enrolled on December 01, 2021. As of December 23, 2021, 65 participants were enrolled into Phase I (planned 112): 12 (18.4%) of them reported AEs, including solicited local and systemic AEs and unsolicited AEs. The blinded data shows that all of these AEs were Grade 1 or 2. There was no ≥ Grade 3 AE observed, and no SAE or AESI either. The most common adverse reactions were injection site pain (8 participants, 12.3%). Other solicited local AEs reported by participants were induration (1, 1.5%), redness (1, 1.5%) and swelling (1, 1.5%). The solicited systemic AEs reported by participants were fever (1, 1.5%), anxiety (1, 1.5%), fatigue (1, 1.5%), and diarrhea (1, 1.5%).

1.6 STUDY SIGNIFICANCE

The purpose of this study is to evaluate the safety and immunogenicity of SCTV01C and SCTV01E in preventing COVID-19 caused by SARS-COV-2 and its concerning variants (VOC) infection, particularly Delta and Omicron, in populations aged 18 years and older. The outbreak and pandemic of COVID-19 have caused serious impact and challenge to the global health care system and posed a serious threat to human survival and health. The nearly all currently vaccine marketed or EUA approved design is basically based on the sequence of the early epidemic strain (Wuhan strain), while SARS-COV-2 is an RNA single-stranded virus that is prone to deletion mutation. Given with fast spread of high-risk mutant strains such as Delta and Omicron, which had the higher transmissibility of the epidemic, aggravate the development of the disease and severely reduce the protective effect of neutralization of antibodies generated by prior infection or immunization, the rapid development of the second-generation vaccines with high protection against those high-risk variants is of utmost importance.

1.7 ASSESSMENT OF BENEFIT/RISK

1.7.1 RISK ASSESSMENT

1.7.1.1 The Risk of Study Vaccine Administration

While the results of preclinical study of SCTV01C have shown the favorable safety profile and immunogenicity, and from mechanistic perspective, supporting the direct use of SCTV01E, and safety risk of SCTV01E has been estimated to be low, there is no long-term study showing persistence of neutralization antibodies, against any individual variants. Though there are some safety data from the on-going clinical trials with SCTV01C, there are no data available from clinical trials on the use of SCTV01E vaccines in humans at the outset of this study. Meanwhile, as with other vaccines, there is a potential risk of allergic reaction, and this vaccine may not be effective for all vaccinated people.

1.7.1.2 Vaccine-enhanced disease

The mechanism of ADE/VED is unclear, and there are no specific clinical indications or laboratory indicators for clinical diagnosis, but ADE/VED is somewhat associated with non-neutralizing antibodies. ADE effects during Fc receptors cell recognition and internalization of non-neutralizing virus-antibody complex will lead to increased viral ingestion and aggravation of viral infection. VED may occur via ADE, activation of leukocyte differentiation antigen 4 positive (CD4+) memory T cells, Th2 deviation or abnormal T cell, which will enhance the viral transfection and replication. While no ADE/VED due to SCTV01C intervention was shown in the nonclinical findings, risk control measures should be developed in clinic and all participants should be closely monitored for VED and followed up throughout the study.

1.7.1.3 Risk of Biological Sample Collection

Venipuncture is a routine clinical procedure adopted by the medical community to collect blood samples. Immediate complications may include mild pain during skin piercing and uncommon dizziness and syncope. In addition, venipuncture may cause hematoma with low risk. Skin/soft tissue infection may occur in the puncture point, vein or the blood flow but in a low change. The risk associated with the nasal/pharyngeal/throat swab collection process is low. Some people may cough and sneeze briefly during and after the swab, and a few may experience irritation in the nasal passages or may bleed slightly.

Researchers will strictly supervise qualified and experienced medical personnel or health workers trained to collect venous blood samples and nasal/pharyngeal/throat swab in accordance with prescribed procedures to minimize the pain and risk to the participants (including local pain and the low probability of venipuncture site infection and nasal mucosal damage).

1.7.1.4 Exposure during Pregnancy

At present, no reproductive toxicity test has been done. In this study, a contraceptive period is set according to the experience of the marketed recombinant protein vaccine, that is, effective contraceptive measures shall be taken by the participants entering the clinical trial from the signing of informed consent to 6 months after vaccination.

Examples of acceptable forms of highly effective contraception include:

- Combined (estrogen and progestogen containing) hormonal birth control that prevents ovulation (oral, intravaginal, transdermal)
- Progestogen-only hormonal birth control that prevents ovulation (oral, injectable, implantable)
- Intrauterine device or intrauterine hormone-releasing system
- Bilateral tubal occlusion and ligation
- Sterilized sexual partner with documented absence of sperm Abstinence (Avoid sexual intercourse)

During this period, the investigator will keep contact with the participants to determine whether pregnancy and related complications occur.

1.7.2 BENEFIT ASSESSMENT

All participants will undergo a physical examination (including but not limited to routine physical examination, vital signs and SARS-COV-2, etc.) and receive the examination results free of charge.

Participation in this study will result in a better understanding of COVID-19, leading to better prevention measures. If SCTV01C and SCTV01E is successful in preventing COVID-19, it will provide an effective way against the virus and make a significant contribution to global public health progress.

1.7.3 ASSESSMENT OF OVERALL BENEFIT-RISK

Control measures against potential risks such as allergic reaction and ADE/VDE will be developed prior to the start of the clinical trial, and all participants will be closely monitored and followed in strict compliance with inclusion and exclusion criteria to ensure timely management and maximum benefit for participants at risk. The investigators will be trained for participant follow-up and safety data collection to ensure the timely updating of adverse events. The independent Data and Safety Monitoring Board (DSMB) will review the safety data collected throughout the study, conduct periodic or temporary meetings to assess risk and benefit, and make recommendations to sponsors for the safety concerns. In summary, the

anticipated risks associated with SCTV01E are expected to be manageable. Given the great potential of COVID-19 prevention, the profile of this vaccine candidate supports initiation of this Phase III clinical trial.

2 STUDY OBJECTIVES AND ENDPOINT

2.1 STUDY OBJECTIVES

Primary Objective:

- To evaluate the immunogenicity of SCTV01C;
- To evaluate the immunogenicity of SCTV01E;

Secondary Objective:

- To evaluate the cellular immune response of SCTV01C;
- To evaluate the cellular immune response of SCTV01E;
- To evaluate the safety of SCTV01C within 180 days after the vaccination.
- To evaluate the safety of SCTV01E within 180 days after the vaccination.

2.2 STUDY ENDPOINT

Primary endpoints

Cohort 1

Immunogenicity

- GMT of nAb against Delta variant on D28.
- GMT of nAb against Omicron BA.1 variant on D28.

Cohort 2

Immunogenicity

- GMT of nAb against Omicron BA.1 variant on D28.
- GMT of nAb against Delta variant on D28.

Secondary endpoints:

Cohort 1

Immunogenicity

- GMT of nAb against Delta variant on D180.
- GMT of nAb against Omicron BA.1 variant on D180.
- GMT of nAb against Omicron BA.5 variant on D28.
- Number of IFN-γ positive (characterizing Th1) and IL-4 positive (characterizing Th2) T cell subsets on D28.
- Seroresponse of nAb (defined as a change from below the low limit of quantitation [LLOQ] to equal to or above LLOQ, or a ≥4-fold rise if baseline is equal to or above LLOQ in nAb to Delta variant from D0) rates on D28.

- Seroresponse of nAb (defined as a change from below LLOQ to equal to or above LLOQ, or a ≥4-fold rise if baseline is equal to or above LLOQ in nAb to Omicron variant from D0) rates on D28.

Safety

- Incidence and severity of solicited AEs of SCTV01C from D0 to D7.
- Incidence and severity of all unsolicited AEs of SCTV01C from D0 to D28.
- Incidence and severity of SAEs and AESIs of SCTV01C within 180 days.
- Incidence and severity of solicited AEs of SCTV01E from D0 to D7.
- Incidence and severity of all unsolicited AEs of SCTV01E from D0 to D28.
- Incidence and severity of SAEs and AESIs of SCTV01E within 180 days.

Cohort 2

Immunogenicity

- GMT of nAb against Delta variant on D180.
- GMT of nAb against Omicron BA.1 variant on D180.
- GMT of nAb against Omicron BA.5 variant on D28.
- Number of IFN-γ positive (characterizing Th1) and IL-4 positive (characterizing Th2) T cell subsets on D28.
- Seroresponse of nAb (defined as a change from below the low limit of quantitation [LLOQ] to equal to or above LLOQ, or a ≥4-fold rise if baseline is equal to or above LLOQ in nAb to Delta variant from D0) rates on D28.
- Seroresponse of nAb (defined as a change from below LLOQ to equal to or above LLOQ, or a ≥4-fold rise if baseline is equal to or above LLOQ in nAb to Omicron variant from D0) rates on D28.

Safety

- Incidence and severity of solicited AEs of SCTV01C from D0 to D7.
- Incidence and severity of all unsolicited AEs of SCTV01C from D0 to D28.
- Incidence and severity of SAEs and AESIs of SCTV01C within 180 days.
- Incidence and severity of solicited AEs of SCTV01E from D0 to D7.
- Incidence and severity of all unsolicited AEs of SCTV01E from D0 to D28.
- Incidence and severity of SAEs and AESIs of SCTV01E within 180 days.

3 STUDY DESIGN

Many studies have shown that the neutralizing abilities against SARS-CoV-2 variants decreased substantially after a period time from the initial fully vaccination mainly because of decreased antibody titers, which justify the needs of booster dose[5-9]. In terms of the time

interval between the booster dose and the last dose of the initial vaccination, most of the studies chose more than 3 to 6 months as the interval time[10-13]. Considering participants safety and the ongoing SARS-CoV-2 vaccine booster studies, we choose ≥3 months and ≤12 months post the last dose administration of the initial vaccination as one of the inclusion criteria.

3.1 STUDY DESIGN

Although there is no clinical data for SCTV01E so far, SCT had initiated three clinical Phase I/II trials for SCTV01C to evaluate the safety and immunogenicity, which can be instructive and meaningful for SCTV01E clinical study consideration because of the same manufacturing processes, extremely similar molecular characteristics and clinical dosing between SCTV01E and SCTV01C. The SCTV01C trials will provide sufficient supportive safety and immunogenicity clinical data prior to the start of SCTV01E trials. The details of these trials are summarized in investigator's brochure.

SCTV01E, the quadrivalent vaccine, will be tested in this Phase III immunogenicity study based on the clinical data on safety, reactogenicity, and immunogenicity generated with the bivalent vaccine (SCTV01C) similarity in manufacturing process for four TM (trimeric drug substance) components of the quadrivalent product compared to the bivalent product; similarity in construct design supporting a similar safety profile of the quadrivalent product to that of the bivalent vaccine. The dose strength of SCTV01E is 30μg (5/5/5/15μg for TM22/TM23/TM28/TM41)/dose based on the nonclinical study of SCTV01E and SCTV01C in combination with the clinical studies of SCTV01C.

The study is a randomized, double-blind, and positive-controlled Phase III booster study. It will evaluate the immunogenicity and safety of one dose of SCTV01C or SCTV01E as booster compared with either one dose of Sinopharm inactivated COVID-19 vaccine (Cohort 1) or one dose of mRNA COVID-19 vaccine (Cohort 2).

Approximately 1,800 participants aged 18 years old and above will be enrolled in this study. 1,350 participants who previously received Sinopharm inactivated COVID-19 vaccine will be enrolled to Cohort 1. 450 participants who previously received mRNA COVID-19 vaccine (Comirnaty from Pfizer or mRNA-1273 from Moderna) or previously diagnosed with COVID-19 will be enrolled to Cohort 2.

In Cohort 1, 300 participants who were previously fully vaccinated with 2 or 3 doses of Sinopharm inactivated COVID-19 vaccine and with no previous COVID-19 history will form an immunogenicity subgroup (Subgroup 1) for nAb tests, and will be randomly assigned to SCTV01C Group, SCTV01E Group and the Sinopharm inactivated COVID-19 vaccine Group in a ratio of 1:1:1. The 300 participants for nAb tests will be stratified by age (18-54 years, ≥55

years) , number of doses of previously received COVID-19 vaccines (2, 3), and interval between previous vaccination and the study vaccination (3-5 months, 6-8 months, 9-12 months, 13-24 months).The first 150 participants will form a cellular immune response subgroup for cellular immune response tests.

In Cohort 1, in addition to the 300 participants for immunogenicity tests, there are 1050 other participants who previously received at least one shot of Sinopharm COVID-19 inactivated vaccine, will form a subgroup (Subgroup 2) mainly for safety observation, and will be randomly assigned to SCTV01C Group, SCTV01E Group and Sinopharm inactivated COVID-19 vaccine Group in a ratio of 1:1:1. The 1050 participants mainly for safety observation will be stratified by age (18-54 years, ≥55 years), previous COVID-19 infection history (yes or no), number of doses of previously received COVID-19 vaccines (1, 2, 3) and interval between previous vaccination and the study vaccination (3-5 months, 6-8 months, 9-12 months, 13-24 months).

In Cohort 2, 450 participants who previously received 2 or 3 doses of mRNA COVID-19 vaccine (Comirnaty or mRNA-1273) or previously diagnosed with COVID-19 will be randomly assigned to SCTV01C Group, SCTV01E Group and the mRNA COVID-19 vaccine Group in a ratio of 1:1:1. Participants will be stratified by age (18-54 years, ≥55 years), number of doses of previously received COVID-19 vaccines (0, 1, 2, 3), previous COVID-19 infection history (yes or no), and interval between previous vaccination and the study vaccination (3-5 months, 6-8 months, 9-12 months, 13-24 months). In Cohort 2, the number of participants previously diagnosed with COVID-19 and previously not received any mRNA COVID-19 vaccine, should not be more than 50. All participants will have nAb tests. The first 150 participants will form a cellular immune subgroup for cellular immune response tests.

In Cohort 1, each participant in SCTV01C Group will receive one dose of SCTV01C on D0; each participant in SCTV01E Group will receive one dose of SCTV01E on D0; each participant in Sinopharm inactivated COVID-19 vaccine Group will receive one dose of Sinopharm inactivated COVID-19 vaccine on D0.

In Cohort 2, each participant in SCTV01C Group will receive one dose of SCTV01C on D0; each participant in SCTV01E Group will receive one dose of SCTV01E on D0; each participant in mRNA COVID-19 vaccine Group will receive one dose of mRNA COVID-19 vaccine on D0.

Trial procedures:

The study procedure is described as Figure 1 and Figure 2. An independent data and safety monitoring board (DSMB) will review the data of the study.

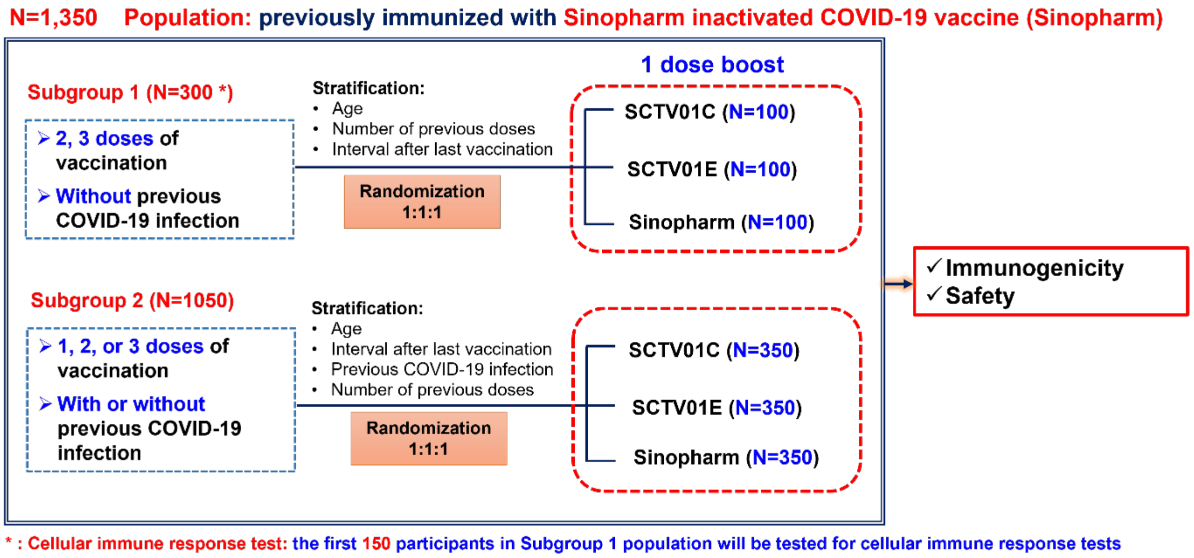

Figure 1 Study design for Cohort 1

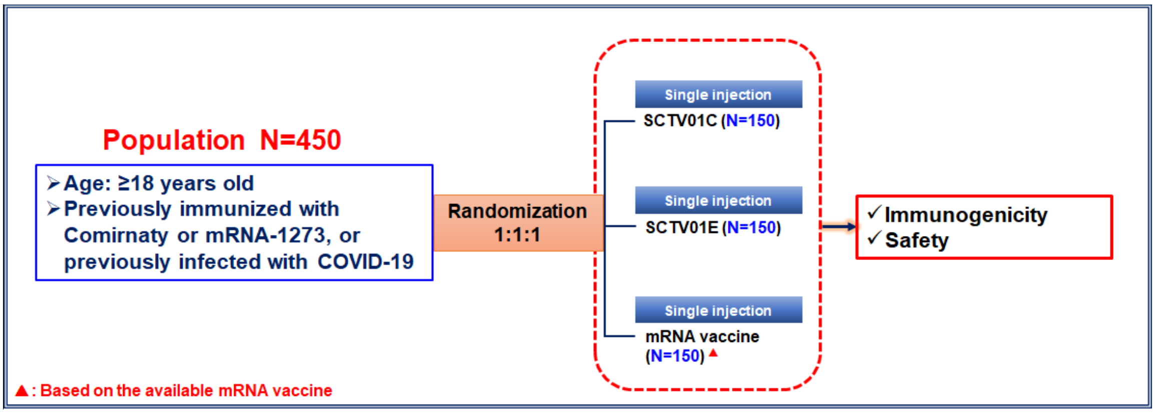

Figure 2 Study design for Cohort 2

The study consists of a screening period, a randomization and vaccination period, and a follow-up period.

Screening period: After participants sign the ICF, the screening phase visit will be conducted within 14 days.

Randomization: The qualified participants will be randomized before the study vaccination.

Vaccination: Randomized participants will be vaccinated on D0.

Follow-up:

Safety follow-up: The participants will be observed at site for at least 30 minutes after the study vaccination. Both the active monitoring and the spontaneous reporting will be used to collect the solicited and the unsolicited AEs. Solicited AEs within 7 days after study vaccination and unsolicited AEs within 28 days after study vaccination will be collected through vaccination record cards. SAEs and AESIs will be followed for 180±7 days after the study vaccination.

Immunogenicity follow-up: The participants in Subgroup 1 in Cohort 1 and all participants in Cohort 2 will be sampled for immunogenicity on D0 (before vaccination), D28 and D180. The nAb against Delta, Omicron variants and other variants will be tested.

The participants in the cellular immune response subgroup will be sampled for cellular immune response test on D0 (before vaccination) and D28.

After administration of the study vaccination, the participants will be continuously and systematically monitored for 180±7 days to ensure a prompt diagnosis and treatment according to FDA diagnosis and treatment practice when a participant experiences the suspicious symptoms of COVID-19. If a SARS-CoV-2 infection is confirmed 14 days after the study vaccination, sample will be collected from the nasopharyngeal/throat swab and viral sequencing will be used to identify the major SARS-CoV-2 variants.

The DSMB will review the safety data within D0-D28 of all the participants to assess the safety SCTV01C and SCTV01E.

Note:

Participants aged between 54 years to less than 55 years old will be taken as 54 years old.

3.2 BLINDING AND UNBLINDING METHODS

3.2.1 RANDOMIZATION

This clinical trial is a randomized, double-blind Phase III clinical trial. Participants are randomly assigned into the different groups according to the ratio specified in the protocol. In order to achieve the relative balance among each group, the participants will be stratified by specified stratification factors. The detail will be described in the randomization plan.

The eligible participants will be randomized by the Interactive Network Response System (IWRS) and vaccinated according to the random number. For randomized participants who withdraw from clinical trials for any reason, regardless of whether they have received the study vaccine, their random numbers will be retained, and participants who withdrew can no longer participate in this trial.

3.2.2 BLINDING AND UNBLINDING METHODS

3.2.2.1 Blinding

This study is randomized and double-blind. An unblinded team at the study site will be set. The unblinded study site personnel will manage vaccine logistics, preparation, and administration so as to maintain the blind from the remainder of the study site personnel and participants. The unblinded study site personnel administer study vaccine, but will not be involved in study-related assessments or have participant contact for data collection after administration of trial

vaccine.

Unblinded analysis by independent unblinded statistician can be performed after all participants have completed the safety assessment 28 days after the study vaccination.

When the experiment is in progress, the unblinded statistician shall ensure that the random blind codes are properly kept and handed over to the blind statistician to save with other project-related materials when the study is completed.

3.2.2.2 Unblinding

The study will be in an EDC/IWRS database. The sponsor and investigator will determine the time for unblinding in each phase of the clinical trial based on the progress of the study. Before unblinding, the principal investigator, sponsor and statistician must jointly sign relevant documents.

3.2.2.3 Unblinding under emergency

If there are serious adverse events or emergencies during the trial, when the investigator believes that it is essential to know the participant's group for his clinical treatment or health, it can be unblinded in emergency. The principal investigator or his designated person in charge should contact the sponsor directly to discuss the necessity of unblinding under emergency. After the sponsor confirms, unblinding under emergency of individual participants can be carried out. The investigator or his designated person in charge can apply for unblinding under emergency at IWRS and record the reason for unblinding. The participant will withdraw from the trial, and the reason for withdrawal must be recorded in the original data.

3.3 DEFINITION OF END OF STUDY

Definition of end of the study for individual participant: the participant completes all the visits specified in the protocol or terminates the study early due to various reasons.

Definition of end of study: the last participant planned to be enrolled completes the last visit specified in the protocol or terminates the study early due to various reasons.

4 STUDY POPULATION

4.1 INCLUSION CRITERIA

Participants are eligible to be included in the study only if the following conditions are met:

1. Male or female aged ≥18 years old when signing ICF;
2. **For Subgroup 1 in Cohort 1:** Participants who were previously vaccinated with 2 or 3 doses of Sinopharm inactivated COVID-19 vaccine. The interval between the date of last dose and the date of this study vaccination should be 3 to 24 months.

   For Subgroup 2 in Cohort 1: 1) Participants who were previously vaccinated with 2 or 3 doses of Sinopharm inactivated COVID-19 vaccine, with or without COVID-19 history;

or 2) Participants who were previously vaccinated with 1 dose of Sinopharm inactivated COVID-19 vaccine and previously diagnosed with COVID-19. The interval between the date of last dose/COVID-19 diagnosis and the date of this study vaccination should be 3 to 24 months.

For Cohort 2: 1) Participants who were previously vaccinated with 2 or 3 doses of mRNA COVID-19 vaccine (Comirnaty or mRNA-1273), with or without COVID-19 history; or 2) Participants who were previously vaccinated with 1 doses of mRNA COVID-19 vaccine (Comirnaty or mRNA-1273) and previously diagnosed with COVID-19; or 3) Participants who were previously not vaccinated with any COVID-19 vaccine and previously diagnosed with COVID-19. The interval between the date of last dose/COVID-19 diagnosis and the date of this study vaccination should be 3 to 24 months.

3. The participant and/or his legally acceptable representative can sign written ICF, and can fully understand the trial procedure, the risk of participating in the trial, and other interventions that can be selected if they do not participate in the trial;
4. The participant and/or his legally acceptable representative have the ability to read, understand, and fill in record cards;
5. Healthy participants or participants with pre-existing medical conditions who are in stable condition. The “pre-existing medical conditions” include but not limited to hypertension, diabetes, chronic cholecystitis and cholelithiasis, chronic gastritis that meet the described criteria. A stable medical condition is defined as disease not requiring significant change in therapy or no need for hospitalization as a consequence of worsening disease state for at least 3 months prior to enrollment;
6. Fertile men and women of childbearing potential voluntarily agree to take effective contraceptive measures from signing ICF to 6 months after the study vaccination; the pregnancy test results of women of childbearing potential are negative on screening.

4.2 EXCLUSION CRITERIA

A participant who conforms to any of the following criteria should not be enrolled in the study:

1. **For Subgroup 1 in Cohort 1 only**: Previously diagnosed with COVID-19.
2. Presence of fever within 3 days before the study vaccination;
3. A history of infection or disease related to severe acute respiratory syndrome (SARS), Middle East respiratory syndrome (MERS), or other disease corresponding use of immunosuppressants;
4. A history of allergic reactions to any vaccine or drug, such as allergy, urticaria, severe skin eczema, dyspnea, laryngeal edema, and angioneurotic edema;

5. A medical or family history of seizure, epilepsy, encephalopathy and psychosis;
6. Immunocompromised patients suffering from immunodeficiency diseases, important organ diseases, immune diseases (including Guillain-Barre Syndrome [GBS], systemic lupus erythematosus, rheumatoid arthritis, asplenia or splenectomy caused by any circumstances, and other immune diseases that may have an impact on immune response in the investigator's opinion), etc.;
7. Long-term use of immunosuppressant therapy or immunomodulatory drugs for ≥14 days within the first six months prior to enrollment. Whereas short-term (≤14 days) use of oral, inhaled and topical steroids are allowed;
8. Patients on antituberculosis therapy;
9. Presence of severe or uncontrollable cardiovascular diseases, or severe or uncontrollable disorders related to endocrine system, blood and lymphatic system, liver and kidney, respiratory system, metabolic and skeletal systems, or malignancies (skin basal cell carcinoma and carcinoma in-situ of cervix are exceptions and will not be excluded), such as severe heart failure, severe pulmonary heart disease, unstable angina, liver failure, or uremia;
10. Contraindications for intramuscular injection or intravenous blood sampling, including thrombocytopenia and other blood coagulation disorders;
11. Participants who received any immunoglobulin or blood products in the previous 3 months before enrollment, or plan to receive similar products during the study;
12. Participants who received other investigational drugs within 1 month before the study vaccination;
13. Participants who is at the acute state of disease, such as acute onset of chronic heart failure, acute sore throat, hypertensive encephalopathy, acute pneumonia, acute renal insufficiency, acute cholecystitis;
14. Participants received other drugs or vaccines used to prevent COVID-19, but participants previously received Sinopharm inactivated COVID-19 vaccine, Comirnaty or mRNA-1273 will not be excluded;
15. Participants vaccinated with influenza vaccine within 14 days or with other vaccines within 28 days before the study vaccination;
16. Those who donated blood or had blood loss (≥450 mL) within 3 months before the vaccination or plan to donate blood during the study period;
17. Those who are pregnant or breast-feeding or plan to be pregnant during the study period;
18. Those who plan to donate ovum or sperms during the study period;

19. Those who cannot follow the trial procedures, or cannot cooperate to complete the study due to planned relocation or long-term outing;
20. Those unsuitable for participating in the clinical trial as determined by the investigator because of other abnormalities that are likely to confuse the study results, or non-conformance with the maximal benefits of the participants;
21. Those who are tested positive for HIV in terms of serology.

5 DISCONTINUATION OF STUDY INTERVENTION

5.1 WITHDRAWAL CRITERIA

A participant may withdraw from the study at any time at his/her own request.

Reasons for discontinuation from the study may include the following:

- Refused further follow-up;
- Lost to follow-up;
- Death;
- Study terminated by sponsor;
- AEs;
- Participant request;
- Investigator request;
- Protocol deviation.

Handling participants withdrawing:

If a participant withdraws consent, the investigator must make every effort to determine the primary reason for this decision and record this information on the treatment disposition eCRF page. If the participant decides to completely withdraw from the study (refuses any further study participation or contact), all study participation for that participant will cease and data to be collected at subsequent visits will be considered missing. Further attempts to contact the participant are not allowed unless safety findings require communication or follow-up.

Participants may refuse further procedures (including vaccination) but are encouraged to remain in the study for safety follow-up. In such cases where only safety is being conducted, participant contact could be managed via phone call, short message, email or other contacting method.

If a participant withdraws from the study or terminates the study (including loss to follow-up) after enrollment, no participant replacement is allowed.

Participants will be informed of the importance of continuing to take other public health measures, such as social distancing, wearing masks and washing hands, to control the spread

of the disease.

5.2 STUDY SUSPENSION/TERMINATION CRITERIA

In one of the following situations, the trial should be suspended or terminated:

- When the DSMB requires a suspension/complete termination of the trial and the sponsor agrees;
- When the sponsor requires a suspension/complete termination of the trial and gives reasons for it;
- When the Ethics Committee requires a suspension/complete termination of the trial and gives reasons for it;
- When the regulatory agency requires a suspension/complete termination of the trial and gives reasons for it.

6 DESCRIPTION OF STUDY PROCEDURES AND VISITS

All required study procedures and evaluations are to be conducted as outlined in this protocol. In the event of a deviation from the protocol due to an emergency, accident, or mistake, the investigator or designee must notify the sponsor as soon as possible.

Additional assessments (vital signs, ECG, laboratory test, etc.) can be done at the discretion of the investigators as clinically indicated.

This study includes a screening period, a randomization and vaccination period, and follow-up period (the follow-up period includes safety follow-up and immunogenicity follow-up), using a combination of on-site visits and electronic forms such as phone call visits.

6.1 V1 (SCREENING, D-14~D0)

After the participants sign the informed consent form, they will be screened during the screening period (14 days before vaccination to the day of vaccination), and their baseline data will be collected. Investigators will determine whether the participants can be included in the clinical trial according to the "inclusion/exclusion criteria". Investigators fill the participant information collected during the screening period (including screening number, age, disease history or vaccination history, test results in screening period, screening date, enrollment status and location [if applicable], and reasons for non-compliance with study enrollment [if applicable], etc.) into the original file and the corresponding part of the eCRF.

The following check must be completed before enrollment:

- Confirming and collecting the ICF signed by the participant;
- Reviewing the inclusion/exclusion criteria;
- Demographic data: including age, sex, race, ethnicity, occupation (work, living

environment), height, weight, BMI (calculated by height and weight). The participants should also provide contact information like current phone number and/or E-mail. In subsequent follow-up visits, if the contact information is changed, it should be updated accordingly (if applicable).

- Records of medical history: including the history of SARS-COV-2 vaccination, history of COVID-19, other vaccinations within 90 days, medication use within 28 days, and major surgery, allergic history, and other known significant diseases.
- Assigning screening number;
- Physical examination: including general conditions, head and neck, lymph nodes, skin, chest, abdomen, musculoskeletal system and other examinations necessary for the study;
- Vital signs: including blood pressure, respiration rate, pulse rate, and body temperature;
- Nasal/pharyngeal/throat swab nucleic acid test;
- HIV test.
- Urine pregnancy test (for women of childbearing potential only).
- Recording the concomitant medication

6.2 V2 (D0, VACCINATION)

The specific visit content is as follows (if the screening and the vaccination are on the same day, there is no need to repeat the vital signs and urine pregnancy test before vaccination):

- Vital signs: including blood pressure, respiration rate, pulse rate, and body temperature;
- Urine pregnancy test (for women of childbearing potential only);
- Reviewing the inclusion/exclusion criteria;
- Randomization: participants are randomized after all the tests have been done and eligibility has been confirmed;
- Vaccination;

Immunogenicity follow-up visit

- Cellular immune response test (before vaccination and only applied to the cellular immune response subgroup): IL-4 and IFN-γ;
- Neutralizing antibodies test against Delta, Omicron variants and other variants (before vaccination and only applied to participants of Subgroup 1 in Cohort 1 and all participants in Cohort 2);

Safety follow-up visit

- Observing for at least 30 minutes after the vaccination;
- Distribution of vaccination record cards (VRCs): distribute the VRCs after the study vaccination;
- Distributing clinical thermometers and instructing participants to measure and record body temperature;
- Solicited and unsolicited AEs: collect solicited AEs and unsolicited AEs after the study vaccination;
- AEs, SAEs and AESIs;
- Recording the concomitant medication.

Providing the participants with an emergency contact number and instructing them to contact the designated medical center immediately when an event that requires emergency medical treatment occurs after the study vaccination. Providing the participants with thermometers and tape measures, and instructing them to record symptoms, signs, and the severity of adverse events within 28 days after the study vaccination. Participants should record solicited and unsolicited AEs within 7 days after the study vaccination, unsolicited AEs within 28 days and concomitant medications after the study vaccination, and other information on the VRC.

6.3 V3 (D7+2D)

Safety follow-up visit

- Solicited and unsolicited AEs: collect solicited AEs and unsolicited AEs after the study vaccination;
- AEs, SAEs and AESIs;
- Reviewing and recovering the VRCs;
- Recording the concomitant medication.

6.4 V4 (D28+3D)

Immunogenicity follow-up visit

- Cellular immune response test (only applied to the cellular immune response subgroup): IL-4 and IFN-γ;
- Neutralizing antibodies test against Delta, Omicron variants and other variants (only applied to participants of Subgroup 1 in Cohort 1 and all participants in Cohort 2);

Safety follow-up visit

- Reviewing and recovering the VRCs;
- Unsolicited AEs: collect unsolicited AEs after the study vaccination;
- AEs, SAEs and AESIs;

- Recording the concomitant medication.

6.5 V5/EOS (D180±7D)

Immunogenicity follow-up visit

- Neutralizing antibodies test against Delta, Omicron variants and other variants (only applied to participants of Subgroup 1 in Cohort 1 and all participants in Cohort 2);

Safety follow-up visit

- SAEs and AESIs;
- Recording the concomitant medication: After 28 days after the study vaccination, only the concomitant medication used to treat SAEs and AESIs should be recorded.

6.6 UNPLANNED CONTACT AND FOLLOW-UP

At the request of the participant, or during the study period, the investigator may conduct unplanned contact and follow-up with the participant (a visit other than the follow-up specified in the regular schedule) according to the situation. All unplanned contacts and follow-ups will be recorded in the participant's original file and eCRF.

7 STUDY VACCINES

7.1 BASIC INFORMATION OF VACCINES

Study vaccine 1

Name:	Bivalent SARS-CoV-2 trimeric spike protein vaccine (SCTV01C)
Components:	Main active ingredients: SCTV01C-TM22 protein, SCTV01C-TM23 protein;<br>SCT-VA02B adjuvant: The adjuvant is comprised of 0.09 mg of citric acid, 0.59 mg of sodium citrate, 1.25 mg of polysorbate 80, 1.25 mg of span 85 and 10.75 mg of squalene;<br>Excipients: citrate, sodium citrate, sodium chloride, polysorbate 80, sodium hydroxide, WFI;
Dosage form:	Solution for injection
Appearance:	Emulsified, white suspension (due to the presence of adjuvant)
Strength:	20μg (10/10μg for TM22/TM23) /0.5mL/vial;
Storage conditions:	Stored and transported at 2~8°C away from light;
Validity period:	24 months
Manufacturer:	Sinocelltech Ltd.

Study vaccine 2

Name:	COVID-19 Alpha/Beta/Delta/Omicron Variants S-Trimer Vaccine (SCTV01E)

Components:	Main active ingredients: SCTV01E-TM22 protein, SCTV01E-TM23 protein, SCTV01E-TM28 protein, SCTV01E-TM41 protein;<br>SCT-VA02B adjuvant: The adjuvant 1X is comprised of 0.09 mg of citric acid, 0.59 mg of sodium citrate, 1.25 mg of polysorbate 80, 1.25 mg of span 85 and 10.75 mg of squalene;<br>Excipients: citrate, sodium citrate, sodium chloride, polysorbate 80, sodium hydroxide, WFI;
Dosage form:	Solution for injection
Appearance:	Emulsified, white suspension (due to the presence of adjuvant)
Strength:	30μg (5/5/5/15μg for TM22/TM23/TM28/TM41)/0.5mL/vial;
Storage conditions:	Stored and transported at 2~8°C away from light;
Validity period:	24 months
Manufacturer:	Sinocelltech Ltd.

Sinopharm inactivated COVID-19 vaccine: It will be used according to the medicine specification.

mRNA COVID-19 vaccine: Based on the available mRNA vaccine. Detailed information refers to medicine specification.

7.2 STUDY VACCINE MANAGEMENT

SCTV01C, SCTV01E and Sinopharm inactivated COVID-19 vaccine will be stored and transported at 2~8°C away from light. mRNA COVID-19 vaccine will be stored based on medicine specification.

Study vaccines are provided by the sponsor free of charge and distributed to the study sites as planned. Trained personnel at the study site are responsible for recording the receipt and preservation of vaccines, medication for each participant, recovery and maintenance. Only eligible participants will receive the study vaccine. All study vaccines must be stored in a safe, environmentally controlled and (manual or automatic) monitoring area according to the prescribed storage conditions, and only investigators and authorized site staff can obtain them. The disposed, expired, and remaining study vaccines should be destroyed in accordance with the requirements of the sponsor, the vaccine management guidelines or equivalent documents.

7.3 VACCINATION ROUTE AND DOSE

Vaccination site: the deltoid muscle on the outer side of the upper arm;

Vaccination route: intramuscular injection;

Vaccination dose: SCTV01C 20μg; SCTV01E 30μg; Sinopharm inactivated COVID-19 vaccine will be used according to the medicine specification. The dosage of mRNA COVID-

19 vaccine and detailed information refers to medicine specification.

Before injection, the injection site should be disinfected with 75% alcohol, and the study vaccine is injected intramuscularly after the skin is slightly dry. Before each vaccination, the study vaccine should be shaken slightly before extraction. If the vaccine is found to be abnormal, such as abnormal color, broken vial, insufficient medicine, unclear label, etc., it must not be used, and a spare vaccine can be used for vaccination. The participants will be observed at site for at least 30 minutes after each study vaccination. The site should be equipped with appropriate emergency medical treatment measures to treat possible allergic reactions after the study vaccination.

Note: The deltoid muscle of the non-dominant hand is preferred for injection; it is forbidden to inoculate in the buttocks and other parts. Do not vaccinate within 2 cm of tattoos, scars or skin defects. Strictly follow the standard vaccination method and do not inject the vaccine into the blood vessel. For other precautions for vaccination, please refer to the SCTV01C Investigator's Brochure and SCTV01E Investigator's Brochure.

7.4 VACCINATION PROCEDURE

Before each vaccination, the information of the participant and the study vaccine must be checked. All participants receive 1 doses of study vaccine (SCTV01C or SCTV01E), active comparator (Sinopharm inactivated COVID-19 vaccine or mRNA COVID-19 vaccine) on D0.

7.5 CONCOMITANT MEDICATION

Concomitant medication refers to all drugs used by participants during the study period except the study vaccine, including treatments related to AEs, SAEs and AESIs that occurred during the study period.

The information of the concomitant medication, including the name of the drug, the purpose of administration, the usage and dosage, and the time of use, must be recorded in the eCRF in detail.

7.5.1 ALLOWED CONCOMITANT MEDICATION

During the study period, the following drugs are allowed:

- Drugs used to control concomitant diseases are allowed to be used continuously during the study period if the investigator judges that they are not expected to interfere with the test results;
- Necessary drug treatment for the participant's AEs is allowed;
- Participants diagnosed with COVID-19 after the study vaccination are allowed to be treated according to local standards;
- During the study period, if participants are participant to routine immunization with

vaccines other than COVID-19 vaccine, they can be vaccinated according to the product manual, but there must be an interval of 14 days between the routine immunization and the test vaccination. Vaccinations for medical emergencies, such as rabies or tetanus, can be vaccinated in time according to the product instructions.

7.5.2 PROHIBITED CONCOMITANT MEDICATION

During the study period, the following drugs are prohibited:

- Prohibiting any other COVID-19 preventive medication;
- Prohibiting unapproved drugs/vaccines other than the study vaccine;
- Prohibiting long-term use of (continuous use> 14 days) glucocorticoids (dose ≥0.5 mg/kg/d prednisone or equivalent) or other immunosuppressive agents (except for inhaled and topical corticosteroid, or short-term ≤14 days oral steroids);
- Prohibiting immunoglobulin or other blood products;
- The participants should avoid taking over-the-counter drugs, such as antipyretics (such as acetaminophen) and anti-inflammatory drugs (such as ibuprofen, naproxen, etc.) within 12 hours before each vaccination.

7.6 PARTICIPANT COMPLIANCE

Participants will receive the study vaccine directly from the staff of the study site. The staff of the study site record the detail of the date and specific time of the participant's vaccination in the original documents and eCRF.

8 COLLECTION, PROCESSING AND TESTING OF BIOLOGICAL SAMPLES

8.1 COLLECTION, PROCESSING AND TESTING OF IMMUNOGENICITY SAMPLES

8.1.1 COLLECTION OF BLOOD SAMPLES

The participants in Subgroup 1 in Cohort 1 and all participants in Cohort 2 will be sampled for neutralizing antibody test against Delta, Omicron variants and other variants on D0 (before vaccination), D28 and D180. The amount of collected blood for nAb testing each time is not more than 10mL, which will depend on actual laboratory requirement.

The participants in the cellular immune response subgroup will be sampled for cellular immune response test on D0 (before vaccination) and D28. The amount of collected blood for cellular immune response testing each time is not more than 10mL, which will depend on actual laboratory requirement.

8.1.2 PROCESSING AND STORAGE OF BLOOD SAMPLES

The biological samples collected in this study will be properly stored as required. The participant can revoke the permission for other ways of using the sample in the future at any time.

For processing, storage and transportation of blood samples, please refer to the relevant documents of the laboratory management manual.

8.2 COLLECTION, PROCESSING AND TESTING OF VIRUS TEST SAMPLES

8.2.1 COLLECTION OF VIRUS TEST SAMPLES

For all participants who have first suspicious symptoms, a sample of nasal/nasopharyngeal/throat swab should be collected within 72 hours and be divided into two parts, one of which is for the RT-PCR test, the other one is for sequencing in case that the diagnosis of COVID-19 is confirmed. If the onset is more than 72 hours, it should be collected as soon as possible. The sampling tube should be labeled with the random number and other information refer to the lab manual. The specific content of the label can be formulated with reference to the corresponding regulations of the regulatory authority of the country where the trial is located. The collected samples should be sent for tests in time.

8.2.2 STORAGE AND TRANSPORTATION OF VIRUS TEST SAMPLES

For details, please refer to the biological sample management guidelines or equivalent documents.

8.2.3 VIRUS-SPECIFIC NUCLEIC ACID TEST

RT-PCR for SARS-CoV-2 nucleic acid test is adopted, using polymerase chain reaction (PCR) amplifiers and kits approved by the drug regulatory authority approved by the sponsor.

RT-PCR testing of samples from participants is carried out by testing laboratories that meet local regulatory standards. If the test result of a suspicious case is in doubt, the test should be repeated.

9 STUDY EVALUATIONS AND REPORTS

9.1 SAFETY ASSESSMENT

All participants will have safety follow-ups until 180±7 days after the study vaccination.

The participants will be observed at the study site for at least 30 minutes after each study vaccination, and the solicited and unsolicited AEs at the vaccination site (local) and non-vaccination site (systemic) will be reported during this period.

Participants need to record the occurrence of solicited AEs within 7 days and unsolicited AEs within 28 days after the study vaccination on the VRCs and return to the study site with the

VRCs on specified time points. And the safety information will be recorded into eCRF. If participants cannot come to site on visit day, phone call, short message, email or other contacting method will be used for safety follow-up. The sponsor will provide a telephone number and instruct the patients to call in case of any adverse events to receive medical assistance.

Note: The investigator should report all serious adverse events to the sponsor and the contract research organization (CRO) designated by the sponsor within 24 hours after being informed of the SAEs and should also report the SAEs to the ethics committees (ERC/IRB) and local regulatory agencies in accordance with local regulations.

9.1.1 DEFINITION

9.1.1.1 AEs

AE refers to all the adverse medical events that occur after the participant receives the study vaccine. It can be manifested as symptoms and signs, diseases, or abnormal laboratory tests, which does not necessarily have to have a causal relationship with the study vaccine. Previous stable conditions that have been abnormal in the past and whose severity has not changed during the trial period are not regarded as AEs but should be recorded in medical history.

9.1.1.2 Adverse reaction

All noxious and unintended responses to a medicinal product related to any dose should be considered adverse drug reactions. The phrase “responses to a medicinal product” means that a causal relationship between a medicinal product and an adverse event is at least a reasonable possibility, i.e., the relationship cannot be ruled out.

9.1.1.3 Solicited AEs

Solicited AEs are pre-specified and actively monitored during the study, and participants are required to record solicited AEs. Investigators will conduct assessment for solicited AE collected within the first 30 minutes after the study vaccination, and 7 days after the study vaccination (data collected from day 0 to day 7). Participants will be provided with VRCs to record whether solicited AEs occur and record the severity and concomitant medications.

Solicited AEs can be divided into injection-site (local) adverse events and non-injection-site (systemic) adverse events according to the site of occurrence. See Table **2** for detailed information.

Table 2 List of solicited AE

Solicited local AEs	Solicited systemic AEs
• Pain at the injection site<br>• Tenderness at the injection site<br>• Erythema at the injection site	• Fever<br>• Nausea<br>• Vomiting

• Redness at the injection site<br>• Swelling at the injection site<br>• Induration at the injection site	• Headache<br>• Fatigue<br>• Myalgia<br>• Arthralgia, joint pain<br>• Chill

9.1.1.4 Unsolicited AEs

Unsolicited AEs are not specified for active monitoring. An unsolicited AE is any AE reported by the participant that is not specified as a solicited AE in the protocol; or is specified as a solicited AE in the protocol, but starts outside the protocol-defined period for reporting solicited AEs (ie, for the 7 days after the IP).

The investigators assess the relevance and severity of unsolicited AEs based on the FDA guidelines in the appendix.

9.1.1.5 SAEs

A serious adverse event or reaction is any untoward medical occurrence that at any dose:

* results in death,
* is life-threatening,

NOTE: The term "life-threatening" in the definition of "serious" refers to an event in which the patient was at risk of death at the time of the event; it does not refer to an event which hypothetically might have caused death if it were more severe.

* requires inpatient hospitalization or prolongation of existing hospitalization,
* results in persistent or significant disability/incapacity, or
* is a congenital anomaly/birth defect.

Medical and scientific judgement should be exercised in deciding whether expedited reporting is appropriate in other situations, such as important medical events that may not be immediately life-threatening or result in death or hospitalization but may jeopardize the patient or may require intervention to prevent one of the other outcomes listed in the definition above. These should also usually be considered serious.

Hospitalizations due to elective surgery, routine clinical procedures, annual check-ups, and hospitalization observation or protocol, rather than adverse events are not considered as serious adverse events. If an unexpected event occurs during this process, it should be reported as a "serious" or "non-serious" adverse event according to conventional standards.

Note: Hospitalization or prolongation of the hospitalization period due to non-medical reasons/convenience, etc. or only for clinical trial purposes does not meet the criteria for medical events and therefore cannot be regarded as a SAE.

9.1.1.6 AESIs

AESIs refers to adverse events that are of special concern to study vaccines from a scientific

or medical point of view.

Throughout the study, AESIs will be collected according to Safety Platform for Emergency Vaccines (SPEAC) and reported to the sponsor within 24 hours after awareness of investigator.

Table 3 List of Potential Immune-mediated Diseases to be Collected in the Context of Vaccines Containing Adjuvant System

Neuroinflammatory disorders	Musculoskeletal disorders	Skin disorders
● Cranial nerve neuropathy, including paralysis and paresis (eg, Bell's palsy).<br>● Optic neuritis.<br>● Multiple sclerosis.<br>● Transverse myelitis.<br>● Guillain-Barre syndrome, including Miller Fisher syndrome and other variants.<br>● Acute disseminated encephalomyelitis, Including site specific variants, eg, noninfectious encephalitis, encephalomyelitis, myelitis, myeloradiculoneuritis.<br>● Myasthenia gravis, including Lambert-Eaton myasthenic syndrome.<br>● Demyelinating peripheral neuropathies including:<br>- Chronic inflammatory demyelinating polyneuropathy.<br>- Multifocal motor neuropathy.<br>- Polyneuropathies associated with monoclonal gammopathy.<br>● Narcolepsy.	● Systemic lupus erythematosus and associated conditions.<br>● Systemic scleroderma (systemic sclerosis), Including:<br>- Diffuse scleroderma.<br>- CREST Syndrome.<br>● Idiopathic inflammatory myopathies, Including:<br>- Dermatomyositis.<br>- Polymyositis<br>● Antisynthetase syndrome.<br>● Rheumatoid arthritis and associated conditions Including:<br>- Juvenile idiopathic arthritis.<br>- Still' s disease.<br>● Polymyalgia rheumatica.<br>● Spondyloarthropathies, Including:<br>- Ankylosing spondylitis.<br>- Reactive arthritis (Reiter's syndrome).<br>- Undifferentiated Spondyloarthritis.<br>- Psoriatic arthritis.<br>- Enteropathic arthritis.<br>● Relapsing polychondritis.<br>● Mixed connective tissue disorder.<br>● Gout.	● Psoriasis.<br>● Vitiligo.<br>● Erythema nodosum.<br>● Autoimmune bullous skin diseases (including pemphigus, pemphigoid, and dermatitis herpetiformis)<br>● Lichen planus.<br>● Sweet's syndrome.<br>● Localized scleroderma (morphea).
**Vasculitis**	**Blood disorders**	**Others**
● Large vessels vasculitis Including:<br>- Giant cell arteritis (temporal arteritis).<br>- Takayasu's arteritis<br>● Medium sized and/or small vessels vasculitis including:<br>- Polyarteritis nodosa.<br>- Kawasaki's disease.<br>- Microscopic polyangiitis.<br>- Wegener's granulomatosis (granulomatosis with polyangiitis)<br>- Churg-Strauss syndrome (allergic granulomatous angiitis or eosinophilic granulomatosis with polyangiitis)<br>- Buergers disease (thromboangiitis Obliterans).<br>- Necrotizing vasculitis (cutaneous or systemic)<br>- Antineutrophil cytoplasmic antibody positive vasculitis (type unspecified)<br>- Henoch-Schonlein purpura (IgA vasculitis).<br>- Behcet' s syndrome.<br>- Leukocytoclastic vasculitis.	● Autoimmune hemolytic anemia.<br>● Autoimmune thrombocytopenia.<br>● Antiphospholipid syndrome.<br>● Pernicious anemia.<br>● Autoimmune aplastic anemia<br>● Autoimmune neutropenia.<br>● Autoimmune pancytopenia.	● Autoimmune glomerulonephritis Including:<br>- IgA nephropathy.<br>- Glomerulonephritis rapidly progressive.<br>- Membranous glomerulonephritis.<br>- Membranoproliferative glomerulonephritis.<br>- Mesangioproliferative glomerulonephritis.<br>- Tubulointerstitial-nephritis and uveitis syndrome.<br>● Ocular autoimmune diseases Including:<br>- Autoimmune uveitis.<br>- Autoimmune retinitis.<br>● Autoimmune myocarditis.<br>● Sarcoidosis.<br>● Stevens-Johnson syndrome.<br>● Sjögren's syndrome.<br>● Alopecia areata.<br>● Idiopathic pulmonary fibrosis.<br>● Goodpasture syndrome.<br>● Raynaud's phenomenon.
**Liver disorders**	**Gastrointestinal disorders**	**Endocrine disorders**
● Autoimmune hepatitis.	● Inflammatory bowel disease,	● Autoimmune thyroiditis

● Primary biliary cirrhosis.<br>● Primary sclerosing cholangitis.<br>● Autoimmune cholangitis.	including:<br>- Crohn's disease.<br>- Ulcerative colitis.<br>- Microscopic colitis.<br>- Ulcerative proctitis.<br>● Celiac disease.<br>● Autoimmune pancreatitis.	● (Hashimoto thyroiditis).<br>● Grave' s or Basedow' s disease.<br>● Diabetes mellitus type I.<br>● Addison' s disease.<br>● Polyglandular autoimmune<br>● syndrome.<br>● Autoimmune hypophysitis.

IgA = immunoglobulin A

Table 4 List of Adverse Events of Special Interest Applicable to COVID-19 vaccines (Guidance Document from SPEAC)

Body System	AESI Type	Rationale for Inclusion as an AESI (see Footnotes)
Neurologic	Generalized convulsion	1，2，4
	Guillain-Barré Syndrome	2
	Acute disseminated encephalomyelitis	3
Hematologic	Thrombocytopenia	1，2
Immunologic	Anaphylaxis	1，2
	Vasculitides	3，4
Other	Serious local/systemic AEs following immunization	1，2

AE = adverse event, AESI = adverse events of special interest, COVID-19 = Coronavirus disease-2019.
1.Proven association with immunization encompassing several different vaccines.
2.Proven association with vaccine that could theoretically be true for CEPI vaccines under development.
3.Theoretical concern based on immunopathogenesis.
4.Theoretical concern related to viral replication during wild type disease.
5.Theoretical concern because it has been demonstrated in an animal model with 1 or more candidate vaccine platforms.

Table 5 List of Adverse Events of Special Interest Relevant to COVID-19 (Guidance Document from SPEAC)

Body System	AESI Type	Rationale for Inclusion as an AESI (see Footnotes)
Respiratory	Acute respiratory distress syndrome	3, 4
	Pneumonitis	3, 4
Immunologic	Enhanced disease following immunization	1, 2, 5
Other	Acute cardiac injury	3, 4
	Arrhythmia	3, 4
	Septic shock-like syndrome	3, 4
	Acute kidney injury	3, 4
	Multi-system inflammatory syndrome similar to Kawasaki's disease	
	Angioedema	

AESI = adverse event of special interest, CEPI = Coalition for Epidemic Preparedness Innovations, CoV = Coronavirus, COVID-19 = Coronavirus disease-2019, HIV = human immunodeficiency syndrome, MERS = middle-eastern respiratory syndrome, SARS = severe acute respiratory syndrome.
1.Proven association with immunization encompassing several different vaccines (formalin-inactivated measles/RSV vaccines; HIV vaccine)
2.Proven association with vaccine that could theoretically be true for CEPI vaccines under development (Chimeric Yellow Fever Dengue vaccine)
3.Theoretical concern based on immunopathogenesis.
4.Theoretical concern related to viral replication during wild type disease.
5.Theoretical concern because it has been demonstrated in an animal model with 1 or more candidate vaccine platforms (mouse models SARS/MERS-CoVs).

The AESIs of this study will be updated or revised with the collection of cumulative safety data.

9.1.1.7 Suspected and unexpected serious adverse reaction

Suspected Unexpected Serious Adverse Reaction (SUSAR) means the nature, severity, outcome, or frequency of these adverse reactions are not consistent with the risk information in the current relevant applicable product information (such as the investigator's brochure). The investigator's brochure serves as the main document to provide safety reference information for judging whether an adverse reaction is expected or unexpected.

9.1.1.8 Severity of adverse events

The grading scales used to assess adverse events are derived from the "Toxicity Rating Scale for Healthy Adult and Adolescent Volunteers in Preventive Vaccine Clinical Trial-FDA Standard" (Appendix I);

For adverse events not listed in the grading table, the intensity will be assessed according to the following standards. For details, see Table 6 .

Table 6 General principles for the grading of adverse events

Grade 1	Grade 2	Grade 3	Grade 4	Grade 5
**Mild**: No interference with activity	**Moderate**: Some interference with activity not requiring medical intervention	**Severe**: Prevents daily activity and requires medical intervention	**Potentially life threatening**: ER visit or hospitalization	**Death**

9.1.1.9 Causality between adverse events and vaccines

In this study, solicited local adverse events are considered to be related to vaccination. For solicited systemic adverse events, unsolicited adverse events, serious adverse events and ASEIs, the investigator is obligated to assess the relationship between study vaccine and each occurrence of each AEs/SAEs. Investigators should assess the relationship between adverse events and study vaccine in a timely manner, make clinical judgments based on the information available at the time of the report, and change the opinion of causality in light of the follow-up information.

Investigators are asked to use a simple binary decision for drug causality (related or not related) for adverse events. One possible approach that has been suggested is to ask simply was there a reasonable possibility? Yes or No.

Yes: There is a plausible temporal relationship between adverse events and the study vaccine, and the adverse events cannot be explained by the participant's clinical status, concurrent disease or concomitant treatment; and/or adverse events follow the known response pattern of the study treatment; and/or once the study vaccine is discontinued or the dose is reduced, the adverse event is improved or recovered, and the adverse event occur again after the vaccine is re-administered under appropriate circumstances.

No: Evidence shows that the adverse event has other triggers other than the study vaccine (for example, original medical condition, underlying disease, concurrent disease or concomitant medication); and/or there is no reasonable temporal relationship between the adverse event and the study vaccination.

9.1.2 OUTCOME OF ADVERSE EVENTS

The outcome of AEs can be described as the following:

- Recovered/Resolved: "Termination date of adverse events" should be indicated. Recovered to baseline level is Recovery.
- Recovered/Resolved with sequelae: only if the participant has long-term or lifelong sequelae, such as blindness caused by diabetes and hemiplegia after stroke. "Termination date of (serious) adverse event" should be indicated.
- Recovering/Resolving: The event has not yet been completely resolved, but the participant is already in the recovery phase.
- Not Recovered/Not Resolved: The event is in progress.
- Fatal: Death of participants directly or mainly caused by AEs.
- Unknown: The investigator cannot obtain the information of the AEs, for example, the participant is lost to follow-up.

The end date of the adverse event is the date at which the participant recover or recover with sequelae or the participant died.

If the outcome of the adverse event is assessed as "recovering", or "unrecovered", or "unknown", it is temporarily not necessary to record the end date of the adverse event.

If the outcome of an adverse event is assessed as "recovered" or "recovered with sequelae", the end date of the adverse event must be recorded.

9.1.3 RECORDING OF ADVERSE EVENTS

9.1.3.1 Time Period for Collecting Adverse Events

Solicited AEs are collected within 7 days after each study vaccination; unsolicited AEs are collected within 28 days after each study vaccination; SAEs and AESIs are collected within 180±7 days after the study vaccination.

The adverse medical occurrences that begin after signing the informed consent and before the study vaccination will be recorded in the "Medical History/Current Medical Condition" section of the CRF instead of the "AE" section.

SAEs (including death) occurring in a participant after withdrawal from the study must be reported to the sponsor or designee if the investigator becomes aware of them and believes to have a reasonable possibility of being related to study vaccine.

For the solicited symptoms and unsolicited symptoms, the investigator should confirm with the participant whether he/she received hospitalization, outpatient treatment, or self-administered medication for any reason, and record this information.

The training for participants emphasizes on the timely reporting of AE. Investigators should be highly vigilant about such events, investigate and deal with them in a timely manner.

When a SAE occurs, the investigator is responsible for reviewing all documents related to the event (such as hospital history records, laboratory reports, and diagnostic reports), or in order to clarify the nature and causality of the SAEs. If the participant is confirmed dead during the study period, the hospital's final conclusions about the deceased should be collected. If an autopsy is performed, a copy of the results, including histopathological results, should be obtained.

9.1.3.2 Methods of discovering adverse events

At each visit, AEs can be found by the following methods:

- Information proactively provided by the participant or caregiver; when the participant has an acute or gradually worsening adverse reaction, the investigator or the corresponding contact person should be contacted for further treatment opinions and/or measures.
- At each follow-up, ask the participants open and non-leading questions: such as "How do you feel? Have you had any (other) medical problems since the last follow-up visit? "
- Abnormalities observed by investigators, other medical staff, and family members.

Investigators will also provide participants with VRCs (electronic and/or paper) to record solicited AEs from 0 to 7 days after each study vaccination and unsolicited AEs from 0 to 28 days after each study vaccination.

9.1.3.3 Recording and follow-up of adverse events

The investigator is responsible for recording all AEs and SAEs, and reports to the sponsor and the sponsor designated CRO within 24 hours after learning of the SAEs. It is required to collect AEs from day 0 to day 7 after each study vaccination, and unsolicited AEs from day 0 to day 28 after each study vaccination, SAEs from day 0/vaccination throughout the study period. During each study site visit or remote follow-up, participants will be questioned including COVID-19 symptom monitoring to ensure their safety. At the same time, the participants will be asked whether they have been hospitalized, whether an accident occurred, whether they are using new drugs, whether they have changed the concomitant medication regimen (including prescription drugs and over-the-counter drugs), or whether they are vaccinated with non-experimental vaccines. Physical examination results or other adverse event information related to the safety of the participant should be recorded. After investigators complete the AE and SAEs reports, they should continue to follow up the AEs and SAEs during follow-up visits. All AEs and SAEs that occurred during the study should be treated correspondingly, and followed

up until recovery, improvement, stability or other outcomes [investigators believe that no further follow-up is necessary for reasonable reasons (such as it cannot be recovered or has improved); when no more information can be obtained (for example, the participant refuses to provide more information, or evidence shows that the participant is still lost to follow-up after best efforts have been made)], or the participant is lost to follow-up.

In order to improve the quality and accuracy of information collection on adverse events, investigators should follow the following guidelines:

- When AE is recorded in eCRF, use recognized medical terms as much as possible;
- Record diagnostic results (i.e., diseases or syndromes), rather than related signs, symptoms, and laboratory test results (for example, record congestive heart failure instead of dyspnea, rales and cyanosis);
- Record and report the SAEs that caused the death;
- For patients who are hospitalized due to surgical procedures or diagnostic procedures, the disease that leads to the surgical procedures or diagnostic procedures, not the procedure itself, should be recorded as SAEs. This process should be recorded in the disease treatment measures in the case narrative;
- Pregnancies of participants during the study are not considered as adverse events, but should be recorded in a separate pregnancy record form, and sent to the sponsor and the sponsor designated CRO. If the pregnancy results meet the SAEs criteria (including spontaneous abortion, stillbirth, or any congenital malformations, etc.), the investigator should report it according to the SAEs reporting process.

9.1.4 SAFETY MONITORING

The investigator and/or designated on-site personnel are responsible for monitoring the safety of all participants and notifying the sponsor when unexpected issues occur. DSMB will conduct independent and continuous monitoring of the safety data of the study vaccine and judge the results.

9.1.5 SAE/SUSAR/PREGNANCY EVENT REPORT

9.1.5.1 Requirement of immediate report by investigators to the sponsor

The following is a list of events that investigators must report to the sponsor within 24 hours of being notified. These events do not necessarily need to be related to the study vaccine:

- SAEs;
- AESI;
- Pregnancy.

For these events, investigators must report new significant follow-up information to the sponsor immediately (that is, within 24 hours after getting the information). New significant information includes the following:

- New signs or symptoms, or changes in diagnosis;
- Important new diagnostic test results;
- New information that may lead to a change in causality assessment;
- Changes in the outcome of the event, including recovered events;
- Other important descriptive information about the clinical course of the event.

All SAEs should also be filled in the eCRF form at the same time, and the information in the SAEs report form must be consistent with the event data recorded in the eCRF.

9.1.5.2 Regulatory Reporting Requirements for SAEs

Prompt notification by the investigator to the sponsor of an SAE is essential so that legal obligations and ethical responsibilities towards the safety of participants and the safety of a study vaccination under clinical investigation are met.

The sponsor has a legal responsibility to notify both the local regulatory authority and other regulatory agencies about the safety of a study vaccination under clinical investigation. The sponsor will comply with country-specific regulatory requirements relating to safety reporting to the regulatory authority, institutional review boards (IRBs)/ethics committees (ECs), and investigators.

Investigator safety reports must be prepared for suspected unexpected serious adverse reactions (SUSARs) according to local regulatory requirements and sponsor policy and forwarded to investigators as necessary.

An investigator who receives an investigator safety report describing an SAE or other specific safety information (e.g., summary or listing of SAEs) from the sponsor will review and then file it along with the investigator's brochure and will notify the IRB/EC, if appropriate according to local requirements.

9.1.5.3 Pregnancy report

When female participants or female partners of male participants become pregnant during the study period (collection period is the same as SAEs), the "pregnancy report form" should be filled out within the same time limit as SAEs report to the sponsor (or CRO appointed by the sponsor).

Pregnancy itself is not considered as an AE. If spontaneous abortion, birth defects or congenital abnormalities of newborns, deformities and abnormalities of stillbirths, severe complications of mothers and newborns, and etc. occur during pregnancy, all of them should be recorded and

reported as SAEs.

During the study period, female participants of childbearing potential or the female partners of male participants should immediately notify the investigator once they become pregnant. The investigator should make recommendations to the participants, discuss the risks of continuing pregnancy and the possible impact on the fetus. Male participants do not need to withdraw from the study, but their female partners need to be monitored. The follow-up time for pregnancy events lasts at least until the pregnancy outcome or 12 months after the birth of the newborn.

Note: Female participants of childbearing potential or female partners of male participants have the right to know the actual grouping information after unblinding because of pregnancy.

9.2 IMMUNOGENICITY ASSESSMENT

9.2.1 POPULATION AND TIME OF IMMUNOGENICITY ASSESSMENT

For specific sampling points, please refer to the description in section 8.1.1.

9.2.2 ASSESSMENT INDICATORS

Primary assessment indicators:

Cohort 1

- GMT of nAb against Delta variant on D28.
- GMT of nAb against Omicron BA.1 (B.1.1.529) variant on D28.

Cohort 2

- GMT of nAb against Omicron BA.1 variant on D28.
- GMT of nAb against Delta variant on D28.

Secondary assessment indicators:

Cohort 1

- GMT of nAb against Delta variant on D180.
- GMT of nAb against Omicron BA.1 variant on D180.
- GMT of nAb against Omicron BA.5 variant on D28.
- Number of IFN-γ positive (characterizing Th1) and IL-4 positive (characterizing Th2) T cell subsets on D28.
- Seroresponse of nAb (defined as a change from below the low limit of quantitation [LLOQ] to equal to or above LLOQ, or a ≥4-fold rise if baseline is equal to or above LLOQ in nAb to Delta variant from D0) rates on D28.
- Seroresponse of nAb (defined as a change from below LLOQ to equal to or above LLOQ, or a ≥4-fold rise if baseline is equal to or above LLOQ in nAb to Omicron

variant from D0) rates on D28.

Cohort 2

- GMT of nAb against Delta variant on D180.
- GMT of nAb against Omicron BA.1 variant on D180.
- GMT of nAb against Omicron BA.5 variant on D28;
- Number of IFN-γ positive (characterizing Th1) and IL-4 positive (characterizing Th2) T cell subsets on D28.
- Seroresponse of nAb (defined as a change from below the low limit of quantitation [LLOQ] to equal to or above LLOQ, or a ≥4-fold rise if baseline is equal to or above LLOQ in nAb to Delta variant from D0) rates on D28.
- Seroresponse of nAb (defined as a change from below LLOQ to equal to or above LLOQ, or a ≥4-fold rise if baseline is equal to or above LLOQ in nAb to Omicron variant from D0) rates on D28.

9.3 DIAGNOSIS AND TREATMENT OF COVID-19 INFECTION

9.3.1 DEFINITION OF COVID-19 CONFIRMED CASES

According to FDA's diagnosis and treatment guidelines for COVID-19[14], COVID-19 is defined according to the following criteria:

Participants with positive result of SARS-CoV-2 using a virologic test (i.e., a nucleic acid amplification test of an antigen test) who have the following clinical symptoms or imaging characteristics of COVID-19:

COVID-19 symptoms consistent with those defined by the US FDA harmonized case definition: fever or chills, cough, shortness of breath or difficulty breathing, fatigue, muscle or body aches, headache, new loss of taste or smell, sore throat, congestion or runny nose, nausea or vomiting, diarrhea.

9.3.2 SEVERITY GRADING CRITERIA OF CONFIRMED COVID-19 CASES

Investigators should closely monitor and treat confirmed patients in accordance with the treatment guidelines formulated by FDA.

Investigators can classify cases according to the FDA COVID-19 disease severity[14]. For details, see Table 7.

Table 7 COVID-19 disease severity

Disease severity	Definition
Mild	Symptoms of mild illness with COVID-19 that could include fever, cough, sore throat, malaise, headache, muscle pain, nausea, vomiting, diarrhea, and loss of taste or smell, without shortness of breath or dyspnea<br>No clinical signs indicative of Moderate, Severe, or Critical Severity

Moderate	Symptoms of moderate illness with COVID-19, which could include any symptom of mild illness or shortness of breath with exertion<br>Clinical signs suggestive of moderate illness with COVID-19, such as respiratory rate ≥20 breaths per minute, heart rate≥90 beats per minute; with saturation of oxygen (SpO2) > 93% on room air at sea level<br>No clinical signs indicative of Severe or Critical Illness Severity
Severe	Symptoms suggestive of severe systemic illness with COVID-19, which could include any symptom of moderate illness or shortness of breath at rest, or respiratory distress<br>Clinical signs indicative of severe systemic illness with COVID-19, such as respiratory rate≥30 per minute, heart rate≥125 per minute, SpO 2 ≤ 93% on room air at sea level or PaO 2 /FiO 2 < 300<br>No criteria for Critical Severity
Critical	Evidence of critical illness, defined by at least one of the following:<br>– Respiratory failure defined based on resource utilization requiring at least one of the following:<br>Endotracheal intubation and mechanical ventilation, oxygen delivered by high-flow nasal cannula (heated, humidified, oxygen delivered via reinforced nasal cannula at flow rates > 20 L/min with fraction of delivered oxygen≥0.5), noninvasive positive pressure ventilation, ECMO, or clinical diagnosis of respiratory failure (i.e., clinical need for one of the preceding therapies, but preceding therapies not able to be administered in setting of resource limitation)<br>– Shock (defined by systolic blood pressure<90 mm Hg, or diastolic blood pressure<60 mm Hg or requiring vasopressors)<br>– Multi-organ dysfunction/failure

9.3.3 DISCOVERY OF SUSPICIOUS CASES OF COVID-19

After the study vaccination, all participants will be monitored for symptoms of COVID-19 at each visit. Participants should actively reported to the investigator once they have any signs/symptoms related to COVID-19.

9.3.4 CONFIRMATION PROCEDURES FOR COVID-19 CASES

Participants with any suspicious symptoms of COVID-19 (see 9.3.1 chapter Definition of CVOID-19 confirmed cases) should receive nasal/nasopharyngeal/throat swab collection at the study site or home for RT-PCR test or receive rapid antigen test as soon as possible (preferably within 72 hours). If a sample of nasal/throat/pharyngeal swab is collected, the sample will be divided into two parts, one of which is for the RT-PCR test, the other one is for sequencing in case that the diagnosis of COVID-19 is confirmed. If a sample of nasal/throat/pharyngeal swab is not collected, the sequencing will not be done.

If the RT-PCR test or rapid antigen test result is positive, the diagnosis of COVID-19 is confirmed. If the test result is negative and the symptom persists, a second sample could be taken at least 24 hours (but not more than 3 days) apart for RT-PCR test or rapid antigen test at the discretion of investigators). If the second test result is still negative, the sampling will not be repeated; if the second test result is positive, follow-up will be carried out according to the confirmed case of COVID-19. For specific COVID-19 diagnosis procedures, see Figure 3 .

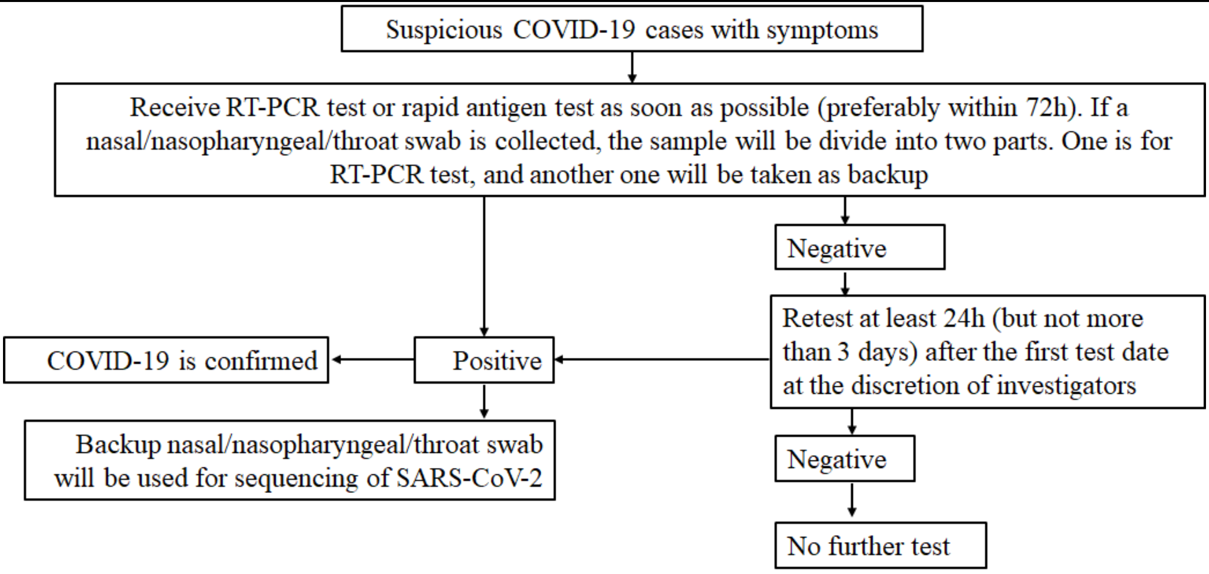

Figure 3 Diagnosis process of COVID-19 cases

9.3.5 FOLLOW-UP OF CONFIRMED COVID-19 CASES

For participants who have been diagnosed with COVID-19, they will be managed and treated in accordance with local policies and regulations. After a participant is diagnosed with COVID-19, the investigator will follow up the participant every 3 to 7 days until the symptoms were sustained resolution, which means all COVID-19 related symptoms remain absent or no worse than mild for selected symptoms that may take a longer time to resolve (e.g., cough, fatigue, loss of smell or taste) for a sustained period of 48 hours. Collect treatment status and medical history of the participant to determine whether the participant meet the severe and critical COVID-19 standards (see 9.3.2 Severity grading criteria of confirmed COVID-19 cases).

If a SARS-CoV-2 infection is confirmed, virus will be isolated from the nasal/nasopharyngeal/throat swab and viral sequencing will be used to identify the major SARS-CoV-2 variants.

Record the participant’s symptom type, time of occurrence, time of report, time of nucleic acid sample collection, time of test, time of report, time of diagnosis, outcome, etc. to form relevant records.

10 DATA MANAGEMENT

10.1 SOURCE DATA AND THE FILLING AND TRANSFER OF ECRF

The electronic data capture system (EDC) is used for data collection in this study. The data management of this study is the responsibility of the sponsor's data department to ensure the authenticity, integrity, privacy and traceability of clinical trial data.

eCRF: data collection form is designed according to the requirements of protocol, to define the study process, the name of the data form and the data items collected, at the same time, the

corresponding eCRF Completion Instructions should be formed, then reviewed by the sponsor to be used by the study Site to fill in the eCRF.

The eCRF data are all derived from the original medical records and filled out by the investigator or investigator designee to ensure the completeness and accuracy of the information. If there are any errors that need to be corrected, the corrections should be made according to the eCRF Completion Instructions, and the EDC system will automatically record the name and date of modification of the data.

After the source data verification (SDV), DM verification, questioning and other processing for the data of the EDC system beyond any doubt, the investigator should conduct electronic signature confirmation before data locking.

10.2 DATABASE PROPOSAL AND DESIGN

The design of eCRF is consistent with the requirements of FDA 21 CFR Part 11 and with the requirements of ICH GCP and GCP (NMPA, 2020) on data collection. The data manager conducts interface tests, including but not limited to: page design, visit period setting, form entry order at visit and order of each data point, etc. As for the new Uniform Resource Locator (URL), the data manager should also test the URL configuration, such as the accuracy of different user browsing permissions, and so on. The database should be established with reference to the Clinical Data Interchange Standards Consortium (CDISC) standards whenever possible.

10.3 ENTRY OF DATA

The investigator should collect participant data in accordance with the requirements of GCP and study protocol, and complete the eCRF accurately, timely, completely, and in accordance with the instructions.

The data is entered into the EDC database by the investigator or a person authorized by the investigator upon completion of the visit. Data entry is carried out in strict accordance with the principle "what you see is what you should record". At the end of data entry, any changes made to the eCRF will be automatically recorded in the system.

10.4 MEDICAL CODING

The coding contents includes, but is not limited to, past medical history, concomitant medications and AEs.

Medical history and AEs will be coded according to the International Medical Dictionary for Regulatory Activities (MedDRA), and concomitant medication will be coded according to the World Health Organization Dictionary of Drugs (WHO DD).

10.5 DATABASE LOCKING AND EXPORTING

The principal investigator, sponsor, statistician, clinical project manager, and DM will decide together to lock the database after all of following items are done: all participants complete the test, the medical records are entered into the system and all data questions are solved, the verifications on the consistency of external data sources show no any errors, the coding report is approved by the sponsor, all problems from database quality control (QC) and data verification meeting (if any) are solved. After all the data is locked, DM will export the data from the system and hand it over to the statistician for statistical analysis. The locked data cannot be edited, and the problems found after the data locking can be corrected in the statistical analysis program after confirmation. If principal investigator, sponsor, statistician, and DM all consider there's solid evidence that it's necessary to unlock after data locking, DM will unlock the data when both the investigator and the sponsor sign the Database Unlock Confirmation Form, then data update is actionable and all updates must be documented. After the update is complete, the locking process should be conducted again.

10.6 ARCHIVE OF STUDY RECORDS

The basic documents for this clinical trial should be maintained for at least 5 years after the approval for marketing of test vaccine by local regulatory authorities. If not used for marketing approval, the documents should be kept for at least 5 years after the end of the clinical trial.

After the period, the study data will be destroyed with the sponsor's written notice.

All documents relating to the test should be stored in strict confidence within the limits of local laws.

11 STATISTICAL ANALYSIS

Detailed methodology for summary and statistical analyses of the data collected in this study is outlined here and will be further detailed in a statistical analysis plan (SAP). The SAP may modify what is outlined in the protocol where appropriate; however, any major modifications of the primary endpoint definitions or their analyses will also be reflected in a protocol amendment.

11.1 HYPOTHESIS

Let

$GMTC1_{Delta}$= GMT of nAb against Delta variant on D28 of SCTV01C in Cohort 1;

$GMTE1_{Delta}$= GMT of nAb against Delta variant on D28 of SCTV01E in Cohort 1;

$GMTS1_{Delta}$= GMT of nAb against Delta variant on D28 of Sinopharm inactivated vaccine in Cohort 1;

$GMTC1_{Omicron1}$= GMT of nAb against Omicron BA.1 variant on D28 of SCTV01C in Cohort 1;

$GMTE1_{Omicron1}$ = GMT of nAb against Omicron BA.1 variant on D28 of SCTV01E in Cohort 1;

$GMTS1_{Omicron1}$ = GMT of nAb against Omicron BA.1 variant on D28 of Sinopharm inactivated vaccine in Cohort 1;

$GMTC1_{Omicron5}$= GMT of nAb against Omicron BA.5 variant on D28 of SCTV01C in Cohort 1;

$GMTE1_{Omicron5}$ = GMT of nAb against Omicron BA.5 variant on D28 of SCTV01E in Cohort 1;

$GMTS1_{Omicron5}$ = GMT of nAb against Omicron BA.5 variant on D28 of Sinopharm inactivated vaccine in Cohort 1;

$GMTC2_{Delta}$= GMT of nAb against Delta variant on D28 of SCTV01C in Cohort 2;

$GMTE2_{Delta}$= GMT of nAb against Delta variant on D28 of SCTV01E in Cohort 2;

$GMTM2_{Delta}$= GMT of nAb against Delta variant on D28 of mRNA COVID-19 vaccine in Cohort 2.

$GMTC2_{Omicron1}$= GMT of nAb against Omicron BA.1 variant on D28 of SCTV01C in Cohort 2;

$GMTE2_{Omicron1}$= GMT of nAb against Omicron BA.1 variant on D28 of SCTV01E in Cohort 2;

$GMTM2_{Omicron1}$= GMT of nAb against Omicron BA.1 variant on D28 of mRNA COVID-19 vaccine in Cohort 2.

$GMTC2_{Omicron5}$= GMT of nAb against Omicron BA.5 variant on D28 of SCTV01C in Cohort 2;

$GMTE2_{Omicron5}$= GMT of nAb against Omicron BA.5 variant on D28 of SCTV01E in Cohort 2;

$GMTM2_{Omicron5}$= GMT of nAb against Omicron BA.5 variant on D28 of mRNA COVID-19 vaccine in Cohort 2.

For the primary efficacy objectives, the null hypotheses are:

For Cohort 1:

- **H11**: $GMR13=GMTE1_{Omicron1}/GMTS1_{Omicron1} \leq 1$;
- **H12**: $GMR14=GMTC1_{Omicron1}/GMTS1_{Omicron1} \leq 1$;
- **H13**: $GMR11=GMTE1_{Delta}/GMTS1_{Delta} \leq 1$;

- **H14**: GMR12= $GMTC1_{Delta}/GMTS1_{Delta} \leq 1$;
- **H15**: GMR15=$GMTE1_{Omicron5}/GMTS1_{Omicron5} \leq 1$;
- **H16**: GMR16=$GMTC1_{Omicron5}/GMTS1_{Omicron5} \leq 1$;

For Cohort 2:

- **H21**: GMR22=$GMTE2_{Omicron1}/GMTM2_{Omicron1} \leq 0.67$;
- **H22**: GMR24=$GMTC2_{Omicron1}/GMTM2_{Omicron1} \leq 0.67$;
- **H23**: GMR21= $GMTE2_{Delta}/GMTM2_{Delta} \leq 0.67$;
- **H24**: GMR23= $GMTC2_{Delta}/GMTM2_{Delta} \leq 0.67$;
- **H25**: GMR26= $GMTE2_{Omicron1}/GMTM2_{Omicron1} \leq 1$;
- **H26**: GMR28=$GMTC2_{Omicron1}/GMTM2_{Omicron1} \leq 1$;
- **H27**: GMR29=$GMTE2_{Omicron5}/GMTM2_{Omicron5} \leq 0.67$;
- **H28**: GMR210=$GMTC2_{Omicron5}/GMTM2_{Omicron5} \leq 0.67$;
- **H29**: GMR211= $GMTE2_{Omicron5}/GMTM2_{Omicron5} \leq 1$;
- **H210**: GMR212=$GMTC2_{Omicron5}/GMTM2_{Omicron5} \leq 1$;
- **H211**: GMR25=$GMTE2_{Delta}/GMTM2_{Delta} \leq 1$;
- **H212**: GMR27= $GMTC2_{Delta}/GMTM2_{Delta} \leq 1$;

11.2 ESTIMAND

The estimand framework of primary efficacy objectives is listed in Tables below.

For Cohort 1:

Table A Estimand framework of primary objectives

Population	Population aged ⩾18 years previously vaccinated with inactivated vaccine
Treatment conditions	Test: SCTV01C, SCTVO1E<br>Control: Sinopharm inactivated COVID-19 vaccine
Variables	Neutralizing antibody titers against Delta or Omicron variant on D28 after first vaccination
Intercurrent event 1	COVID-19 infection up to D28 after first vaccination. A principal stratum strategy will be used, the participants who are diagnosed with COVID-19 up to D28 after first vaccination are excluded from this estimand.
Intercurrent event 2	Receiving of other drugs or vaccines that will modify the immunity against Delta or Omicron variant up to D28 after first vaccination. A principal stratum strategy will be used, the participants who receive other drugs or vaccines that will modify the immunity against Delta or Omicron variant up to D28 after first vaccination are excluded from this estimand.
Population-level summary	Ratio of geometric means of the neutralizing antibody titers

For Cohort 2:

Table B Estimand framework of primary objectives

Population	Population aged ≥18 years previously vaccinated with mRNA vaccine
Treatment conditions	Test: SCTV01C, SCTVO1E<br>Control: mRNA COVID-19 vaccine
Variables	Neutralizing antibody titers against Delta or Omicron variant on D28 after first vaccination
Intercurrent event 1	COVID-19 infection up to D28 after first vaccination. A principal stratum strategy will be used, the participants who are diagnosed with COVID-19 up to D28 after first vaccination are excluded from this estimand.
Intercurrent event 2	Receiving of other drugs or vaccines that will modify the immunity against Delta or Omicron up to D28 after first vaccination. A principal stratum strategy will be used, the participants who receive other drugs or vaccines that will modify the immunity against Delta or Omicron variant up to D28 after first vaccination are excluded from this estimand.
Population-level summary	Ratio of geometric means of the neutralizing antibody titers

11.3 MULTIPLICITY

For Cohort 1:

A fixed sequential hierarchical approach will be used to control the type I error at one-sided 0.025. The hypothesis will be tested according to the Estimand frame as defined in Table A in an order of H11, H12, H13, H14, H15 and H16. The following test will be tested only when the previous one reaches the statistical significance at one-sided significance level of 0.025.

For Cohort 2:

A fixed sequential hierarchical approach will be used to control the type I error at one-sided 0.025. The hypothesis will be tested in an order of H21, H22, H23, H24, H25, H26, H27, H28, H29, H210, H211, and H212 the participants according to the Estimand frame as defined in **Table B**. The following test will be done only when the previous one reaches the statistical significance at one-sided significance level of 0.025.

The multiplicity control procedure may be adjusted according to external information. More details will be defined in the SAP which will be finalized before the study is unblinded.

11.4 SAMPLE SIZE CALCULATION

Totally 1800 participants aged ≥18 years who were previously vaccinated with either inactivated or mRNA COVID-19 vaccine or previously diagnosed with COVID-19 will be enrolled. 300 participants (100 in SCTV01C Group, 100 in SCTV01E Group, 100 in

Sinopharm COVID-19 vaccine Group) in subgroup 1 in Cohort 1 will have nAb tests. 450 participants (150 in SCTV01C Group, 150 in SCTV01E Group, 150 in mRNA COVID-19 vaccine Group) in Cohort 2 will have nAb tests.

For Cohort 1, the sample size is determined based on below assumptions:

- The standard deviation of neutralizing antibody titers under log10 transformation is 0.4;
- The 1-sided type I error is 0.025
- Power is above 80%;
- GMR between SCTV01C/E and Sinopharm vaccine=1.6
- The dropout rate during study is about 10%;

For Cohort 2, the sample size is determined based on below assumptions:

- The standard deviation of neutralizing antibody titers under log10 transformation is 0.4;
- The 1-sided type I error is 0.025
- Power is above 80%;
- GMR between SCTV01C/E and mRNA vaccine =1;
- non-inferiority margin as 0.67;
- The dropout rate during study is about 10%;

11.5 STATISTICAL POPULATIONS

Full Analysis Set (FAS): All randomized participants who received one dose of investigational product (IP).

Per-Protocol Set (PPS): All participants in the FAS set who received planned doses of IP per schedule and have no major protocol deviations, as determined and documented by Sponsor prior to database lock and unblinding, that impact critical or key study data. Those who are COVID-19 infected or take other vaccine/drug after the vaccination and before D28 that could compromise the immunogenicity evaluation will be excluded from PPS as defined in the Estimand frame.

Safety Set (SS): All randomized participants who received one dose of IP.

Immunogenicity full analysis set (I-FAS): All participants in the FAS who had a valid immunogenicity test result prior to receiving the IP and at least 1 valid result after receiving the IP.

Immunogenicity per-protocol set (I-PPS): All participants in the PPS who had a valid

immunogenicity test result prior to receiving the IP and at least 1 valid result after receiving the IP.

11.6 STATISTICAL ANALYSIS METHODS

For each cohort, once the safety data within 28 days and immunogenicity data on D28+3 were acquired, it will be analyzed by unblinded team who are independent to the study operation team and are not directly involved in the study activities. The result will be further used for submission to regulatory authority. The specific analysis time point may be adjusted according to the progress of the trial.

General principles

The statistical analysis is carried out with the descriptive and pre-specified statistical test method. The analytical procedures will be detailed in the statistical analysis plan (SAP).

American SAS 9.4 or above will be used for statistical analysis.

Descriptive statistics of continuous variables will include mean, standard deviation, median, minimum, and maximum values. The classification variable will be described by number and percentage. The calculation method of percentage will be defined in the SAP.

The expected values, standard errors, and 95% confidence interval (Cl) will be calculated based on the assumed distribution and pre-specified models, as defined in the SAP.

The Demographic and Baseline Characteristics

The Demographic and Baseline Characteristics, including protocol deviations will be listed.

Demographic data and baseline indicators will be analyzed among the FAS. All demographic data (age, sex, race, ethnicity, et al) and baseline variables (physical examination, pregnancy test, history of diseases, history of COVID-19, medication history, interval between time of administration of IP and the last time of COVID-19 vaccine administration/diagnosed with COVID-19, the type of previous COVID-19 vaccinations and serum antibody titer before the administration of IP) are summarized.

For continuous variables, descriptive statistics (the number of participants, mean, standard deviation, minimum, median and maximum values) are used; and for classified variables, the number and percentage are calculated.

Study treatment exposure and compliance

The exposure dose and trial compliance are descriptively summarized, including safety evaluation and immunogenicity-testing compliance.

Immunogenicity and exploratory analysis

The GMT of neutralizing antibody for each group with corresponding 2-sided 95% CI will be estimated at each post-baseline time point using an analysis of covariance. The comparison of

GMT of neutralizing antibody between the treatment groups at each post-baseline time point will also be provided using an analysis of covariance.

The 95% CI of seroresponse using the Clopper-Pearson method will be provided. Cochran-Mantel-Haenszel method will be used for comparison of the seroresponse between the treatment groups.

The change in the number of IFN-γ positive (characterizing Th1) and IL-4 positive (characterizing Th2) T cell subsets at each post-baseline time point will be statistically described, and the nonparametric test will be applied for the statistical comparison between groups. Detailed statistical analysis methods are described in the SAP for further reference.

Safety Analysis

Safety analysis will be based on SS.

AEs and SAEs are encoded based on the *Medical Dictionary for Regulatory Activities* (MedDRA) and also based on the document the classified statistics was made according to the system organ class (SOC) and preferred term (PT). In this trial, the treatment emergent adverse events (TEAEs) are summarized, and the adverse medical conditions occurring before the study vaccination are listed. Unless otherwise specified, the adverse events as described below are TEAEs.

The incidence of AEs, SAEs and AESIs, the number and percentage of participants with AEs, SAEs and AESIs in each group will be summarized respectively. The 2-sided 95% CI will be also provided for the percentage of participants with any solicited AE for each treatment group using the Clopper-Pearson method. The adverse events related to the study vaccine, SAEs and AESIs will be listed.

12 MANAGEMENT OF CLINICAL TRIAL

12.1 DECLARATION

This study will be conducted in accordance with ICH GCP, the Declaration of Helsinki, the SOPs of the sponsor and its agents (e.g., CRO) and all applicable regulations.

12.2 ETHICS

The clinical trial protocol, ICF, Investigator's Brochure (IB) and other relevant documents should be submitted to the appropriate Ethics Committee (ERC) for approval prior to the start of the test. The test shall not be carried out in any form before the sponsor obtain written consent or approval from the appropriate EC. Any amendments to relevant documents, such as informed consent, to the clinical trial protocol must be implemented with the approval of the ERC.

The investigator and the personnel involved in the study should be familiar with the protocol and be able to prepare measures in advance, such as measures and reports in the event of SUSAR.
In the course of a clinical trial, if any SAEs or SUSAR related to clinical trial safety occurs that may affect the safety of the participant or the conduct of the study, the investigator should report the ERC as required by regulations.

12.3 INFORMED CONSENT

Participants must give informed consent to this study before receiving treatment in order to protect their legal rights and interests. It is the responsibility of the principal investigator or investigator of the clinical trial to fully and comprehensively introduce the purpose, methods, reasonable expected benefits, possible adverse reactions and risks of the study to the participants. At the same time, participants should be informed that the participation to the clinical trial is voluntary and that they have the right to withdraw from the test at any time without prejudice to their personal interests. ICFs signed by participants own or the legal representative must be obtained before any clinical trial related procedures are performed. The ICF is prepared in two copies, one for the participant and one for the filing.
Prior to obtaining informed consent, the investigator or designee should provide the participant with sufficient time and opportunity to ask about the details of the study and to decide whether to participate in the study. The process of informed consent should be documented in the progress notes on the day of screening visit.
The investigator is responsible for the informed consent process. If any information is obtained during the test relating to the participant's willingness to continue the test, the written informed consent must be updated and given to the participant to confirm the willingness to continue to participate. Ethical approval is required before the revised informed consent is provided to the participant.
By signing the informed consent, participants should also agree to allow the sponsor, the drug approval administration, the auditor and/or the sponsor's authorized clinical trial monitor to review the obtained raw data related to the clinical trial in compliance with the confidentiality statement.
The investigator should use the latest version of ICF and other information provided to participants as agreed by the ERC. If any information is obtained during the test relating to the participant's willingness to continue the test, the written ICF must be updated and given to the participant to confirm the willingness to continue to participate. Ethical approval is required before the revised ICF is provided to the participant. Participants may withdraw

unconditionally from the study at any time during the study, and participants will not be penalized for the withdrawal.

12.4 REVISION OF CLINICAL TRIAL PROTOCOL

During the course of the study, the sponsor should communicate with the investigator and make modifications to the protocol, which should be implemented only after the approval of the ERC. Any changes to the protocol, whether material or non-material, are required to be in writing. Approval from the ERCs of all study Sites is required for substantive protocol changes that will clearly affect the safety of participants, the scope of the study, or the scientific quality of the study. For the safety of all participants in the study, the above requirements shall not hinder the investigator or sponsor from taking any urgent actions. If the investigator deems that an immediate change of protocol is necessary for safety reasons, the sponsor's designated institution must be notified in time and the study Site ERC should be notified in accordance with the policies made by the ERC that approves the study, as well as local regulations and policies. Changes that only affect the management of the study do not require substantial protocol revision or ERC approval, but such changes must be notified to ERC. In these cases, the sponsor will send an official letter to the ERC detailing the changes.

12.5 PROTOCOL DEVIATION

The investigator should conduct the study in accordance with a protocol agreed by the sponsor and the regulatory authority (if necessary) and approved by the ERC.

During the test, the investigator should not deviate from the protocol unless urgent measures are taken to eliminate the immediate risk to participants. In the event of other unexpected circumstances that require deviation from the procedures specified in the protocol, the investigator should consult with the medical monitor (and the ERC, if necessary) to determine appropriate actions.

The study Site should record all protocol deviations in the participant's original data, including but not limited to the time of occurrence of protocol deviations, time of discovery, description of events, and measures taken, etc. In the event of a serious protocol deviation, the center should notify the medical monitor, Clinical Research Associate, or ERC promptly.

12.6 MONITORING

The sponsor and/or its agents (e.g., CRO) conduct Clinical Research Associating of the study. The Clinical Research Associate should follow the appropriate SOPs. The monitor should maintain regular communication with the investigator and sponsor.

Before the clinical trial: the monitor should confirm that the investigator has sufficient qualifications and resources to complete the test, that the clinical trial institution has the appropriate conditions to complete the test, including staffing and training, and that the laboratory is well equipped in good working order and is well-qualified for various tests related to the test. At the same time, the monitor should discuss with the investigator the specific items required as the original data and determine the nature and location of all the original data to ensure that the sponsor or investigator knows the source of the original data used to complete the eCRF.

During the clinical trial: the monitor will regularly visit the clinical site (online visit is allowed) to review the protocol compliance, data integrity, accuracy and consistency, as well as compliance with ICH GCP and relevant regulations. Depending on the risk assessment, remote centralized monitoring may be considered as a replacement or supplement to on-site monitoring. As necessary, the monitor will also provide clarification and additional training to help resolve on-site issues identified during the monitoring visit.

During the study period, the investigator should agree to direct access to all relevant documents by the monitor and ensure that he/she and relevant study staff meet with the monitor regularly to discuss the findings from the visit and any related issues.

12.7 QUALITY ASSURANCE AND AUDIT

During the study, the sponsor or sponsor's representative will conduct quality assurance audits of the study Site, databases and related documents. At the same time, the relevant regulatory authorities can also inspect the study Site, databases and relevant documents at their own discretion. The purpose is to determine whether the recording, analysis and reporting of these activities and data comply with the study protocol, GCP, ICH guidelines and any relevant regulatory requirements. During the process of audit or inspection, the investigator should support the audit or inspection and allow the auditor or inspector direct access to original data or documents, including all medical records, documents and letters related to the study, and informed consent documents for the clinical trial, etc.

12.8 INTELLECTUAL PROPERTY

All information obtained from the sponsor is the sponsor's intellectual property and thus must be kept strictly confidential by the investigator and all other relevant personnel and shall not be disclosed to third parties without the prior consent of the study sponsor.

12.9 PARTICIPANTS' PRIVACY

Study staff must ensure that the privacy of participants is maintained. For all submissions to

the sponsor, participants shall be identified only by the participant code and name abbreviation, but not by the participant name or admission number. The investigator must keep the name, address and other private information of participants in the clinical trial in strict confidence and shall not submit it to the sponsor.

12.10 MONITORING BOARD

12.10.1 DATA AND SAFETY MONITORING BOARD (DSMB)

This study were organized by the sponsor to establish a DSMB to periodically evaluate the progress of clinical trial, and to advise the sponsor about whether to continue, modify or discontinue the ongoing clinical trial based on the data results.

DSMB members include experts in the clinical research field of vaccine, biostatisticians and epidemiologists, etc. DSMB should have prior knowledge of the clinical trial protocol, develop and sign the DSMB regulations for this study. The primary task of DSMB are to review the safety data of participants reported after the study vaccination for the participants' safety and interests. At the same time, DSMB also monitors the entire process of the clinical trial, including protocol compliance, recruitment status, and drop-out rate of participants, to ensure the validity and credibility of the test.

For more information, see the DSMB charter and carry out the work as required by the DSMB charter.

13 FINANCE AND INSURANCE

The sponsor will provide insurance that meets regulatory and legal requirements. The sponsor has purchased liability insurance for this clinical trial and the liability policy complies with local laws and requirements. The liability insurance policy will be submitted to the ERC, IRB or regulatory authority as required by the corresponding country.

14 PUBLISHING AND DATA SHARING POLICIES

The author should be identified before the writing of the manuscript. Unless the consent of Sinocelltech Ltd. is obtained, no individual writing is allowed to be published before the final report of the study is completed. With respect to the manuscript and publication, the decision of Sinocelltech Ltd. has the right of final decision.

15 APPENDICES

15.1 APPENDIX I: TOXICITY RATING SCALE FOR HEALTHY ADULT AND ADOLESCENT VOLUNTEERS IN PREVENTIVE VACCINE CLINICAL TRIAL - FDA STANDARD

Table 8 Scale of clinical abnormalities

Local reaction of the injected product	Mild (grade 1)	Moderate (grade 2)	severe (grade 3)	Potentially life-threatening (grade 4)
**Pain**	Does not interfere with activity	Repeated use of non-narcotic pain reliever > 24 hours or interferes with activity	Any use of narcotic pain reliever or prevents daily activity	Emergency care or hospitalization required
**Tenderness**	Mild discomfort to touch	Discomfort with movement	Significant discomfort at rest	ER visit or hospitalization
**Erythema/redness***	2.5-5 cm	5.1-10 cm	> 10 cm	Necrotic or exfoliative dermatitis
**Induration/swelling****	2.5 – 5 cm and does not interfere with activity	5.1 – 10 cm or interferes with activity	> 10 cm or prevents daily activity	Necrosis

* In addition to grading of the measured local reactions at the maximum diameter, diameter changes should also be recorded;
** Induration or swelling should be assessed and graded using a functional scale and actual measurements.

Table 9 Scale of vital signs

Vital signs*	Mild (grade 1)	Moderate (grade 2)	Severe (grade 3)	Potentially life-threatening (grade 4)
**Fever °C**<br>°F****	38.0 – 38.4<br>100.4 – 101.1	38.5 – 38.9<br>101.2 – 102.0	39.0 – 40<br>102.1 – 104	> 40<br>> 104
**Tachycardia (bpm)**	101 – 115	116 – 130	> 130	Emergency care or hospitalization required due to arrhythmias
**Bradycardia (bpm)*****	50 – 54	45 – 49	< 45	Emergency care or hospitalization required due to arrhythmias
**High blood pressure (systolic) mmHg**	141 – 150	151 – 155	> 155	Emergency care or hospitalization resulted from malignant hypertension
**High blood pressure (diastolic) mmHg*****	91 – 95	96 – 100	> 100	Emergency care or hospitalization resulted from malignant hypertension
**Low blood pressure (systolic)**	85 – 89	80 – 84	< 80	Emergency care or hospitalization

**mmHg**				resulted from hypotensive shock
**Respiration rate (times per minute)**	17 – 20	21 – 25	> 25	Endotracheal intubation is required

* All vital signs should be measured for participants after rest;
** Oral temperature, with no hot or cold drinks or smoking before testing;
*** Resting heart rate is between 60 and 100 beats per minute. For some healthy participants, such as certain athletes, the characteristics of bradycardia should be judged clinically.

Table 10 Scale of adverse events

Systemic reaction	Mild (grade 1)	Moderate (grade 2)	Severe (grade 3)	Potentially life-threatening (grade 4)
**Nausea and vomiting**	No interference with activity or 1 – 2 episodes/24 hours	Some interference with activity or > 2 episodes/24 hours	Prevents daily activity, requires outpatient IV hydration	ER visit or hospitalization for hypotensive shock
**Diarrhea**	2 – 3 loose stools or < 400 gms/24 hours	4 – 5 stools or 400 – 800 gms/24 hours	6 or more watery stools or > 800gms/24 hours or requires outpatient IV hydration	ER visit or hospitalization
**Headache**	No interference with activity	Repeated use of non-narcotic pain reliever > 24 hours or some interference with activity	Significant; any use of narcotic pain reliever or prevents daily activity	ER visit or hospitalization
**Fatigue**	No interference with activity	Some interference with activity	Significant; prevents daily activity	ER visit or hospitalization
**Myalgia**	No interference with activity	Some interference with activity	Significant; prevents daily activity	ER visit or hospitalization
**Other diseases or clinical adverse events (as defined in applicable regulations)**	No interference with activity	Some interference with activity not requiring medical intervention	Prevents daily activity and requires medical intervention	ER visit or hospitalization

Table 11 Abnormal Results of Laboratory Examinations

Serum*	Mild (grade 1)	Moderate (grade 2)	Severe (grade 3)	Potentially life-threatening (grade 4)**
**Sodium - hyponatremia mEq/L**	132 – 134	130 – 131	125 – 129	< 125
**Sodium - hypernatremia mEq/L**	144 – 145	146 – 147	148 – 150	> 150
**Potassium - hyperkalemia mEq/L**	5.1 – 5.2	5.3 – 5.4	5.5 – 5.6	> 5.6
**Potassium - hypokalemia mEq/L**	3.5 – 3.6	3.3 – 3.4	3.1 – 3.2	< 3.1
**Glucose - hypoglycemia**	65 – 69	55 – 64	45 – 54	< 45

Serum*	Mild (grade 1)	Moderate (grade 2)	Severe (grade 3)	Potentially life-threatening (grade 4)**
**mg/dL**				
**Glucose - hyperglycemia**<br>**Fasting mg/dL**<br>**Random mg/dL**	100 – 110<br>110 – 125	111 – 125<br>126 – 200	>125<br>>200	Insulin treatment required or hyperosmolar coma
**BUN mg/dL**	23 – 26	27 – 31	> 31	Hemodialysis required
**Serum creatinine mg/dL**	1.5 – 1.7	1.8 – 2.0	2.1 – 2.5	> 2.5 or hemodialysis required
**Calcium - hypocalcemia mg/dL**	8.0 – 8.4	7.5 – 7.9	7.0 – 7.4	< 7.0
**Calcium - hypercalcemia mg/dL**	10.5 – 11.0	11.1 – 11.5	11.6 – 12.0	> 12.0
**Magnesium - hypomagnesemia mg/dL**	1.3 – 1.5	1.1 – 1.2	0.9 – 1.0	< 0.9
**Phosphorus - hypophosphatemia mg/dL**	2.3 – 2.5	2.0 – 2.2	1.6 – 1.9	< 1.6
**CPK mg/dL**	1.25 – 1.5×ULN***	1.6 – 3.0×ULN	3.1 –10×ULN	> 10×ULN
**Albumin – hypoalbuminemia g/dL**	2.8 – 3.1	2.5 – 2.7	< 2.5	--
**Total protein – hypoproteinemia g/dL**	5.5 – 6.0	5.0 – 5.4	< 5.0	--
**Alkaline phosphatase increased**	1.1 – 2.0×ULN	2.1 – 3.0×ULN	3.1 – 10×ULN	> 10×ULN
**Liver function test - ALT and AST increased**	1.1 – 2.5×ULN	2.6–5.0×ULN	5.1 – 10×ULN	> 10×ULN
**Bilirubin increased - with increased liver function indicators**	1.1 – 1.25×ULN	1.26 – 1.5×ULN	1.51 – 1.75×ULN	> 1.75×ULN
**Bilirubin increased - normal liver function**	1.1 – 1.5×ULN	1.6 – 2.0×ULN	2.0 – 3.0×ULN	> 3.0×ULN
**Cholesterol**	201 – 210	211 – 225	> 226	--
**Trypsin, amylase and lipase**	1.1 – 1.5×ULN	1.6 – 2.0×ULN	2.1 – 5.0×ULN	> 5.0×ULN

* The laboratory testing values provided in the table as a guide are determined based on the normal values of the medical facilities. Therefore, a specified range of normal reference values should be provided to prove its applicability.

** Clinical signs and symptoms associated with the abnormal results of laboratory examinations may result in a potentially life-threatening (grade 4) presentation of abnormalities. For example, if the participant had a new seizure attack associated with low sodium level, a sodium level as low as grade 3 (125-129mE/L) will also be recorded as a grade 4 hyponatremia event.

*** "ULN" represents the upper limit of the normal range.

Table 12 Hematological Abnormality

Hematology*	Mild (grade 1)	Moderate (grade 2)	Severe (grade 3)	Potentially life-threatening (grade 4)
**Hemoglobin (female) gm/dL**	11.0 – 12.0	9.5 – 10.9	8.0 – 9.4	< 8.0
**Changes in hemoglobin (female)**	Increase - 1.5	1.6 – 2.0	2.1 – 5.0	> 5.0

**compared to baseline gm/dL**				
**Hemoglobin (male) gm/dL**	12.5 – 13.5	10.5 – 12.4	8.5 – 10.4	< 8.5
**Changes in hemoglobin (male) compared to baseline gm/dL**	Increase - 1.5	1.6 – 2.0	2.1 – 5.0	> 5.0
**Leukocytes increased - cell/mm^3**	10,800 – 15,000	15,001 – 20,000	20,001 – 25, 000	> 25,000
**Leukocytes decreased - cell/mm^3**	2,500 – 3,500	1,500 – 2,499	1,000 – 1,499	< 1,000
**Lymphocyte decreased - cell/mm^3**	750 – 1,000	500 – 749	250 – 499	< 250
**Neutrophil decreased - cell/mm^3**	1,500 – 2,000	1,000 – 1,499	500 – 999	< 500
**Eosnophils decreased - cell/mm^3**	650 – 1500	1501 - 5000	> 5000	Eosinophilia
**Platelet count decreased - cell/mm^3**	125,000 – 140,000	100,000 – 124,000	25,000 – 99,000	< 25,000
**Prolonged coagulation time (PT)**	1.0 – 1.10×ULN**	1.11 – 1.20×ULN	1.21 – 1.25×ULN	> 1.25×ULN
**Prolonged partial thromboplastin time (PTT)**	1.0 – 1.2×ULN	1.21 – 1.4×ULN	1.41 – 1.5×ULN	> 1.5×ULN
**Fibrinogen increased mg/dL**	400 – 500	501 – 600	> 600	--
**Fibrinogen decreased mg/dL**	150 – 200	125 – 149	100 – 124	< 100 or related to the total amount of blooding, or occurance of DIC

* The laboratory testing values provided in the table as a guide are determined based on the normal values of the medical facilities. Therefore, a specified range of normal reference values should be provided to prove its applicability.
** "ULN" represents the upper limit of the normal range.

Table 13 Abnormalities of Urine Routine Examination

**Urine***	**Mild (grade 1)**	**Moderate (grade 2)**	**Severe (grade 3)**	**Potentially life-threatening (grade 4)**
**Protein**	Micro amount	1+	2+	Hospitalization or dialysis treatment
**Glucose**	Micro amount	1+	2+	Hospitalization due to hyperglycemia
**Red blood cells (microscopic examination) Number of red blood cells per high power field (rbc/hpf)**	1-10	11-50	> 50 and/or whole blood	Hospitalization or Packed Red Blood Cells (PRBC) infusion required

* The laboratory testing values provided in the table as a guide are determined based on the normal values of the medical facilities. Therefore, a specified range of normal reference values should be provided to prove its applicability.

16 REFERENCES

1. *World Health Organization*. Available from: https://covid19.who.int/.
2. *National Health Commission of the People's Republic of China. Diagnosis and treatment plan for COVID-19 (trial version 8).* Chin J Clin Infect Dis., 2020. **13**(5).
3. *Sinocelltech Ltd. Bivalent SARS-CoV-2 Trimeric Spike Protein Vaccine (SCTV01C) Investigator's Brochure.*
4. *National Medical Product Administration. General Principles of Technical Review for Preclinical Safety Assessment of Prophylactic Biologics.*
5. Karim, S.S.A., *Vaccines and SARS-CoV-2 variants: the urgent need for a correlate of protection.* Lancet, 2021. **397**(10281): p. 1263-1264.
6. Wang, P., et al., *Antibody resistance of SARS-CoV-2 variants B.1.351 and B.1.1.7.* Nature, 2021. **593**(7857): p. 130-135.
7. Abu-Raddad, L.J., et al., *Effectiveness of the BNT162b2 Covid-19 Vaccine against the B.1.1.7 and B.1.351 Variants.* N Engl J Med, 2021. **385**(2): p. 187-189.
8. Shinde, V., et al., *Efficacy of NVX-CoV2373 Covid-19 Vaccine against the B.1.351 Variant.* N Engl J Med, 2021. **384**(20): p. 1899-1909.
9. Madhi, S.A., et al., *Efficacy of the ChAdOx1 nCoV-19 Covid-19 Vaccine against the B.1.351 Variant.* N Engl J Med, 2021. **384**(20): p. 1885-1898.
10. *A study to evaluate the immunogenicity and safety of mRNA-1273.211 vaccine for COVID-19 variants. ClinicalTrials.gov (NCT04927065).*
11. *The Phase I Clinical trial of booster vaccination of adenovirus type-5 vectored COVID-19 vaccine. ClinicalTrials.gov (NCT04568811).*
12. *Immunogenicity and safety of a third dose, and immune persistence of CoronaVac vaccine in healthy adults aged 18-59 years: interim results from a double-blind, randomized, placebo-controlled phase 2 clinical trial. ClinicalTrials.gov (NCT04979949).*
13. Borobia, A.M., et al., *Immunogenicity and reactogenicity of BNT162b2 booster in ChAdOx1-S-primed participants (CombiVacS): a multicentre, open-label, randomised, controlled, phase 2 trial.* Lancet, 2021. **398**(10295): p. 121-130.
14. *Food and Drug Administration. COVID-19: Developing Drugs and Biological Products for Treatment or Prevention Guidance for Industry, 2021.*

Protocol Title: A randomized, double-blind, and positive-controlled Phase III clinical trial to evaluate the immunogenicity and safety of SCTV01C (A Bivalent SARS-CoV-2 Trimeric Spike Protein Vaccine) and SCTV01E (A COVID-19 Alpha/Beta/Delta/Omicron Variants S-Trimer Vaccine) in population aged ≥18 years previously vaccinated with either inactivated or mRNA COVID-19 vaccine or previously diagnosed with COVID-19

Protocol No.: SCTV01C-E-01-UAE-1

Protocol Version: 2.0

Version Date: May 20, 2022

Summary of Major Changes from Protocol Version 1.0 to Version 2.0

**Section # and Name**	**Description of Change**	**Brief Rationale**
Title Page, Synopsis, Headers and footers	Updated the version and date.	Reflect the new date of the protocol.
Synopsis; Section 7.1 and 7.3	Changed positive control vaccine (mRNA-1273) to mRNA COVID-19 vaccine and the detailed information refers to medicine specification.	mRNA-1273 may be not available, so the new other vaccine may be used in the study. Furthermore, many specifications were used in marked. The confirmed specification will be used according to the available vaccine.
Section 8.2.1 and 9.3.4	Changed "two samples of nasal/nasopharyngeal/throat swab should be collected within 72 hours" to "a sample collection of nasal/nasopharyngeal/throat swab should be collected within 72 hours and be divided into two parts"	According to library procedure.
Section 9.3.5	The confirmed COVID-19 cases follow up was changed to only follow up the participant every 3 to 7 days until the symptoms were sustained resolution.	More consistent with clinical procedure.

Protocol Title: A randomized, double-blind, and positive-controlled Phase III clinical trial to evaluate the immunogenicity and safety of SCTV01C (A Bivalent SARS-CoV-2 Trimeric Spike Protein Vaccine) and SCTV01E (A COVID-19 Alpha/Beta/Delta/Omicron Variants S-Trimer Vaccine) in population aged ≥18 years previously vaccinated with either inactivated or mRNA COVID-19 vaccine or previously diagnosed with COVID-19

Protocol No.: SCTV01C-E-01-UAE-1

Protocol Version: 3.0

Version Date: June 6, 2022

Summary of Major Changes from Protocol Version 2.0 to Version 3.0

Section # and Name	Description of Change	Brief Rationale
Title Page, Synopsis, Headers and footers	Updated the version and date.	Reflect the new date of the protocol.
Synopsis, Section 6.1 and 8.2.1	The interval time was changed to 14 days regarding the result of nucleic acid test for SARS-CoV-2 prior to vaccination at screening.	Per the suggestion of PI

Protocol Title: A randomized, double-blind, and positive-controlled Phase III clinical trial to evaluate the immunogenicity and safety of SCTV01C (A Bivalent SARS-CoV-2 Trimeric Spike Protein Vaccine) and SCTV01E (A COVID-19 Alpha/Beta/Delta/Omicron Variants S-Trimer Vaccine) in population aged ≥18 years previously vaccinated with either inactivated or mRNA COVID-19 vaccine or previously diagnosed with COVID-19

Protocol No.: SCTV01C-E-01-UAE-1

Protocol Version: 4.0

Version Date: September 22, 2022

Summary of Major Changes from Protocol Version 3.0 to Version 4.0

Section # and Name	Description of Change	Rationale
Title Page, Synopsis, Headers and footers	Updated the version and date.	• Reflect the new date of the protocol.
Synopsis, Section 2.2	Moved secondary endpoint “GMT of nAb against Omicron BA.1 (B.1.1.529) variant on D28” to primary endpoint in Cohort 2. And added GMT of nAb against Omicron BA.5 variant on D28 as secondary endpoint in Cohort 1 and Cohort 2.	• The main circulating strain of SARS-CoV-2 is Omicron. It will be more meaningful to achieve significant immunogenicity results against omicron variant.<br>• The evaluation of GMT of nAb against Omicron variant on D28 was a key secondary endpoint in previous protocol and the evaluation was also included in multiplicity analysis. In addition, the analysis of immunogenicity data of SCTV01C from Phase I/II study in UAE, showed that the titers of nAb and total IgG dramatically increased and were obviously higher than that induced by other inactivated or mRNA COVID-19 vaccine. SCTV01E was designed based on alpha, beta, delta and omicron variants, so SCTV01E should probably have better immunogenicity against omicron variant than SCTV01C which was designed based on alpha and beta variants. Hence, we moved secondary endpoint “GMT of nAb against Omicron variant on D28” to primary

Section # and Name	Description of Change	Rationale
		endpoint in Cohort 2.
Synopsis, Section 11	• For Hypothesis, the BA.5 comparison between SCTV01C/E and active control were added into the list of hypothesis tests.<br>• The multiplicity strategy was adjusted by separating Cohort 1 and Cohort 2, and hypothesis tests for BA.5 were added into multiplicity procedure.	• BA.5 reflect most current variant of interest<br>• Cohort 1 and Cohort 2 are independent, separating them will not inflate the Type I error; add, BA.5 hypothesis tests for BA.5 were added into multiplicity procedure to control the Type I error for corresponding tests.
Table 1 Schedule of Activities and Section 6.1, 8.2.3 and 9.3.4	• Added SARS-CoV-2 rapid antigen test for confirmation of COVID-19 case.	• SARS-CoV-2 rapid antigen test is also one method to confirm the infection with SARS-CoV-2.
Synopsis, Section 1.0	• Updated preclinical data of SCTV01E	• More scientific.
Section 9.1.5.3	• Deleted artificial abortion from SAE list	• If the artificial abortion is not medically necessary, it is not considered as SAE.

Statistical Analysis Plan

Protocol SCTV01C-E-01-UAE-1

A randomized, double-blind, and positive-controlled Phase III clinical trial to evaluate the immunogenicity and safety of SCTV01C (A Bivalent SARS-CoV-2 Trimeric Spike Protein Vaccine) and SCTV01E (A COVID-19 Alpha/Beta/Delta/Omicron Variants S-Trimer Vaccine) in population aged ≥18 years previously vaccinated with either inactivated or mRNA COVID-19 vaccine or previously diagnosed with COVID-19

Statistical Analysis Plan
(SAP)

Version: **1.0**

Date: 29SEP2022

神州细胞
SinoCellTech

This document contains confidential information belonging to SinoCellTechExcept as otherwise agreed to in writing, by accepting or reviewing this document, you agree to hold this information in confidence and not copy or disclose it to others (except where required by applicable law) or use it for unauthorized purposes. In the event of any actual or suspected breach of this obligation, SinoCellTech must be promptly notified.

TABLE OF CONTENT

TABLE OF CONTENT 3
VERSION HISTORY 5
LIST OF ACRONYM/ABBREVIATION 5
1. INTRODUCTION 6
1.1. Study Objectives 6
1.2. Study Design 6
1.2.1. Overall Study Design 6
1.2.2. Assessment Schedule 8
1.2.3. Schedule of Investigational Product (IP) Administration 9
1.3. Sample Size and Power considerations 9
1.4. Blinding 10
2. ENDPOINTS AND BASELINE VARIABLES: DEFINITIONS AND CONVENTIONS 11
2.1. Primary Endpoint(s) 11
2.2. Secondary Endpoint(s) 11
2.3. Baseline Variables 13
2.4. Safety Endpoints 13
2.4.1. Adverse Events (AE) 13
2.5. Immunogenicity Endpoints 14
2.6. Covariates 15
3. ANALYSIS SETS 16
4. GENERAL METHODOLOGY AND CONVENTIONS 17
4.1.1. Statistical Hypotheses 17
4.2. General Methods 19

4.2.1. General Presentation consideration 19
4.2.2. Methods to Manage Missing Data 19
4.3. Multiplicity 20
5. ANALYSES AND SUMMARIES 20
5.1. Baseline and Other Summaries and Analyses 20
5.1.1. Study Conduct and Participant Disposition 21
5.1.2. Demographics and Baseline Characteristics 21
5.1.3. Medical history 22
5.1.4. Concomitant Medications and Nondrug Treatments 22
5.2. Study Treatment Exposure 22
5.3. Efficacy Analyses 22
5.3.1. Primary Analysis 22
5.3.1.1. Sensitivity/Supplementary Analyses 25
5.3.1.2. Subgroup Analyses 25
5.3.2. Analyses of Secondary Efficacy Endpoints 25
5.4. Safety Summaries and Analyses 26
5.4.1. Adverse Events 26
5.4.2. Deaths 28
5.4.3. Analysis of Other Safety Data 28
6. INTERIM ANALYSES 28
7. CHANGES IN THE CONDUCT OF THE STUDY OR PLANNED ANALYSIS 28
8. REFERENCES 28

VERSION HISTORY

Table 1. Summary of Changes

Version/ Date	Associated Protocol Amendment	Rationale	Author	Specific Changes
1<br>12 Oct 2021	V4 Sep 22, 2022	N/A	Dongfang LIU	First Version

LIST OF ACRONYM/ABBREVIATION

Abbreviation	Term
AE	Adverse Event
AESI	Adverse Event of Special Interest
CMH	Cochran-Mantel-Haenszel
FAS	Full Analysis Set
GMT	Goemetric Mean Titer
GMR	Goemetric Mean Ratio
ITT	Intent-To-Treat Set
LLOQ	Lower Limit of Quantitation
PPS	Per-Protocol Set
SAE	Serious Adverse Event
ULOQ	Upper Limit of Quantitation

1. INTRODUCTION

This statistical analysis plan (SAP) provides the detailed methodology for summary and statistical analyses of the data collected in Study SCTV01C-E-01-UAE-1. This document may modify the plans outlined in the protocol; however, any major modifications of the primary endpoint definition or its analysis will also be reflected in a protocol amendment.The analyses described in this SAP are based upon the following study documents:

- Study Protocol, Version 4 (Sep 22, 2022)

1.1. Study Objectives

Primary objectives of the study are as below:

- To evaluate the immunogenicity of SCTV01C
- To evaluate the immunogenicity of SCTV01E

Secondary objectives of each part of the study are as below:

- To evaluate the cellular immune response of SCTV01C
- To evaluate the cellular immune response of SCTV01E
- To evaluate the safety of SCTV01C within 180 days after the vaccination
- To evaluate the safety of SCTV01E within 180 days after the vaccination

1.2. Study Design

1.2.1. Overall Study Design

The study is a randomized, double-blind, and positive-controlled Phase III booster study. It will evaluate the immunogenicity and safety of one dose of SCTV01C or SCTV01E as booster compared with either one dose of Sinopharm inactivated COVID-19 vaccine (Cohort 1) or one dose of mRNA COVID-19 vaccine (Cohort 2).

Approximately 1,800 participants aged 18 years old and above will be enrolled in this study. 1,350 participants who previously received Sinopharm inactivated COVID-19 vaccine will be enrolled to Cohort 1. 450 participants who previously received mRNA COVID-19 vaccine will be enrolled to Cohort 2.

In Cohort 1, 300 participants who were previously fully vaccinated with 2 or 3 doses of Sinopharm inactivated COVID-19 vaccine and with no previous COVID-19 history will form an

immunogenicity subgroup (Subgroup 1) for nAb tests, and will be randomly assigned to SCTV01C Group, SCTV01E Group and the Sinopharm inactivated COVID-19 vaccine Group in a ratio of 1:1:1. The 300 participants for nAb tests will be stratified by age (18-54 years, ≥55 years), number of doses of previously received COVID-19 vaccines (2, 3), and interval between previous vaccination and the study vaccination (3-5 months, 6-8 months, 9-12 months, 13-24 months). The first 150 participants will form a cellular immune response subgroup for cellular immune response tests.

In Cohort 2, 450 participants who previously received 2 or 3 doses of mRNA COVID-19 vaccine will be randomly assigned to SCTV01C Group, SCTV01E Group and the mRNA COVID-19 vaccine Group in a ratio of 1:1:1. Participants will be stratified by age (18-54 years, ≥55 years), number of doses of previously received COVID-19 vaccines (2, 3), SARS-CoV-2 nucleocapsid antibody (positive or negative), and interval between previous vaccination and the study vaccination (3-5 months, 6-8 months, 9-12 months, 13-24 months). In Cohort 2, the number of participants with negative SARS-CoV-2 nucleocapsid antibody test result should not be less than 225. All participants will have nAb tests. The first 150 participants with negative SARS-CoV-2 nucleocapsid antibody test result will form a cellular immune subgroup for cellular immune response tests.

The study procedure is described as **Figure 1** and **Figure 2:**

Figure 1. Study design for Cohort 1

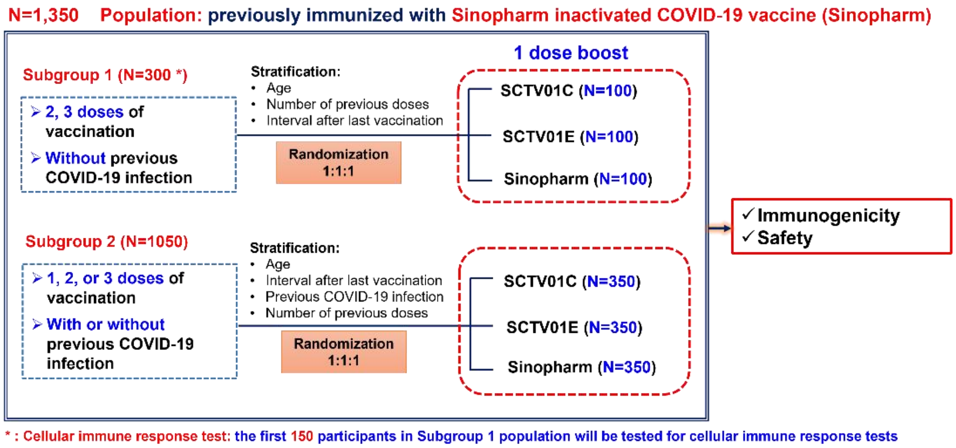

Figure 2. Study design for Cohort 2

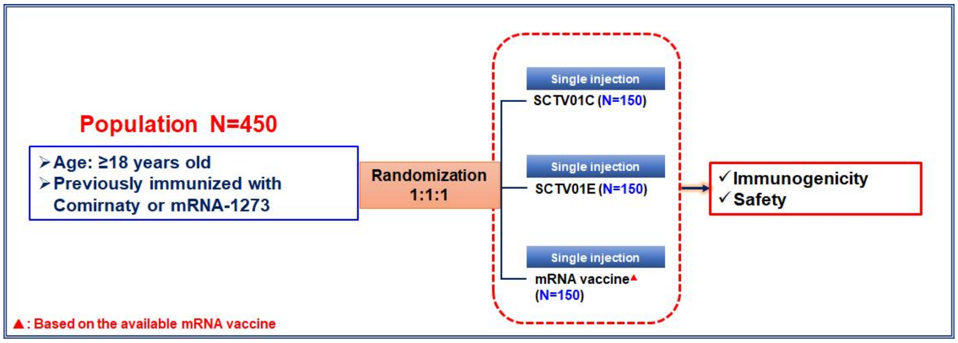

1.2.2. Assessment Schedule

The assessment schedule are listed in **Table 1**. More details can be found in the protocol.

Table 1. Schedule of Activities

	Screening period	Vaccination	Follow-up period		
**Visit**	**V1**	**V2**	**V3**	**V4**	**V5**
**Planned visit date**	**D-14～D0**	**D0**	**D7**	**D28**	**D180 (EOS)**
**Visit window period**	/	/	+2 days	+3 day	±7 days
**Management and general procedures**					
Signing the informed consent form	●				
Confirm participant meets inclusion and exclusion criteria	●	●			
Demographic data	●				
Recording the medical history	●				
Assigning the screening number	●				
Physical examination	●				
Vital signs	●	●			
Nasal/pharyngeal/throat swab nucleic acid test or rapid antigen test	●				
SARS-CoV-2 nucleocapsid antibody (only in Cohort 2)	●			●	
HIV testing	●				
Urine pregnancy test (for women of childbearing potential only)	●	●			
Randomization		●			
Vaccination		●			

	**Screening period**	**Vaccination**	**Follow-up period**		
**Visit**	**V1**	**V2**	**V3**	**V4**	**V5**
**Planned visit date**	**D-14～D0**	**D0**	**D7**	**D28**	**D180 (EOS)**
**Visit window period**	/	/	**+2 days**	**+3 day**	**±7 days**
Viral sequencing				●	
**Immunogenicity follow-up visit**					
Neutralizing antibodies test for Delta, Omicron variants and other variants		●		●	●
Cellular immune response		●		●	
**Safety follow-up visit**					
Solicited AEs		Record solicited AE			
Unsolicited Aes		Record unsolicited AE			
SAEs and AESIs		●	●	●	●
Observing for at least 30 minutes after the vaccination		●			
Distributing the vaccination record cards (VRCs)		●			
Reviewing the VRCs			●	●	
Distributing the thermometer		●			
Recording the concomitant medication	●	●	●	●	●

1.2.3. Schedule of Investigational Product (IP) Administration

All participiants will receive one booster vaccination, SCTV01C 20μg, SCTV01E 30μg or the active control (Sinopharm inactivated COVID-19 vaccine for Cohort 1 and mRNA COVID-19 vaccine for Cohort 2) via intramuscular injection at Visit 2 (D0).

1.3. Sample Size and Power considerations

Totally 1800 participants, 1350 in Cohort 1 and 450 in Cohort 2, aged ≥18 years who were previously vaccinated with either inactivated or mRNA COVID-19 vaccine or previously diagnosed with COVID-19 will be enrolled.

In Cohort 1, 300 of 1350 participants (100 in SCTV01C Group, 100 in SCTV01E Group, 100 in Sinopharm COVID-19 vaccine Group) will be assessed for both immunogenicity and safety of the investigational product. The rest 1050 will be assessed for safety. The number of participants for immunogenicity assessment, i.e., 300, is deterimed to achieve at least 80% power to demonstrate SCTV01E/SCTV01C is superior to the Sinopharm at a significance level of one-sided 0.025 based on below assumptions:

- The standard deviation of neutralizing antibody titers under log10 transformation: 0.4
- Goemetric mean ratio (GMR), SCTV01E/C vs. mRNA vaccine:1.6
- Proportion of participants not evaluable due to being COVID-19 infected before Day 28 OR missing the data: 10%

In Cohort 2, all 450 participants (150 in SCTV01C Group, 150 in SCTV01E Group, 150 in mRNA COVID-19 vaccine Group) will be assessed for both immunogenicity and safety of the investigational product. This number is determined to achieve at least 90% power to demonstrate SCTV01E/SCTV01C is non-inferiority to the mRNA COVID-19 vaccine at a significance level of one-sided 0.025 based on below assumptions:

- The standard deviation of neutralizing antibody titers under log10 transformation: 0.4
- Goemetric mean ratio (GMR), SCTV01E/C vs. mRNA vaccine:1
- Non-inferiority margin: 0.67
- Proportion of participants not evaluable due to being COVID-19 infected before Day 28 OR missing the data: 10%

1.4. Blinding

This trial is designed as double-blinded. An unblinded team at the study site will be set. The unblinded study site personnel will manage vaccine logistics, preparation, and administration so as to maintain the blind from the remainder of the study site personnel and participants. The

unblinded study site personnel administer study vaccine, but will not be involved in study-related assessments or have participant contact for data collection after administration of trial vaccine.

In case of an emergency, if unblinding is necessary for individual participants due to serious adverse events, in order to give corresponding emergency treatments, unblinding under emergency can be carried out.

2. ENDPOINTS AND BASELINE VARIABLES: DEFINITIONS AND CONVENTIONS

2.1. Primary Endpoint(s)

The primary endpoint(s) of each part of the study are as follows:

Cohort 1

- GMT of nAb against Delta variant on D28
- GMT of nAb against Omicron BA.1 variant on D28

Cohort 2

- GMT of nAb against Omicron BA.1 variant on D28
- GMT of nAb against Delta variant on D28

2.2. Secondary Endpoint(s)

The secondary endpoints are as follows:

Cohort 1

Immunogenicity

- GMT of nAb against Delta variant on D180
- GMT of nAb against Omicron BA.1 variant on D180
- GMT of nAb against Omicron BA.5 variant on D28
- Number of IFN-γ positive (characterizing Th1) and IL-4 positive (characterizing Th2) T cell subsets on D28

- Seroresponse of nAb (defined as a change from below the low limit of quantitation [LLOQ] to equal to or above LLOQ, or a ≥4-fold rise if baseline is equal to or above LLOQ in nAb to Delta variant from D0) rates on D28
- Seroresponse of nAb (defined as a change from below LLOQ to equal to or above LLOQ, or a ≥4-fold rise if baseline is equal to or above LLOQ in nAb to Omicron variant from D0) rates on D28

Safety

- Incidence and severity of solicited AEs of SCTV01C from D0 to D7
- Incidence and severity of all unsolicited AEs of SCTV01C from D0 to D28
- Incidence and severity of SAEs and AESIs of SCTV01C within 180 days
- Incidence and severity of solicited AEs of SCTV01E from D0 to D7
- Incidence and severity of all unsolicited AEs of SCTV01E from D0 to D28.
- Incidence and severity of SAEs and AESIs of SCTV01E within 180 days.

Cohort 2

Immunogenicity

- GMT of nAb against Delta variant on D180.
- GMT of nAb against Omicron BA.1 variant on D180.
- GMT of nAb against Omicron BA.5 variant on D28.
- Number of IFN-γ positive (characterizing Th1) and IL-4 positive (characterizing Th2) T cell subsets on D28.
- Seroresponse of nAb (defined as a change from below the low limit of quantitation [LLOQ] to equal to or above LLOQ, or a ≥4-fold rise if baseline is equal to or above LLOQ in nAb to Delta variant from D0) rates on D28.
- Seroresponse of nAb (defined as a change from below LLOQ to equal to or above LLOQ, or a ≥4-fold rise if baseline is equal to or above LLOQ in nAb to Omicron variant from D0) rates on D28.

Safety

- Incidence and severity of solicited AEs of SCTV01C from D0 to D7.
- Incidence and severity of all unsolicited AEs of SCTV01C from D0 to D28.
- Incidence and severity of SAEs and AESIs of SCTV01C within 180 days.
- Incidence and severity of solicited AEs of SCTV01E from D0 to D7.
- Incidence and severity of all unsolicited AEs of SCTV01E from D0 to D28.
- Incidence and severity of SAEs and AESIs of SCTV01E within 180 days.

2.3. Baseline Variables

The baseline value is defined as the last valid assessment before the vaccination. For the immunogenicity assessments, if baseline assessment at Visit 2 (Day 0) is the below LLOQ, half of the LLOQ wll be used as the baseline value. If the baseline is >ULOQ, the ULOQ will be used as the baseline value.

2.4. Safety Endpoints

2.4.1. Adverse Events (AE)

Solicited AEs

Solicited AEs are divided local adverse events and systemic adverse events. See Table 2 for detailed information.

Table 2. List of solicited AE

Solicited local AEs	Solicited systemic AEs
• Pain at the injection site<br>• Tenderness at the injection site<br>• Erythema at the injection site<br>• Redness at the injection site<br>• Swelling at the injection site<br>• Induration at the injection site	• Fever<br>• Nausea<br>• Vomiting<br>• Headache<br>• Fatigue<br>• Myalgia<br>• Arthralgia, joint pain<br>• Chill

Solicited AE were collected within the first 30 minutes after the study vaccination, and 7 days after the study vaccination (data collected from day 0 to day 7).

Unsolicited AEs

Unsolicited AEs are not specified for active monitoring , and also include those that held the same name with the solicited AE occur after the solicitation period, and are primarily spontaneously reported by participants, observed by the investigator, or noted during review of medical records or source documents.

2.5. Immunogenicity Endpoints

Live virus neutralization antibody agaigst COVID-19 were assessed at Baseline, Day 28, and Day 180 for Delta, Omicron variants respectively.

Cellular immune response, including IFN-γ and IL-4, were assessed at Baseline and Day 28.

Extreme value handling convention for immunogenicity data

In order to appropriately manage extreme values (< lower limit of quantification (LLOQ) or > Upper limit of quantification (ULOQ)), the following computational rule is applied to the values provided in the clinical database during the analysis:

- If a value is < LLOQ, half of the LLOQ will be used for analysis
- If a value is between LLOQ and ULOQ, the reported value will be used for analysis
- If a value is > ULOQ, use ULOQ, ULOQ will be used for analysis

Fold-increase

The derived endpoint fold-incrase is driven by both pre-vaccination and post-vaccination values， and is computed as individual titer ratio:

Fold increase = Post-vaccination value/Baseline value

If any of the pre-vaccination or post-vaccination values is missing, then fold-increase is missing for the corresponding timepoint(s).

Seroresponse

For each of COVID-19 variants, seroresponse is defined as a change of ≥4-fold increase in nAb if baseline value is equal to or above LLOQ or a change in nAb from below LLOQ to equal to or above LLOQ if the baseline value is below LLOQ.

2.6. Covariates

The comparison of immunogenicity data between different intervent groups will be adjusted by stratification foctors used in the randomization as well as the baseline level before the randomization, i.e., :

For Cohort 1 immugenicity subgroup, the comparison will be adjusted by below covariates:

- Age group
- Number of doses of COVID-19 vaccine previously received
- Interval from last COVID-19 vaccination
- Baseline nAb level

For Cohort 2, the comparison will be adjusted by below covariates:

- Age group
- Number of doses of COVID-19 vaccine previously received
- Previous COVID-19 infection history
- Interval from last COVID-19 vaccination
- Baseline nAb level

Of note, in case of wrong selection of the randomization strata in the IVRS system, the covarite will be re-derived based on the information that confirmed to be correct, and the derived one will be used as the covariate. Specially, the interval from last COVID-19 vaccination will be re-drived using below rule:

Interval (in days) = date of randomization – date of last COVID-19 vaccination

- 3-5 months: interval < 6*30
- 6-8 months: 6*30 <= interval < 9*30
- 9-12 months: 90*30 <= interval <=12*30
- 13-24 months: interval >120*30

In case there are very few participants in some stratums, which is leading to non-convergence of the regression model or non-robust parameter estimatioin, the covariate list could be adjusted by grouping those stratums with few participants with near levels.

3. ANALYSIS SETS

According to the objectives of the study, the analysis sets are defined as in Table 3.

The baseline characteriscs of studied population will be described based on ITT population according to the vaccine group assigned by the randomization.

The primary analysis of immunogenicity data will be based on I-PPS population according to the vaccine group that the participant actually recieved. Supplimental analysis will be provided based on I-FAS population.

The safety assessment will be based on Safety Set according to the vaccine group that the participant actually recieved.

Table 3. Analysis Sets

Analysis set	Critieria
Intent-To-Treat Set (ITT)	All randomized participants
Full Analysis Set (FAS)	Randomized participants who received the study vaccine
Per-Protocol Set (PPS)	Participants from FAS set and without major protocol deviations, as determined and documented by Sponsor prior to database lock and unblinding, that impact the immunogenicity data
Safety Set	Randomized participants who received the study vaccine
Immunogenicity per-	Participants from FAS set and with a valid immunogenicity test

protocol set (I-FAS)	result at baseline and at least 1 valid result after receiving the study vaccine
Immunogenicity per-protocol set (I-PPS)	Participants from PPS set and with a valid immunogenicity test result at baseline and at least 1 valid result after receiving the study vaccine

4. GENERAL METHODOLOGY AND CONVENTIONS

4.1.1. Statistical Hypotheses

For **Cohort 1** immunogenicity assessment, let nAb result be denoted as below:

- $GMTC1_{Delta}$= GMT of nAb against Delta variant on D28 of SCTV01C
- $GMTE1_{Delta}$= GMT of nAb against Delta variant on D28 of SCTV01E
- $GMTS1_{Delta}$= GMT of nAb against Delta variant on D28 of Sinopharm inactivated vaccine
- $GMTC1_{Omicron1}$= GMT of nAb against Omicron BA.1 variant on D28 of SCTV01C
- $GMTE1_{Omicron1}$ = GMT of nAb against Omicron BA.1 variant on D28 of SCTV01E
- $GMTS1_{Omicron1}$ = GMT of nAb against Omicron BA.1 variant on D28 of Sinopharm inactivated vaccine
- $GMTC1_{Omicron5}$= GMT of nAb against Omicron BA.5 variant on D28 of SCTV01C
- $GMTE1_{Omicron5}$ = GMT of nAb against Omicron BA.5 variant on D28 of SCTV01E
- $GMTS1_{Omicron5}$ = GMT of nAb against Omicron BA.5 variant on D28 of Sinopharm inactivated vaccine

For **Cohort 2** immunogenicity assessment, let nAb result be denoted as below:

- $GMTC2_{Delta}$= GMT of nAb against Delta variant on D28 of SCTV01C
- $GMTE2_{Delta}$= GMT of nAb against Delta variant on D28 of SCTV01E
- $GMTM2_{Delta}$= GMT of nAb against Delta variant on D28 of mRNA COVID-19 vaccine
- $GMTC2_{Omicron1}$= GMT of nAb against Omicron BA.1 variant on D28 of SCTV01C
- $GMTE2_{Omicron1}$= GMT of nAb against Omicron BA.1 variant on D28 of SCTV01E

- $GMTM2_{Omicron1}$= GMT of nAb against Omicron BA.1 variant on D28 of mRNA COVID-19 vaccine
- $GMTC2_{Omicron5}$= GMT of nAb against Omicron BA.5 variant on D28 of SCTV01C
- $GMTE2_{Omicron5}$= GMT of nAb against Omicron BA.5 variant on D28 of SCTV01E
- $GMTM2O_{micron5}$= GMT of nAb against Omicron BA.5 variant on D28 of mRNA COVID-19 vaccine

For comparison of the immunogenicity response between vaccine groups, the null hypotheses are as below:

For Cohort 1:

- **H11**: $GMR13=GMTE1_{Omicron1}/GMTS1_{Omicron1} \leq 1$
- **H12**: $GMR14=GMTC1_{Omicron1}/GMTS1_{Omicron1} \leq 1$
- **H13**: $GMR12= GMTE1_{Delta}/GMTS1_{Delta} \leq 1$
- **H14**: $GMR12= GMTE1_{Delta}/GMTS1_{Delta} \leq 1$
- **H15**: $GMR15=GMTE1_{Omicron5}/GMTS1_{Omicron5} \leq 1$
- **H16**: $GMR16=GMTC1_{Omicron5}/GMTS1_{Omicron5} \leq 1$

For Cohort 2:

- **H21**: $GMR22=GMTE2_{Omicron1}/GMTM2_{Omicron1} \leq 0.67$
- **H22**: $GMR24=GMTC2_{Omicron1}/GMTM2_{Omicron1} \leq 0.67$
- **H23**: $GMR21= GMTE2_{Delta}/GMTM2_{Delta} \leq 0.67$
- **H24**: $GMR23= GMTC2_{Delta}/GMTM2_{Delta} \leq 0.67$
- **H25**: $GMR26= GMTE2_{Omicron1}/GMTM2_{Omicron1} \leq 1$
- **H26**: $GMR28=GMTC2_{Omicron1}/GMTM2_{Omicron1} \leq 1$
- **H27**: $GMR29=GMTE2_{Omicron5}/GMTM2_{Omicron5} \leq 0.67$
- **H28**: $GMR210=GMTC2_{Omicron5}/GMTM2_{Omicron5} \leq 0.67$
- **H29**: $GMR211= GMTE2_{Omicron5}/GMTM2_{Omicron5} \leq 1$
- **H210**: $GMR212=GMTC2_{Omicron5}/GMTM2_{Omicron5} \leq 1$
- **H211**: $GMR25=GMTE2_{Delta}/GMTM2_{Delta} \leq 1$
- **H212**: $GMR27= GMTC2_{Delta}/GMTM2_{Delta} \leq 1$

4.2. General Methods

4.2.1. General Presentation consideration

Immunogenicity data will be summarized by providing number of observations (n), GMT, 95% CI of the GMT, Median, Q2, Q3, minimum, maximum. For post vaccination time points, fold increase along with 95% CI will be provided as well.

Continuous data other than immunogenicity will be summarized in terms of the number of observations (n), mean, standard deviation (SD), median, minimum and maximum.

Categorical data will be summarized in terms of the number of subjects providing data at the relevant time point (n), frequency counts and percentages.

4.2.2. Methods to Manage Missing Data

Immnogenicity data

The immunogenicity missing data will NOT be imputed. In case the result is LLOQ, half of the LLOQ will be used for analysis.

For a given post vaccination timepoint, the seroresponse rate will be assessed based on participants with both valid baseline value and valid value at the corresponding post vaccination visit.

Adverse event (AE)

For partial or missing onset date of AE, the date will be handled as followed:

- If only Day is missing, the onset date will be imputed as the first date of that month, unless the year and month of the onset date are same as that of vaccination and the AE end date (or ogoing) is on/after the vaccination, in which the onset date will be imputed as same as vaccination date.
- If Day and month are both missing, the onset date will be imputed as the first date of that year, unless the year of the onset date are same as that of vaccination and the AE end date (or ogoing) is on/after the vaccination, in which the onset date will be imputed as same as vaccination date.
- If Day, Month and Year are all missing, the onset date will not be imputed.

Partial or missing end date of AE will not be imputed.

Prior/concomitant medicaitons

The partial or missing start date of medication will be handled in the same approach as the onset date of adverse event.

The partial or missing end date of the medication will not be imputed.

4.3. Multiplicity

For each Cohort, the type I error rate will be controlled at one-sided 0.025 by utilizing a fixed sequency method[1].

For Cohort 1, the hypothesis tests, as noted in **Section 4.1**, will be tested in an order of H11, H12, H13, H14, H15 and H16.

For Cohort 2, the hypothesis tests, as noted in **Section 4.1**, will be tested in an order of H21, H22, H23, H24, H25, H26, H27, H28, H29, H210, H211, and H212

The following test will be tested only when the previous one reaches the statistical significance at one-sided significance level of 0.025.

5. ANALYSES AND SUMMARIES

Unless otherwise specified, the following intervention groups will be used for summary:

- BBIBP-CorV (for Cohort 1 only)
- SCTV01C 20 μg
- SCTV01E 30 μg
- BNT162b2 (for Cohort 2 only)

5.1. Baseline and Other Summaries and Analyses

Baseline information will be summarized based on the ITT population.

5.1.1. Study Conduct and Participant Disposition

For all screened participatns, following categories will be summarized based on participatns screened:

- Number of participatns screened
- Number and percentage of screen failure participatns and the reason for screen failure

Also, the number and percentage of participatns in the following categories will be presented based on ITT population:

- Full Analysis Set (FAS)
- Per-Protocol Set (PPS)
- Safety Set
- Immunogenicity Full Analysis Set (I-FAS)
- Immunogenicity Per-Protocol Set (I-PPS)

The number and percentage of participatns in each of the following disposition categories will be summarized based on ITT population:

- Completed Day 28
- Completed the study
- Prematurely discontinued from the study and the reason for discontinuation

For all participatns prematurely discontinued from the study, the reason will be listed.

5.1.2. Demographics and Baseline Characteristics

Descriptive statistics will be calculated for the following continuous demographic and baseline characteristics: age (years), weight (kg), height (cm), body mass index (BMI) (kg/m 2)). The number and percentage of subjects will be provided for categorical variables such as sex, race, ethnicity, occupation.

Also, below baseline characteristics will be summarized by providing number and percentage of subjects in each category:

- Age group (18 – 54, >= 55 years old)

- Number of doses of COVID-19 vaccine previously received (0, 1, 2, 3)
- Time interval from last COVID-19 vaccine (3-5, 6-8, 9-12, 13-24 months)
- Previous COVID-19 infection history (yes or no)

5.1.3. Medical history

Medical history will be coded by system organ class (SOC) and preferred term (PT) using the Medical Dictionary for Regulatory Activities (MedDRA).

The number and percentage of participants with any medical history will be summarized by SOC and PT based on ITT Set. A participant will be counted only once for multiple events within each SOC and PT. SOC will be displayed in internationally agreed order. PT will be displayed in descending order of frequency of SCTV01E.

5.1.4. Concomitant Medications and Nondrug Treatments

Prior and concomitant medications and non-study vaccinations will be coded using the WHO drug dictionary (WHODD).

The number and percentage of subjects using concomitant medications and non-study vaccinations during the 7-day follow-up period (i.e. on the day of injection and the 6 subsequent days), during the 28-day follow-up period after the vaccination (i.e. on the day of injection and the 27 subsequent days) and during the study (i.e., on the day of injection and subsequent days until end of the study) will be summarized by intervention group.

All concomitant mediation taken during the 28-day follow-up period will be listed.

5.2. Study Treatment Exposure

The vaccination data will be listed for each participant.

5.3. Efficacy Analyses

Unless otherwise specified, the efficacny analysis will be performed using the i-FAS set and i-PPS set. The main conclusion for immunogenicity analysis will be based on the i-PPS set.

5.3.1. Primary Analysis

The primary objective is to evaluate the immunogenicity of the study vaccines, i.e., SCTV01C and SCTV01E. To address this objective the primary Estimand[2] are defined in **Table 4** and

Table 5 for Cohort 1 and Cohort 2 respectively. In case of intercurrent event of COVID-19 infection or non-study drug/vaccine administration that impacts the immunogenicity assessment up to Day 28 after the vaccination, a principle stratum strategy will be utilized in which participants will excluded from the main estimation.

Table 4. Estimand for Cohort 1 Immunogenicity Assessment

Population	Population aged ≥18 years previously vaccinated with inactivated vaccine
Treatment	Arm 1: one dose of SCTV01C vaccine on Day 0<br><br>Arm 2: one dose of SCTV01E vaccine on Day 0<br><br>Arm 3: one dose of Sinopharm inactivated COVID-19 vaccine on Day 0
Variables	Neutralizing antibody titers against Delta/Omicron variant on Day 28 after the vaccination
Intercurrent events and Handling Strategies	**Intercurrent Event 1: COVID-19 infection up to Day 28 after first vaccination:** A principal stratum strategy will be used, i.e., the participants who are diagnosed with COVID-19 up to Day 28 after first vaccination are excluded from this estimand.<br><br>**Intercurrent Event 2: Receiving of other drugs or vaccines that will modify the immunity against Delta or Omicron variant up to Day 28 after first vaccination:** A principal stratum strategy will be used, i.e., the participants who receive other drugs or vaccines that will modify the immunity against Delta or Omicron variant up to Day 28 after first vaccination are excluded from this estimand.
Population-level summary	Ratio of geometric means of the neutralizing antibody titers

Table 5. Estimand for Cohort 2 Immunogenicity Assessment

Population	Population aged ≥18 years previously vaccinated with mRNA vaccine
Treatment	Arm 1 : one dose of SCTV01C vaccine on Day 0

	Arm 2: one dose of SCTV01E vaccine on Day 0<br><br>Arm 3: one dose of BNT162b2 mRNA COVID-19 vaccine on Day 0
Variables	Neutralizing antibody titers against Delta/Omicron variant on D28 after the vaccination
Intercurrent events and Handling Strategies	**Intercurrent Event 1: COVID-19 infection up to Day 28 after first vaccination:** A principal stratum strategy will be used, i.e., the participants who are diagnosed with COVID-19 up to Day 28 after first vaccination are excluded from this estimand.<br><br>**Intercurrent Event 2: Receiving of other drugs or vaccines that will modify the immunity against Delta or Omicron variant up to Day 28 after first vaccination:** A principal stratum strategy will be used, i.e., the participants who receive other drugs or vaccines that will modify the immunity against Delta or Omicron variant up to Day 28 after first vaccination are excluded from this estimand.
Population-level summary	Ratio of geometric means of the neutralizing antibody titers

In details, the immunogenicity nAb again Delta and Omicron at Day 28 for each intervention group will be summarized based on i-PPS set by providing below descriptive statistics:

- Geometric mean titer (GMT) or value with corresponding 95% CI will be provided at each time point. The 95% CIs will be calculated based on the t-distribution of the log-transformed values then back transformed to the original scale for presentation. The following descriptive statistics will be also provided at each time point: the number of subjects (n), median, minimum and maximum.

- Geometric mean fold increase with corresponding 95% CI will be provided at each post-baseline timepoint over pre-injection baseline at Day 0. The 95% CIs will be calculated based on the t-distribution of the log-transformed values then back transformed to the original scale for presentation. The following descriptive statistics will be also provided at each time point: the number of subjects (n), median, minimum and maximum.

In addition, the quantitative immunogenicity data in log-transformed scale will be analyzed using analysis of covariance (ANCOVA) model with covariates as the intervention group, age group, number of doses of COVID-19 vaccine received, interval from last COVID-19 vaccination, previous COVID-19 infection history (Cohort 2 only), and baseline values (in log-transformed scale). The Least Square Goemetric Mean with 95% CI for each intervention group, GMR with 95% CI for treatment difference between study vaccine and the active control (BBIBP-CorV for Cohort 1 and BNT162b2 for Cohort 2) will be estimated from the ANCOVA model.

5.3.1.1. Sensitivity/Supplementary Analyses

As a supportive analysis, the analysis of immunogenicity nAb will be based on i-FAS set regardless of the COVID-19 infection or administration of other drugs or vaccines that will modify the immunity against Delta or Omicron variant up to Day 28 after first vaccination.

5.3.1.2. Subgroup Analyses

Below subgroup analysis will be performed for the primary efficacy endpoint based on i-PPS population:

- Age group: 18-54 and >=55 years old
- Gender (female, male)
- Race
- Ethnicity
- Number of previous COVID-19 vaccines (0, 1, 2, 3)
- COVID-19 infection history (Yes, No)
- Time interval between first dose of IP and the last COVID-19 vaccine (3-5, 6-8, 9-12, 13-24)
- Baseline nAb level (<20, 20 – 80, >80 for Omicron BA.1 and BA.5; Delta for <=20, 20-80, >80-320, and >320)

5.3.2. Analyses of Secondary Efficacy Endpoints

Seroresponse rate, as defined in Section 2.5, will be summarized by providing number and percentage of participants with seroresponse for each post vaccination time point by intervention group. The 95% CI of response rate for each intervention group will be estimated based on

Clopper-Pearson exact method. The rate difference and P value for comparison from Cochran–Mantel–Haenszel test (CMH) stratified by strafication factors will be provided.

Other secondary endpoints based on quantitative immunogenicity assays will by summarized at each assessment time point by intervention group in a similar approach as that in the primary analysis. Specially, in the analysis of cellular immune response paramters, e.g., IFN-γ and IL-4, the comparison between study vaccine and active control will be based on Mann–Whitney U test.

5.4. Safety Summaries and Analyses

Unless otherwise specified, the analysis of safety data will be based on safety set according to treatment group as defined in Section 5.

5.4.1. Adverse Events

Adverse events will be coded using the Medical Dictionary for Regulatory Activities (MedDRA) Version valid at time of database lock.

Treatment-emergent adverse events (TEAE) are defined as those adverse events that either start or worsen in severity on or after the date/time of study vaccination. Where dates are missing or partially missing, adverse events will be assumed to be treatment-emergent, unless there is clear evidence (through comparison of partial dates) to suggest otherwise.

Overview of TEAE

For each study, an overall summary of TEAEs including the number and percentage of subjects who experience the following will be presented:

- Any TEAEs
- Any TEAEs within 30 minutes after study vaccination
- Any TEAEs within 7 days after study vaccination
- Any TEAE within 28 days after study vaccination
- Any vaccination-related AEs
- Any vaccination-related AEs (grade 3 or above)

- Any TEAE leading to premature study discontinuation
- Any SAEs
- Any AESI
- Any TEAEs leading to death

Solicited AEs

The solicited AEs consist of two part, i.e., local AEs and systemic AEs, as defined in Section **2.4.1.**

The number and percentage of subjects who reported each individual solicited local AE and solicited systemic AE during the 7 days after the vaccination will be provided by severity grade.

TEAE by primary System Organ Class (SOC) and Preferred Term (PT)

The following summary tables of TEAEs will be provided by SOC and PT using frequency counts and percentages:

- All unsolicited AEs from Day 0 to Day 28 after the study vaccination
- All TEAEs including both solicited and unsolicited AEs Day 0 to Day 28 after the study vaccination
- All TEAEs related to study vaccine
- All TEAEs leading to premature study discontinuation
- All SAEs
- All AESIs

TEAE by primary System Organ Class, Preferred Term and severity grade

Tables of TEAEs will be provided by SOC, PT and severity grade using frequency counts and percentages for same setting of TEAEs by SOC and PT.

5.4.2. Deaths

All death will be listed by providing all relevant AE information.

5.4.3. Analysis of Other Safety Data

Other safety parameters, e.g., vital sign, physical examination, laboratory assessments (if any) will be listed only.

6. INTERIM ANALYSES

For each cohort, once the safety data within 28 days and immunogenicity data on Day 28 were acquired, it will be analyzed by unblinded team who are independent to the study operation team and are not directly involved in the study activities. The result will be further used for submission to regulatory authority. The specific analysis time point may be adjusted according to the progress of the trial.

7. CHANGES IN THE CONDUCT OF THE STUDY OR PLANNED ANALYSIS

Not applicable.

8. REFERENCES

ICH E9 Statistical Principles for Clinical Trials

ICH E9R1 E9(R1) Statistical Principles for Clinical Trials: Addendum: Estimands and Sensitivity Analysis in Clinical Trials
